# Variation in fungal microbiome (mycobiome) and aflatoxins during simulated storage of in-shell peanuts and peanut kernels

**DOI:** 10.1038/srep25930

**Published:** 2016-05-16

**Authors:** Fuguo Xing, Ning Ding, Xiao Liu, Jonathan Nimal Selvaraj, Limin Wang, Lu Zhou, Yueju Zhao, Yan Wang, Yang Liu

**Affiliations:** 1 Institute of Food Science and Technology, Chinese Academy of Agricultural Sciences/Key Laboratory of Agro-Products Processing, Ministry of Agriculture, P.R. China.

## Abstract

Internal transcribed spacer 2 (ITS2) sequencing was used to characterize the peanut mycobiome during 90 days storage at five conditions. The fungal diversity in in-shell peanuts was higher with 110 operational taxonomic units (OTUs) and 41 genera than peanut kernels (91 OTUs and 37 genera). This means that the micro-environment in shell is more suitable for maintaining fungal diversity. At 20–30 d, *Rhizopus*, *Eurotium* and *Wallemia* were predominant in in-shell peanuts. In peanut kernels, *Rhizopus* (>30%) and *Eurotium* (*>*20%) were predominant at 10–20 d and 30 d, respectively. The relative abundances of *Rhizopus*, *Eurotium* and *Wallemia* were higher than *Aspergillus*, because they were xerophilic and grew well on substrates with low water activity (*a*_w_). During growth, they released metabolic water, thereby favoring the growth of *Aspergillus*. Therefore, from 30 to 90 d, the relative abundance of *Aspergillus* increased while that of *Rhizopus*, *Eurotium* and *Wallemia* decreased. Principal Coordinate Analysis (PCoA) revealed that peanuts stored for 60–90 days and for 10–30 days clustered differently from each other. Due to low *a*_w_ values (0.34–0.72) *a*nd low levels of *A. flavus*, nine of 51 samples were contaminated with aflatoxins.

Peanuts are important economic crops in the world and are cultivated on a large scale, with Africa, China and India being the greatest producers. In 2013, the annual peanut yield in China was 17.0 million tons (http://data.stats.gov.cn/easyquery.htm?cn=C01), which accounts for more than 45% of the worldwide peanut production (http://faostat.fao.org). Peanut kernels have high nutritional and commercial value due to the presence of calcium, carbohydrates, fatty acids, fibers, phosphorus, proteins and vitamins. Peanuts are mainly used in the manufacture of oil, sweets, candies and pastes. More than 60% of the worldwide peanut yield is destined to oil production, with peanut oil being the fifth most consumed type of oil around the world^1^.

The contamination of peanuts with *Aspergillus flavus* and aflatoxins is considered to be one of the most serious safety problems in the world^2^,^3^. This fungal pathogen infects peanut kernels before and after harvest^4^,^5^. Pre-harvest peanut kernels contain mycelia and spores of aflatoxigenic fungi, which contribute to significant economic losses and aflatoxin accumulation during storage^6^. Contamination with aflatoxins compromises the quality of the product because aflatoxins are the most toxic and carcinogenic compounds among the toxins. Aflatoxin B_1_ (AFB_1_), which is the most toxic, has both teratogenic and mutagenic properties and causes toxic hepatitis, hemorrhage, edema, immunosuppression, and hepatic carcinoma^7^. AFB_1_, which poses a health risk in animals and humans, has been classified as a class I human carcinogen by the International Agency for Research on Cancer^8^,^9^. More than 100 countries and organizations including the National Health and Family Planning Commission P.R. of China, the European Union and the U.S. Food and Drug Administration, have established limits for total aflatoxins and AFB_1_ levels in peanuts^10^,^11^.

*A. flavus* growth and subsequently aflatoxin production depend on several factors, including geographical region, season, and the environmental conditions during peanut growth and storage. Tropical and subtropical regions are the most favorable for *A. flavus* growth and aflatoxin production^12^. *A. flavus* growth and aflatoxin production are markedly affected by environmental factors, especially water activity (*a*_w_) and temperature^13^. The Yangtze River zone, especially Hubei province, China, is the major peanut-producing region of the country. However, this region has a subtropical climate with high temperatures and high relative humidity, which is the favorable environmental condition for *A. flavus* growth and aflatoxins production in stored peanuts. In 2012, the distribution and toxigenicity of *A. flavus* and *A. parasiticus* in peanut soils of four agroecological zones in China (Southeast coastal zone, Yangtze River zone, Yellow River valley and Northeast zone) were investigated in our laboratory. Previous findings revealed that Yangtze River zone had the highest concentration of *A. flavus* (2749.3 CFU/g) and the highest toxigenic potential of aflatoxin production among the four agroecological zones. It is not surprising that peanut contamination with aflatoxins is frequently reported in this region^3^,^14^. However, there are other fungal population that can affect *A. flavus* growth and aflatoxin production. Therefore, it is necessary to characterize the fungal microbiome of peanuts and its variations during storage under the environmental conditions of this region.

High-throughput sequencing technologies have opened new frontiers in microbial community analyses by providing an economic and efficient means of identifying the microbial phylotypes in samples. Furthermore, next-generation sequencing techniques have led to a revolution in microbial ecology by providing opportunities to generate unprecedented numbers of sequences and detect rare or low-abundance organisms^15^,^16^. Studies have revolutionized our understanding of the microbial communities present in our bodies^17^,^18^,^19^,^20^,^21^, soils^22^,^23^ and deep seas^24^. This revolution in sequencing technology, coupled with the development of advanced computational tools that exploit metadata to relate hundreds of samples to one another in ways that reveal clear biological patterns, has re-invigorated studies focused on the internal transcribed spacer 2 (ITS2) region of rDNA. The ITS2 region, which is an excellent phylogenetic marker suitable for fungal taxon assignment^25^, has been successfully used in comparative ecology studies where it gives results that are convergent with, if not comparable to, those for other markers^25^,^26^. ITS-based surveys are extremely valuable because they allow the assessment of biodiversity and ecological characteristics of whole communities or individual microbial taxa. However, alternative techniques, such as metagenomics can provide insight into all genes and their functions in a given community. ITS2 region phylogenies tend to match trends in overall gene content; the ability to relate at the species level to the host or the environmental parameters has proven immensely powerful.

In the present study, the barcoded Illumina paired-end sequencing (BIPES) technique was used to characterize the mycobiome and its variations in stored in-shell peanuts and peanut kernels under different conditions (i.e. temperature and relative humidity). This study characterizes the mycobiome and its variation in stored peanuts using ITS2 sequencing and provides a direct comparison of the peanut mycobiome diversity during simulated storage. The findings of this study are likely to encourage the implementation and design of mould and aflatoxin management strategies.

## Results

### Data characteristics

In in-shell peanuts, the average number of raw reads generated per sample was 84,834, of which 56,709 were retained following filtering and denoising steps, and 54,426 reads were subsequently clustered into different operational taxonomic units (OTUs). The average length of each read was 329 bp (range: 307–348 bp) (Table 1). Of 245 OTUs, 6 OTUs cannot be identified with other organisms, 23 OTUs represent peanut and other plants, 14 OTUs represent nematode and other animals, and 3 OTUs represent *E. coli*. Remaining 199 OTUs represent fungi, and of them 18 OTUs represent uncultured fungi (Table S1).

In peanut kernels, the average number of raw reads generated per sample was 61,243, of which 38,396 were retained following filtering and denoising steps, and 36,718 reads were subsequently clustered into 196 OTUs. The average length of each reads was 331 bp (range: 311–363 bp) (Table 1). Of 196 OTUs, 2 OTUs cannot be identified with other organisms, 21 OTUs represent peanut and other plants, 13 OTUs represent nematode and other animals, and 7 OTUs represent bacteria. Remaining 153 OTUs represent fungi, and of them 13 OTUs represent uncultured fungi (Table S2).

### Fungal diversity in in-shell peanuts and peanut kernels

The average number of OTUs detected per sample was 110 (range: 81–140). On average, each OTU contained 495 reads. OTUs representing 41 genera of fungi were detected. 61.1% of OTUs and 50.7% of reads belonged to the Ascomycota, 9.4% of OTUs and 13.0% of reads belonged to Basidiomycota, and 7.1% of OTUs and 23.7% of reads belonged to Zygomycota. Dominant orders among Ascomycota included Dothideomycetes, Eurotiomycetes, Saccharomycetes and Sordariomycetes. Dominant Eurotiomycetes groups included the genera *Aspergillus* (12.8% of OTUs and 12.1% of reads), *Eurotium* (4.8% of OTUs and 21.6% of reads) and *Penicillium* (7.7% of OTUs and 10.1% of reads) genera. Dominant genera among Zygomycota and Basidiomycota were *Rhizopus* (5.2% of OTUs and 23.5% of reads) and *Wallemia* (5.3% of OTUs and 11.3% of reads), respectively (Table 2). *Aspergillus* species that found were *Aspergillus aculeatus, Aspergillus candidus, Aspergillus flavipes, A. flavus, Aspergillus gracilis, Aspergillus niger, Aspergillus ochraceus, Aspergillus penicillioides* (7.19% of reads)*, Aspergillus restrictus, Aspergillus tamarii, Aspergillus versicolor, Aspergillus vitricola* and *Aspergillus wentii*. The predominant species in *Eurotium* was *Eurotium niveoglaucum* (39.78% of reads). The predominant species in *Penicillium* were *Penicillium citrinum*, *Penicillium pinophilum*, and *Penicilliium simplicissium*. In *Wallemia*, *Wallemia sebi* was the only species. The predominant species in *Rhizopus* was *Rhizopus oryzae* (46.16% of reads) (Table S1).

The average number of OTUs detected per sample was 91 (range: 69–114). On average, each OTU contained 403 reads. OTUs representing 37 genera of fungi were detected. 60.5% of OTUs and 44.2% of reads belonged to the Ascomycota, 9.7% of OTUs and 13.5% of reads belonged to Basidiomycota, and 7.8% of OTUs and 27.8% of reads belonged to Zygomycota. Dominant orders among Ascomycota were Dothideomycetes, Eurotiomycetes, Saccharomycetes and Sordariomycetes. Dominant Eurotiomycetes groups included the genera *Aspergillus* (14.4% of OTUs and 10.1% of reads), *Eurotium* (6.1% of OTUs and 19.6% of reads) and *Penicillium* (8.4% of OTUs and 9.7% of reads). Dominant genera among Zygomycota and Basidiomycota were *Rhizopus* (5.9% of OTUs and 27.8% of reads) and *Wallemia* (6.8% of OTUs and 13.4% of reads) (Table 2). The species in *Aspergillus* were *A. aculeatus*, *Aspergillus ellipticus, A. flavipes, A. flavus, Aspergillus glaucus, A. niger* (10.50% of reads), *A. ochraceus, A. penicillioides* (2.05% of reads), *A. restrictus, A. tamarii, Aspergillus terreus, A. versicolor* and *A. vitricola*. The predominant species in *Eurotium* was *E. niveoglaucum* (34.35% of reads). The predominant species in *Penicillium* were *P. citrinum*, *P. pinophilum*, and *P. simplicissium*. In *Wallemia*, *W. sebi* and *Walleia* sp. F53 were the main species. The predominant species in *Rhizopus* was *R. oryzae* (48.79% of reads) (Table S1).

### Storage time and fungal diversity

**Figure 1:**
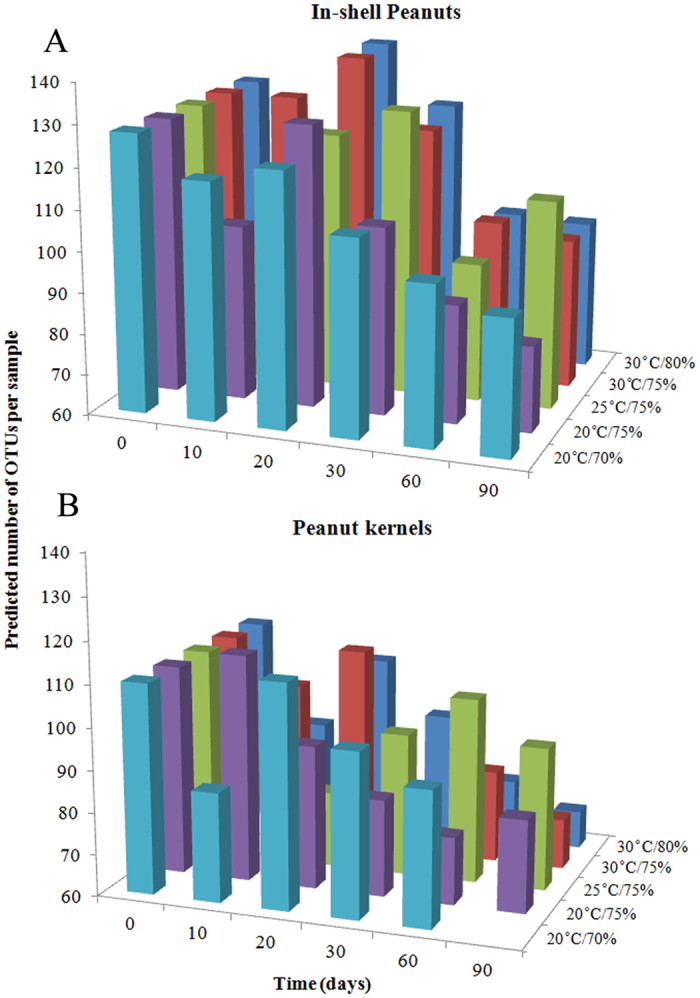
Predicted number of operational taxonomic units (OTUs) per sample in in-shell peanuts (**A**) and peanut kernels (**B**) stored for 0–90 d at different conditions.

There were significant variation in per-sample OTUs richness based on storage conditions and storage time (Fig. 1). In in-shell peanuts, all denoised reads were clustered into 245 OTUs using a minimum pair-wise identity of 97%. The average number of OTUs detected per sample in in-shell peanuts was 110 (range: 81–140). In general, OTU number decreased at 10 d, reached its highest value at 20 d, and subsequently decreased, except in samples stored at 25 °C with 75% relative humidity. Though there were differences among storage conditions, but no significant difference in the results.

In peanut kernels, all denoised reads were clustered into 196 OTUs using a minimum pair-wise identity of 97%. The average number of OTUs detected per sample in in-shell peanuts was 91 (range: 69–114). In general, the number of OTUs decreased with storage time, and the differences were seen among the storage conditions but not significant (*p* > 0.05).

### Fungal community variation in phylum across storage time

**Figure 2:**
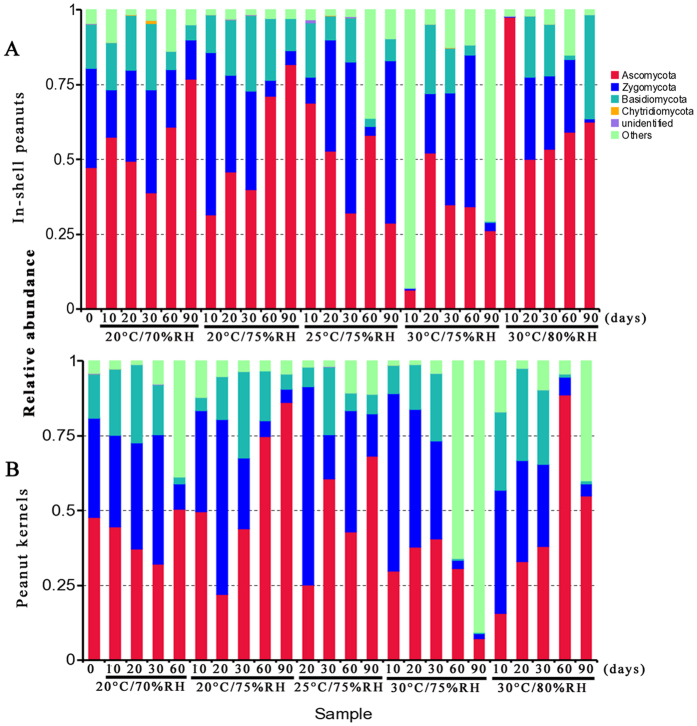
Overall distribution of fungi at phylum level in in-shell peanuts (**A**) and peanut kernels (**B**) stored for 0–90 d at different conditions. RH: relative humidity.

There are obvious variations in the relative abundance of fungal phyla per sample based on storage conditions and storage time (Fig. 2). In in-shell peanuts, four fungal phyla, i.e., Ascomycota, Basidiomycota, Chytridiomycota, and Zygomycota were identified. Of them, Ascomycota, Basidiomycota, and Zygomycota were the predominant phyla, with 50.7%, 13.0% and 23.7% relative abundance, respectively. In peanut kernels, the same four fungal phyla were identified. Of them, Ascomycota, Basidiomycota, and Zygomycota were the main phyla (44.2%, 13.5% and 27.8% relative abundance, respectively).

In in-shell peanuts, the relative abundance of Ascomycota fungi increased from 30 to 90 d at 20 °C with 70% and 75% relative humidity, and from 20 to 90 d at 30 °C with 80% relative humidity. At 25 °C with 75% relative humidity, there were obvious oscillations in the relative abundance of Ascomycota fungi during storage. Similarly, in peanut kernels, there were also obvious oscillations in the relative abundance of Ascomycota fungi, but the regularity was absent.

### Fungal community variation in genus level across storage time

**Figure 3:**
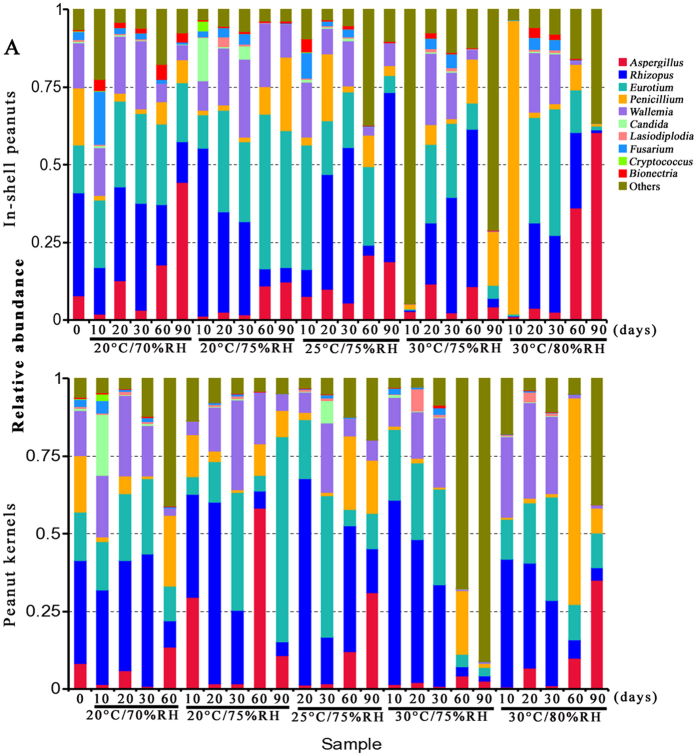
Overall distribution of fungi at genus level in in-shell peanuts (**A**) and peanut kernels (**B**) stored for 0–90 d at different conditions. RH: relative humidity.

There were obvious variation in the relative abundance of fungal genera per-sample based on storage conditions and storage time (Fig. 3). In in-shell peanuts, the average number of clean reads retained after the filtering and denoising steps was 56,709, of which 54,426 reads were subsequently clustered into 110 OTUs and 41 fungal genera. Of them, *Aspergillus*, *Eurotium*, *Penicillium, Rhizopus* and *Wallemia* were the main genera, with average relative abundances of 12.1%, 21.6%, 10.1%, 33.1% and 11.3%, respectively. *Fusarium* had a relative abundance of 2.4%. The relative abundance of *Aspergillus* fungi at 60 and 90 d was significantly higher than that at 10, 20 or 30 d under all storage conditions (*p* < 0.05). In general, the relative abundance of *Aspergillus* fungi increased from 30 to 90 d, except in samples stored at 30 °C with 75% relative humidity. The relative abundance of *Aspergillus* fungi reached its maximum value (60.3%) at 30 °C with 80% relative humidity. At 20 and 30 d, *Eurotium, Rhizopus* and *Wallemia* were the main genera, with relative abundance higher than 15%, 20% and 10%, respectively.

In peanut kernels, the average number of clean reads retained after the filtering and denoising steps was 38,936, of which 36,718 were subsequently clustered into 91 OTUs; 37 fungal genera were identified. Of them, *Aspergillus, Eurotium*, *Penicillium, Rhizopus* and *Wallemia* were the most predominant genera (19.6%, 10.1%, 9.7%, 27.8% and 13.4% relative abundance, respectively). The relative abundance of *Aspergillus* fungi at 60 and 90 d was significantly higher than other days at 10, 20 or 30 d (*p* < 0.05). The relative abundance of *Rhizopus* fungi at 10 and 20 d were high (>30%) and decreased from 30 to 90 d. The relative abundance of *Wallemia* fungi was high at 20 and 30 d but further decreased from 30 to 90 d. The relative abundance of *Eurotium* fungi reached a maximum value at 30 d (> 20%) and subsequently decreased from 30 to 90 d except in samples stored at 20 °C with 75% relative humidity. The relative abundance of *Penicillium* reached the highest level at 60 d.

### Changes in Mycobiome are associated with storage time

**Figure 4:**
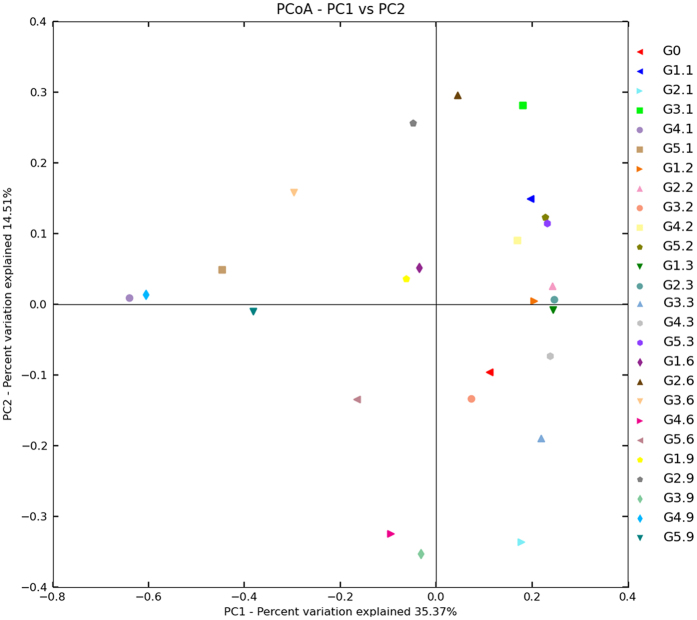
Principal Coordinate Analysis (PCoA) of distribution of fungi in in-shell peanuts during storage. G0: raw peanuts; G1.1, G1.2, G1.3, G1.6, G1.9: stored in-shell peanuts at 10, 20, 30, 60, 90 d with 20 °C/70% RH; G2.1, G2.2, G2.3, G2.6, G2.9: stored in-shell peanuts at 10, 20, 30, 60, 90 d with 20 °C/75% RH; G3.1, G3.2, G3.3, G3.6, G3.9: stored in-shell peanuts at 10, 20, 30, 60, 90 d with 25 °C/75% RH; G4.1, G4.2, G4.3, G4.6, G4.9: stored in-shell peanuts at 10, 20, 30, 60, 90 d with 30 °C/75% RH; G5.1, G5.2, G5.3, G5.6, G5.9: stored in-shell peanuts at 10, 20, 30, 60, 90 d with 30 °C/80% RH. RH: relative humidity.

**Figure 5:**
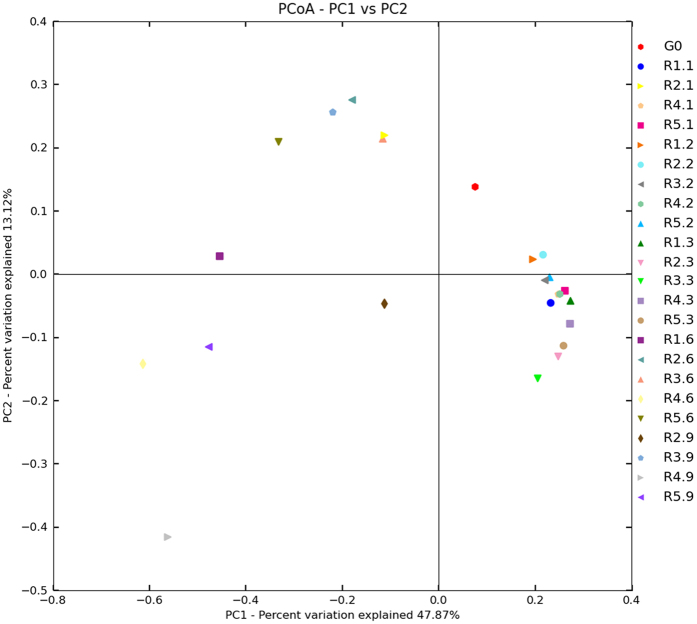
Principal Coordinate Analysis (PCoA) of distribution of fungi in peanut kernels during storage. G0: raw peanuts; R1.1, R1.2, R1.3, R1.6, R1.9: stored in-shell peanuts at 10, 20, 30, 60, 90 d with 20 °C/70% RH; R2.1, R2.2, R2.3, R2.6, R2.9: stored in-shell peanuts at 10, 20, 30, 60, 90 d with 20 °C/75% RH; R3.1, R3.2, R3.3, R3.6, R3.9: stored in-shell peanuts at 10, 20, 30, 60, 90 d with 25 °C/75% RH; R4.1, R4.2, R4.3, R4.6, R4.9: stored in-shell peanuts at 10, 20, 30, 60, 90 d with 30 °C/75% RH; R5.1, R5.2, R5.3, R5.6, R5.9: stored in-shell peanuts at 10, 20, 30, 60, 90 d with 30 °C/80% RH. RH: relative humidity.

To investigate whether there is an association between any of the subject storage parameters and changes in mycobiome, we performed Principal Coordinate Analysis (PCoA). The results revealed that peanuts stored for 60–90 days and peanuts stored for 10–30 days clustered differently from each other (Figs 4 and 5). In in-shell peanuts, all peanuts at 60 and 90 d clustered in the left and PCoA case scores (Bray Curtis) were less than zero, except for G2.6 (from 60 d at 20 °C with 75% relative humidity); all samples at 10, 20 and 30 d clustered in the right and PCoA case scores were more than zero, except for G4.1 (from 10 d at 30 °C with 75% relative humidity) and G5.1 (from 10 d at 30 °C with 80% relative humidity). In peanuts kernels, all samples at 60 and 90 d clustered in the left and PCoA case scores were less than zero; all samples at 10, 20 and 30 d clustered in the right and PCoA case scores were more than zero, except for R2.1 (from 10 d at 20 °C with 75% relative humidity). These suggest a trend in association between storage time and the peanuts mycobiome.

### Aflatoxins in stored peanuts

As shown in Table 3, of 25 in-shell peanuts five (20%) were contaminated with AFB_1_ (ranging from 0.34 to 10.40 μg/kg, four (16%) were contaminated with AFB_2_ (0.10–1.87 μg/kg), one (4%) were contaminated with AFG_1_ (0.72 μg/kg), and two (8%) were contaminated with AFG_2_ (0.15 μg/kg). Of 25 peanut kernels, four (16%) were contaminated with AFB_1_ (0.34–68.79 μg/kg, three (12%) were contaminated with AFB_2_ (0.07–6.25 μg/kg), and one (4%) was contaminated with AFG_2_ (0.24 μg/kg).

## Discussion

The average number of raw reads, clean reads, and taxon reads in in-shell peanuts were 84,834, 56,709 and 54,426, respectively, and 61,243, 38,396 and 36,718 in peanut kernels, respectively. This result suggested that the number of fungi in in-shell peanuts was higher than that in peanut kernels. Furthermore, the total number of fungal OTUs in in-shell peanuts was 199, which was higher than in peanut kernels (OTUs: 153). The average number of OTUs detected per sample in in-shell peanuts was 110 (range 81–140), while that in peanut kernels was 91 (range: 69–114). Furthermore, in in-shell peanuts, 41 fungal genera were identified, which was higher than that in peanut kernels. Fungal diversity of in-shell peanuts was significantly higher than in peanut kernels during storage. This indicates that the micro-environment in peanut shell proves favorable for maintaining fungal diversity.

In general, *Aspergillus*, *Eurotium*, *Penicillium Rhizopus*, and *Wallemia* were predominant genera in both in-shell peanuts and peanut kernels during storage. This result attributes to the greater adaptation of these fungi to the substrate, especially during storage^27^,^28^. The occurrence of *Aspergillus*, *Penicillium*, and *Rhizopus* agrees with the findings of other investigators studying peanut kernels from Brazil^27^,^28^,^29^ and India^30^ using traditional isolation, enumeration and identification methods of the mycoflora on the Dichloran Rose Bengal Chloramphenicol agar (DRBC) or *A. flavus and A. parasiticus* agar (AFPA) media. *Eurotium* was not detected in these studies because *Eurotium* did not grow well on DRBC or AFPA media. Nakai *et al*.^27^ found a predominance of *Fusarium* spp. (67.7% in hulls and 25.8% in kernels) and *Aspergillus* spp. (10.3% in hulls and 21.8% in kernels). In the previous studies, only eight genera were isolated from peanuts kernels and hulls i.e. *Aspergillus*, *Cladosporium, Drechslera, Fusarium*, *Penicillium*, *Phoma*, *Rhizopus*, and *Trichoderma*. However, in our study, 41 and 37 fungal genera were detected in in-shell peanuts and peanut kernels during storage using high-throughput ITS2 sequencing technologies, respectively. The number of fungal genera reported is about 5-folds increase compared to previous studies. This is because the studies of evaluated the mycoflora in stored peanuts using traditional isolation, enumeration and identification provided only a limited snap shot of the fungal members of the microbiome. While, the barcoded Illumina paired-end sequencing (BIPES) method using in this study^31^ provided a more in-depth comprehensive profile of the mycobiome.

**Figure 6:**
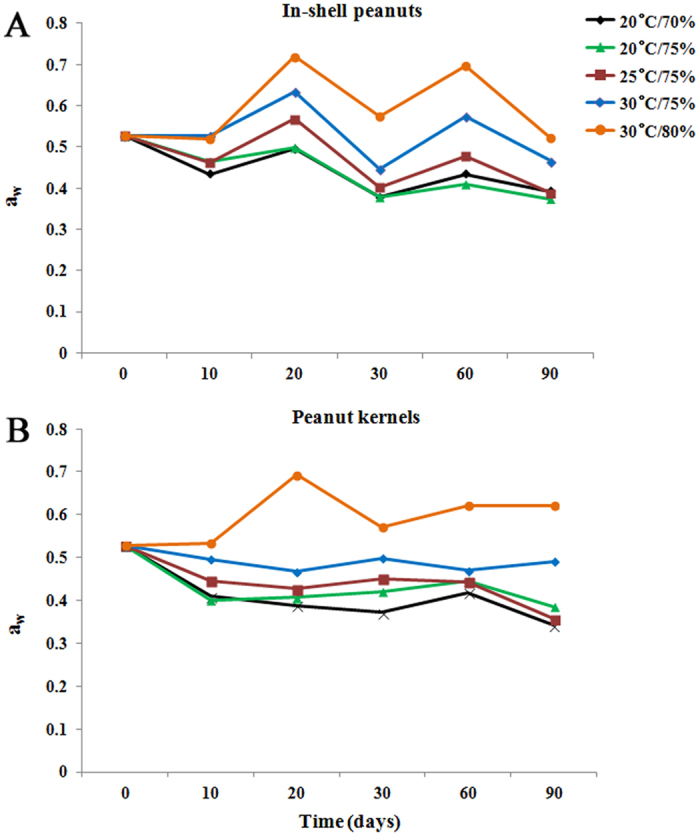
Storage time variation of water activity (*a*_w_) in in-shell peanuts (up) and peanut kernels (below) stored for 0–90 d at different conditions.

At 20–30 d, the relative abundance of *Eurotium*, *Rhizopus* and *Wallemia* were higher than that of *Aspergillus*, because the three genera fungi were xerophilic and grew well on substrates with low *a*_w_ (Fig. 6). During growth, *Eurotium*, *Rhizopus* and *Wallemia* fungi released metabolic water on substrates with low *a*_w_, thereby favoring the growth of *Aspergillus*, which are less xerophilic fungi. Therefore, from 30 to 90 d, the relative abundance of *Aspergillus* increased while that of *Eurotium*, *Rhizopus* and *Wallemia* decreased. Similarly, the results from PCoA analysis showed some tendency for stored peanuts at 60–90 d to cluster together, and stored peanuts at 10–30 d to cluster together. These results suggested that storage time plays a vital role in impacting the variation of mycobiome in stored peanuts. However, given the limitation of lesser study samples in the current study, it is difficult to draw definite conclusions regarding association of the mycobiome with storage time and/or conditions. Therefore, further confirmations on researching larger population sizes were needed.

*Wallemia* is a genus of cosmopolitan xerophilic fungi that are present in several environments characterized by low *a*_w_^32^,^33^, and is frequently involved in food spoilage. In 2006, Sun *et al*.^34^ isolated one *W. sebi* isolate from the surface of apples. This was the first report of its occurrence in China as a saprophyte on foods. In the present study, *Wallemia* was identified in peanuts grown in China. The results confirmed that *Wallemia* grows well on substrates with low *a*_w_ because *a*_w_ of peanuts is ≤0.72 (ranging from 0.36 to 0.72) (Fig. 6). In general, the relative abundance of *Wallemia* in stored in-shell peanuts and peanut kernels were higher at 20–30 d and subsequently decreased from 30 to 90 d with the concomitant increase in *Aspergillus*.

In both in-shell peanuts and peanut kernels during storage, *Aspergillus*, *Eurotium*, *Penicillium, Rhizopus* and *Wallemia* were the predominant genera. Of them, *Rhizopus* was the most abundant genus with a relative abundances >20% as they could grow well on peanuts since it has a low *a*_w_ of ≤0.72. *Rhizopus* is common saprobic fungi on plants and specialized parasites on animals. In general, the relative abundance of *Rhizopus* in stored in-shell peanuts and peanut kernels were high at 20–30 d and decreased from 30 to 90 d with the concomitant increase in *Aspergillus*. *Eurotium* is the teleomorph genus associated with *Aspergillus*, which could grow on substrates with low *a*_w_. These organisms are universally distributed in nature and are usually referred to as halophilic or xerophilic. They cause significant damage in stored grains, cereals and food products preserved by drying or salt/sugar addition^35^,^36^,^37^,^38^. More important, *Eurotium* release metabolic water on substrates with low *a*_w_, thereby creating favorable conditions for less xerophilic fungi (e.g., *A. flavu*s and *A. niger*) that can produce more hazardous mycotoxins (e.g., aflatoxins and ochratoxins). The results of this study confirmed the above findings. The relative abundance of *Aspergillus* (with the exception of *Eurotium*) increased from 30 to 90 d in stored in-shell peanuts and peanut kernels, i.e 2.6% to 60.3%, and 1.1% to 35%, respectively, especially at 30 °C with 80% relative humidity. So we could conclude that rapidly grown *Eurotium* at initial stage could release metabolic water which in turn increases *a*_w_, thereby creating a favorable condition for *Aspergillus* species growth. Most fungi from grains grow well on DRBC, and Pitt and Hocking^37^ reported that DRBC is adequate for the numeration of fungi present in food and feed. *Eurotium* growth was observed on DG18 medium rather than on DRBC. Therefore, *Eurotium* in peanuts should be determined using DG18, which is the medium for xerophilic fungi^32^.

The *a*_w_ in this study (0.37–0.72 in in-shell peanuts and 0.34–0.69 in peanut kernels) (Fig. 6) was below the minimum range of 0.78–0.80 established for the growth of *A. flavus*^39^. The low *a*_w_ in the stored samples is probably due to the previously used process of peanut drying. In the present experiment, the temperature ranged from 20 °C to 30 °C and relative humidity ranged from 70% to 80%. Therefore, the temperature was lower than 32–33 °C, which is the optimum temperature for the growth of *A. flavus*^40^. The relative humidity values in the samples were lower than those measured by Christensen *et al*.^41^, who reported a relative humidity of approximately 83–85%, which favors the growth of *A. flavus*. Due to the low *a*_w_ of stored peanuts, temperature and relative humidity, only nine of the 51 samples were contaminated with aflatoxins at levels ranging from 0.34 to 68.79 μg/kg. And only one sample was contaminated with aflatoxins at levels >20 μg/kg, which is the maximum level allowed by the National Health and Family Planning Commission of the P.R. China for AFB_1_ (http://www.nhfpc.gov.cn/cmsresources/mohwsjdj/cmsrsdocument/doc11939.pdf). The results of aflatoxins are also in accordance with the results of the mycological analysis, because the percentages of *A. flavus*, which are well-known aflatoxin-producing species, are lower than 0.01%.

## Conclusions

The results of this study revealed that there were more genera, species and number of fungi in in-shell peanuts than in peanut kernels, and suggested that the micro-environment in shell was more suitable for maintaining the fungal biodiversity and resist infection of fungi from outer environment. *Aspergillus*, *Eurotium*, *Penicillium, Rhizopus* and *Wallemia* were the predominant genera in both in-shell peanuts and peanut kernels during storage. At 20 to 30 d, *Eurotium*, *Rhizopus* and *Wallemia* were the main genera; however, from 30 to 90 d, their relative abundance decreased and that of *Aspergillus* increased. Due to low *a*_w_ values (0.34–0.72) of stored peanuts, nine of 51 samples were contaminated with aflatoxins, and only one sample had AFB_1_ levels >20 μg/kg. This study identified the mycobiome and its variation in stored peanuts during simulated storage using high-throughput ITS2 sequencing, and provided the basis for a detailed characterization and identification of mycobiome in stored peanuts.

## Methods

### Ethics Statement

Specific permission was not needed for our field studies. The peanuts variety used in our field study was main cultivar name Baisha 1016 in Hubei province. No transgenic or created mutant plant has been used in our study. Also we confirm that the field studies did not involve endangered or protected species.

### Sample preparation

Peanuts were obtained from Xiangyang City, Hubei province, which is in the center of Yangtze River valley. After harvest, the peanuts were transported to Beijing in 25-kg bags (a total of five bags) in 3 d. Half of the peanuts were unshelled (peanut kernels). Both peanut kernels and in-shell peanuts were stored at similar conditions. According to climatic conditions (temperature and relative humidity) of Xiangyang City from April to June, both peanut kernels and in-shell peanuts were stored in a ZXMP-A1230 constant temperature and humidity incubator (Zhicheng, Shanghai, China) at five storage conditions: 20 °C with 70% relative humidity, 20 °C with 75% relative humidity, 25 °C with 75% relative humidity, 30 °C with 75% relative humidity, and 30 °C with 80% relative humidity. Samples were collected at 10, 20, 30, 60 and 90 d of storage and analyzed.

### Total microbiome genomic DNA extraction

Water was sterilized at 121 °C for 30 min, and then filtered through 0.22 μm filters. The sterile water was used as a negative control for the experiment. In this experiment, peanuts kernels (100 g) were separated from the hulls and washed with 100 ml sterile water. Water samples were collected and vacuum-filtered through 0.22 μm filters within 24 hours. Filters containing sample were placed in 50-ml tubes and stored at −20 °C. Genomic DNA was extracted from the filters using the MoBio PowerWater^®^ DNA Isolation Kit (MoBio Laboratories, Inc., Carlsbad, CA, USA) according to manufacturer’s recommendations. The final DNA elution was performed with sterile deionized water instead of the provided buffer. DNA quality and quantity were measured by spectrophotometric quantification in a Beckman DU800 (Beckman, USA) and NanoDrop 1000 (Thermo Fisher Scientific, USA), and by agarose gel electrophoresis. Extracted DNA was stored at −80 °C prior to amplification and sequencing.

### PCR amplification and ITS2 sequencing

PCR was used to amplify the ITS2 region of rDNA. The barcoded primers ITS2F (5′-GCATCGATGAAGAACGCAGC-3′) and ITS2R (5′-TCCTCCGCTTATTGATATGC-3′) were used to amplify fungal ITS2 fragments. PCR reactions were carried out in a total reaction volume of 30 μl consisting 15 μl of Phusion High-Fidelity PCR Master Mix (New England Biolabs Inc. Ipswich, MA, USA), 0.2 μM of forward and reverse primers, and 10 ng of template DNA. The PCR amplification program consisted of a initial heating to 98 °C for 1 min, 30 cycles of denaturation at 98 °C for 10 s, annealing at 50 °C for 30 s and extension at 72 °C for 60 s, followed by a 5-min extension at 72 °C prior to storage at 4 °C. Amplified products were cleaned and purified using the GeneJET Gel Extraction Kit (Thermo Scientific, South Logan, UT 84321, USA) according to the manufacturer’s instructions. Of 51 peanut samples, 49 samples were successfully amplified. Amplicons were then quantified and sequencing libraries were generated using NEB Next Ultra DNA library Prep Kit for Illumina (New England Biolabs Inc. USA) according to manufacturer’s recommendations. The amplicon libraries were subsequently sequenced on an Illumina MiSeq platform at Novogene (Novogene, Beijing, P.R. China).

### Bioinformatics analyses

Paired-end reads from the original DNA fragments were merged by using FLASH^42^. Sequences were analyzed with the QIIME^43^ software package using default parameters for each step. The UCLUST method^44^,^45^ was used to cluster the sequences into OTUs at an identity threshold of 97%. Meanwhile, the RDP Classifier^46^ was used to assign each OTU to a taxonomic level. Other analyses, including rarefaction curves, Shannon index, and Good’s coverage, were performed with QIIME. In addition, the OTU table produced by the QIIME pipeline was imported into MEGAN 4 and mapped on the NCBI taxonomy database^47^. Abundance-based comparisons were therefore made solely within selected taxonomic groups such as *Aspergillus*, *Eurotium*, *Penicillium*, *Rhizopus* and *Wallemia*, using an OTU table that was rarified in QIIME.

PCoA has been recognized as a simple and straight-forward method to group and separate samples in a dataset, and has been used in disease-association, gender-association and ethnicity studies^19^,^48^,^49^. In the current study, PCoA was used to analyze the sequencing results using the Multivariate Statistical Package, MVSP (Kovach, Wales, UK) and SAS (Cary, NC). The PCoA performs an Eigen analysis on the data matrix using a Brays Curtis distance metric.

### Determination of aflatoxins

Aflatoxins levels were determined by Chinese standard methods^50^ and AOAC method 994.08^51^ with minor modifications. In this experiment, peanut kernels (50 g) were manually de-shelled, ground, mixed to obtain peanut paste, and stored at −20 °C until analysis. Finely ground samples (5.0 g) were extracted with 15 ml of acetonitrile:water (84:16, v/v). Following ultrasonic extraction at 50 °C for 10 min and filtration through double-layer slow quantitative filter paper, 4 ml of the resulting filtrate was mixed with 2 ml petroleum ether. The mixture was mixed on a vortex for 30 s and allowed to stand for 15–20 min. The lower layer (3 ml) was collected, mixed with 8 ml pure water and filtered through a 0.45 μm organic membrane. Extracts (8 ml) were passed through immunoaffinity columns with a flow rate of one droplet per second and eluted with 2 ml of methanol into glass tubes. The eluate was evaporated to dryness under a stream of nitrogen gas at 60 °C. The purified extract was re-dissolved with 1 ml of acetonitrile : water (15 : 85, v/v). The resulting supernatant was collected in glass tubes for high-performance liquid chromatography (HPLC) quantitative analysis.

The determination of aflatoxin levels was performed by HPLC. HPLC analysis was performed with a Waters 2695 (Waters Corporation, Milford, MA, USA) coupled to a Waters 2475 fluorescence detector (λexc 360 nm; λem 440 nm) and a post-column derivation system, and an Agilent TC-C18 column (250 × 4.6 mm, 5 μm particle size). The mobile phase (water : methanol : acetonitrile, 4 : 1 : 1) was pumped at a flow rate of 0.5 mL/min. AFB_1_, B_2_, G_1_ and G_2_ (Sigma-Aldrich, St. Louis, MO, USA) were used as standards. The mean recovery of the method used was calculated by spiking peanut kernels at different levels ranging from 1 to 100 ng/g of aflatoxins and was estimated at 95.2 ± 8.4%. The lowest detection limit was 1 ng/g.

### Statistics

All the experiments results were evaluated using analysis of variance (ANOVA) for multiple comparisons followed by the Turkey test. Differences were considered significant at *p* < 0.05.

## Additional Information

**How to cite this article**: Xing, F. *et al*. Variation in fungal microbiome (mycobiome) and aflatoxins during simulated storage of in-shell peanuts and peanut kernels. *Sci. Rep*. **6**, 25930; doi: 10.1038/srep25930 (2016).

## Supplementary Material

Supplementary Information

## Figures and Tables

**Table 1:** Summary of pyrosequencing analysis.

Storage		In-shell peanuts		Peanut kernels	
Temperature/relative humidity	Time	Number of Reads	Average Read Length	Number of Reads	Average Read Length (bp)
	0	48352	324	48352	324
20 °C/70%	10	36868	327	10440	327
	20	44551	327	49526	331
	30	39340	332	45115	334
	60	54865	327	47447	334
	90	8694	319	–	–
20 °C/75%	10	21355	323	60505	328
	20	42635	327	41625	333
	30	23883	332	22802	333
	60	39909	325	31118	323
	90	20617	318	43916	318
25 °C/75%	10	8134	325	–	–
	20	49697	323	14472	327
	30	50917	331	20643	324
	60	22340	332	70923	328
	90	148647	333	44956	324
30 °C/75%	10	119292	328	31395	328
	20	50890	329	75468	328
	30	63462	337	13954	332
	60	63108	331	56194	346
	90	136917	348	49436	363
30 °C/80%	10	19000	307	17193	334
	20	90315	334	41494	334
	30	65118	334	31678	334
	60	42468	328	23112	311
	90	163054	340	29730	339
Average		56709	329	38396	331

**Table 2:** Distribution of operational taxonomic units (OTUs) among fungal lineages including the 10 most abundant genera detected in in-shell peanuts and peanut kernels.

In-shell peanuts			Peanut kernels		
Taxnomic affinity	Percent of OTUs	Percent of reads	Taxnomic affinity	Percent of OTUs	Percent of reads
Ascomycota	61.1	50.7	Ascomycota	60.5	44.2
Eurotiomycetes	30.1	44.1	Eurotiomycetes	31.5	39.5
*Eurotium*	4.8	21.6	*Eurotium*	6.1	19.6
*Aspergillus*	12.8	12.1	*Aspergillus*	14.4	10.1
*Penicillium*	7.7	10.1	*Penicillium*	8.4	9.7
Dothideomycetes	8.2	0.8	Dothideomycetes	7.1	1.0
*Lasiodiplodia*	1.4	0.4	*Lasiodiplodia*	1.1	0.8
*Cladosporium*	2.0	0.2	*Cladosporium*	1.7	0.2
Saccharomycetes	2.1	0.9	Saccharomycetes	2.1	1.3
*Candida*	1.8	0.9	*Candida*	1.5	1.3
Sordariomycetes	16.9	4.7	Sordariomycetes	16.5	2.2
*Fusarium*	3.3	2.4	*Fusarium*	3.9	0.8
*Bionectria*	1.3	1.2	*Gibberella*	2.8	0.2
Zygomycota	7.1	23.7	Zygomycota	7.8	27.8
Mucomycotina	7.1	23.7	Mucomycotina	7.8	27.8
*Rhizopus*	5.2	23.5	*Rhizopus*	5.9	27.8
Basidiomycota	9.4	13.0	Basidiomycota	9.7	13.5
Wallemiomycetes	5.3	11.3	Wallemiomycetes	6.8	13.4
*Wallemia*	5.3	11.3	*Wallemia*	6.8	13.4
Tremellomycetes	2.4	0.2	Tremellomycetes	1.7	0.1
*Cryptococcus*	1.1	0.2	*Cryptococcus*	0.7	0.1

**Table 3:** Mean levels of aflatoxins B_1_, B_2_, G_1_ and G_2_ in stored peanuts.

Storage		In-shell peanuts Aflatoxins (μg/kg)				Peanut kernels Aflatoxins (μg/kg)			
Temperature/relative humidity	Time	AFB_1_	AFB_2_	AFG_1_	AFG_2_	AFB_1_	AFB_2_	AFG_1_	AFG_2_
G0	0	–	–	–	–				
20 °C/70%	10	10.40	1.87	–	0.15	–	–	–	–
	20	–	–	–	–	–	–	–	–
	30	–	–	–	–	–	–	–	–
	60	0.88	0.08	–	–	–	–	–	–
	90	0.35	0.07	–	–	0.35	0.07	–	–
20 °C/75%	10	–	–	–	–	–	–	–	–
	20	–	–	–	–	–	**–**	**–**	**–**
	30	**–**	**–**	**–**	**–**	**–**	**–**	**–**	**–**
	60	0.76	0.10	0.72	0.15	**–**	**–**	**–**	**–**
	90	**–**	**–**	**–**	**–**	**–**	**–**	**–**	**–**
25 °C/75%	10	**–**	**–**	**–**	**–**	**–**	**–**	**–**	**–**
	20	**–**	**–**	**–**	**–**	**–**	**–**	**–**	**–**
	30	**–**	**–**	**–**	**–**	68.79	6.25	**–**	0.24
	60	**–**	**–**	**–**	**–**	**–**	**–**	**–**	**–**
	90	**–**	**–**	**–**	-	**–**	**–**	**–**	**–**
30 °C/75%	10	**–**	**–**	**–**	**–**	7.65	0.54	**–**	**–**
	20	**–**	**–**	**–**	**–**	**–**	**–**	**–**	**–**
	30	**–**	**–**	**–**	**–**	**–**	**–**	**–**	**–**
	60	**–**	**–**	**–**	**–**	**–**	**–**	**–**	**–**
	90	**–**	**–**	**–**	**–**	**–**	**–**	**–**	**–**
30 °C/80%	10	**–**	**–**	**–**	**–**	**–**	**–**	**–**	**–**
	20	**–**	**–**	**–**	**–**	**–**	**–**	**–**	**–**
	30	**–**	**–**	**–**	**–**	**–**	**–**	**–**	**–**
	60	**–**	**–**	**–**	**–**	**–**	**–**	**–**	-
	90	0.34	**–**	**–**	**–**	0.34	**–**	**–**	**–**
