# Supplementary material for: Variation in fungal microbiome (mycobiome) and aflatoxins during simulated storage of in-shell peanuts and peanut kernels
Source: Sci Rep. 2016 May 16;6:25930. doi: 10.1038/srep25930 (PMC4867573; doi:10.1038/srep25930)
Supplement: Supplementary Information [file srep25930-s1.pdf]

Title:

Variation in fungal microbiome (mycobiome) and aflatoxins during simulated storage of in-shell peanuts and peanut kernels

Fuguo Xing[1], Ning Ding[1], Xiao Liu, Jonathan Nimal Selvaraj, Limin Wang, Lu Zhou, Yueju Zhao, Yan Wang, Yang Liu*

[1]These authors contributed equally to this work.

*Corresponding Author

S1 Bioinformatics analyses

Paired-end reads from the original DNA fragments were merged by using FLASH[1]. Sequences were analyzed with the QIIME[2] software package (Quantitative Insights into Microbial Ecology) using default parameters for each step. Sequences with lengths < 300 bp, ambiguous bases, or primer mismatches were removed.

OTU picking was performed according to Gregory Caporaso *et al.*[3] with minor modifications. To facilitate OTU picking on so many sequences, OTUs were chosen in a two-step process. First, denoised sequences were clustered into OTUs using the UCLUST method[4, 5] at an identity threshold of 97%. Those clusters represented globally by a single sequence were discarded as likely sequencing errors according to the suggestions by Tedersoo *et al.* (2010)[6]. The second step of OTU picking was performed by BLASTing the representative sequences against the NCBI-nr/nt database (November 2015) and a customized database combing the UNITE and INSDC databases (November 2015). All filtered version of the greengenes was used as the reference database, and sequences were clustered by their representative BLAST match. Representative BLAST matches were chosen on the basis of three criteria. First, the BLAST match must achieve an E-value less than 1e-10. Next, the percent sequence identity of the alignment between a BLAST match and the read must be greater than or equal to the OTU selection threshold (0.97 here, corresponding to species-like OTUs). Of the remaining BLAST matches, the match that achieves the longest alignment to the read is chosen as the representative BLAST match. For each resulting OTU, a representative sequence was chosen as the most abundant postquality filtering read.

Taxonomy for the sequences was assigned to the representative sequence of each

OUT using QIIME's parallel wrappers for the RDP Classifier[7]. The most detailed taxonomic level assigned to an OTU's representative sequence at confidence of greater than or equal to 0.80 was taken as the taxon of the OTU. Each representative sequence was assigned the taxonomy of the best BLAST hit with an E-value less than 0.001, and the relative abundances of each taxon were then computed at each taxonomic level. Other analyses, including rarefaction curves, Shannon index, and Good's coverage, were performed with QIIME. In addition, the OTU table produced by the QIIME pipeline was imported into MEGAN 4 and mapped on the NCBI taxonomy database[8]. The genera shared among the three samples were represented by a Scale-Venn diagram using eulerAPE (http://www.eulerdiagrams.org/eulerAPE/). Even though read abundance cannot be used for direct quantification without calibration and does not allow for valid between-taxa comparisons, it does provide reliable measures for within-taxon comparisons[9]. Abundance-based comparisons were therefore made solely within selected taxonomic groups such as *Aspergillus*, *Eurotium*, *Penicillium*, *Rhizopus* and *Wallemia*, using an OTU table that was rarified in QIIME.

Principal Coordinate Analysis (PCoA) has been recognized as a simple and straight-forward method to group and separate samples in a dataset, and has been used in disease-association, gender-association and ethnicity studies[10-12]. In the current study, PCoA was used to analyze the sequencing results using the Multivariate Statistical Package, MVSP (Kovach, Wales, UK) and SAS (Cary, NC). The PCoA performs an Eigen analysis on the data matrix using a Brays Curtis distance metric. Graphically, PCoA is a rotation of a swarm of data points in two-dimensional space so the axis with the greatest variance in the first principal component axis (PC1). The second axis orthogonal to the first is the second principal component and represents the second greatest variance of the data. The first two or three principal components

generally accoChem and Biodiversunt for most of the variance of the data[10].

1. Magoc, T. & Salzberg, S. L. FLASH: fast length adjustment of short reads to improve genome assemblies. *Bioinformatics* **27**, 2957−2963 (2011).
2. Gillever, P., Siskaroodi, M., Keshavarzian, A. & Mudu, E. A. Quantitative assessment of the human gut microbiome using multitag pyrosequencing. *Chem. Biodivers.* **7**, 1065−1075 (2010).
3. Gregory Caporaso, J. *et al*. Global patterns of 16S rRNA diversity at a depth of millions of sequences per sample. *Proc. Natl. Acad. Sci. USA.* **108**, 1516−4522 (2011).
4. Caporaso, J. G. *et al.* QIIME allows analysis of high-throughput community sequencing data. Nat. Methods **7**, 335−336 (2010).
5. Edgar, R. C. Search and clustering orders of magnitude faster than BLAST. *Bioinformatics* **26,** 2460−2461 (2010).
6. Tedersoo, L. *et al*. 454 Pyrosequencing and Sanger sequencing of tropical mycorrhizal fungi provide similar results but reveal substantial methodological biases. *New Phytol.* **188,** 291−301 (2010).
7. Wang, Q., Garrity, G. M., Tiedje, J. M. & Cole, J. R. Naive Bayesian classifier for rapid assignment of rRNA sequences into the new bacterial taxonomy. *Appl. Environ. Microbiol.* **73**, 5261−5267 (2007).
8. Huson, D., Mitra, S., Ruscheweyh, H. J., Weber, N. & Schuster, S.C. Integrative analysis of environmental sequences using MEGAN4. *Genome Res.* **21**, 1552−1560 (2011).
9. Amend, A. S., Seifert, K. A. & Bruns, T. D. Quantifying microbial communities with 454 pyrosequencing: does read abundance count? *Mol. Ecol.* **19**, 5555−5565 (2010).
10. Ghannoum, M. A. *et al*. Characterization of the oral fungal microbiome (Mycobiome) in healthy individuals. *PLoS Pathog*. **6**, e1000713 (2010).
11. Price, A. L. *et al*. Principal component analysis corrects for stratification in genome-wide association studies. *Nat. Genet*. **38**, 904-909 (2006).
12. McVean, G. A genealogical interpretation of principal components analysis. *PLoS Genet.* **5**, e1000686 (2009).

Table S1 OTUs sequence and annotation in stored in-shell peanuts

>OTU_1
AAAGTGCGATAACTAGTGTGAATTGCATATTCAGTGAATCATCGAGTCTTTGAACGCAGCTT
GCACTCTATGGTTtttCTATAGAGTACGCCTGCTTCAGTATCATCACAAACCCAcacaTAAC
ATTTGTTTATGTGGTGATGGGTCGCATCGCTGTTTTATTACAGTGAGCACCTAAAATGtgtg
tgATTTTCTGTCTGGCTTGCTAGGCAGGAATATTACGCTGGTCTCAGGATCTTtttttttGG
TTCGCCCAGGAAGTAAAGTACAAGAGTATAATCCAGTAACTTTCAAACTATGATCTGAAGTC
AGGTGGGATTACCCGCTGAACTTAAGCATATCAATAAGCGGAGGAGTCCGTAGGTGAACCTG
CATATCAATAAGCGGAGGAGCATATCAATAAGCGGAGGAATCCGTAGGTGAACCTGCATATC
AATAAGCGGAGGA
k__Fungi;p__Zygomycota;c__Incertae_sedis;o__Mucorales;f__Mucoraceae;g__Rhizopus;s__Rhizopus_oryzae 1.000

>OTU_2
GAAATGCGATAATTAATGTGAATTGCAGAATTCAGTGAATCATCGAGTCTTTGAACGCACAT
TGCGCCcccTGGTATTCCGGggggCATGCCTGTCCGAGCGTCATTGCTGCCCTCAAGCACGG
CTTGtgtgtTGGGCTTCCGTCCCTGGTAACGGGGACGGGCCCAAAAGGCAGTGGCGGCACCA
TGTCTGGTCCTCGAGCGTATGGGGCTTTGTCACCCGCTCCCGTAGGTCCAGCTGGCAGCTAG
CCTCGCAACCAATCTTtttAACCAGGTTGACCTCGGATCAGGTAGGGATACCCGCTGAACTT
AAGCATATCAATAAGCGGAGGAATCCGTAGGTGAACCTGCGGAAGGACGTTGGCATATCAAT
AAGCGGAGGATGAGTCCGTAGGTGAACCTGCGGCATATCAATAAGCGGAGGATGAGTCCGTA
GGTGAACCTGC
k__Fungi;p__Ascomycota;c__Eurotiomycetes;o__Eurotiales;f__Trichocomaceae;g__Eurotium;s__Eurotium_niveoglaucum 1.000

>OTU_3
GAAATGCGATACTTGGTGTGAATTGCAGAATCCCGTGAACCATCGAGTCTTTGAACGCAAGT
TGCGCCCGAAGCCCTTAGGCTGAGGGCACGCCTGCCTGGGTGTCACCAAAAGGCGCCccccc
GTCTCGCCCGTCCCAGGGCACGGGGAGGgggCGAACGTTGGCCTCCCGGGAGCCCCTGGCTC
GCGGTTGGTTCAAAGAGACGGGCTCTTGGTGGGGAGCGGCACCGCGGCAGATGGTGGTCGAG
AACAACCCTCGTGGCCAGTCGcgcgcgcCTCTCCcccGGTTCAAGGCACGGCGACCCGCGGG
CGACGTGGATCGTCCCGAGCGCGACCTCAGGTCAGGCGGGGCTACCCGCTGAGTTTAAGCAT
ATCAATGAAGAACGCAGCTCCGTAGGTGAACCTGCGGA
Arachis hypogaea

>OTU_4
GAAATGCGATAAGTAATGTGAATTGCAGAATTCCGTGAATCATCGAATCTTTGAACGCACAT
TGCGCCcccTGGCATTCCGGggggCATGCCTGTCCGAGCGTCATTTCTGCCCTCAAGCACGG
CTTGtgtgtTGGGTGTGGTCCccccGGGGACCTGCCCGAAAGGCAGCGGCGACGTCCGTCTG
GTCCTCGAGCGTATGGGGCTCTGTCACTCGCTCGGGAAGGACCTGCGGgggTTGGTCACCAC
CATGTTtttACCACGGTTGACCTCGGATCAGGTAGGAGTTACCCGCTGAACTTAAGCATATC
AATAAGCGGAGGAATCCGTAGGTGAACCTGCGGCATATCAATAAGCGGAGGATGAGTCCGTA
GGTGAACCTGCGGAGCATATCAATAAGCGGAGGAATCCGTAGGTGAACCTGCGGAAGGATAG
AC

k__Fungi;p__Ascomycota;c__Eurotiomycetes;o__Eurotiales;f__Tric
hocomaceae;g__Penicillium ;S__*Penicillium_pinophilum* 1.000

>OTU_5
GAAATGCGATAAGTAATGTGAATTGCAGATACAGTGAATCATCGAATCTTTGAACGCATATT
GCACCTTTTGGTATTCCATAAGGTACGTCTGTTTGAGCGTCGCGAACATCTCATAATTAATG
AATTtttttttGTTAATTATGGTCTTTGAGTTTGTCTATAATTtttAGACTCACTTTAAATT
GATTAGTAGTTTAACTTTTGAAAGGGTTAAAATTAGGTGTTttttAATGTACATTACTTTGT
GCATCATCTAATCAAGAGTTACTTACTCTGCCTTAGTATTAATGTTACTGCTTCTAATAGCT
TATTAAGCAAGTAATATTTCAATCGACCTCAAATCAGATGGGATTACCCGCTGAACTTAAGC
ATATCAATAAGCGGAGGAGCATATCAATAAGCGGAGGAGCATATCAATAAGCGGAGGA
Uncultured fungus

>OTU_6
GAAATGCGATAACTAGTGTGAATTGCAGAATTCCGTGAATCATCGAGTCTTTGAACGCACAT
TGCGCCcccTGGTATTCCGGggggCATGCCTGTCCGAGCGTCATTGCTGCCCATCAAGCACG
GCTTGtgtgtTGGGTCGTCGTCCCCTCTCCGGgggggACGGGCCCCAAAGGCAGCGGCGGCA
CCGCGTCCGATCCTCGAGCGTATGGGGCTTTGTCACCCGCTCTGTAGGCCCGGCCGGCGCTT
GCCGAACGCAAATCAATCTTtttCCAGGTTGACCTCGGATCAGGTAGGGATACCCGCTGAAC
TTAAGCATATCAATAAGCGGAGGAATCCGTAGGTGAACCTGCGGCATATCAATAAGCGGAGG
A
k__Fungi;p__Ascomycota;c__Eurotiomycetes;o__Eurotiales;f__Tric
hocomaceae;g__Aspergillus ;s__Aspergillus_flavus 0.990

>OTU_7
GAAATGCGATAACTAATGTGAATTGCAGAATTCAGTGAATCATCGAGTCTTTGAACGCACAT
TGCGCCcccTGGTATTCCGGggggCATGCCTGTCCGAGCGTCATTGCTGCCCTCAAGCCCGG
CTTGtgtgtTGGGTCGCCGTCCcccTCTCCGGggggACGGGCCCGAAAGGCAGCGGCGGCAC
CGCGTCCGATCCTCGAGCGTATGGGGCTTTGTCACATGCTCTGTAGGATTGGCCGGCGCCTG
CCGACGTTTTCCAACCATTCTTTCCAGGTTGACCTCGGATCAGGTAGGGATACCCGCTGAAC
TTAAGCATATCAATAAGCGGAGGAGCATATCAATAAGCGGAGGAATCCGTAGGTGAACCTGC
GGACGCATATCAATAAGCGGAGGAATCCGTAGGTGAACCTGCG
k__Fungi;p__Ascomycota;c__Eurotiomycetes;o__Eurotiales;f__Tric
hocomaceae;g__Aspergillus s__Aspergillus_niger 1.000

>OTU_8
GAAATGCGATAAGTAATGTGAATTGCAGATACAGTGAATCATCGAATCTTTGAACGCAAATG
GCACTCTATGGTATTCCGTAGAGTACGTCTGTTTGAGCGTCGCGAACATCTCCATAATTGGT
TtttttAAATTGATTGTGGGTTTTGAGGTTGTCATATAAACAATGACTCCCTTTAAAATAAT
TAGTGATGACCTTATGAATGGGTTAATACTGtgtgtTATAATGGATTACATCCATCACCAGT
CAGagagTAATCTCGCCTTAGTAATTTGTAGTGATTGCTTCTAACTGCCATTGGCAAACAAA
CTGATCAAATCGACCTCAAATCAGATGGGATTACCCGCTGAACTTAAGCATATCAATAAGCG
GAGGAATCCGTAGGTGAACCTGCGGCCAGGTTGAAGAACGCAGCGTCGTCGCATCGATGAA
GAAC

k__Fungi;p__Basidiomycota;c__Wallemiomycetes;o__Wallemiales;f_
_Wallemiaceae;g__Wallemia;s__Wallemia_sebi   0.990

>OTU_9
GAAATGCGATAACTAGTGTGAATTGCAGAATTCCGTGAATCATCGAGTCTTTGAACGCACAT
TGCGCCcccTGGTATTCCGGggggCATGCCTGTCCGAGCGTCATTGCTGCCCATCAAGCACG
GCTTGtgtgtTGGGTCGTCGTCCCCTCTCCGGgggggACGGGCCCCAAAGGCAGCGGCGGCA
CCGCGTCCGATCCTCGAGCGTATGGGGCTTTGTCACCCGCTCTGTAGGCCCGGCCGGCGCTT
GCCGAACGCAAATCAATCTTTTCCAGGTTGACCTCGGATCAGGTAGGGATACCCGCTGAACT
TAAGCATATCAATAAGCGGAGGAGCATCGATGAAGAACGCAGGCATCGTGAAGAACGCAGCA
TCGATGAAGAAC
k__Fungi;p__Ascomycota;c__Eurotiomycetes;o__Eurotiales;f__Tric
hocomaceae;g__Aspergillus;s__Aspergillus_flavus 1.000

>OTU_10
GAAATGCGATACGTAATGTGAATTGCAGAATTCAGTGAATCATCGAGTCTTTGAACGCACAT
TGCGCCcccTGGTATTCCGGggggCATGCCTGTCCGAGCGTCATTGCTGCCCTCAAGCACGG
CTTGtgtgtTGGGCCcccGTCCcccTCCCAGGAAGGGGACGGGCCCGAAAGGCAGCGGCGGC
ACCGTGTCCGGTCCTCGAGCGTATGGGAAGCAACTTtttGTCACCCGCTCCTGTAGGTCCGG
CCGGCGGCCTGCCCAACCCCAACCTTtttttAACCAGGTTGACCTCGGATCAGGTAGGGATA
CCCGCTGAACTTAAGCATATCAATAAGCGGAGGAAaaaTGTGCATCGATGAAGAACGCA
k__Fungi;p__Ascomycota;c__Eurotiomycetes;o__Eurotiales;f__Tric
hocomaceae;g__Aspergillus;s__Aspergillus_penicillioides 0.990

>OTU_11
GAAATGCGATACGTAATGTGAATTGCAGAATTCAGTGAATCATCGAGTCTTTGAACGCACAT
TGCGCCcccTGGTATTCCGGggggCATGCCTGTCCGAGCGTCATTGCTGCCCTCAAGCACGG
CTTGtgtgtTGGGCCcccGTCCccccccAAGGGGACGGGCCCGAAAGGCAGCGGCGGCACCG
TGTCCGGTCCTCGAGCGTATGGGAAGCAACTTtttttGTCACCCGCTCCTGTAGGTCCGGCC
GGCGGCCTGCCCAACCCCAACCTTTTACTTTAACCAGGTTGACCTCGGATCAGGTAGGGATA
CCCGCTGAACTTAAGCATATCAATAAGCGGAGGAGGAGTCCGTAGGTGAACCTGCGGGCATC
GATGAAGAAC
k__Fungi;p__Ascomycota;c__Eurotiomycetes;o__Eurotiales;f__Tric
hocomaceae;g__Aspergillus;s__Aspergillus_penicillioides 0.990

>OTU_12
GAAATGCGATACGTAATGTGAATTGCAGAATTCAGTGAATCATCGAGTCTTTGAACGCACAT
TGCGCCcccTGGTATTCCGGggggCATGCCTGTCCGAGCGTCATTGCTGCCCTCAAGCACGG
CTTGtgtgtTGGGCCcccGTCCcccTGTTCTAGGgggggACGGGCCCGAAAGGCAGCGGCGG
CACCGTGTCCGGTCCTCGAGCGTATGGGAAGCAACTTtttGTCACCCGCTCCCGTAGGTCCG
GCCGGCGGCCTGCCCAACCcccATCAATCTTtttttAACCAGGTTGACCTCGGATCAGGTAG
GGATACCCGCTGAACTTAAGCATATCAATAAGCGGAGGAGCATATCAATAAGCGGAGGAGCA
TCGATGAAGAC
k__Fungi;p__Ascomycota;c__Eurotiomycetes;o__Eurotiales;f__Tric

hocomaceae;g__Aspergillus;s__Aspergillus_penicillioides 0.960

>OTU_13
TTGCACTCTATGGTTtttCTATAGAGTACGCCTGCTTCAGTATCATCACAAACCCAcacaTA
ACATTTGTTTATGTGGTGATGGGTCGCATCGCTGTTTTATTACAGTGAGCACCTAAAATGtg
tgtgATTTTCTGTCTGGCTTGCTAGGCAGGAATATTACGCTGGTCTCAGGATCTTttttttt
GGTTCGCCCAGGAAGTAAAGTACAAGAGTATAATCCAGTAACTTTCAAACTATGATCTGAAG
TCAGGTGGGATTACCCGCTGAACTTAAGCATATCAATAAGCGGAGGAATCTGTAGGTGAACC
TGCATATCAATAAGCGGAGGAGCATATCAATAAGCGGAGGAGCATATCAATAAGCGGAGGAG
CATATCAATAAGCGGAGGAGCATATCAATAAGCGGAGGAGCATATCAATAAGCGGAGGA
k__Fungi;p__Zygomycota;c__Incertae_sedis;o__Mucorales;f__Mucor
aceae;g__Rhizopus;s__Rhizopus_oryzae 1.000

>OTU_14
GAAATGCGATACGTAATGTGAATTGCAGAATTCAGTGAATCATCGAGTCTTTGAACGCACAT
TGCGCCcccTGGTATTCCGGggggCATGCCTGTCCGAGCGTCATTGCTGCCCTCAAGCACGG
CTTGtgtgtTGGGCCcccGTCCcccTCTTttttAGGgggggggACGGGCCCGAAAGGCAGC
GGCGGCACCGTGTCCGGTCCTCGAGCGTATGGGAAGCAACTCTTtttGTCACCCGCTCCTGT
AGGTCCGGCCGGCGGCCTGCCCAACCCTCAATCAATCTTttttAACCAGGTTGACCTCGGAT
CAGGTAGGGATACCCGCTGAACTTAAGCATATCAATAAGCGGAGGAATCCGTAGGTGAACCT
GCG
k__Fungi;p__Ascomycota;c__Eurotiomycetes;o__Eurotiales;f__Tric
hocomaceae;g__Aspergillus;s__Aspergillus_penicillioides 1.000

>OTU_15
GAAATGCGATAAGTAATATGAATTGCAGATATTCGTGAATCATCGAATCTTTGAACGCACAT
TGCGCCCTTTGGTATTCCAAAGGGCATGCCTGTTTGAGCGTCATTTCTCCCTCAAACCCTCG
GGTTTGGTGTTGAGCGATACGCTGGGTTTGCTTGAAAGAAAGGCGGAGTATAAACTAATGGA
TAGGTTttttCCACTCATTGGTACAAACTCCAAAACTTCTTCCAAATTCGACCTCAAATCAG
GTAGGACTACCCGCTGAACTTAAGCATATCAATAAGCGGAGGATCCGTAGGTGAACCTGCGG
AGCATATCAATAAGCGGAGGAGTCCGTAGGTGAACCTGCGGCATATCAATAAGCGGAGGATG
AGTCCGTAGGTGAACCTGCGGA
k__Fungi;p__Ascomycota;c__Saccharomycetes;o__Saccharomycetales
;f__Incertae_sedis;g__Candida;s__Candida_parasilosis 1.000

>OTU_16
GAAATGCGATAAGTAATGTGAATTGCAGAATTCAGTGAATCATCGAATCTTTGAACGCACAT
TGCGCCCGCCAGTATTCTGGCGGGCATGCCTGTCTGAGCGTCATTTCAACCCTCATGCCCCT
AGGGCGTGGTGTTGGGGATCGGCCAAAGCCCGCGAGGGACGGCCGGCCCCTAAATCTAGTGG
CGGACCCGTCGTGGCCTCCTCTGCGAAGTAGTGATATTCCGCATCGGAGAGCGATGAGCCCC
TGCCGTTAAACCcccAACTTTCTAAGGTTGACCTCAGATCAGGTAGGAATACCCGCTGAACT
TAAGCATATCAATAAGCGGAGGAATCCGTAGGTGAACCTGCGGAAGGATAGACGCATATCAA
TAAGCGGAGGAATCCGTAGGTGAACCTGCGGA
k__Fungi;p__Ascomycota;c__Sordariomycetes;o__Hypocreales;f__Bi

onectriaceae;g__Bionectria;s__Clonostachys_rosea 1.000

>OTU_17
GAAATGCGATAAGTAATGTGAATTGCAGAATTCAGTGAATCATCGAATCTTTGAACGCACAT
TGCGCCCCTTGGTATTCCGGggggCATGCCTGTTCGAGCGTCATTTCAACCCTCAAGCTCTG
CTTGGTATTGGGCACCGTCCTTTGCGGGCgcgcCTCAAAGACCTCGGCGGTGGCGTCTTGCC
TCAAGCGTAGTAGAATACACCTCGCTTCGGAGCGTAAGGCGTCGCCCGCCGGACGAACCTTC
TGAACTTTTCTCAAGGTTGACCTCGGATCAGGTAGGGATACCCGCTGAACTTAAGCATATCA
ATAAGCGGAGGAATCCGTAGGTGAACCTGCGGCATATCAATAAGCGGAGGATGAGTCCGTAG
GTGAACCTGCATATCAATAAGCGGAGGAATCCGTAGGTGAACCT
k__Fungi;p__Ascomycota;c__Dothideomycetes;o__Botryosphaeriales
;f__Botryosphaeriaceae;g__Macrophomina;s__Macrophomina_phaseolin
a 1.000

>OTU_18
AAAATGCGATAAGTAATGTGAATTGCAGAATTCAGTGAATCATCGAATCTTTGAACGCACAT
TGCGCCCGCCAGTATTCTGGCGGGCATGCCTGTTCGAGCGTCATTTCAACCCTCAAGCACAG
CTTGGTGTTGGGACTCGCGTTAATTCGCGTTCCCCAAATTGATTGGCGGTCACGTCGAGCTT
CCATAGCGTAGTAGTAAAACCCTCGTTACTGGTAATCGTCGCGGCCACGCCGTTAAACCCCA
ACTTCTGAATGTTGACCTCGGATCAGGTAGGAATACCCGCTGAACTTAAGCATATCAATAAG
CGGAGGAGTCCGTAGGTGAACCTGCGGAGCATATCAATAAGCGGAGGAATCCGTAGGTGAAC
CTGCG
k__Fungi;p__Ascomycota;c__Sordariomycetes;o__Hypocreales;f__Ne
ctriaceae;g__Fusarium s__Fusarium_oxysporum 1.000

>OTU_19
GAAATGCGATAAGTAATGTGAATTGCAGAATTCAGTGAATCATCGAATCTTTGAACGCACAT
TGCGCCCGCCAGTATTCTGGCGGGCATGCCTGTTCGAGCGTCATTACAACCCTCAGGCCccc
GGGCCTGGCGTTGGGGATCGGCGGAAGCCcccTGCGGGCACAACGCCGTCCcccAAATACAG
TGGCGGTCCCGCCGCAGCTTCCATTGCGTAGTAGCTAACACCTCGCAACTGGAGAGCGGCGC
GGCCACGCCGTAAAACACCCAACTTCTGAATGTTGACCTCGAATCAGGTAGGAATACCCGCT
GAACTTAAGCATATCAATAAGCGGAGGAGCATATCAATAAGCGGAGGAATCCGTAGGTGAAC
CTGCGGCATATCAATAAGCGGAGGAGCATATCAATAAGCGGAGGAATCCGTAGGTGAACCTG
CG
k__Fungi;p__Ascomycota;c__Sordariomycetes;o__Hypocreales;f__Ne
ctriaceae;g__Fusarium;s__Fusarium_solani 0.990

>OTU_20
GAAATGCGATAAGTAATGTGAATTGCAGAATTCAGTGAATCATCGAATCTTTGAACGCACAT
TGCGCCCCTTGGTATTCCGGggggCATGCCTGTTCGAGCGTCATTACAACCCTCAAGCTCTG
CTTGGAATTGGGCACCGTCCTCACTGCGGACGCGCCTCAAAGACCTCGGCGGTGGCTGTTCA
GCCCTCAAGCGTAGTAGAATACACCTCGCTTTGGAGCGGTTGGCGTCGCCCGCCGGACGAAC
CTTCTGAACTTTTCTCAAGGTTGACCTCGGATCAGGTAGGGATACCCGCTGAACTTAAGCAT
ATCAATAAGCGGAGGAGCATATCAATAAGCGGAGGAATCCGTAGGTGAACCTGCGGA

k__Fungi;p__Ascomycota;c__Dothideomycetes;o__Botryosphaeriales;f__Botryosphaeriaceae;g__Lasiodiplodia;s__Lasiodiplodia_theobromae 1.000

>OTU_21
CAAATGCGATAAGTAATGTGAATTGCAGAGTTCCGTGAATCATCGAATTtttGAACGCATAT
TGCGCCTTGTGGTAATCCGCAAGGCATGCCTGTCTGAGCGTCATTACATCATTCAAGTAAAA
GTTttttttttACTTGGTCTTGGAATTTGACTTtttACCAACTTGTTGGTAGTTATGTCT
GTTCTGTAATGTATGATTGCAGTCTATTGACACTTTTAGTTGTAGTACATACCATCAACTTT
AAAAGCCTCTAGACCTGGTCACCTTTACCATCAATCGCTTGATTGAAATTTTCTTGTAAAGT
TTGACCTCAGATCAGGTAGGACTACCCGCTGAACTTAAGCATATCAATAAGCGGAGGAATCC
GTAGGTGAACCTGCGGA
Uncultured fungus 0.910

>OTU_22
GAAATGCGATAAGTAATGTGAATTGCAGAATTCAGTGAATCATCGAATCTTTGAACGCACAT
TGCGCCcccTGGTATTCCGGggggCATGCCTGTTCGAGCGTCATTTCACCACTCAAGCCTCG
CTTGGTATTGGGCAACGCGGTCCGCCGCGTGCCTCAAATCGACCGGCTGGGTCTTCTGTCCC
CTAAGCGTTGTGGAAACTATTCGCTAAAGGGTGCTCGGGAGGCTACGCCGTAAAACAAACCC
ATTTCTAAGGTTGACCTCGGATCAGGTAGGGATACCCGCTGAACTTAAGCATATCAATAAGC
GGAGGAGCATATCAATAAGCGGAGGAGCATATCAATAAGCGGAGGAGCATCGATGAAGAACG
CA
k__Fungi;p__Ascomycota;c__Dothideomycetes;o__Capnodiales;f__Mycosphaerellaceae;g__Cladosporium 1.000

>OTU_23
GAAATGCGATAAGTAATGTGAATTGCAGAATTCAGTGAATCATCGAGTCTTTGAACGCACAT
TGCGCCcccTGGTATTCCGGggggCATGCCTGTCCGAGCGTCATTGCTGCCCTCAAGCACGG
CTTGtgtgtTGGGCTTCGCCccccGGCTCCCGGggggCGGGCCCGAAAGGCAGCGGCGGCAC
CGCGTCCGGTCCTCGAGCGTATGGGGCTTCGTCACCCGCTCTGTAGGCCCGGCCGGCGCCCG
CCGGCGACCCCAATCAATCTTTCCAGGTTGACCTCGGATCAGGTAGGGATACCCGCTGAACT
TAAGCATATCAATAAGCGGAGGA
k__Fungi;p__Ascomycota;c__Eurotiomycetes;o__Eurotiales;f__Trichocomaceae;g__Penicillium;s__Penicillium_simplicissimum 0.990

>OTU_24
GAAATGCGATAAGTAGTGTGAATTGCAGAATTCAGTGAATCATCGAATCTTTGAACGCACAT
TGCGCCCTTTGGTATTCCAAAGGGCATGCCTGTTCGAGCGTCATTTGTACCCTCAAGCTTTG
CTTGGTGTTGGGCGTCTTGTCTCTAGCTTTGCTGGAGACTCGCCTTAAAGTAATTGGCAGCC
GGCCTACTGGTTTCGGAGCGCAGCACAAGTCGCACTctctATCAGCAAAGGTCTAGCATCCA
TTAAGCCTTtttttCAACTTTTGACCTCGGATCAGGTAGGGATACCCGCTGAACTTAAGCAT
ATCAATAAGCGGAGGAGCATATCAATAAGCGGAGGAATCCGTAGGTGAACCTGCATATCAAT
AAGCGGAGGA

k__Fungi;p__Ascomycota;c__Dothideomycetes;o__Pleosporales;f__Pleosporaceae;g__Alternaria;s__Alternaria_alternata 1.000

>OTU_25
GAAATGCGATACGTAATGTGAATTGCAGAATTCAGTGAATCATCGAGTCTTTGAACGCACAT
TGCGCCcccTGGTATTCCGGggggCATGCCTGTCCGAGCGTCATTGCTGCCCTCAAGCACGG
CTTGtgtgtTGGGCCcccGTCCCCTCCCTAGGgggggACGGGCCCGAAAGGCAGCGGCGGCA
CCGTGTCCGGTCCTCGAGCGTATGGGAAGCACAATCTTtttttGCCAACCCGCTCCTGTAGG
TCCGGCCGGCGGCCTGCCCCAATCAACCCATTGTTtttAACCAGGTTGACCTCGGATCAGGT
AGGGATACCCGCTGAACTTAA
k__Fungi;p__Ascomycota;c__Eurotiomycetes;o__Eurotiales;f__Trichocomaceae;g__Aspergillus;s__Aspergillus_penicillioides 1.000

>OTU_26
GAAATGCGATAACTAATGTGAATTGCAGAATTCAGTGAATCATCGAGTCTTTGAACGCACAT
TGCGCCCTCTGGTATTCCGGAGGGCATGCCTGTCCGAGCGTCATTGCTGCCCTCAAGCCCGG
CTTGtgtgtTGGGCCCCGTCCccccccGCCGGggggACGGGCCCGAAAGGCAGCGGCGGCACC
GCGTCCGGTCCTCGAGCGTATGGGGCTTCGTCACCCGCTCTAGTAGGCCCGGCCGGCGCCAG
CCGACCcccAACCTTTAATTATCTCAGGTTGACCTCGGATCAGGTAGGGATACCCGCTGAAC
TTAA
k__Fungi;p__Ascomycota;c__Eurotiomycetes;o__Eurotiales;f__Trichocomaceae;g__Penicillium;s__Penicillium_citrinum 1.000

>OTU_27
TAACTGCGATAAGTAGCGTGAATTGCAGACGCTTTGAACGTTAAACTTTCGAACGCACATTG
CGCCGTAGGAGTTCTACCCTGCGGCACATCTGGTTGAGGGTCGTGATCAAaaaCTGCCCGAA
TGCAGCAGTATAGAGATAGCTGGCGAACCATGATGAAAGTCGTGATTTGCCATACGACGCTA
TAGAGGACAATTCTCCACGTGAgagagTTGCCTGCCACACGGATGATCTACTAGTTGACGCA
GTAGATCCCGGTCCCAGGTATTATGCACGAAACGTATTATGCCGAGGGATGCGCGGGGATAC
AGCTAGTGGGAGAATCCGTGCGAGCTATATGAGCTATTCGATTTCCCGACCTCAACTCAGGT
GTGATTACCCGCTGAACTTAAGCATATCAATAAGCGGAGGA
Pratylenchus goodeyi

>OTU_28
GAAATGCGATACGTAGTGTGAATTGCAGAATTCAGTGAATCATCGAATCTTTGAACGCACAT
TGCGCCCTTTGGTATTCCAAAGGGCATGCCTGTTCGAGCGTCATTTGTACCCTCAAGCTTTG
CTTGGTGTTGGGCGTTTTGTCTTTGCATCAAAGACTCGCCTTAAAACGATTGGCAGCCGGCC
TACTGGTTTCGGAGCGCAGCACATTtttGCGCTTGCAATCAGCAAGAGGTCGGCAATCCATC
AAGTCCATTTCTCACTTTTGACCTCGGATCAGGTAGGGATACCCGCTGAACTTAAGCATATC
AATAAGCGGAGGAATCCGTAGGTGAACCTGCGGCATCGATGAAGAACGCATCC
k__Fungi;p__Ascomycota;c__Dothideomycetes;o__Pleosporales;f__Pleosporaceae;g__Curvularia;s__Curvularia_sp_LH3 0.990

>OTU_29

GAAATGCGATAAGTAATGTGAATTGCAGAATTCCGTGAATCATCGAATCTTTGAACGCACAT
TGCGCCcccTGGCATTCCGGggggCATGCCTGTCCGAGCGTCATTTCTGCCCTCAAGCACGG
CTTGtgtgtTGGGTGTGGTCCccccGGGGACCTGCCCGAAAGGCAGCGGCGACGTCCGTCTG
GTCCTCGAGCGTATGGGGCTCTGTCACTCGCTCGGGAAGGACCTGCGGgggTTGGTCACCAC
CACATTTTACCACGGTTGACCTCGGATCAGGTAGGAGTTACCCGCTGAACTTAAGCATATCA
ATAAGCGGAGGATCCGTAGGTGAACCTGCGGCATATCAATAAGCGGAGGAGTCCGTAGGTGA
ACCTGCG
k__Fungi;p__Ascomycota;c__Eurotiomycetes;o__Eurotiales;f__Tric
hocomaceae;g__Penicillium ;s__Penicillium_pinophilum1.000

>OTU_30
AAACTGCGATAAGTAGCGTGAATTGCAGACGCTTTGAACGTTAAACTTTCGAACGCACATTG
CGCCGTAGGAGTCCTACCCTGCGGCACATCTGGTTGAGGGTCGTGATCAAaaaCAGCAGGAC
TATGGCTGTAATAGAAATAGCTGGTGAATCACGAGTTCGGCCTACCGCCTCTGGCCGAACGT
GTTTTGCCATAATGCTAGAAGAGGACAATCTCACGAGAGTGTGATTGCCTACCACACGGATG
ATCTATTAGCTGAAGCAATAGGTCTCGGTCACCGGGTATCATTCTTTTAtataCCATGTGGC
GTCTGAGATACAGCTAGTGGGCTTCATACGTGCCTGGTATTGAGAGTATTCGCTCTCCCGAC
CTCAACTCAGGTGTGATTACCCGCTGAACTTAA
Pratylenchus goodeyi

>OTU_31
GAAATGCGATAAGTAATGTGAATTGCAGATACAGTGAATCATCGAGTCTTTGAACGCACATT
GCGCCcccTGGTATTCCGGggggCATGCCTGTCCGAGCGTCATTGCTGCCCTCAAGCCCGGC
TTGtgtgtTGGGTCGCCGTCCcccTCTCCGGggggACGGGCCCGAAAGGCAGCGGCGGCACC
GCGTCCGATCCTCGAGCGTATGGGGCTTTGTCACATGCTCTGTAGGATTGGCCGGCGCCTGC
CGACGTTTTCCAACCATTCTTTCCAGGTTGACCTCGGATCAGGTAGGGATACCCGCTGAACT
TAAGCATATCAATAAGCGGAGGAGTCCGTAGGTGAACCTGCGGAAGGATAGAC
k__Fungi;p__Ascomycota;c__Eurotiomycetes;o__Eurotiales;f__Tric
hocomaceae;g__Aspergillus s__Aspergillus_niger 0.990

>OTU_32
GAAATGCGATAAGTAATGTGAATTGCAGAATTCAGTGAATCATCGAATCTTTGAACGCACCT
TGCGCTCCTTGGTATTCCGAGGAGCATGCCTGTTTGAGTGTCATGAAACCCTCAAACCCAAG
TTTTGGATTTCGATCCATGCTTGAGTTTGGATTTGGATGTTTGCCGGTGATGAACCGACTCA
TCTTAAAAGTATTAGCTTGGATCTGTCTATATGACTGGTTTGACTTGGCATAATAAGTATTT
TGCTGAGGACATCTTCGGATGGCCAGGACCTAGACTACTGTCTGCTAACTAAACCATCACTT
TAAGTGCATCTTTGGATGTTACTCATTGTGTAACTTTGACATCTGGCCTCAAATCAAGTAGG
ACTACCCGCTGAACTTAAGCATATCAATAAGCGGAGGAATCCGTAGGTGAACCTGCGGA
k__Fungi;p__Basidiomycota;c__Tremellomycetes;o__Filobasidiales
;f__Filobasidiaceae;g__Cryptococcus;s__Cryptococcus_magnus
1.000

>OTU_33
GAAATGCGATAAGTAATGTGAATTGCAGATACAGTGAATCATCGAGTCTTTGAACGCACATT

GCGCCcccTGGTATTCCGGggggCATGCCTGTCCGAGCGTCATTGCTGCCCTCAAGCACGGC
TTGtgtgtTGGGCTTCCGTCCCTGGCAACGGGGACGGGCCCAAAAGGCAGTGGCGGCACCAT
GTCTGGTCCTCGAGCGTATGGGGCTTTGTCACCCGCTCCCGTAGGTCCAGCTGGCAGCTAGC
CTCGCAACCAATCTTtttAACCAGGTTGACCTCGGATCAGGTAGGGATACCCGCTGAACTTA
AGCATATCAAGAAGCGGAGTAATCCGTAGGTGAACCTGCGGAGCATATCAATAAGCGGAGGA
k__Fungi;p__Ascomycota;c__Eurotiomycetes;o__Eurotiales;f__Tric
hocomaceae;g__Eurotium 0.990

>OTU_34
GAAATGCGATAAGTAATGTGAATTGCAGATACAGTGAATCATCGAATCTTTGAACGCATATT
GCACCTTTTGGTATTCCATAAGGTACGTCTGTTTGAGCGTCGCGAACATCTCATAATTAATG
AATTtttttttGTTAATTATGGTCTTTGAGTTTGTCTCTTACTAGTAGACTCACTTTAAATT
GATTAGTAGTTTAACTTTTGAAAGGGTTAAAATTAGGTGTTttttAATGTACATTACTTTGT
GCATCATCTAATCAAGAGTTAAACTTAAACTCTGCCTTAGTATTAATGTTACTGCTTCTAAT
AGCTTATTAAGCAAGTAATATTTCAATCGACCTCAAATCAGATGGGATTACCCGCTGAACTT
AA
Uncultured fungus

>OTU_35
GAAATGCGATACCTAGTGTGAATTGCAGCCATCGTGAATCATCGAGTTCTTGAACGCACATT
GCGCCCCTCGGCATTCCGGggggCATGCCTGTTTGAGCGTCGTTTCCATCTTGCGCGTGCGC
AGAGTTGGgggAGCGGAGCGGACGACGTGTAAAGAGCGTCGGAGCTGCGACTCGCCTGAAAG
GGAGCGAAGCTGGCCGAGCGAACTAGACTTtttttCAGGGACGCTTGGCGGCCGAGAGCGAG
TGTTGCGAGACAACAAaaaGCTCGACCTCAAATCAGGTAGGAATACCCGCTGAACTTAAGCA
TATCAATAAGCGGAGGAATCCGTAGGTGAACCTGA
k__Fungi;p__Ascomycota;c__Saccharomycetes;o__Saccharomycetales
;f__Incertae_sedis;g__Candida;s__Pichia_kudriavzevii 1.000

>OTU_36
GAAATGCGATAAGTAATGTGAATTGCAGATACAGTGAATCATCGAATCTTTGAACGCACATT
GCGCCCGCCAGTATTCTGGCGGGCATGCCTGTCTGAGCGTCATTTCAACCCTCATGCCCCTA
GGGCGTGGTGTTGGGGATCGGCCAAAGCCCGCGAGGGAGGGCCGGCCCCTAAATCTAGTGGC
GGACCCGTCGTGGCCTCCTCTGCGAAGTAGTGATATTCCGCATCGGAGAGCGATGAGCCCCT
GCCGTTAAACCcccAACTTTCTAAGGTTGACCTCAGATCAGGTAGGAATACCCGCTGAACTT
AAGCATATCAATAAGCGGAGGAGTCCGTAGGTGAACCTGCGGCATATCAATAAGCGGgggAA
TCCGTAGGTGAACCTGCGGAGGA
k__Fungi;p__Ascomycota;c__Sordariomycetes;o__Hypocreales;f__Bi
onectriaceae;g__Bionectria;s__Clonostachys_rosea 0.990

>OTU_37
AAAGTGCGATAACTAGTGTGAATTGCATATTCAGTGAATCATCGAGTCTTTGAACGCAGCTT
GCACTCTATGGTTtttCTATAGAGTACGCATGCTTCAGTATCATCACAAACCCAcacaTAAC
ATTTGTTTATGTGGTGATGGGTCGCATCGCTGTTTTATTACAGTGAGCACCTAAAATGtgtg
tgATTTTCTGTCTGGCTTGCTAGGCAGGAATATTACGCTGGTCTCAGGATCTTtttttttGG

TTCGCCCAGGAAGTAAAGTACAAGAGTATAATCCAGTAACTTTCAAACTATGATCTGAAGTC
AGGTGGGATTACCCGCTGAACTTAAGCATATCAATAAGCGGAGGAGTATATCAATAAGCGGA
GGAGCATATCAATAAGCGGAGGA
k__Fungi;p__Zygomycota;c__Incertae_sedis;o__Mucorales;f__Mucoraceae;g__Rhizopus;s__Rhizopus_oryzae 0.990

>OTU_38
GAAATGCGATAAGTAGTGTGAATTGCAGAATTCAGTGAATCATCGAATCTTTGAACGCACAT
TGCGCCCCTTGGTATTCCATGGGGCATGCCTGTTCGAGCGTCATTTGTACCTTCAAGCTTTG
CTTGGTGTTGGGTGTTTGTCTCGCCTTTGCGTGTAGACTCGCCTTAAAACAATTGGCAGCCG
GCGTATTGATTTCGGAGCGCAGTACATCTCGCGCTTTGCACTCATAACGACGACGTCCAAAA
GTACATTtttACACTCTTGACCTCGGATCAGGTAGGGATACCCGCTGAACTTAAGCATATCA
ATAAGCGGAGGAATCCGTAGGTGAACCTGCGGAGCATATCAATAAGCGGAGGAATCCGTAGG
TGAACCTGCGGCATATCAATAAGCGGAGGA
k__Fungi;p__Ascomycota;c__Dothideomycetes;o__Pleosporales 0.930

>OTU_39
GAAATGCGATAAGTAATGTGAATTGCAGAATCTCGTGAATCATTGAATCTTTGAACGCACAT
TGCGCCCTATGGTATTCCGTAGGGCATGCCTGTCTGAGCGTCAGCTCGTCTTCTCAAGCTCT
TTTGCTTGGATAACATTGGAACAGCAATACAAGCTCCATCTGGGGTCTGtgtgCTCTCCAGA
AATCAATAGGCGGTACGGTTCTAGCAAACCAGACGTAATAATATTCTTATCTATCGTTAATT
TGTTTAAACTGTTGCTTGCCTCTTAATCCACATCTTtttCAAGATGACCTCAGATCAGGTAG
GGATACCCGCTGAACTTAAGCATATCAATAAGCGGAGGAATCCGTAGGTGAACCTGCGCATA
TCAATAAGCGGAGGA
k__Fungi;p__Ascomycota;c__Pezizomycetes;o__Pezizales;f__unidentified;g__unidentified;s__Pezizales_sp_P10 1.000

>OTU_40
GAAATGCGATACGTAATGTGAATTGCAGAATTCAGTGAATCATCGAGTCTTTGAACGCACAT
TGCGCCCTCTGGTATTCCGGggggCATGCCTGTCCGAGCGTCATTGCTGCCCTCAAGCACGG
CTTGtgtgtTGGGCCcccGTCCCGGTTAAGCATTAGCCCCGGGACGGGCCCGAAAGGCAGCG
GCGGCACCGTGTCCGGTCCTCGAGCGTATGGGGCTCTGTCACCCGCTCGTGTAGGTCCGGCC
GGCGGCCAGCCCGACCCTTTAACCAATCTTTTAACCAGGTTGACCTCGGATCAGGTAGGGAT
ACCCGCTGAACTTAA
k__Fungi;p__Ascomycota;c__Eurotiomycetes;o__Eurotiales;f__Trichocomaceae;g__Aspergillus;s__Aspergillus_vitricola 1.000

>OTU_41
GAAATGCGATAAGTAATGTGAATTGCAGAATTCAGTGAATCATCGAATCTTTGAACGCACAT
TGCGCCCGCCAGTATTCTGGCGGGCATGCCTGTTCGAGCGTCATTTCAACCCTCAGGCCccc
AGTGCCTGGTGTTGGGGATCGGCCCAGCCTTCTCGCAAGGCCGCCGGCCCCGAAATCTAGTG
GCGGTCTCGCTGTAGTCCTCCTCTGCGTAGTAGCACAACCTCGCAGTTGGAACGCGGCGGTG
GCCATGCCGTTAAACACCCCACTTCTGAAAGTTGACCTCGGATCAGGTAGGAATACCCGCTG
AACTTAAGCATATCAATAAGCGGAGGAATCCGTAGGTGAACCTGCGGCATCGATGAAGAAC

k__Fungi;p__Ascomycota;c__Sordariomycetes;o__Hypocreales;f__Incertae_sedis;g__Myrothecium;s__Myrothecium_verrucaria 1.000

>OTU_42
AAAGTGCGATAATTATTGCGACTTGCATTCATAGTGAATCATCGAGTTCTTGAACGCATCTTGCGCCTAGTAGTCAATCTACTAGGCACAGTTGTTTCAGTATCTGCATCCACCAATCAACACGACTTGCTTGTGTTGGAACTGGGCTTACTTTTGATGGCATTTAGTTGCTGTCATGGCCTTAAATGTATTTAGTCCTAGGTGTTAACTTGTTAATGCCGGATGGAGACTCTAGAGTGCCTTAGAAGCAGCTTGGTTAGTGAGTTCATAATTCCAAGTGTTAGTCTTTTATTGAACTGGGTTTCTAGTCTATGGGACATGGAGTCGTAAGACTCTTAAACAACAAACTCACATTTAGATCTGAAATCAACTGAGACCACCCGCTGAACTTAAGCATATCAATAAGCGGAGGA
k__Fungi;p__Zygomycota;c__Incertae_sedis;o__Mucorales;f__Lichtheimiaceae;g__Lichtheimia;s__Lichtheimia_corymbifera 1.000

>OTU_43
GAAATGCGATAAGTAATGTGAATTGCAGAATTCAGTGAATCATCGAATCTTTGAACGCACATTGCGCCCATTAGTATTCTAGTGGGCATGCCTGTTCGAGCGTCATTTCAACCCCTAAGCACAGCTTATTGTTGGGACTCTACGGCTTCGTAGTTCCCCAAAGACATTGGCGGAGTGGCAGCAGTCCTCTGAGCGTAGTAATTCTTTATCTCGCTTTTGTTAGGCGCTGCCcccccGGCCGTAAAACCcccAATTttttCTGGTTGACCTCGGATCAGGTAGGAATACCCGCTGAACTTAAGCATATCAATAAGCGGAGGAGCATATCAATAAGCGGAGGAGCATATCAATAAGCGGAGGA
k__Fungi;p__Ascomycota;c__Sordariomycetes;o__Trichosphaeriales;f__Incertae_sedis;g__Khuskia;s__Nigrospora_oryzae 0.980

>OTU_44
GAAATGCGATAAGTAATGTGAATTGCAGATACAGTGAATCATCGAATCTTTGAACGCAAATGGCACTCTATGGTATTCCGTAGAGTACGTCTGTTTGAGCGTCGCGAACATCTCCACAATTAGTTtttttAATTAGTTGAGGGTTTTGAGGTTGTCATATAAACAATGACTCCCTTTAAAATAATTAGTGATGACCTTATGAATGGGTTAATACTGtgtgtTATAATGGATTACATCCATCACCAGTCAGagagTAATCTCGCCTTAGTAATTTGTAGTGATTGCTTCTAACTGCCAATTtttGGCAAACAACCTGATCAAATCGACCTCAAATCAGATGGGATTACCCGCTGAACTTAAGCATATCAATAAGCGGAGGA
k__Fungi;p__Basidiomycota;c__Wallemiomycetes;o__Wallemiales;f__Wallemiaceae;g__Wallemia ;S__Wallemia_sebi 0.980

>OTU_45
GAAATGCGATACGTAGTGTGAATTGCAGAATTCAGTGAATCATCGAATCTTTGAACGCACATTGCGCCCTTTGGTATTCCAAAGGGCATGCCTGTTCGAGCGTCATTTGTACCCTCAAGCTTTGCTTGGTGTTGGGCGTTtttGTCTTTGGCCCGCCAAAGACTCGCCTTAAAATGATTGGCAGCCGGCCTACTGGTTTCGCAGCGCAGCACATTtttGCGCTTGCAATCAGCAAAAGAGGACGGCAATCCATCAAGACTCCTTCTCACGTTTGACCTCGGATCAGGTAGGGATACCCGCTGAACTTAAGCATATCAATAAGCGGAGGAATCCGTAGGTGAACCTGCG
k__Fungi;p__Ascomycota;c__Dothideomycetes;o__Pleosporales;f__Pleosporaceae;g__Bipolaris;s__Bipolaris_spicifera 1.000

>OTU_46
GAAATGCGATAAGTAATGTGAATTGCAGAATTCAGTGAATCATCGAATCTTTGAACGCACAT
TGCGCTCCTTGGTATTCCGGGGAGCATGCCTGTTTGAGAATCAGTAAATTCATCGAACACGG
TTTGGTTTCATACCTGCTGTGATCGGATATGGGCCTCTTTGCGCCTGGCGCAATGGCCTGAA
ATTCAATGTCACGGAATCATTGGACACCAGTCCGGTTTCAAGCGCATGAAAGGGTTAGTTCt
ctcGATTACGTCTTGTAGTATCGTTCTGGTTTTGAAGGACTTTGACTTCTGGGATTCAACTA
TCACTCCAGATGGCATTCTTTTAGGGTGCTTTCTGAACACTTGGTCTCAAATCAGGTAGGAA
AACCCGCTGAACTTAA
k__Fungi;p__Chytridiomycota;c__Chytridiomycetes;o__Rhizophlyctidales;f__Rhizophlyctidaceae;g__Rhizophlyctis;s__Rhizophlyctis_rosea 1.000

>OTU_47
GAAATGCGATAAGTAATGTGAATTGCAGAATTCAGTGAATCATCGAATCTTTGAACGCACCT
TGCGCTCCTTGGTATTCCTTGGAGCATGCCTGTTTGAGTATCATGATATCTTCAAAGTAAAT
CTTTTGTTAATTCAATTGGTTCTACTTTGGTATTGGAGGTTtttGCAGTTTCACACCTGCTC
CTCTTTGTGCATTAGCTGGATCTCAGTGTTATGCTTGGTTCCACTCGGCGTGATAAGTTATC
TATCGCTGAGGACACTGTAACAGGTGGCCAAGGTAAATGCAGATGAACCGCTTCTAATAGTC
CATTGACTTGGACAATATTtttATGATCTGATCTCAAATCAGGTAGGACTACCCGCTGAACT
TAAGCATATCAATAAGCGGAGGATCCGTAGGTGAACCTGCGGAGGA
k__Fungi;p__Basidiomycota;c__Agaricomycetes;o__Cantharellales;f__Ceratobasidiaceae;g__Rhizoctonia;s__Rhizoctonia_solani 0.980

>OTU_48
GAAATGCGATACTTGGTGTGAATTGCAGAATCCCGTGAACCATCGAGTCTTTGAACGCAAGT
TGCGCCCGAAGCCATCAGGCCGAGGGCACGTCTGCCTGGGCGTCACGCACCGTTGCCCTCCC
AACATCACCGTCCTTCGAGGCTACGTTTTGTTGCGAAGGCGCATATTGGCTTCCCGTGAGCT
TTGCCTCGTGGTTGGCCCAAAATCTTAGTCGTTGGCGACCCGTGCCGCGACGACGGTGGTTG
TCGAAACTTCGGTGCCCTGTCGTgtgtgCGGGTCGTTCGTGACGCAAGGACCCAACGCATTC
GAATGTGATGCCTCTCGATGCGACCCCAGGTCAGGCGGGGCTACCCGCTGAGTTTAAGCATA
TCAATAAGCGGAGGA
Hippophae tibetana

>OTU_49
GAAATGCGATAAGTAATGTGAATTGCAGAATTCAGTGAATCATCGAGTCTTTGAACGCACAT
TGCGCCcccTGGTATTCCGGggggCATGCCTGTCCGAGCGTCATTGCACCCCTCAAGCCCGG
CTTGTCCTTGGGTTGTGTCCCTCCGTTCCGGggggACAGGCCTCAAATGCAATGGCGGCACC
GCGTCCGGTCCTCGAGCGTATGGGGCTTTGTCACCCGCTTTGTAGGCCGGCCGGTCGCTTGC
CCGTCAAGCACAACTTCTTATGTTGACCTCGGATCAGGTAGGGATACCCGCTGAACTTAAGC
ATATCAATAAGCGGAGGAATCCGTAGGTGAACCTGCGGA
k__Fungi;p__Ascomycota;c__Eurotiomycetes;o__Eurotiales;f__Trichocomaceae;g__Penicillium;s__Penicillium_georgiense 1.000

>OTU_50
AAAGTGCGATAACTAGTGTGAATTGCATATTCAGTGAATCATCGAGTCTTTGAACGCAGCTT
GCACTCTATGGTTtttCTATAGAGTACGCCTGCTTCAGTATCATCACAAACCCAcacaTAAC
ATTTGTTTATGTGGTGATGGGTCGCATCGCTGTTTTATTACAGTGAGCACCTAAAATGtgtg
tgATTTTCTGTCTGGCTTGCTAGGCAGGAATATTACGCTGGTCTCAGGATCTTtttttttGG
TTCGCCCAGGAAGTAAAGTACAAGAGTATAATCCAGGAACTTTCAAACTATGATCTGAAGTC
AGGTGGGATTACCCGCTGAACTTAAGCATATCAATAAGCGGAGGAGTCCGTAGGTGAACCTG
CGGAGCATATCAATAAGCGGAGGA
k__Fungi;p__Zygomycota;c__Incertae_sedis;o__Mucorales;f__Mucor
aceae;g__Rhizopus;s__Rhizopus_oryzae 1.000

>OTU_51
GAAATGCGATAAGTAATGTGAATTGCAGAATTCAGTGAATCATCGAATCTTTGAACGCAACT
TGCGCCCTCTGGTATTCCGGAGGGCATGCCTGTTTGAGTGTCATGTAGACTCGATCCCTCGG
GTTTCCGAGGAGATTGGATTTGGGTGTCTGCCTCTTCTGGCTCACCTCAAAAGACTTAGCGG
GATAGCACCGTAGTCGACGTAATAAGTTTCGTCGGTAAAAGTGTGATGTCTGCTCACAATCG
CCCCTGGGCACTTTTGACTCTGACCTCAAATCAGGTAGGACTACCCGCTGAACTTAAGCATA
TCAATAAGCGGAGGAGCATATCAATAAGCGGAGGA
Uncultured fungus

>OTU_52
GAAATGCGATAAGTAATGTGAATTGCAGAATTCCGTGAATCATCGAATCTTTGAACGCACAT
TGCGCCcccTGGCATTCCGGggggCATGCCTGTCCGAGCGTCATTTCTGCCCTCCAGCACGG
CTGGGTGTTGGGTGCTGTCCccccGGGGACACGCCCCAAAAGCAGTGGCGGCGCCGCGTCGG
GTCCTCGAGCGTATGGGGCTCTGTCACCCGCTCGGGAGGGACTCGGTCGGCGCTGGTCTTCC
TCTAGGCGACCCTTCGGGGCTCGTCTCCTCCGGTTGACCTCGGATCAGGTAGGGCTACCCGC
TGAACTTAA
k__Fungi;p__Ascomycota;c__Eurotiomycetes;o__Eurotiales;f__Tric
hocomaceae;g__Talaromyces;s__Talaromyces_loliensis 0.980

>OTU_53
GAAATGCGATACTTGGTGTGAATTGCAGAATCCCATGAACCATCGATTCCTTGAACGCAAGT
TGCGCCCGAAGCCCTTAGGCTGAGGGCACGCCTGCCTGGGTGTCACCAAAAGTCGCCccccc
GTCTCGCCTGTCCCAGGGCACGGGGAGGgggCAAACGTTGGCCTCCCGGGAGCCCCTGGCTC
GCGGTTGGTTCAAaaaGACGGGCTCTTGGTGGAGAGCGGCACCGCGGCAGATAGTGGTCGAG
AACAACACTCGTGGCCAGTTGCGCGTGCCTCTCCcccGGTTCAAGGCACGGCGACCTCAGGT
CAGGCGGGGCTACCCGCTGAGTTTAA
Arachis hypogaea

>OTU_54
GAAATGCGATAAGTAATGTGAATTGCAGAATTCAGTGAATCATCGAATCTTTGAACGCACAT
TGCGCCCGCCAGTATTCTGGCGGGCATGCCTGTTCGAGCGTCATTTCAACCCTCAAGCTCAG
CTTGGTGTTGGGACTCGCGGTAACCCGCGTTCCCCAAATCGATTGGCGGTCACGTCGAGCTT

CCATAGCGTAGTAATCATACACCTCGTTACTGGTAATCGTCGCGGCCACGCCGTAAAACCCC
AACTTCTGAATGTTGACCTCGGATCAGGTAGGAATACCCGCTGAACTTAA
k__Fungi;p__Ascomycota;c__Sordariomycetes;o__Hypocreales;f__Ne
ctriaceae;g__Fusarium 0.990

>OTU_55
AAATCGCGATATGTAATGTGATCTGCCTATAGTGAATCATCAAATCTTTGAACGCATCTTGC
ACCTTATGGTATTCCATAAGGTACGTCTGTTTCAGTACCACTAATAAATCtctctctATCCT
TGATGATAGAAaaaaaaGAGATAAATTATTACTGGTCCTGGTGATTCTTtttttttttttAT
TAAaaaGAACCACTCTCGGCCTAAATATAAGGCTCGACTTttttttACCAGATCTTGCATCT
AGTAAaaaCCTAGTCGGCTTTAATAGATTtttATTTTCTATTAAGTTTATAGCCATTCTTAT
ATTttttAAAATCTTGGCCTGAAATCAGATGGGACTACCCGCTGAACTTAA
k__Fungi;p__Zygomycota;c__Incertae_sedis;o__Mucorales;f__Cunni
nghamellaceae;g__Cunninghamella;s__Cunninghamella_bertholletia
e1.000

>OTU_56
AAACTGCGATAAGTAGCGTGAATTGCAGACGCTTTGAACGTTAAACTTTCGAACGCACATTG
CGCCGTAGGAGTCCTACCCTGCGGCACATCTGGTTGAGGGTCGTGATCAAaaaCAGCAGGAC
TATGGCTGTAATAGAAATAGCTGGTGAATCACGAGTTCGGCCTACCGCCTCTGGCCGAACGT
GTTTTGCCATAATGCTAGAAGAGGACAATCACTCTGCTCGAGTGATTGCCTACCACACGGAT
GATCTATTAGCTGAAGCAATAGGTCTCGGTCACCGGGTATCATTCTTAtataCCATGTGGCG
TCTGAGATACAGCTAGTGGGCTTCATACGTGCCTGGTATTGAGAGGAGTATTCGCTCTCCCG
ACCTCAACTCAGGTGTGATTACCCGCTGAACTTAAGCATATCAATAAGCGGAGGA
Pratylenchus goodeyi

>OTU_57
GAAATGCGATAATTAATGTGAATTGCAGAATTCAGTGAATCATCGAGTCTTTGAACGCACAT
TGCGCCcccTGGTATTCCGGggggCATGCCTGTCCGAGCGTCATTGCTGCCCTCAAGCACGG
CTTGtgtgtTGGGCCcccGTCCccccTCCAAAGGGGACGGGCCCGAAAGGCAGCGGCGGCAC
CGTGTCCGGTCCTCGAGCGTATGGGAAGCAACTTtttGTCACCCGCTCCTGTAGGTCCGGCC
GGCGGCCTGCCCAACCCCAATCAATCTTTTAACCAGGTTGACCTCGGATCAGGTAGGGATAC
CCGCTGAACTTAA
k__Fungi;p__Ascomycota;c__Eurotiomycetes;o__Eurotiales;f__Tric
hocomaceae;g__Aspergillus;s__Aspergillus_penicillioides 0.990

>OTU_58
AAAATGCGATAAGTAATGTGAATTGCAGAATTCAGTGAATCATCGAATCTTTGAACGCACAT
TGCGCCCGCCAGTATTCTGGCGGGCATGCCTGTCTGAGCGTCATTTCAACCCTCGGGACCCC
GTTCGCGGGACCCGGCGTTGGGGATCAGCCCGAAGCCcccAGGGCGGGCGGCTGGCCCCGAA
ATCTAGTGGCGGTCCccccAGCGACCTCCTCTGCGCAGTAGTAACTCACCTCGCAGCTGGAC
AGCGGGAGGGCCACGCCGTAAAACCcccAACTTCTCAAAGGTTGACCTCAGATCAGGTAGGA
ATACCCGCTGAACTTAA
k__Fungi;p__Ascomycota;c__Sordariomycetes;o__Hypocreales;f__In

certae_sedis;g__Acremonium;s__Acremonium_sp_OUCMBI101028 1.000

>OTU_59
GAAATGCGATAAGTAATGTGAATTGCAGAATTCCGTGAATCATCGAATCTTTGAACGCACAT
TGCGCCccccGGAATGCCTGTCCGAGCGTCATTTCTGCCCTCAAGCACGGCTTGtgtgtTGG
GTGTGGTCCccccGGGGACCTGCCCGAAAGGCAGCGGCGACGTCCGTCTGGTCCTCGAGCGT
ATGGGGCTCTGTCACTCGCTCGGGAAGGACCTGCGGgggTTGGTCACCACCATGTTtttACC
ACGGTTGACCTCGGATCAGGTAGGAGTTACCCGCTGAACTTAA
k__Fungi;p__Ascomycota;c__Eurotiomycetes;o__Eurotiales;f__Tric
hocomaceae;g__Penicillium ;s__Penicillium_pinophilum 0.960

>OTU_60
CAAATGCGATAAGTAATGTGAATTGCAGAATTCAGTGAATCATCGAATCTTTGAACGCACAT
TGCGCCCGCCAGTATTCTGGCGGGCATGCCTGTTCGAGCGTCATTACAACCCTCAGGCCccc
GGGCCTGGCGTTGGGGATCGGCGGAAGCCcccTGCGGGCACAACGCCGTCCcccAAATACAG
TGGCGGTCCCGCCGCAGCTTCCATTGCGTAGTAGCTAACACCTCGCAACTGGAGAGCGGCGC
GGCCACGCCGTAAAACACCCAACTTCTGAATGTTGACCTCGAATCAGGTAGGAATACCCGCT
GAACTTAAGCATATCAATAAGCGGAGGAGTCCGTAGGTGAACCTGCG
k__Fungi;p__Ascomycota;c__Sordariomycetes;o__Hypocreales;f__Ne
ctriaceae;g__Fusarium;s__Fusarium_solani 1.000

>OTU_61
GAAATGCGATAAGTAATGTGAATTGCAGATACAGTGAATCATCGAATCTTTGAACGCACATT
GCGCCCCTTGGTATTCCGGggggCATGCCTGTTCGAGCGTCATTACAACCCTCAAGCTCTGC
TTGGAATTGGGCACCGTCCTCACTGCGGACGCGCCTCAAAGACCTCGGCGGTGGCTGTTCAG
CCCTCAAGCGTAGTAGAATACACCTCGCTTTGGAGTGGTTGGCGTCGCCCGCCGGACGAACC
TTCTGAACTTTTCTCAAGGTTGACCTCGGATCAGGTAGGGATACCCGCTGAACTTAAGCATA
TCAATAAGCGGAGGAGGAGTCCGTAGGTGAACCTGCGGA
k__Fungi;p__Ascomycota;c__Dothideomycetes;o__Botryosphaeriales
;f__Botryosphaeriaceae;g__Lasiodiplodia;s__Lasiodiplodia_theob
romae 0.990

>OTU_62
GAAATGCGATAAGTAATGTGAATTGCAGAATTCAGTGAATCATCGAATCTTTGAACGCACAT
TGCGCCCGCCAGTACTCTGGCGGGCATGCCTGTTCGAGCGTCATTTCAACCCTCAAGCTCTG
CTTGGTGTTGGGGATCGGCGTAGTGCCTTCGGGCGCTGCCGTCCCCTAAATCTAGTGGCGGT
CCCGCTGTAGCCTCCTCTGCGTAGTAGTTTTCTCCTCGCAATGGAATCTCGGCGGTGCCTTG
CCGTTAAACCcccAACTTCTGAAAGGTTGACCTCGAATCAGGTAGGAATACCCGCTGAACTT
AAGCATATCAATAAGCGGAGGAATCCGTAGGTGAACCTGCGGA
Uncultured fungus

>OTU_63
GAAATGCGATAAGTAATGTGAATTGCAGAATTCAGTGAATCATCGAGTCTTTGAACGCACAT
TGCGCCcccTGGTATTCCGGggggCATGCCTGTCCGAGCGTCATTGCTGCCCTCAAGCACGG

CTTGtgtgtTGGGCTCTCGCCccccGCTTCCGGggggCGGGCCCGAAAGGCAGCGGCGGCAC
CGCGTCCGGTCCTCGAGCGTATGGGGCTTCGTCACCCGCTCTGTAGGCCCGGCCGGCGCCCG
CCGGCGAACACCATCAATCTTAACCAGGTTGACCTCGGATCAGGTAGGGATACCCGCTGAAC
TTAA
k__Fungi;p__Ascomycota;c__Eurotiomycetes;o__Eurotiales;f__Tric
hocomaceae;g__Penicillium;s__Penicillium_oxalicum 1.000

>OTU_64
GAAATGCGATAACTAATGTGAATTGCAGAATTCAGTGAATCATCGAGTCTTTGAACGCACAT
TGCGCCcccTGGTATTCCGGggggCATGCCTGTCCGAGCGTCATTTCTCCCCTCCAGCCCCG
CTGGTTGTTGGGCCGCGCCccccccGGgggCGGGCCTCGAgagaAACGGCGGCACCGTCCGGT
CCTCGAGCGTATGGGGCTCTGTCACCCGCTCTATGGGCCCGGCCGGGGCTTGCCTCGACCcc
cAATCTTCTCAGATTGACCTCGGATCAGGTAGGGATACCCGCTGAACTTAAGCATATCAATA
AGCGGAGGAATCCGTAGTGAACCTGCGGCATATCAATAAGCGGAGGAATCCGTAGGTGAACC
TGCGGAGCATCGATGAAGAAC
k__Fungi;p__Ascomycota;c__Eurotiomycetes;o__Eurotiales;f__Tric
hocomaceae;g__Aspergillus ;s__Aspergillus_aculeatus 1.000

>OTU_65
GAAATGCGATAAGTAATGTGAATTGCAGAATTCAGTGAATCATCGAATCTTTGAACGCACAT
TGCGCCCGCCAGAATTCTGGCGGGCATGCCTGTTCGAGCGTCATTTCAACCCTCGGTCTCCC
TCCGGGAgagaCCGGCGTTGGGGACCGGCACTTACCCCGCCGGCCCCGAAATGAAGTGGCGG
CCCGTCCGCGGCGACCTCTGCGTAGTAACTCCACTCGCACCGGAACCCGGGCGCGGCCACGC
CGTAAAACCcccAACTTCCGAATGTTGACCTCGAATCAGGTAGGAATACCCGCTGAACTTAA
GCATATCAATAAGCGGAGGA
k__Fungi;p__Ascomycota;c__Sordariomycetes;o__Hypocreales;f__In
certae_sedis;g__Acremonium;s__Acremonium_sp 06857 0.990

>OTU_66
GAAATGCGATAAGTAATGTGAATTGCAGAATTCAGTGAATCATCGAATCTTTGAACGCACCT
TGCGCTCCTTGGTATTCCGAGGAGCATGCCTGTTTGAGTGTCATGAAACTCTCACCCTCTAG
CTTTCTTAATCGTGGCTAGCGGCGTGGACGTGAGCGCTGCTGCTTTGTTGCGGCTCGCTCGA
AATGCATTAGCAGACCCTTTTCGTAATCGGTTCCACTCAACGTGATAAGTATTTCGTTGAGG
ACAGTTGCAGCAATGCGGCTGGCCGGGATAAGAAAGGCATAGTTGTCAGCTTCTAATCGCCC
TTGGGCAATTttttATGATCTGGCCTCAAATCAGGTAGGACTACCCGCTGAACTTAAGCATA
TCAATAAGCGGAGGA
k__Fungi;p__Basidiomycota;c__Tremellomycetes;o__Cystofilobasid
iales;f__Cystofilobasidiaceae;g__Guehomyces;s__Guehomyces_pull
ulans 1.000

>OTU_67
AGATCAATACGGAAGCCCGGCTTGAAGTTGCGGTCCATCACCATCGGCGCTTTGGCATCCAG
CACGGTACTGCCCGCCAGTCCACCGCGAATTGCCTGATAAACCAGGTCCGGGTTAACGCCCG
CTTTAGTTGCCAGCGTTAACGCTTCTGACATCGCGGCAATATTCAGCGCCACAATGACCTGA

TTTGCCAGTTTGGTGACGTTACCTGCACCGATTTCCCCGGTATGCACCACGGAACCCGCCAT
CGCTTTCATCAAATCATAGTATTTGTCGAAAATAGCCTTGTCGCCGCCCACCATCACTGACA
GCGTACCGTCGATGGCTTTCGGTTCACCGCCGCTCACCGGAGCATCCA
Escherichia coli 1.000

>OTU_68
GAAATGCGATAAGTAATGTGAATTGCAGAATTCAGTGAATCATCGAATCTTTGAACGCACAT
TGCGCCCCTTGGTATTCCGAGGGGCATGCCTGTTCGAGCGTCATTACACCACTCAAGCACTG
CTTGGTATTGGGCATCGTCCGTCGAAAGGCGGGCGTGCCTCGAAGACCTCGGCGGGGTTTCT
CCAACTTCGGGCGTAGTAGAGTTAAATCGAACGTCTTATAAGCTTGGTGGGACTCCATTGCC
GTTAAACCTTTTATTTTCTAGGTTGACCTCGGATCAGGTAGGGATACCCGCTGAACTTAA
k__Fungi;p__Ascomycota;c__Dothideomycetes;o__Dothideales;f__Do
thideaceae;g__Dothidea;s__Dothidea_sp_CanS_64 1.000

>OTU_69
GAAATGCGATAAGTAATGTGAATTGCAGAATTCAGTGAATCATCGAATCTTTGAACGCACCT
TGCGCCTTTTGGTATTCCGAAAGGCATGCCTGTTTGAGTGTCATGAAATCTCAATCCcccTG
GGTTTATGATCTGGGTCGGACTTGGATATGGGCGTCTGCCGGTCACACGGCTCGCCTCAAAT
GACTTAGTGGATCtctctGCATCCGTGACAGACGTAATAAGTTTCGTCTTGTCCCTTGCTTA
TGAGTCTGCTCATAACCTGCCATCGCGCACTTTAGACTCTGACCTCAAATCAGGTAGGACTA
CCCGCTGAACTTAA
k__Fungi;p__Basidiomycota;c__Tremellomycetes;o__Filobasidiales
;f__Filobasidiaceae;g__Cryptococcus;s__Cryptococcus_laurentii
1.000

>OTU_70
GAAATGCGATACTTGGTGTGAATTGCAGAATCCCGTGAATCATTGAGTCTTTGAACGCAAGT
TGCGCCTGAAGCCCTTAGGCTGAGGGTACGCCTGCCTGGGTGTCACCAAAAGGCGCCccccA
TTTCGCCCGTCCCAAGGCACGGGGAGGgggCGAACGTTGGCCTCCCGGGAGCCCCTGGCTCG
CGGTTGGTTCAAAGAGACGGGTTCTTGGTGGAGAGCGGCACCGCGACAGATGGTGGTCGAGA
ACAACCCTCGTGGCCAGCCGcgcgcgcCTCTCCcccGGTTCAAGGCACGGTGACCCGCGGGC
GACGTGGATCGTCTCGAGCGCAACCTCAGGTCAGGCGGGGCTACCCGCTGAGTTTAA
Arachis hypogaea

>OTU_71
TGGATGCTCCGGTGAGCGGCGGTGAACCGAAAGCCATCGACGGTACGCTGTCAGTGATGGTG
GGCGGCGACAAGGCTATTTTCGACAAATACTATGATTTGATGAAAGCGATGGCGGGTTCCGT
GGTGCATACCGGGGAAATCGGTGCAGGTAACGTCACCAAACTGGCAAATCAGGTCATTGTGG
CGCTGAATATTGCCGCGATGTCAGAAGCGTTAACGCTGGCAACTAAAGCGGGCGTTAACCCG
GACCTGGTTTATCAGGCAATTCGCGGTGGACTGGCGGGCAGTACCGTGCTGGATGCCAAAGC
GCCGATGGTGATGGACCGCAACTTCAAGCCGGGCTTCCGTATTGATCT
Escherichia coli

>OTU_72

GAAATGCGATAAGTAATGTGAATTGCAGAATTCAGTGAATCATCGAATCTTTGAACGCACAT
TGCGCCCGCCAGCATTCTGGCGGGCATGCCTGTTCGAGCGTCATTTCAACCCTCAAGCTCAG
CTTGGTGTTGGGGCCCTACGCCTGGCGTAGTCCCCTAAAGGTAGTGGCGGACCCTCTCGGAG
CCTCCTTTGCGTAGTAACTAACGTCTCGCACTGGGATCCGGAGGGACCTCTCGCCGTAAAAC
CcccAATTtttCTTAGGTTGACCTCGGATCAGGTAGGAATACCCGCTGAACTTAA
k__Fungi;p__Ascomycota;c__Sordariomycetes;o__Glomerellales;f__
Glomerellaceae;g__Colletotrichum;s__Colletotrichum_chlorophyti
1.000

>OTU_73
GAAATGCGATAAGTAGTGTGAATTGCAGAATTCAGTGAATCATCGAATCTTTGAACGCACAT
TGCGCCCCTCGGTATTCCGTGGGGCATGCCTGTTCGAGCGTCATCTACACCCTCAAGCTCTG
CTTGGTGTTGGGCGTCTGTCCCGCCTCCGcgcgTGGACTCGCCCCAAATTCATTGGCAGCGG
TCCTTGCCTCCTCTCGCGCAGCACATTGCGCTTCTCGAGGGGCTACGGCTCGCGTCCAACAA
GCACATTTACCGTCTTTGACCTCGGATCAGGTAGGGATACCCGCTGAACTTAAGCATATCAA
TAAGCGGAGGAAaaaTGTGCATCGATGAAGAACGCA
k__Fungi;p__Ascomycota;c__Dothideomycetes;o__Pleosporales;f__P
leosporaceae;g__Leptosphaerulina;s__Leptosphaerulina_chartarum
1.000

>OTU_74
GAAATGCGATAAGTAATGTGAATTGCAGAATTCAGTGAATCATCGAATCTTTGAACGCACAT
TGCGCCCGCCAGTATTCTGGCGGGCATGCCTGTTCGAGCGTCATTTCAACCCTCAAGCCCCA
GCGGCTTGGTGTTGGGGACCGGCCCCGGCCGCCccccAAATGCAGTGGCGACCTCGCCGCAG
CCTCCCCTGCGTAGTAGCACAACTCGCACCGGAGCGCGGAGACGGTCACGCCGTAAAACGCC
CAACTTCTCAGAGTTGACCTCGGATCAGGTAGGAATACCCGCTGAACTTAA
k__Fungi;p__Ascomycota;c__Sordariomycetes;o__Hypocreales;f__Op
hiocordycipitaceae;g__Tolypocladium;s__Tolypocladium_inflatum
1.000

>OTU_75
TAACTGCGATAAGTAGCGTGAATTGCAGACGCTTTGAACGTTGAACTTTCGAACGCACATTG
CGCCGTAGGAGTTCTACCCTACGGCACATCTGGTTGAGGGTCGTGATCAAAACTAGCCGGAA
TCGTATGGCTATAAGagagGAATAGCTGGTGAATTACTCGGCGAGCCGATGCCTCCGGCTCG
CAGTGGTTCCCCATCATGCTATACAGGGGACACCCGCTCGCGGGAGCCCGCCACACGGATGA
TCGACGGCTAGCCGATGCCCGTAGATCCCGGTCCAACGGTATTATACTTATATGCTCGAGGA
CGTCTGGGATACGGCTCGCCGTATTGTGGTTGTACGTGCCCGTGGCGAATGCACTCGTTCTC
CCGACCTCAACTCAGGTGTGATTACCCGCTGAACTTAA
Pratylenchus goodeyi

>OTU_76
GAATTGCGTTAAGAATCACGAATTACAGATATTATGAGTGATATGTTTTCGATTGCATATTG
CATCGTTGGGCACTTGCCCATCGGTATACCCAACTCAGGGTgtgtATCACTAATCGGAAGAC
ACAATGCACTTGTTGGTTGTGTCTGAGCTTGGAGCTGTGCAATACCTGTGATTGCATTGTTT

TATTGAGGTGTGGCTGAATTGTAGAAATGAAGGAGAATGAGGTTGAAAATGCTTGGTGGTTG
TTGGAAGTTTAATGAATTtttGAGCTTTCGGTGACCGTTCGGTTtttCGACTTttttCAcac
aGCCTTGCATACACCAGCCAATTGTTtttGGTCTAATCCACCTGAGTCGGATATGATCACCC
GCCGAACTTAA
Aphelenchoides fragariae

>OTU_77
GAAATGCGATAAGTAATGTGAATTGCAGAATTCAGTGAATCATCGAATCTTTGAACGCACAT
TGCGCCCGCCAGCATTCTGGCGGGCATGCCTGTTCGAGCGTCATTTCAACCCTCGAGCCCGA
CTCTTTTGAGGACGGCTCGGCGTTGGgggACGGCAGACTCCccccGGCATCTCGCCGGGGAC
GCCGCCcccGAAATCTAGTGGCGGCCCTCCGAGGCGACCTCTGCGTAGTAACTTGTTCTCGC
ACCGGAAAGCTCAGAGCGGCCACGCCGTAAAACCCCAACTTttttCAGGTTGACCTCGAATC
AGGTAGGACTACCCGCTGAACTTAAGCATATCAATAAGCGGAGGA
k__Fungi;p__Ascomycota;c__Leotiomycetes;o__Helotiales;f__Incer
tae_sedis;g__Gloeotinia;s__Gloeotinia_temulenta 1.000

>OTU_78
GAAATGCGATACTTGGTGTGAATTACAGAATCCCGTGAACCATCGAGTCTTTTAACGCAAGT
TGCGCCCGAAGCCCTTAGGCTGAGGGCACGCCTGCCTGGGTGTCACCAAAAGGCACCccccG
TCCCGCCCGTCCGAGGGCACGGGGAGGgggCGAATGTTGGCCTCCCGGGAGCCCCTGGCTCG
CGGTTGGTTCAAAGAGACGGGCTCTTGGTGGGGAGCGGCACCGCGGCAGATGGTGATCGAGA
ACAACCCTCATGGCCAGTCGcgcgcgcTTCTCCcccGGTTCAAGGCACGGCGACCCGCGGGC
GACGTGGACCGTCCCGAGCGCGACCTCAGGTCAGGCGGGGCTATCCACTGAGTTTAA
Arachis hypogaea

>OTU_79
GAAATGCGATAACTAATGTGAATTGCAGAATTCAGTGAATCATCGAGTCTTTGAACGCACAT
TGCGCCCTCTGGTATTCCGGAGGGCATGCCTGTCCGAGCGTCATTGCTGCCCTCAAGCACGG
CTTGtgtgtTGGGCCcccGTCCccccccTCTGCCGGggggACGGGCCCGAAAGGCAGCGGCGG
CACCGCGTCCGGTCCTCGAGCGTATGGGGCTTCGTCACCCGCTCTTGTAGGCCCGGCCGGCG
CCAGCCGACCCCAACCCTAAATTtttttCAGGTTGACCTCGGATCAGGTAGGGATACCCGCT
GAACTTAA
k__Fungi;p__Ascomycota;c__Eurotiomycetes;o__Eurotiales;f__Tric
hocomaceae;g__Penicillium;s__Penicillium_meleagrinum 1.000

>OTU_80
GAAACGCGATATGTAATGTGAATTGCAGAATTCAGTGAATCATCGAATCTTTGAACGCACAT
GGCGCCTTCCAGTATCCTGGGAGGCATGCCTGTCCGAGCGTCGTTTCAACCCTCGAGCCccc
GTGGCCCGGCGTTGGGGACCTGCCCAGGCAGTCCCCGAAAACCAGTGGCGGACCCGACGGGC
CCTTCCTTTGCGTAGTAACATCTGCCTCGCATCGGGAGCCcccGGGCTATCCGGCCTCTAAA
CCccccTCAAGCCCGCTCCGGCGGCACCAAGGTTGACCTCGGATCAGGTAGGAATACCCGCT
GAACTTAAGCATATCAATAAGCGGAGTCCGTAGGTGAACCTGCGGA
k__Fungi;p__Ascomycota;c__Sordariomycetes;o__Incertae_sedis;f_
_Plectosphaerellaceae;g__Gibellulopsis;s__Gibellulopsis_nigres

cens	1.000

>OTU_81
AAAATGCGATAAGTAATGTGAATTGCAGAATTCAGTGAATCATCGAATCTTTGAACGCACAT
TGCGCCCGCCAGTATTCTGGCGGGCATGCCTGTTCGAGCGTCATTTCAACCCTCAAGCCCAG
CTTGGTGTTGGGAGCTGCAGTCCTGCTGCACTCCCCAAATACATTGGCGGTCACGTCGAGCT
TCCATAGCGTAGTAATTTACACATCGTTACTGGTAATCGTCGCGGCCACGCCGTTAAACCCC
AACTTCTGAATGTTGACCTCGGATCAGGTAGGAATACCCGCTGAACTTAAGCATATCAATAA
GCGGAGGAATCCGTAGGTGAACCTGCGGCATGAATTGT
k__Fungi;p__Ascomycota;c__Sordariomycetes;o__Hypocreales;f__Ne
ctriaceae;g__Gibberella;s__Gibberella_zeae	1.000

>OTU_82
GAAATGCGATAAGTAATGTGAATTGCAGATACAGTGAATCATCGAATCTTTGAACGCAAATG
GCACTCTATGGTATTCCGTAGAGTACGTCTGTTTGAGCGTCGCGAACATCTCTATAATTAGT
TtttttttAAAATTGATTATGGGTTTTGAGGTTGTCATGTATAATGACTCCCTTTAAAATAA
TTAGTGATGACCTTATGAATGGGTTAATACTGtgtgtTATAATGGATTACATCCATCACCAG
TCAGagagTAATCTCGCCTTAGTAATTTGTAGTGATTGCTTCTAACTGCCAATTTGGCAAAC
AACCTGATCAAATCGACCTCAAATCAGATGGGATTACCCGCTGAACTTAA
k__Fungi;p__Basidiomycota;c__Wallemiomycetes;o__Wallemiales;f_
_Wallemiaceae;g__Wallemia;s__Wallemia_sebi	1.000

>OTU_83
GAAATGCGATACTTGGTGTGAATTGCAGAATCCCGTGAACCATCGAGTCTTTGAACGCAAGT
TGCGCCCGAAGCCCTTAGGCTGAGGGCACGCCTGCCTGGGTGTCACCAAAAGGCGCCccccG
TCCCGCCCGTCCGAATGTTGGCGAATGTTGGCCTCCCGGGAGCCCCTGGCTCGCGGTTGGTT
CAAAGAGACGGGCTCTTGGTGGGGAGCGGCACCGCGGCAGATGGTGGTCGAGAACAACCCTC
GTGGCCAGTCGcgcgcgcCTCTCCcccGGTTCAAGGCACGGCGACCCGCGGGCGACGTGGAT
CGTCCCGAGCGCGACCTCAGGTCAGGCGGGGCTACCCGCTGAGTTTAA
Arachis hypogaea

>OTU_84
TAACTGCGATAAGTAGCGTGAATTGCAGACGCTTTGAACGTTAAACTTTCGAACGCACATTG
CGCCGTAGGAGTTCTACCCTGCGGCACATCTGGTTGAGGGTCGTGATCAAaaaCTGCCCGAA
TGTAGCAGTATAGATAGCTGGCGAACCATGATGAAAGTCGTGATTTGCCATATAACGCTATA
GAGGATAGTCTCCTGTACGGgggACTACCTGCCACACGGATGATCTACTAGTTGACGCAGTA
GGTCCCGGTCCCAGGTATTATGCACGAAACGTATTATGCCGAGGGACGTCAGGGATACGGCT
AGTGGgggAATCTGTGCGAGCTATATGAGCTATTCGATTTCCCGACCTCAACTCAGGTGTGA
TTACCCGCTGAACTTAAGCATATCAATAAGCGGAGGA
Pratylenchus goodeyi

>OTU_85
GAATTGCGTTAAGAATCACGAATTACAGATATTATGAGTGATATGTTTTCGATTGCATATTG
CATCGTTGGGCACTTGCCCATCGGTATACCCAACTCAGGGTgtgtATCACTAATCGGAAGAC

ACAATGCACTTGTTGGTTGTGCCTGAATTCGGAGCTGTGCCAATACTTGTATTGCATTGTTT
TATTTAAGGTGTGGCTGAATTGTAGAAATGAAGGAGAATGAGGTTGAAAATGCTTGGTGGTT
GTTGGAGGTTTAATGAATTtttGAGCTTTTGATGACTACTCGGTTttttGACTATTtttACA
CAGCCCTATACacacCAACCAATCGTTtttGGTTTAATCCACCTGAGTCGGATATGATCACC
CGCCGAACTTAA
Aphelenchoides ritzemabosi

>OTU_86
GAAATGCGATAAGTAATGTGAATTGCAGAATTCAGTGAATCATCGAATCTTTGAACGCACAT
TGCGCCCATTAGTATTCTAGTGGGCATGCCTGTTCGAGCGTCATTTCAACCCCTAAGCACAG
CTTACTGTTGGGACTCTACGGCCTCCGTAGTTCCCCAAAGCGATTGGCGGAGTGGCAGTAGT
CCTCTGAGCGTAGTAATTttttATCTCGCTTTTGTTAGGTGCTGCCccccccGGCCGTTAAAC
CccccccAATTttttCTGGTTGACCTCGGATCAGGTAGGAATACCCGCTGAACTTAA
k__Fungi;p__Ascomycota;c__Sordariomycetes;o__Trichosphaeriales
;f__Incertae_sedis;g__Khuskia;s__Nigrospora_oryzae 1.000

>OTU_87
GAAATGCGATAAGTAATGTGAATTGCAGAATTCAGTGAATCATCGAATCTTTGAACGCACAT
TGCGCCCGCCAGTATTCTGGCGGGCATGCCTGTCCGAGCGTCATTTCAACCCTCGAACCCCT
CCGGgggATCGGCGTTGGGGATCGGGACCCCTCACCGGGTGCCGGCCCTGAAATACAGTGGC
GGTCTCGCCGCAGCCTCTCCTGCGCAGTAGTTTGCACAACTCGCACCGGGAGCGCGGCGCGT
CCACGTCCGTAAAACACCCAACTTCTGAAATGTTGACCTCGGATCAGGTAGGAATACCCGCT
GAACTTAA
k__Fungi;p__Ascomycota;c__Sordariomycetes;o__Hypocreales;f__Hy
pocreaceae;g__Trichoderma;s__Trichoderma_hamatum 1.000

>OTU_88
GAAATGCGATAAGTAATGTGAATTGCAGAATTCAGTGAATCATCGAATCTTTGAACGCACCT
TGCGCTCCTTGGTATTCCTTGGAGCATGCCTGTTTGAGTATCATGAAATCTTCAAAGTCAAA
CCTTTTGTTAACTCAATTGGTTCTGCTTTGGTATTGGAGGTCTATTGCAGCTTCACACCTGC
TCCTCTTTGTGCATTAGCTGGATCTCAGTGTTATGCTTGGTTCCACTCAGCGTGATAAGTTA
TCTATCGCTGAGGACACCCTGTTAAaaaGGGGTGGCCAAGGTAAATGCAGATGAACTGCTTC
TAACAGTCCATTGACTTGGACAAATATTAATTTTATGATCTGATCTCAAATCAGGTAGGACT
ACCCGCTGAACTTAA
k__Fungi;p__Basidiomycota;c__Agaricomycetes;o__Cantharellales;
f__Ceratobasidiaceae;g__Rhizoctonia;s__Rhizoctonia_solani 1.000

>OTU_89
GAAATGCGATACTTGCATCGAGTCTTTGAACGCAAGTTGCGCCCGAAGCCCTTAGGCTGAGG
GCACGCCTGCCTGGGTGTCACCAAAAGGCGCCCCTCGTCCCGCCCGTCCGAGGGCACGGGGA
GGgggCGAATGTTGGCCTCCCGGGAGCCCCTGGCTCGCGGTTGGTTCAAAGAGACGGGCTCT
TGGTGGGGAGCGGCACCGCGGCAGATGGTGGTCGAGAACAACCCTCGTGGCCAGTCGcgcgc
gcCTCTCCcccGGTTCAAGGCACGGCGACCCGCGGGCGACGTGGATCGTCCCGAGCGCGACC
TCAGGTCAGGCGGGGCTACCCGCTGAGTTTAA

Arachis hypogaea

>OTU_90
GAAATGCGATAAGTAATGTGAATTGCAGAATTCAGTGAATCATCGAATCTTTGAACGCACAT
TGCGCCCGCCAGTATTCTGGCGGGCATGCCTGTTCGAGCGTCATTTCAACCATCAAGCCccc
GGCTTGTGTTGGGGACCTGCGGCTGCCCGCAGGCCCTGAAAACCAGTGGCGGGCTCGCTGTC
ACACCGAGCGTAGTAGCATACATCTCGCTCAGGGCGTGCTGCGGGTTCCGGCCGTTAAACGA
CCTCTATAACCCAAGGTTGACCTCGGATCAGGTAGGAAGACCCGCTGAACTTAAGCATATCA
ATAAGCGGAGGAGCATCGATGAGAACGCAGCGTCGTCGCATCGATGAAGAACGCA
k__Fungi;p__Ascomycota;c__Sordariomycetes;o__Sordariales;f__Chaetomiaceae;g__Chaetomium;s__Chaetomium_sp_TPL42 1.000

>OTU_91
AAACTACGTTAATCAGTGTAAACTGCACAATAGCAGAACACTTGAACTTCGAACGCACATTG
CGGCTAAAGGGTAACTCCTCTAGCCTTATCTGCGTGAGGGCTGTATAGCGATACTCAACTCA
TGTTTAGGGAGTGATCACTTTACCGTGAGACTCCGCGACGTGAACCTCGCTAGTGGTCTCTG
ACTGCTGACGTCTCAGGCAGGCAAGACCACGGGTTAGCGAGTCGCTAACACGAGAGTGTTTA
CACTCCCTGTGTTTCCACTACAACAGCCTCACGTCAGGTAAGATCACCCGCTGAACTTAA
No similar sequence

>OTU_92
GAAATGCGATAAGTAATGTGAATTGCAGAATTCAGTGAATCATCGAATCTTTGAACGCACAT
TGCGCCcccTGGTATTCCGGggggCATGCCTGTTCGAGCGTCATTACAACCCTCAAGCTCTG
CTTGGTATTGGGCCCCGCCGGTTCCGGCGGGCCCTAAAGTCAGTGGCGGTGCCATCCGGCTC
CGAGCGTAGTAATTCTTCTCGCTCTGGAGACCCGGCTGtgtgCTTGCCAGCAACCcccAATT
ttttAAGGTTGACCTCGGATCAGGTAGGGATACCCGCTGAACTTAA
k__Fungi;p__Ascomycota;c__Leotiomycetes;o__ Leotiomycetes incertae sedis;f__ Pseudeurotiaceae;g__Pseudeurotium;s__Pseudeurotium_sp_T5_10_-1 1.000

>OTU_93
GAAATGCGATAAGTAATGTGAATTGCAGAATTCAGTGAATCATCGAATCTTTGAACGCACAT
TGCGCCCATTAGTATTCTAGTGGGCATGCCTGTTCGAGCGTCATTTCAACCCTTAAGCCTAG
CTTAGTGTTGGGAGACTGCCTAATACGCAGCTCCTCAAAACCAGTGGCGGAGTCTGTTCGTG
CTCTGAGCGTAGTAATTCTTTATCTCGCTTCTGCAAGCCGATTAGACAACAGCCATAAACCG
CACCCTTCGGgggCACTTtttAATGGTTGACCTCGGATCAGGTAGGAATACCCGCTGAACTT
AA
k__Fungi;p__Ascomycota;c__Sordariomycetes;o__Xylariales;f__Hyponectriaceae;g__Microdochium;s__Microdochium_nivale 1.000

>OTU_94
GAAATGCGATACTTGGTGTGAATTGCAGAATCCCGTGAACCATCGAGTCTTTGAACGCAAGT
TGCGCCCGAAGCCCTTAGGCTGAGGGCACGCCTGCCTGGGTGTCACCAAAAGGCGCCTCCCG

TCTCGCCCGTCCCAGGGGCCTCCCGGGAGCCCCTGGCTCGCGGTTGGTTCAAAGAGACGGGC
TCTTGGTGGGGAGCGGCACCGCGGCAGATGGTGGTCGAGAACAACCCTCGTGGCCAGTCGcg
cgcgcCTCTCCcccGGTTCAAGGCACGGCGACCCGCGGGCGACGTGGATCGTCCCGAGTGCG
ACCTCAGGTCAGGCGGGGCTACCCGCTGAGTTTAA
Arachis hypogaea

>OTU_95
GAACTGCGATAAGTAATGTGAATTGCAGAATTCAGTGAATCATCGAGTCTTTGAACGCACAT
TGCGCCcccTGGCATTCCGGggggCATGCCTGTCCGAGCGTCATTGCTGCCCATCAAGCCCG
GCTTGtgtgtTGGGTCGTCGTCCcccccGGgggACGGGCCCGAAAGGCAGCGGCGGCACCGT
GTCCGGTCCTCGAGCGTATGGGGCTTTGTCACCCGCTCGACTAGGGCCGGCCGGGCGCCAGC
CGACGTCTCCAACCATTtttCTTCAGGTTGACCTCGGATCAGGTAGGGATACCCGCTGAACT
TAA
k__Fungi;p__Ascomycota;c__Eurotiomycetes;o__Eurotiales;f__Tric
hocomaceae;g__Aspergillus;s__Aspergillus_versicolor   1.000

>OTU_96
GAAATGCGATAAGTAATGTGAATTGCAGAATTCAGTGAATCATCGAATCTTTGAACGCACAT
TGCGCCCTTTGGTATTCCGAAGGGCATGCCTGTTCGAGCGTCATTATCAACCATCAAGCTCT
GCTTGGCATTGGGTGTCAACCTCCCCTAACCGGGCGGTCGCGCCTCAAACTGTTCGGCGGTG
GCTCAGGGCCTCAAGCGTTAGTAATACTTCCCGCTTCAGAGAAACTGAGTTGCCTGCCTCTA
GAAACCCACATCTTAAGGTTGACCTCGGATCAGGTAGGGATACCCGCTGAACTTAA
Uncultured fungus 1.000

>OTU_97
GAAATGCGATAAGTAGTGTGAATTGCAGAATTCAGTGAATCATCGAATCTTTGAACGCACAT
TGCGCCCTATGGTATTCCGTAGGGCATGCCTGTTCGAGCGTCATTCAACCCCTCAAGCTCTG
CTTGGTGTTGGGCGTCTGTCCCGCCTCACGGCGCGGACTCGCCTCAAATCTATTGGCGGCCG
GCACGTTGGCTTCGAGCGCAGCAGAAACGCGAACTCGAGGCCCGGCGGATCGGCTCCCAGAA
GCTACCccccATGAATTTGACCTCGGATCAGGTAGGGATACCCGCTGAACTTAA
k__Fungi;p__Ascomycota;c__Dothideomycetes;o__Pleosporales;f__S
porormiaceae;g__Preussia; s__Preussia_funiculata 1.000

>OTU_98
GAAATGCGATAATTAATGTGAATTGCAGAATTCAGTGAATCATCGAGTCTTTGAACGCACAT
TGCACCcccTGGTATTCCGGggggTATGCCTGTCCGAGCGTCATTGCTGCCCTCAAGCACGG
CTTGtgtgtTGGGTCGTCGTCCccccccAGGgggACGGGCCCGAAAGGCAGCGGCGGCACCG
CGTCCGGTCCTCGAGCGTATGGGGCTTTGTCACCCGCTCTTGTAGGCCCGGCCGGCTGCTGG
CCGACGCTGAAAAGCAACCAACTATTtttCCAGGTTGACCTCGGATCAGGTAGGGATACCCG
CTGAACTTAA
k__Fungi;p__Ascomycota;c__Eurotiomycetes;o__Eurotiales;f__Tric
hocomaceae;g__Aspergillus;s__Aspergillus_ochraceus 1.000

>OTU_99

GAAGTGCGATAAGCAATGCGAATTGCAGAACCGTGAGTCATCAGATTtttGAACGCAACTGG
CGCTGGCTGGATCTCCAGCCAGCATGCTTGTTTCAGTGTCTTGTTTCCTCCTCACCCAAACC
TTAATGCGAgagaTACCCTTCTCTTGTCAAGCACGAAAGCACTCTGCGCTCTGCGAGCGGCT
TCTGACTAGCTCAGAGGTCGCACTCAATGCAGACGTCACATTTCTCACAATGTGAACTCATT
GAGAGTAGAGGCTGTCAGCTCACGCTGTCAGTGCTTAGTCACTCAAACTTTCTTTGCATCTG
AAATCAAGCAGGATCACCCGCTGAACTTAA
Uncultured fungus

>OTU_100
GAAATGCGATACTTGGTGTGAATTGCAGAATCCCGTGAACCATCGAGTCTTTGAACGCAAGT
TGCGCCCGACGCCTTCGGGCTGAGGGCACGCCTGCCTGGGCGTCACGCATCGCGTCGCCccc
TATACCAATTTGGTGCGGggggCGGATAATGGCATCCCGTTAGCTTGGTTTGCCCAAaaaGG
ATCCCTCATCGACGGATGTCACAACCAGTGGTGGTTGAAAGATCATTGGTGTTGTTGTGCAT
CACTCTGTCGCATGCTTGGGCATCGTTATAAAACAATGGTGCTAACGCGCCTTCGACCGCGA
CCCCAGGTCAGACGGGACTACCCGCTGAGTTTAA
Plantago camtschatica

>OTU_101
GAAATGCGATACGTAATGTGAATTGCAGAATTCAGTGAATCATCGAGTCTTTGAACGCACAT
TGCGCCCTCTGGTATTCCGGggggCATGCCTGTCCGAGCGTCATTGCTGCCCTCAAGCACGG
CTTGtgtgtTGGGCCcccGTCCCGGTTCTTTAAGCCGGGACGGGCCCGAAAGGCAGCGGCGG
CACCGTGTCCGGTCCTCGAGTGTATGGGGCTCTGTCACCCACTCGTGTAGGTCCGGCCGGCG
GCCAGCCTCTTCAACCAAACTTTTAACCAGGTTGACCTCGGATCAGGTAGGGATACCCGCTG
AACTTAA
k__Fungi;p__Ascomycota;c__Eurotiomycetes;o__Eurotiales;f__Tric
hocomaceae;g__Aspergillus;s__Aspergillus_restrictus 1.000

>OTU_102
GAAATGCGATAAGTAATGTGAATTGCAGAATTCCGTGAATCATCGAATCTTTGAACGCACAT
TGCGCCcccTGGCATTCCGGggggCATGCCTGTCCGAGCGTCATTTCTGCCCTCAAGCACGG
CTTGtgtgtTGGGTGTGGTCCccccGGGGACCTGCCCGAAAGGCAGCGGCGACGTCCGTCTG
GTCCTCGAGCGTATGGGGCTCTGTCACTCGCTCGGGAAGGACCTTCGGgggTTGGTCACCAC
CATGTTtttACCACGGTTGACCTCGGATCAGGTAGGAGTTACCCGCTGAACTTAAGCATATC
AATAAGCGGAGGAGCATATCAATAAGCGGAGGA
k__Fungi;p__Ascomycota;c__Eurotiomycetes;o__Eurotiales;f__Tric
hocomaceae;g__Penicillium; g__Penicillium_pinophilum 0.990

>OTU_103
AAACTACGTTAATCAGTGTAAACTGCACAATAGCAGAACACTTGAACTTCGAACGCACATTG
CGGCTAAAGGGTAACTCCTCTAGCCTTATCTGCGTGAGGGCTGTATAGCGATACTCAACTCA
TGTTTAGGGAGTGATCACTTTACCGTGAGACTCCGCGACGTGAACCTCGCTAGTGGTCTCTG
ACTGCTGACGTCTCAGGCAGGCAAGACCACGGGTTAGCGAGGAGTACACTAACACAGTgtgt
gtTAACAACAGCCTCACGTCAGGTAAGATCACCCGCTGAACTTAA
No similar sequence

>OTU_104
GAAATGCGATACGTAGTGTGAATTGCAGAATTCAGTGAATCATCGAATCTTTGAACGCACAT
TGCGCCCTTTGGTATTCCAAAGGGCATGCCTGTTCGAGCGTCATTTGTACCTTCAAGCTTTG
CTTGGTGTTGGGCGTCTTttttttttGTCTTGGGGCCTTTTGTGCCCTGAGACTCGCCTTAA
AACGATTGGCAGCCGGCCTACTGGTTTCGGAGCGCAGCACATTtttGCGCTTGCAATCAGCA
AAAGAGGCCAGCCATCCATCAAGACCATTCTTCTCACTTTTGACCTCGGATCAGGTAGGGAT
ACCCGCTGAACTTAA
k__Fungi;p__Ascomycota;c__Dothideomycetes;o__Pleosporales;f__P
leosporaceae;g__unidentified;s__Pleosporaceae_sp_LH40 1.000

>OTU_105
GAAATGCGATAAGTAATGTGAATTGCAGATACAGTGAATCATCGAATCTTTGAACGCACATT
GCGCCCATTAGTATTCTAGTGGGCATGCCTGTTCGAGCGTCATTTCAACCCCTAAGCACAGC
TTATTGTTGGGCGTCTACGTCTGTAGTGCCTCAAAGACATTGGCGGAGCGGCAGCAGTCCTC
TGAGCGTAGTAATTCTTTATCTCGCTTCTGTTAGGCGCTGCCccccccGGCCGTAAAACCccc
AATTttttCTGGTTGACCTCGGATCAGGTAGGAATACCCGCTGAACTTAAGCATATCAATAA
GCGGAGGA
k__Fungi;p__Ascomycota;c__Sordariomycetes;o__Trichosphaeriales
;f__Incertae_sedis;g__Khuskia;s__Nigrospora_sphaerica 0.990

>OTU_106
GAAATGCGATAAGTAATGTGAATTGCAGATACAGTGAATCATCGAATCTTTGAACGCAAATG
GCACTCTATGGTATTCCGTAGAGTACGTCTGTTTGAGCGTCGCGAACATCTCCATAATTGGT
TtttttAAATTGATTGCGGGTTTTGAGGTTGTCATATAACAATGACTCCCTTTAAAATAATT
AGTGATGACCTTATGAATGGGTTAATACTGtgtgtTATAATGGATTACATCCATCACCAGTC
AGagagTAATCTCGCCTTAGTAATTTGTAGTGATTGCTTCTAACTGCCATTGGCAAACAAAC
TGATCAAATCGACCTCAAATCAGATGGGATTACCCGCTGAACTTAAGCATATCAATAAGCGG
AGGAGCATATCAATAAGCGGAGGA
k__Fungi;p__Basidiomycota;c__Wallemiomycetes;o__Wallemiales;f_
_Wallemiaceae;g__Wallemia 1.000

>OTU_107
GAAATGCGATAAGTAATGTGAATTGCAGAATTCAGTGAATCATCGAATCTTTGAACGCACAT
TGCGCCCTATGGTATTCCGTAGGGCATGCCTGTTCGAGCGTCATTACacacCTCAAGCTCTG
CTTGGTATTGGGCGTCGTCCCCTAAACGGACGTGCCTCAAAGACCTCGGCGGTGGCGTCTTT
GCCTCAAGCGTAGTAATACTTTTATCTCGCTTTGGAGTCGAAGGCGTCGCCCGCCGGACGAA
ACCTTTATTATTTCTATCAAGGTTGACCTCGGATCAGGTAGGGATACCCGCTGAACTTAA
k__Fungi;p__Ascomycota;c__Dothideomycetes;o__Botryosphaeriales
;f__Botryosphaeriaceae;g__Aplosporella;s__Aplosporella_hesperi
dica 1.000

>OTU_108
GAATTGCGTTAAGAATCACGAATTACAGATATTATGAGTGATATGTTTTCGATTGCATATTG

CATCGTTGGGCACTTGCCCATCGGTATACCCAACTCAGGGTgtgtATCACTAGTTGAAAGAC
ACAATGCACTTGTTGGTTGTTTCTAAAATTTGGAGGTTTGAAGTACTAGTTACTTCAAACCT
ACTGTTACGGTGTGGCTGAATTGTAGAAATGAAGGAGAATGAGGTTGAAAATACTTGGTGGT
TGTTGGAAGTTTAATGAATTtttGAGCTTTCGGTGACTATTCGGTTtttCGACTATTtttAC
ACAGCCCTATACacacCAGCCAATCGTTtttGGTTTAATCCACCTGAGTCGGATATGATCAC
CCGCCGAACTTAA
Aphelenchoides fragariae

>OTU_109
GAAATGCGATAAGTAATGTGAATTGCAGAATTCAGTGAATCATCGAATCTTTGAACGCACAT
TGCGCCCGCCAGTATTCTGGCGGGCATGCCTGTTCGAGCGTCATTTCAACCATCAAGCCCCG
GGCTTGTGTTGGGGACCTGCGGCTGCCGCAGGCCCTGAAATGCAGTGGCGGGCTCGCTGTCA
CTCCGAGCGTAGTAGTTACATCTCGCTCTGGGCGTGCTGCGGGTTCCGGCCGTTAAAAGCCT
TATTTACCCAAGGTTGACCTCGGATCAGGTAGGAAGACCCGCTGAACTTAA
k__Fungi;p__Ascomycota;c__Sordariomycetes;o__Sordariales;f__Ch
aetomiaceae;g__Humicola;s__Humicola_fuscoatra 1.000

>OTU_110
GAAATGCGATAAGTAATGTGAATTGCAGAATTCAGTGAATCATCGAATCTTTGAACGCACAT
TGCGCCCTTTGGCATTCCGAAGGGCATGCCCGTTCGAGCGTCATTACACCAATCAAGCCTGG
CTTGGTATTGGGCGTCGGGGACCCTCCcccGCGCCcccAATTCGTCGGCTGGACGGTTCGAA
TCTCAGCGTTGTGGTCAATCAATTCGCTGGCGACGACGACCGGACGCGCCGTTAAACCTCAC
TACAGGTTGACCTCGGATCGGGTAGGGATACCCGCTGAACTTAAGCATATCAATAAGCGGAG
GA
k__Fungi;p__Ascomycota;c__Dothideomycetes;o__Capnodiales;f__Te
ratosphaeriaceae;g__Devriesia;s__Devriesia_sp_NG_p52 1.000

>OTU_111
GAAATGCGATACTTGGTGTGAATTGCAGAATCCCGTGAACCATCGAGTCTTTGAACGCAAGT
TGCGCCCCAAGCCTTTGGCCGAGGGCACGTCTGCCTGGGTGTCACAAATCGTCGTCCccccc
AATCCTTTTGGATAAGGGACGGAAGTTGGTCTCCCGTGTGATACCGCATGCGGTTGGCCGAA
ATCCGAGCTTAGGACGTCAGGAGCGTCTCGACATGCGGTGGTGAAAATCTCCCTCATTATGT
CGGTCGTTCCTGTCCATAAGCTCTCGATGACCCAAAGTCCTCAACGCGACCCCAGGTCAGGC
GGGATCACCCGCTGAGTTTAA
Braya glabella

>OTU_112
GAAATGCGATAAGTAATGTGAATTGCAGATACAGTGAATCATCGAATTtttGAACGCATATT
GCGCCTTGTGGTAATCCGCAAGGCATGCCTGTCTGAGCGTCATTACATCATTCAAGTAAAAG
TTttttttttttACTTGGTCTTGGAATTTGACTTtttACCAACTTGTTGGTAGTTATGTCTG
TTCTGTAATGTATGATTGCAGTCTATTGACACTTTTAGTTGTAGTACATACCATCAACTTTA
AAAGCCTCTAGACCTGGTCACCTTTACCATCAATCGCTTGATTGAAATTTTCTTGTAAAGTT
TGACCTCAGATCAGGTAGGACTACCCGCTGAACTTAAGCATATCAATAAGCGGAGGAGCATA
TCAATAAGCGGAGGA

Uncultured fungus

>OTU_113
GAAATGCGATACCTGGTGTGAATTGCAGAATCCCGCGAACCATCGAGTCTTTGAACGCAAGT
TGCGCCCGAGGCCACTCGGCCGAGGGCACGCCTGCCTGGGCGTCACGCCAAAACACGCTCCC
AACCACTCTCAACGGGAATCGGGATGCGGCATCTGGTCCCTCGTCtctcAAGGGACGGTGGA
CCGAAGATTGGGCTGCCGGCGTACCGCGCCGGACACAGCGCATGGTGGGCGTCCTCGCTTTA
TCAATGCAGTGCATCCGGCgcgcAGCTGGCATTATGGCCTTTGAACGACCCAACAAACGAAG
CGCACGTCGCTTCGACCGCGACCCCAGGTCAGGCGGGACTACCCGCTGAGTTTAA
k__Fungi;p__Ascomycota;c__Eurotiomycetes;o__Eurotiales;f__Tric
hocomaceae;g__Emericella;s__Emericella_nidulans 1.000

>OTU_114
AAAGTGCGATAACTAGTGTGAATTGCATATTCAGTGAATCATCGAGTCTTTGAACGCAGCTT
GCACTCTATGGTTtttCTATAGAGTACGCCTGCTTCAGTATCATCACAAACCCAcacaTAAC
ATTTGTTTATGTGGTGATGGGTCGCATCGCTGTTTTATTACAGTGAGCACCTAAAATGtgtg
tgATTTTCTGTCTGGCTTGCTAGGCAGGAATATTACGCTGGTCTCAGGATCTTtttttttGG
TTCGCCCAGGAAGTAAAGTACAAGAGTATAATCCAGTAACTTTCAAACTATGATCTGAAGTC
AGGTGGGATTACCCGCTGAACTTAAGCATATCAATAAGCGGAGGATGAGTCCGTAGGTGAAC
CTGCGGCATCGATGAAGAACGCA
k__Fungi;p__Zygomycota;c__Incertae_sedis;o__Mucorales;f__Mucor
aceae;g__Rhizopus;s__Rhizopus_oryzae 1.000

>OTU_115
GAAATGCGATAACTAATGTGAATTGCAGAATTCAGTGAATCATCGAGTCTTTGAACGCACAT
TGCGCCcccTGGTATTCCGGggggCATGCCTGTCCGAGCGTCATTGCTGCCCTCAAGCCCGG
CTTGtgtgtTGGGTGTGGTCCccccGGGGACCTGCCCGAAAGGCAGCGGCGGCACCGCGTCC
GATCCTCGAGCGTATGGGGCTTTGTCACATGCTCTGTAGGATTGGCCGGCGCCTGCCGACGT
TTTCCAACCATTCTTTCCAGGTTGACCTCGGATCAGGTAGGGATACCCGCTGAACTTAA
k__Fungi;p__Ascomycota;c__Eurotiomycetes;o__Eurotiales;f__Tric
hocomaceae;g__Aspergillus s__Aspergillus_niger 0.960

>OTU_116
GAAATGCGATAAGTAATGTGAATTGCAGAATTCAGTGAATCATCGAATCTTTGAACGCACAT
TGCGCCCACTAGTATTCTGGTGGGCATGCCTGTTCGAGCGTCATTTCAACCCTCAAGCCTGG
CTTGGTGTTGGGGCTCTGCGCAACGCAGTCCCTTAAATTCAGTGGCGGACACGCTAGGTCTC
CGAGCGCAGTAGTTTCTTCTCGCTTCGGGCGTCCGGCGTGGGCTTGCCTCGCACCCAACTTC
TCAAGGTTGACCTCGGATCAGGTAGGAATACCCGCTGAACTTAA
k__Fungi;p__Ascomycota;c__Sordariomycetes;o__Incertae_sedis;f_
_Incertae_sedis;g__Myrmecridium;s__Myrmecridium_schulzeri 1.000

>OTU_117
AAACTACGTTAATCAGTGTAAACTGCACAATAGCAGAACACTTGAACTTCGAACGCACATTG
CGGCTAAAGGGTAACTCCTCTAGCCTTATCTGCGTGAGGGCTGTATAGAGATATTGAACCCA

TGTGTAGGGAGTGATCACTTGAACGTGAGACTCCGCAGAGTGAACCTCGCTAGTGGTCTCTG
ACTGCTGACGTCTCAGGCAGGCAAGACCGCGGGTTAGCGAAGGTGACTAACACTCAcacacT
TATCTACAACAGCCTCACGTCAGGTAAGATCACCCGCTGAACTTAA
No significant similarity

>OTU_118
AAAGTGCGATAATTATTGCGACTTGCATTCATAGTGAATCATCGAGTTCTTGAACGCATCTT
GCGCCTAGTAGTCAATCTACTAGGCACAGTTGTTTCAGTATCTGCATCCACCAATCAACACT
ACTTGTGTTGGAACTGGGCTTACTTTGATGGCATTCAGTTGCTGTCATGGCCTTAAATGTAT
TTAGTCCTAGGTGTTAACTTGTTAATGCCGGATGGAGACTCTAGAGTGCCTTAGAAGCAACT
TGGTTAGTGAGTTCATAATTCCAAGTGTTAGTCTTTTATTGAACTGGGTTTCTAGTCTATGG
GACTTACTAGGGAGACTAGATCtctcAAGTAAACCAAACTCACATCTAGATCTGAAATCAAC
TGAGACCACCCGCTGAACTTAA
k__Fungi;p__Zygomycota;c__Incertae_sedis;o__Mucorales;f__Licht
heimiaceae;g__Lichtheimia;s__Lichtheimia_sp_AA_2009a 1.000

>OTU_119
AAAATGCGATAAGTAATGTGAATTGCAGAATTCAGTGAATCATCGAATCTTTGAACGCACAT
TGCGCCCGCCAGTATTCTGGCGGGCATGCCTGTTCGAGCGTCATTTCAACCCTCAAGCCccc
GGGTTTGGTGTTGGGGATCGGCGAGCCCTTGCGGCAAGCCGGCCCCGAAATCTAGTGGCGGT
CTCGCTGCAGCTTCCATTGCGTAGTAGTAAAACCCTCGCAACTGGTACGCGGCGCGGCCAAG
CCGTTAAACCcccAACTTCTGAATGTTGACCTCGGATCAGGTAGGAATACCCGCTGAACTTA
A
k__Fungi;p__Ascomycota;c__Sordariomycetes;o__Hypocreales;f__Ne
ctriaceae;g__Fusarium;s__Fusarium_proliferatum 1.000

>OTU_120
GAAATGCGATAAGTAATGTGAATTGCAGAATTCCGTGAATCATCGAATCTTTGAACGCACAT
TGCGCCcccTGGCATTCCGGggggCATGCCTGTCCGAGCGTCATTTCTGCCCTCAAGCACGG
CTTGtgtgtTGGGTCGCCGTCCcccTCTCCGGggggACGGGCCCGAAAGGCAGCGGCGACGT
CCGTCTGGTCCTCGAGCGTATGGGGCTCTGTCACTCGCTCGGGAAGGACCTGCGGgggTTGG
TCACCACCATGTTtttACCACGGTTGACCTCGGATCAGGTAGGAGTTACCCGCTGAACTTAA
k__Fungi;p__Ascomycota;c__Eurotiomycetes;o__Eurotiales;f__Tric
hocomaceae;g__Penicillium 0.960

>OTU_121
GAAATGCGATACTTGGTGTGAATTGTAGAATCCCGTGAACCATCGAGTCTTTGAACGCAAGT
TGCACCCGAAGCCCTTAGGCTGAGGGCACGCCTGCCTGGGTGTCACCAAaaaGCGCCccccT
GTCTCGCCCATCCCAAGGCCCGGGGAGGgggCGAACGTTGGCCTCCCGGGAGCCCCTGGCTC
GCGGTTGGTTCAAAGAGACGGGCTCTTGGTGGGGAGCGGCACCGCGGCAGATGGTGGTCGAG
AACAACCCTCGTGGCCAGTCGcgcgTGCCTCTCCccccGGTTCAAGGCACGGCGACCCGCGGG
CGACGTGGATCGTCCCGAGCGTGACCTCAGGTCAGGCGGGGCTACCCGCTGAGTTTAAGCAT
ATCAATAAGCGGAGGA
Arachis hypogaea

>OTU_122
GAAATGCGATACGTAATGTGAATTGCAGAATTCAGTGAATCATCGAGTCTTTGAACGCACAT
TGCGCCcccTGGTATTCCGGggggCATGCCTGTCCGAGCGTCATTGCTGCCCTCAAGCACGG
CTTGtgtgtTGGGCCCCGCCccccGGTCCCGGggggCGGCCCGAAAGGCAGCGGCGGCACC
GCGTCCGGTCCTCGAGCGTATGGGGCTTTGTCACCCGCTCCGTAGGCCCGGCCGGCGCCCGC
CGGCGACCcccAATCAATCTATCCAGGTTGACCTCGGATCAGGTAGGGATACCCGCTGAACT
TAA
k__Fungi;p__Ascomycota;c__Eurotiomycetes;o__Eurotiales;f__Tric
hocomaceae;g__Penicillium;s__Penicillium_ochrochloron 1.000

>OTU_123
GAAATGCGATAATTAATGTGAATTGCAGAATTCAGTGAATCATCGAATCTTTGAACGCACAT
TGCGCCCGCCAGTATTCTGGCGGGCATGCCTGTTCGAGCGTCATTACAACCCTCAGGCCccc
GGGCCTGGCGTTGGGGATCGGCGGAAGCCccccTGTGGGCACACGCCGTCCCTCAAATACAG
TGGCGGTCCCGCCGCAGCTTCCATTGCGTAGTAGCTAACACCTCGCAACTGGAGAGCGGCGC
GGCCATGCCGTAAAACACCCAACTTCTGAATGTTGACCTCGAATCAGGTAGGAATACCCGCT
GAACTTAA
k__Fungi;p__Ascomycota;c__Sordariomycetes;o__Hypocreales;f__Ne
ctriaceae;g__Fusarium;s__Fusarium_solani 0.990

>OTU_124
GAAATGCGATAAGTAATGTGAATTGCAGAATTCAGTGAATCATCGAATCTTTGAACGCACAT
TGCGCCcccTGGTATTCCGGggggCATGCCTGTTCGAGCGTCATTTCACCACTCAAGCCTCG
CTTGGTATTGGGCGACGCGGTCCGCCGcgcgcCTCAAATCGACCGGCTGGGTCTTTCGTCCC
CTCAGCGTTGTGGAAACTATTCGCTAAAGGGTGCCGCGGGAGGCCACGCCGTAAAACAACCC
CATTTCTAAGGTTGACCTCGGATCAGGTAGGGATACCCGCTGAACTTAA
k__Fungi;p__Ascomycota;c__Dothideomycetes;o__Capnodiales;f__My
cosphaerellaceae;g__Cladosporium 1.000

>OTU_125
GAAATGCGATAAGTAATGTGAATTGCAGAATTCAGTGAATCATCGAATCTTTGAACGCACAT
TGCGCCCGCCAGTATTCTGGCGGGCATGCCTGTCCGAGCGTCATTTCAACCCTCGAACCCCT
CCGGggggTCGGCGTTGGGGATCGGCCCTTTACGGGGCCGGCCCCGAAATACAGTGGCGGTC
TCGCCGCAGCCTCTCCTGCGCAGTAGTTTGCACACTCGCATCGGGAGCGCGGCGCGTCCACA
GCCGTTAAACACCCCAAACTTCTGAAATGTTGACCTCGGATCAGGTAGGAATACCCGCTGAA
CTTAA
k__Fungi;p__Ascomycota;c__Sordariomycetes;o__Hypocreales;f__Hy
pocreaceae;g__Trichoderma;s__Trichoderma_virens 1.000

>OTU_126
TAACTGCGATAAGTAGCGTGAATTGCAGACGCTTTGAACGTTAAACTTTCGAACGCACATTG
CGCCGTAGGAGTTCTACCCTGCGGCACATCTGGTTGAGGGTCGTGATCAAaaaCTGCCCGAA
TGTAGCAGTATAGATAGCTGGCGAACCATGATGAAAGTCGTGATTTGCCATACGACGCTATA

GAGGACAATTCTCCACGTGAgagagTTGCCTGCCACACGGATGATCTACTAGTTGACGCAGT
AGATCCCGGTCCCAGGTATCGTATTATGCCGAGGGATGCGCGGGGATACAGCTAGTGAgaga
ATCTGTGCGGCGCTATGAGCTATTCGATTTCCCGACCTCAACTCAGGTGTGATTACCCGCTG
AACTTAA
Pratylenchus goodeyi

>OTU_127
GAAATGCGAAAAGTAATGTGAATTGCAGAATTCAGTGAATCATCGAATCTTTGAACGCACAT
TGCGCCCGCTGGTACTCCGGCGGGCACGCCTGTCCGAGCGTCATTTCAACCCTCAAGCCCTC
CTTCGGGATGGGCCTGGTGTTGGGGCTCGGCCGCCATCTCCGGCGGCCGTCCCCTAAATACA
GTGGCGGTCACGTCGCGACTCCTTTGCGTAGTAGAAACACCTCGCTCCGGATAGCAACGCGG
TCCACGCCGTAAAACCcccAATTTTCAATGGTTGACCTCGGATCAGGTGGGAATACCCGCTG
AACTTAA
k__Fungi;p__Ascomycota;c__Sordariomycetes;o__Hypocreales;f__Hy
pocreales incertaesedis;g__Acremonium;s__Acremonium_sp_Zi105
1.000

>OTU_128
GAAATGCGATAACTAATGTGAATTGCAGAATTCAGTGAATCATCGAGTCTTTGAACGCACAT
TGCGCCcccTGGTATTCCGGggggCATGCCTGTCCGAGCGTCATTACTGCCCTCAAGCCCGG
CTTGTATTGGGTCCTCGTCCcccTCCCCGGgggACGGGCCCGAAAGGCAGCGGCGGCACCGC
GTCCGGTCCTCGAGCGTATGGGGCTTTGTCACCCGCTCTGTAGGCCCGGCCGGCGCCAGCCC
ACGCAACACCTTttttttCAGGTTGACCTCGGATCAGGTAGGGATACCCGCTGAACTTAA
k__Fungi;p__Ascomycota;c__Eurotiomycetes;o__Eurotiales;f__Tric
hocomaceae;g__Aspergillus;s__Aspergillus_flavipes 1.000

>OTU_129
GAAATGCGATAAGTAATGTGAATTGCAGAATTCAGTGAATCATCGAATCTTTGAACGCACAT
TGCGCCcccTGGTATTCCGGggggCATGCCTGTCCGAGCGTCATTACAACCCTCAAGCTCAG
CTTGGTATTGGGCCCCGCCGACCCGGCGGGCCCTAAAGTCAGTGGCGGTGCCGTCCGGCTCC
GAGCGTAGTAATTCTTCTCGCTCTGGAGGTCCGGTCGTGTGCTCGCCAGCAACCcccAATTt
tttttCAGGTTGACCTCGGATCAGGTAGGGATACCCGCTGAACTTAA
k__Fungi;p__Ascomycota;c__Dothideomycetes;o__Incertae_sedis;f_
_Pseudeurotiaceae;g__Pseudogymnoascus;s__Pseudogymnoascus_pann
orum 0.990

>OTU_130
GAAATGCGATAAGTAATGCGAATTGCAGAATTCTCGTGAGTCATCGAATCTTTGAACGCACA
TTGCGCCCTTTGGTATTCCGAAGGGCATGCCTGTTCGAGCGTCATTTTCACCCCTCAAGCCT
CCGGCTTGGTGTTGGACGGTTTGGTCCAGGGTCCccccTGGACCCCTCCCAAAGACAATGAC
GGCGGGCTGTTGCACCcccGGTACACTGAGCGTCTTCACGGAGCACGTACCGGTCTCAAGGG
TCGACGGCACCCGGTCTACACCTATATCTTTCACAAGGTTGACCTCGGATCAGGTAGGAATA
CCCGCTGAACTTAAGCATATCAATAAGCGGAGGA
k__Fungi;p__Ascomycota;c__Eurotiomycetes;o__Chaetothyriales;f_

_Herpotrichiellaceae;g__Rhinocladiella;s__Rhinocladiella_simil
is 0.990

>OTU_131
GAACTGCGATAAGTAATGTGAATTGCAGAATTTTGTGAATCATCGAATCTTTGAACGCACAT
TGCGCCTTTTGGTATTCCAGAAGGCATACCTGTTTGAGAGTCATTTCTTCCTCAATCCTCGG
ATTGGTGTTGACTCGAGCGTTGCGAAACGCTGAGTCGAAAAGAATTGGCAGGCAGGAATTCA
GTGCTTTACAGTGTCTTAGGTTTTACCAACTACGCTGGCCTAGTAACTGTTTTCTGAGCTGG
CCTTTATAACAGTTCTTTTAAAAGTTTGACCTCAAATCAGGCAAGACTACCCGCTGAACTTA
A
k__Fungi;p__Ascomycota;c__Saccharomycetes;o__Saccharomycetales
;f__Trichomonascaceae;g__Arxula;s__Arxula_adeninivorans 1.000

>OTU_132
AAAGTGCGATAACTAGTGTGAATTGCATATTCGTGAATCATCGAGTCTTTGAACGCAGCTTG
CACTCTATGGATCTTCTATAGAGTACGCTTGCTTCAGTATCATAACCAACCCAcacaTAAAA
TTTATTTTATGTGGTGATGGACAAATTCGGTTAGATTTAATTATTATACCGATTGTCTAAAA
TACAGCCTCTTTGTAATTTTCATTAAATTACGAACTACCTAGCCATCGTGCTTTttttGGTCC
AACCAAaaaaCATTTAATCTAGGGGTTCTGCCAGCCAGCAGATATTTTAATGCTCTTTAACT
ATGATCTGAAGTCAAGTGGGACTACCCGCTGAACTTAA
k__Fungi;p__Zygomycota;c__Incertae_sedis;o__Mucorales;f__Mucor
aceae;g__Rhizopus;s__Rhizopus_microsporus 1.000

>OTU_133
GAAATGCGATAAGTAATGCGAATTGCAGAATTCAGTGAATCATCGAATCTTTGAACGCACAT
TGCGCCCGCCAGTACTCTGGCGGGCATGCCTGTCTGAGCGTCATTTCAACCCTCGCACCCGG
CTTCTGTCGGgggCGGTGTTGGGGATCGGCCTCCCGTCATCGGGCGGCCGGCCCCTAAATAG
AGTGGCGACCACGCCGTAGCCTCCTCTGCGTAGTAGTGAAACACTCGCGGGCGGAGAGCGGT
GCGGCCTGCCGTAAAACCcccAACTCTTTCTAAGGTTGACCTCAGATCAGGTAGGAATACCC
GCTGAACTTAA
k__Fungi;p__Ascomycota;c__Sordariomycetes;o__
Hypocreales;f__Hypocreales incertae sedis;g__Fusariella; s__
Fusariella_sinensis 1.000

>OTU_134
TAACTGCGATAAGTAGCGTGAATTGCAGACGCTTTGAACGTTGAACTTTCGAACGCACATTG
CGCCGTAGGAGTTCTACCCTACGGCACATCTGGTTGAGGGTCGTGATCAAAACTAGCCGGAA
TCGTATGGCTATAAGagagGAATAGCTGGCGAATTACTCGGCGAGCCGATGCCTCCGGCgcg
cAGTGGTTCACCATCATGCTATAGGGGACACCCATTTGCGGGAGCCCGCCACACGGATGATC
GACGGCTAGCCGATGCTCGTAGATCCCGGTCCAACGGTATTATACTTATATGCTCGAGGACG
TCCGGGATACGGCTTGCCATTGTGGTTGTACGTGCCCGTGGCGAATGCACTCGTTCTCCCGA
CCTCAACTCAGGTGTGATTACCCGCTGAACTTAA
Pratylenchus goodeyi

>OTU_135
GAAATGCGATACTTGGTGTGAATTGCAGAATTCAGTGAATCATCGAGTCTTTGAACGCACAT
TGCGCCcccTGGTATTCCGGggggCATGCCTGTCCGAGCGTCATTGCTGCCCATCAAGCACG
GCTTGtgtgtTGGGTCGTCGTCCCCTCTCCGGgggggACGGGCCCCAAAGGCAGCGGCGGCA
CCGCGTCCGATCCTCGAGCGTATGGGGCTTTGTCACCCGCTCTGTAGGCCCGGCCGGCGCTT
GCCGAACGCAAATCAATCTTTTCCAGGTTGACCTCGGATCAGGTAGGGATACCCGCTGAACT
TAAGCATATCAATAAGCGGAGGACGTACGGGATGAAGAACGCATATCAATAAGCGGAGGA
k__Fungi;p__Ascomycota;c__Eurotiomycetes;o__Eurotiales;f__Tric
hocomaceae;g__Aspergillus;s__Aspergillus_flavus 0.990

>OTU_136
TAGATACGTTAATCGGTGTTATATGCTATAGCCATAAGACATCACTCGACTATTCTAATGCG
CAATGCAGCTAGTCCCTTAAAGACTAGTTACATCTGATTGAGAGTATCATTAAGTTTGTTCA
ATAATTATTATTGAATGAGAACTGCATTTGACTTATAGTGATGACTATACTTGTATAGACGT
CATGTAAAAGCGATACTAAGGCACGCTCGTGTGCTTTGGATCGCTATTGTGTTTCACTATGA
AACTAATTTCAAaaaGCAAaaaaGCAATTtttAGCTATGTTTGAAGTAATCAAATGAATACT
CAGAACACGTATCTCAGTCAGGTGAGAATACCCGCTGAACTTAA
Acarapis woodi

>OTU_137
GAAATGCGATAACTAGTGTGAATTGCAGAATTCCGTGAATCATCGAGTCTTTGAACGCACAT
TGCGCCcccTGGTATTCCGGggggCATGCCTGTCCGAGCGTCATTGCTGCCCATCAAGCACG
GCTTGtgtgtTGGGTGTGGTCCccccGGGGACCTGCCCGAAAGGCAGCGGCGACGTCCGTCT
GGTCCTCGAGCGTATGGGGCTTTGTCACCCGCTCCCGTAGGTCCAGCTGGCAGCTAGCCTCG
CAACCAATCTTtttAACCAGGTTGACCTCGGATCAGGTAGGGATACCCGCTGAACTTAA
k__Fungi;p__Ascomycota;c__Eurotiomycetes;o__Eurotiales;f__Tric
hocomaceae   1.000

>OTU_138
GAAATGCGATAAGTAATGTGAATTGCAGAATTCAGTGAATCATCGAGTCTTTGAACGCACAT
TGCGCCcccTGGTATTCCGGggggCATGCCTGTCCGAGCGTCATTGCTGCCCTCAAGCACGG
CTTGtgtgtTGGGCTTCCGTCCCTGGTAACGGGGACGGGCCCAAAAGGCAGTGGCGGCACCA
TGTCTGGTCCTCGAGCGTATGGGAAGCAACTCTTtttGTCACCCGCTCCCGTAGGTCCAGCT
GGCAGCTAGCCTCGCAACCAATCTTtttAACCAGGTTGACCTCGGATCAGGTAGGGATACCC
GCTGAACTTAA
k__Fungi;p__Ascomycota;c__Eurotiomycetes;o__Eurotiales;f__Tric
hocomaceae;g__Eurotium;s__Eurotium_niveoglaucum 0.9730

>OTU_139
GAAACGCGATAAGTAATGTGAATTGCAGAATTCAGTGAATCATCGAATCTTTGAACGCACAT
GGCGCCTTCCAGTATCCTGGGAGGCATGCCTGTCCGAGCGTCGTTTCAACCCTCGAGCCccc
GTGGCCCGGCGTTGGGGATCTGCCCAGGCAGGCCCCGAAAACCAGTGGCGGACCCGTTACAG
GCCCTTCCTTTGCGTAGTAATACTGCCTCGCATCGGGAGCCGGCGGGCTTCCAGCCTCTAAA
CCcccAGCAAGTCCGCCCCGGCGGCACCAAGGTTGACCTCGGATCAGGTAGGAATACCCGCT

GAACTTAA
k__Fungi;p__Ascomycota;c__Sordariomycetes;o__Glomerellales;f__Plectosphaerellaceae;g__Chordomyces;s__Chordomyces_antarcticum 1.000

>OTU_140
AAACTACGTTAATCAGTGTAAACTGCACAATAGCAGAACACTTGAACTTCGAACGCACATTG
CGGCTAAAGGGTAACTCCTCTAGCCTTATCTGCGTGAGGGCTGTATAGAGATATTGAACCCA
TGTGTAGGGAGTGATCACTTGACGGTGAGACTCCGCAGAGTGAACCTCGCTAGTGGTCTCTG
ACTGCTGACGTCTCAGGCAGGCAAGACCGCGGGTTAGCGAAGGTGACTATCACTCAGTCAca
caAACACATCTACAACAGCCTCACGTCAGGTAAGATCACCCGCTGAACTTAAGCATATCAAT
AAGCGGAGGATCCGTAGGTGAACCTGCGGCATGAATTGT
No significant similarity

>OTU_141
GATCCGCGATACGTCCTGGAAGCCGCCGTGAACCATCAATTTTCGAACGCACATTGCATACA
GGGAGGGTGGGGTGAGTCTACTCTCACTATCGTGGTTTAAATACAAAGGATGACTAATACAA
TCGTCAGCCcccAAATTTTCTCCcccGCCcccTCAGATTACCCGCTGAACTTAAGCATATCA
ATAAGCGGAGGAGCATATCAATAAGCGGAGGAGCATATCAATAAGCGGAGGAGCATATCAAT
AAGCGGAGGATCCGTAGGTGAACCTGCG
k__Fungi;p__Ascomycota;c__Saccharomycetes;o__Saccharomycetales;f__Incertae_sedis;g__Candida;s__Candida_cellae 1.000

>OTU_142
GAAATGCGATAAGTAATGTGAATTGCAGAATTCCGTGAATCATCGAATCTTTGAACGCACAT
TGCGCCcccTGGCATTCCGGggggCATGCCTGTCCGAGCGTCATTTCTGCCCTCAAGCACGG
CTTGtgtgtTGGGTGTGGTCCccccGGGGACCTGCCCGAAAGGCAGCGGCGACGTCCGTCTG
GTCCTCGAGCGTATGGGGCTCTGTCACTCGCTCGGGAAGGACCTGCGGgggTTGGTCACCAC
CATGTTtttACCACGGTTGACCTCGGATCAGGTAGGAGTTACCCGCTGAACTTAAGCATATC
AATAAGCGGAGGATGAGTCCGTAGGTGAACCTGCGGAAGGATAGAC
k__Fungi;p__Ascomycota;c__Eurotiomycetes;o__Eurotiales;f__Trichocomaceae;g__Penicillium;s__Penicillium_pinophilum 1.000

>OTU_143
GAAATGCGATAAGTAATGTGAATTGCAGAATTCAGTGAATCATCGAATCTTTGAACGCACAT
TGCGCCCGCTAGCACTCTAGCGGGCATGCCTGTCCGAGCGTCATTTCAACCCTCAGACCccc
TTCGGggggACTGGCGTTGGGGATCGGCCCGCCTCAGTGCGGTGCCGTCCcccAAATACAGT
GGCGGTCTCGCTGCAGCCTCCCCTGCGTAGTAGCACACCTCGCATCGGAGAGCGGTGAGGCC
ACGCCGTGAAACCccccACTTCTGAACGTTGACCTCGGATCAGGTAGGAATACCCGCTGAAC
TTAA
k__Fungi;p__Ascomycota;c__Sordariomycetes;o__Hypocreales;f__Incertae_sedis;g__Stachybotrys;s__Stachybotrys_longispora 0.990

>OTU_144

GAAGTGCGATAAGCAATGCGAATTGCAGAACCGTGAGTCATCAGATTtttGAACGCAACTGG
CGCTGGCTGGGTCTCCAGCCAGCATGCTTGTTTCAGTGTCTTGTTTTCTCCTCACCCAAACC
TTAATGCGAgagaTACCCTTCTCTTGCCAAGCACGAAAGCACTCTGCGCTCTGCGAGCTTCT
CCTTGACTAGCTCAGGgggTCGCACTCAATGCAGCCGTCACATTTCTCACAATGTGAACTCA
TTGGGAGCAGAGGCTGTCAGCTCACGCTGTCAGTGCTTAGTCACTTAAACTTTCTTTGCATC
TGAAATCAAGCAGGATCACCCGCTGAACTTAA
Uncultured fungus

>OTU_145
AAAGTGCGATAATTATTGCGACTTGCATTCATAGTGAATCATCGAGTTCTTGAACGCATCTT
GCGCCTAGTAGTCAATCTACTAGGCACAGTTGTTTCAGTATCTGCATCCACCAATCAATACA
ACTTGCTTGTGTTGGAACTGGGCTTACTTTTGATGGCATTTGGTTGCTGTCATGGCCTTAAA
TGTATTTAGTCCTAGGTGTTAACTTGTTAATGCCGGATGGAGACTCTAGAGTGCCTTAGAAG
CAGCTTGGTTAGTGAGTTCATAATTCCAAGTGTTAGTCTTTTATTGAACTGGGTTTCTAGTC
TATGGGACTTACTTGAGAGTCGACCTctctAGTAAATCAAACTCACATCTAGATCTGAAATC
AACTGAGATCACCCGCTGAACTTAA
k__Fungi;p__Zygomycota;c__Incertae_sedis;o__Mucorales;f__Licht
heimiaceae;g__Lichtheimia;s__Lichtheimia_sp_CNRMA/F_05_100
1.000

>OTU_146
GAAATGCGATACGTAATGTGAATTGCAGAATTCAGTGAATCATCGAGTCTTTGAACGCACAT
TGCGCCcccTGGTATTCCGGggggCATGCCTGTCCGAGCGTCATTGCTGCCCTCAAGCACGG
CTTGtgtgtTGGGCCcccGTCCcccTCTCCGGggggACGGGCCCGAAAGGCAGCGGCGGCAC
CGCGTCCGATCCTCGAGCGTATGGGGCTTTGTCACCCGCTCTGTAGGCCCGGCCGGCGCTTG
CCGAACGCAAATCAATCTTtttCCAGGTTGACCTCGGATCAGGTAGGGATACCCGCTGAACT
TAA
k__Fungi;p__Ascomycota;c__Eurotiomycetes;o__Eurotiales;f__Aspe
rgillaceae;g__ Penicillium;s__Penicillium_infrapurpureum 0.970

>OTU_147
AAACTGCGATAAGCAATGCGAACCGCAGTGTCTCGTGAGTCATCCGGTATTCGAACGCACAT
GGCAGGTCCTCGGACCTACGTCTGGTTGAGGGTCATATGGACTACTCATGGAGTGATGTGGC
ACGGCGCTTGCCGTGCTCAAAGGCATGGCGCAGGGCTGCTTGGTGGACAATCAGGTGGCCAT
GCACTGCCCGCAGTAGCGTGGGCATTGATGCTTGACCTCAACTCAGACGAGACGACCCGCTG
AACTTAA
Uncultured fungus

>OTU_148
GAAATGCGATAAGTAATGTGAATTGCAGAATTCAGTGAATCATCGAATCTTTGAACGCACAT
TGCGCCCCTTGGTATTCCGAGGGGCATGCCTATTCGAGCGTCATTATCACCCCTCAAGCCTA
GCTTGGTGTTGAGACCTGCTGTCAAGGCAGTCTCTAAAATCAGTGGCAGTGCTGTCAGGCTC
TAAGCGTAGTAAATTCATCGCTATAGACACCTGGTGGCCACTCGCCAGAACCccccccATTtt
ttAATGATTGACCTCGGATTAGGTAGGGATACCCGCTGAACTTAA

Uncultured fungus

>OTU_149
GAAATGCGATAAGTAATGCGAATTGCAGAATTTCCGTGAGTCATCGAATCTTTGAACGCACA
TTGCGCCCATTGGTATTCCGATGGGCATGCCTGTTCGAGCGTCATTATCCTCCCTCAAACCT
CGCGTTTGGTGTTGGACCGCGTCGGTTCTCCGTGACCGACGGGTCTCAAAGTCAATGACGGC
GTCCGTGGGACCCTCGGTGCAACGAGCTTTTCGGAGCACGCGTCGAGTCGAAAGGACCCTCC
GGGCCGGTCAGACCTTTCCATTttttATCAGGTTGACCTCGGATCAGGTAGGAATACCCGCT
GAACTTAA
k__Fungi;p__Ascomycota;c__Eurotiomycetes;o__Chaetothyriales;f_
_Herpotrichiellaceae;g__Penicillium;s__ Cladophialophora sp.
KO-groupP 2014 0.950

>OTU_150
AAAGTGCGATAACTAGTGTGAATTGCAGAATTCAGTGAATCATCGAATCTTTGAACGCACAT
TGCGCCCGCCAGTATTCTGGCGGGCATGCCTGTTCGAGCGTCATTACAACCCTCAGGCCTCC
GGGCCTGGCGTTGGGGATCGGCGGAGCCccccGTGGGCACACGCCGTCCcccAAATACAGTG
GCGGTCCCGCCGCAGCTTCCATTGCGTAGTAGCTAACACCTCGCAACTGGAGAGCGGCGCGG
CCACGCCGTAAAACACCCAACTTCTGAATGTTGACCTCGAATCAGGTAGGAATACCCGCTGA
ACTTAA
k__Fungi;p__Ascomycota;c__Sordariomycetes;o__Hypocreales;f__Ne
ctriaceae;g__Neocosmospora;s__Neocosmospora_vasinfecta0.990

>OTU_151
AAATCGCGATATGTAATGTGATCTGCCTATAGTGAATCATCAAATCTTTGAACGCATCTTGC
ACCTTATGGTATTCCATAAGGTACGTCTGTTTCAGTACCACTAATAAATCtctctctATCCT
TGATGATAGAAaaaaaaGAGATAAATTATTACTGGTCCTGGTGATTCTTTCTCTTTTGAAaa
aaTCACTCTCGGCCTAAATATAAGGCTCGACTTttttttACCAGATCTTGCATCTAGTAAaa
aCCTAGTCGGCTTTAATAGATTtttATTTTCTATTAAGTTTATAGCCATTCTTATATTtttt
AAAATCTTGGCCTGAAATCAGATGGGACTACCCGCTGAACTTAA
k__Fungi;p__Zygomycota;c__Incertae_sedis;o__Mucorales;f__Cunni
nghamellaceae;g__Cunninghamella;s__Cunninghamella_polymorpha
0.990

>OTU_152
GAAATGCGATAACTAATGTGAATTGCAGAATTCAGTGAATCATCGAGTCTTTGAACGCACAT
TGCGCCcccTGGTATTCCGGggggCATGCCTGTCCGAGCGTCATTGCTGCCCTCAAGCCCGG
CTTGtgtgtTGGGCCCCGCCccccGGCTCCGGggggCGGGCCCGAAAGGCAGCGGCGGCACC
GCGTCCGGTCCTCGAGCGTATGGGGCTTCGTCACCCGCTCCGTAGGCCCGGCCGGCGCCCGC
CGGCGACCccccTCAATCTTTCTCAGGTTGACCTCGGATCAGGTAGGGATACCCGCTGAACT
TAA
k__Fungi;p__Ascomycota;c__Eurotiomycetes;o__Eurotiales;f__Tric
hocomaceae;g__Penicillium ;s__Penicillium_janthinellum 0.990

>OTU_153
GAAATGCGATAAGTAATGTGAATTGCAGAATTCCGTGAATCATCGAATCTTTGAACGCACAT
TGCGCCCTCTGGTATTCCGGggggCATGCCTGTCCGAGCGTCATTGCAACCCCTTCAAGCCC
GGCTTGtgtgtTGGGCGTCGTCCCCGCTGGACGCGCCCGAAAGGCAGTGGCGGCTCCGTGTC
CGGTGCCCGAGCGTATGGGCTTCAATCACCCGCTCTGGTGGCCCGGCCGGCGCTGGCCTTCT
CAGTCTTGACCAACTTTTGGTTGAGGCCTTGTGAACTTTCGTGGTTGACCTCGGATCAGGTA
GGGATACCCGCTGAACTTAA
Uncultured soil fungus

>OTU_154
GAAATGCGATAAGTAATGTGAATTGCAGAATTCAGTGAATCATCGAATCTTTGAACGCACCT
TGCGCTCCTTGGTATTCCGAGGAGCATGCCTGTTTGAGTGTCATGAAATCTTCAACCTACAA
GCTTTTGTGGTTTGTAGGCTTGGACTTGGAGGCTTGTCGGCCGTTATCGGTCGGCTCCTCTT
AAATGCATTAGCTTGGTTCCTTGCGGATCGGCTCTCGGTGTGATAATGTCTACGCCGCGACC
GTGAAGCGTTTGGCGAGCTTCTAACCGTCTTATAAGACAGCTTTATGACCTCTGACCTCAAA
TCAGGTAGGACTACCCGCTGAACTTAA
k__Fungi;p__Basidiomycota;c__Agaricomycetes;o__Polyporales;f__
Ganodermataceae;g__Ganoderma;s__Ganoderma_lucidum 1.000

>OTU_155
GAAATGCGATAAGTAATGTGAATTGCAGAATTCAGTGAATCATCGAATCTTTGAACGCACAT
TGCGCCCCTTGGTATTCCGAGGGGCATGCCTGTTCGAGCGTCATTACACCACTCAAGCTATG
CTTGGTATTGGGCGTCGTCCTTAGTTGGGCgcgcCTTAAAGACCTCGGCGAGGCCACTCCGG
CTTTAGGCGTAGTAGAATTTATTCGAACGTCTGTCAAAGGAGAGGAACTCTGCCGACTGAAA
CCTTTATTtttCTAGGTTGACCTCGGATCAGGTAGGGATACCCGCTGAACTTAA
k__Fungi;p__Ascomycota;c__Dothideomycetes;o__Dothideales;f__Au
reobasidiaceae;g__Aureobasidium;s__Aureobasidium_pullulans
1.000

>OTU_156
GAAATGCGATAAGTAATGTGAATTGCAGATACAGTGAATCATCGAATCTTTGAACGCATATT
GCACCTTTTGGTATTCCATAAGGTACGTCTGTTTGAGCGTCGCGAACATCTCATAATTAATA
AATTtttttGATTGTTAATTATGGTCTTTGAGTTTGTCTATAATTATAGACTAACTTTAAAT
TGATTAGTAGTTTAACTTTTGAAAGGGTTAAAATTAGGTGTTtttAATGTACATTACTTGTG
CATCATCTAATCAAGAGTTTTAAATTCTGCCTTAGTATTAATGTTACTGCTTCTAATAGCTT
TAATTAAGCAATAAAGATTTCAATCGACCTCAAATCAGATGGGATTACCCGCTGAACTTAA
Uncultured fungus

>OTU_157
AAATCGCGATATGTAATGTGATCTGCCTATAGTGAATCATCGAATCTTTGAACGCACATTGC
GCCCGCCAGTATTCTGGCGGGCATGCCTGTTCGAGCGTCATTACAACCCTCAGGCCccccGGG
CCTGGCGTTGGGGATCGGCGGAAGCCcccTGCGGGCACAACGCCGTCCcccAAATACAGTGG
CGGTCCCGCCGCAGCTTCCATTGCGTAGTAGCTAACACCTCGCAACTGGAGAGCGGCGCGGC
CACGCCGTAAAACACCCAACTTCTGAATGTTGACCTCGAATCAGGTAGGAATACCCGCTGAA

CTTAAGCATATCAATAAGCGGAGGAATCCGTAGGTGAACCTGCG
k__Fungi;p__Ascomycota;c__Sordariomycetes;o__Hypocreales;f__Nectriaceae;g__Fusarium;s__Fusarium_solani 0.970

>OTU_158
GAAATGCGATAAGTAGTGTGAATTGCAGAATTCAGCGAATCATCGAATCTTTGAACGCACAT
TGCGCCCTTCGGTTATTCCTTAGGGCATGCCTGTTCGAGCGTCATTTCAACCTTCAAGCCCC
GCTTGGTGTTGGGCGCTGTCCCGCCTCTGCGCGAGGACTCGCCCCAAATGAATTGGCAGTCG
CACCcccGATACGCGAGCGCAGCAAAGTCGcgcgAGCCGAACCGGCAGGGACGGACGCTCCA
CGAGACCCACCACAGTCTTGACCTCGGATCAGGTAGGGATACCCGCTGAACTTAA
k__Fungi;p__Ascomycota;c__unidentified;o__unidentified;f__unidentified;g__unidentified 1.000

>OTU_159
GAAATGCGATAATTAATGTGAATTGCAGAATTCAGTGAATCATCGAGTCTTTGAACGCACAT
TGCGCCcccTGGTATTCCGGggggCATGCCTGTCCGAGCGTCATTGCTGCCCTCAAGCACGG
CTTGtgtgtTGGGTCGCCGTCCCCTTCCCGGgggACGGGCCCGAAAGGCAGCGGCGGCACCG
CGTCCGGTCCTCGAGCGTATGGGGCTTTGTCACCCGCTCTGTAGGCCCGGCCGGCGCCTGCC
GACCACCAACCTTttttttACCAGGTTGACCTCGGATCAGGTAGGGATACCCGCTGAACTTA
A
k__Fungi;p__Ascomycota;c__Eurotiomycetes;o__Eurotiales;f__Trichocomaceae;g__Aspergillus;s__Aspergillus_wentii 1.000

>OTU_160
GAAATGCGATAAGTAATGTGAATTGCAGAATTCAGTGAATCATCGAATCTTTGAACGCACAT
TGCGCCcccTGGTATTCCGGggggCATGCCTGTTCGAGCGTCATTTCACCACTCAAGCCTCG
CTTGGTATTGGGCAACGCGGTCCGCCGCGTGCCTCAAATCGACCGGCTGGGTCTTCTGTCCC
CTAAGCGTTGTGGAAACTATTCGCTAAAGGGTGCTCGGGAGGCTACGCCGTAAAACAAACCC
ATGTCTAAGGTTGACCTCGGATCAGGTAGGGATACCCGCTGAACTTAAGCATATCAATAAGC
GGAGGAGTCCGTAGGTGAACCTGCGGCATCGATGAAGAACGCA
k__Fungi;p__Ascomycota;c__Dothideomycetes;o__Capnodiales;f__Mycosphaerellaceae;g__Cladosporium;s__Cladosporium_sp_RA10-1 0.990

>OTU_161
GAAATGCGATAAGTAGTGTGAATTGCAGAATTCAGTGAATCATCGAATCTTTGAACGCACAT
TGCGCCCCTTGGTATTCCATGGGGCATGCCTGTTCGAGCGTCATCTACACCCTCAAGCTCTG
CTTGGTGTTGGGCGTCTGTCCCGCCTCTGCGCGTGGACTCGCCCCAAATTCATTGGCAGCGG
TCCTTGCCTCCTCTCGCGCAGCACATTGCGCTTCTCGAGGTGCGCGGCCCGCGTCCACGAAG
CAACATTACCGTCTTTGACCTCGGATCAGGTAGGGATACCCGCTGAACTTAAGCATATCAAT
AAGCGGAGGAATCCGTAGGTAACCTGCGGCATCGATGAAGAAC
k__Fungi;p__Ascomycota;c__Dothideomycetes;o__Botryosphaeriales;f__Botryosphaeriaceae;g__Microdiplodia;s__Microdiplodia_hawaiiensis 0.800

>OTU_162
GAAATGCGATACGTAGTGTGAATTGCAGAATTCAGTGAATCATCGAATCTTTGAACGCACAT
TGCGCCCTTTGGTATTCCAAAGGGCATGCCTGTTCGAGCGTCATTTGTACCCTCAAGCTTTG
CTTGGTGTTGGGCGTTGTTTGTCTTTGGTCCGCCCAAAGACTCGCCTTAAAACAATTGGCAG
CCGGCCTACTGGTTTCGCAGCGCAGCACATTtttGCGCTTGCAATCAGCAAAAGAGGTTGGT
CATCCATCAAGACTACATTTATACGTTTGACCTCGGATCAGGTAGGGATACCCGCTGAACTT
AA
k__Fungi;p__Ascomycota;c__Dothideomycetes;o__Pleosporales;f__P
leosporaceae;g__Curvularia;s__Curvularia_lunata 1.000

>OTU_163
GAAATGCGATACGTAATGTGAATTGCAGAATTCAGTGAATCATCGAGTCTTTGAACGCACAT
TGCGCCCTCTGGTATTCCGGggggCATGCCTGTCCGAGCGTCATTGCTGCCCTCAAGCACGG
CTTGtgtgtTGGGCCcccccGTCCCGGTTCTCCAGCCGGGACGGGCCCGAAAGGCAGCGGCG
GCACCGTGTCCGGTCCTCGAGTGTATGGGGCTCTGTCACCCACTCGTGTAGGCCCGGCCGGC
GGCCAGCCTCTTTCAACCAAAACTTCTTTAACCAGGTTGACCTCGGATCAGGTAGGGATACC
CGCTGAACTTAA
k__Fungi;p__Ascomycota;c__Eurotiomycetes;o__Eurotiales;f__Tric
hocomaceae;g__Aspergillus;s__Aspergillus_gracilis 0.990

>OTU_164
GAAATGCGATACTTGGTGTGAATTGCAGAATCCCGTGAACCATCGAGTCTTTGAACGCAAGT
TACGCCCGAAGCCCTTAGGCTGAGGGCACGCCTGCCTAGGTGTCACCAAaaaaaGCCccccG
TCTCGCCCGTCCCAGGGCACGGGGAGGGGCGAACGTTGGCCTCCCGGGAGCCCCTGGCTCGC
GGTTGGTTCAAAGAGACGGGCTCTTGTTGGGGAGCGGCACCGCGGCAGATGGTGGTCGAGAA
CAACCCTCGTGGCCAGCCGcgcgTGCCTctctcCCGGTTCAAGGCACGGCGACCCGCGGCCG
ACGTGGATCGTCCCGAGCGCGACCTCAGGTCAGGCGGGGCTACCCGCTGAGTTTAA
Arachis hypogaea

>OTU_165
AAAATGCGATACTTGGTGTGAATTGCAGAATCCCGTGAATCATCGAGTTtttGAACGCAAGT
TGCGCCCGAAGCCTTTCGGCCGAGGGCACGCCTGCCTGGGTGTCACGCAATTGTCGCCcccA
ACCCTTTCGATACATCGAGAGGggggCGGATTATGGCCTCCCGTGCGCCTCGTGCATGCGGT
TGGCCTAAaaaTTGAGTCCCCGGCGACTATCGCCACGGCAATCGGTGGTTGTAAGACTctct
GAAACTGCCGTGCgcgcTCGTCTGCCAAGAGGGAACCCTCGAGACCCCGATGCTGCCGTAAA
GGGCATGCTCCAACTGCGACCCCAGGTCAGGCGGGATTACCCGCTGAGTTTAA
Ricinus communis

>OTU_166
ACATTTCCAACCTGCGTCGTAAACTGCCGGATCGTAAAGATGGTCACCCGTGGTTTAAAACC
TTGCGTGGTCGCGGCTATCTGATGGTTTCTGCTTCATGATAGGCAGCTTAACCGcgcgcATC
TTCGCCATCTTCTGGCTGACGCTGGCGCTGGTGTTGATGTTGGTTTTGATGTTACCCAAGCT
CGATTCACGCCAGATGACCGAGCTTCTGGATAGCGAACAGCGTCAGGGGCTGATGATTGAGC

A
Escherichia coli

>OTU_167
GAAATGCGATAAGTAATGTGAATTGCAGAATTCAGTGAATCATCGAATCTTTGAACGCACAT
TGCGCCCCTCGGTATTCCGGggggCATGCCTGTTCGAGCGTCATTACACCACTCAAGCCTCG
CTTGGTCTTGGGCGTCCGCGGTCCGCCGcgcgcCCCAATGTCTCCGGCTGAGCCGTCCGTCT
CTAAGCGTTGTGATAAACTGTTCGCTTGCGAGGCCGGGCTGGCTTGTTTGCCGTTAAACCcc
ccATTtttACAGGTTGACCTCGGATCAGGTAGGGATACCCGCTGAACTTAA
k__Fungi;p__Ascomycota;c__Dothideomycetes;o__Capnodiales;f__Te
ratosphaeriaceae;g__Catenulostroma;s__Catenulostroma_protearum
0.990

>OTU_168
GAAATGCGATACGTAATGTGAATTGCAAATTCAGTGAATCATCGAGTCTTTGAACGCACATT
GCGCCccccTGGTATTCCGGggggCATGCCTGTCCGAGCGTCATTTCTGCCCTCAAGCACGGC
TTGtgtgtTGGGCCCCGTCCTCCGATCCCGGgggACGGGCCCGAAAGGCAGCGGCGGCACCG
CGTCCGGTCCTCGAGCGTATGGGGCTTTGTCACCCGCTCTGTAGGCCCGGCCGGCGCTTGCC
GATCAACCCAAATTtttATCCAGGTTGACCTCGGATCAGGTAGGGATACCCGCTGAACTTAA
GCATATCAATAAGCGGAGGA
k__Fungi;p__Ascomycota;c__Eurotiomycetes;o__Eurotiales;f__Tric
hocomaceae;g__Penicillium;s__Penicillium_chrysogenum 1.000

>OTU_169
GAAATGCGATAAGTAATGTGAATTGCAGATACAGTGAATCATCGAATCTTTGAACGCAAATG
GCACTCTATGGTATTCCGTAGAGTACGTCTGTTTGAGCGTCGCGAACATCTCCATAATTAGT
TtttttAAATTGATTGTGGGTTTTGAGGTTGTCATATAACAATGACTCCCTTTAAAATAATT
AGTGATGACCTTATGAATGGTTTAATACTGtgtgtTATAATGGATTACATCCATCACCAGTC
AGagagTAATCTCGCCTTAGTAATTTGTAGTGATTGCTTCTAACTGCCATTGGCAAACAAAC
TGATCAAATCGACCTCAAATCAGATGGGATTACCCGCTGAACTTAAGCATATCAATAAGCGG
AGGAATCGTGGAGTTACCCGCTGAACTTAA
k__Fungi;p__Basidiomycota;c__Wallemiomycetes;o__Wallemiales;f_
_Wallemiaceae;g__Wallemia 1.000

>OTU_170
GAAATGCGATAAGTAATGTGAATTGCAGAATTCAGTGAATCATCGAATCTTTGAACGCACAT
TGCGCCCGCCAGTATTCTGGCGGGCATGCCTGTCTGAGCGTCATTTCAACCCTCATGCCCCT
AGGGCGTGGTGTTGGGGATCGGCCAAAGCCCGCGAGGGACGGCCGGCCCCTAAATCTAGTGG
CGGACCCGTCGTGGCCTCCTCTGCGAAGTAGTGATATTCCGCATCGGAGAGCGATGAGCCCC
TGCCGTTAAACCcccAACTTTCTAAGGTTGACCTCAGATCAGGTAGGAATACCCGCTGAACT
TAAGCATATCAATAAGCGGAGGAGCATATCAATAAGCGGAGGAGCATATCAATAAGCGGAGG
AATCCGTAGGTGAACCTGCGGA
k__Fungi;p__Ascomycota;c__Sordariomycetes;o__Hypocreales;f__Bi
onectriaceae;g__Bionectria;s__Bionectria_ochroleuca 0.970

>OTU_171
GAAATGCGATAAGTAATGTGAATTGCAGAATCTCGTGAATCATTGAATCTTTGAACGCACAT
TGCGCCCTATGGTATTCCGTAGGGCATGCCTGTCTGAGCGTCAGCTCGTCTTCTCAAGCTCT
TTTGCTTGGATAACATTGGAATAGCAATGCAAGCTCCTCTTTGGGGTCTGCGTGCTCTCCAG
AAATAAATAGGCGGTACGGTTCTAGCAAACCAGACGTAATAATATTCTTATCATTCGTTAAT
TTGTTTAAACTGTTGCTTGCCTCTTAATCCACATCTTttttCAAGATGACCTCAGATCAGGT
AGGGATACCCGCTGAACTTAA
k__Fungi;p__Ascomycota;c__Pezizomycetes;o__Pezizales;f__uniden
tified;g__unidentified;s__Pezizales_sp_P10 1.000

>OTU_172
AAAATGCGATAAGTAATGTGAATTGCAGAATTCAGTGAATCATCGAATCTTTGAACGCACAT
TGCGCCCGCTGGTATTCCGGCGGGCATGCCTGTTCGAGCGTCATTTCAACCCTCAAGCCccc
GGGTTTGGTGTTGGGGATCGGCTCTGCCTTCTGGCGGCGCCGCCcccGAAATACATTGGCGG
TCTCGCTGCAGCCTCCATTGCGTAGTAGCTAACACCTCGCAACTGGAACGCGGCGCGGCCAT
GCCGTAAAACCCCAACTTCTGAATGTTGACCTCGGATCAGGTAGGAATACCCGCTGAACTTA
A
k__Fungi;p__Ascomycota;c__Sordariomycetes;o__Hypocreales;f__Ne
ctriaceae;g__Fusarium 1.000

>OTU_173
GAAATGCGATAAGTAATGTGAATTGCAGAATTCAGTGAATCATCGAATCTTTGAACGCACAT
TGCGCCCGCCAGTATTCTGGCGGGCATGCCTGTCCGAGCGTCATTTCAACCCTCGAACCCCT
CCGGggggTCGGCGTTGGGGATCGGGAACCCCTCAGACGGGATCCCGGCCCCGAAATACAGT
GGCGGTCTCGCCGCAGCCTCTCCTGCGCAGTAGTTTGCACAACTCGCACCGGGAGCGCGGCG
CGTCCACGTCCGTAAAACACCCAACTTCTGAAATGTTGACCTCGGATCAGGTAGGAATACCC
GCTGAACTTAA
k__Fungi;p__Ascomycota;c__Sordariomycetes;o__Hypocreales;f__Hy
pocreaceae;g__Trichoderma;s__Trichoderma_erinaceum 1.000

>OTU_174
GAAATGCGATACGTAATATGAATTGCAGATATTCGTGAATCATCGAATCTTTGAACGCACAT
TGCGCCCTCTGGTATTCCGGAGGGCATGCCTGTTTGAGCGTCGTTTCTCCCTCAAACCGCTG
GGTTTGGTGTTGAGCAATACGACTTGGGTTTGCTTGAAAGACGGTAGTGGTAAGGCGGGATC
GCTTTGACAATGGCTTAGGTCTAACCAAaaaCATTGCTTGCGGCGGTAACGTCCACCACGTA
TATCTTCAAACTTTGACCTCAAATCAGGTAGGACTACCCGCTGAACTTAA
k__Fungi;p__Ascomycota;c__Saccharomycetes;o__Saccharomycetales
;f__Incertae_sedis;g__Candida;s__Candida_albicans 1.000

>OTU_175
GAAATGCGATAAGTAATGTGAATTGCAGAATTCAGTGAATCATCGAATCTTTGAACGCACAT
TGCGCCCCTTGGTATTCCGGggggCATGCCTGTTCGAGCGTCATTATAACCCTCAAGCCTAG
CTTGGTGTTGGAGCATGCTACCTAGCAGCTCTTAAAATCAGTGGCAGTGCCCTCTGGCTCTA

AGTGTAGTAACTTCtctcGCTATGGAACCCAGAGGGACCGCGCCAGAACCcccAACTTCTAA
TGATTGACCTCGGATCAGGTAGGGATACCCGCTGAACTTAA
Uncultured fungus

>OTU_176
GAAATGCGATACGTAATGTGAATTGCAGAATTCAGTGAATCATCGAGTCTTTGAACGCACAT
TGCGCCcccTGGTATTCCGGggggCATGCCTGTCCTAGCGTCATTGCTGCCCTCAAGCACGG
CTTGtgtgtTGGGCTTCCGTCCCTGGTAACGGGGACGGGCCCAAAAGGCAGTGGCGGCACCA
TGTCTGGTCCTCGAGCGTATGGGGCTTTGTCACCCGCTCCCGTAGGTCCAGCTGGCAGCTAG
CCTCGCAACCAATCTTtttAACCAGGTTGACCTCGGATCAGGTAGGGATACCCGCTGAACTT
AAGCATATCAATAAGCGGAGGAGTCCGTAGGTGAACCTGCGGGCATATCAATAAGCGGAGGA
k__Fungi;p__Ascomycota;c__Eurotiomycetes;o__Eurotiales;f__Tric
hocomaceae;g__Eurotium;s__Eurotium_niveoglaucum 1.000

>OTU_177
ATCGATGAAGAACGCAGCATCGATGAAGAACGCAGCATCGATGAAGAACGCAGCATCGATGA
AGAACGCAGCATCGATGAAGAACGCAGCATCGATGAAGAACGCAGCATCGATGAAGAACGCA
GCATCGATGAAGAACGCAGCATCGATGAAGAACGCAGCATCGATGAAGAACGCAGCATCGAT
GAAGAACGCAGCATCGATGAAGAACGCAGCATCGATGAAGAACGCAGCATCGATGAAGAAC
k__Fungi;p__Ascomycota 0.970

>OTU_178
GAAACGCGATATGTAATGTGAATTGCAGAATTCAGTGAATCATCGAATCTTTGAACGCACAT
GGCGCCTTCCAGTATCCTGGGAGGCATGCCTGTCCGAGCGTCGTTTCAACCCTCGAGCCccc
GTGGCCCGGCGTTGGGGATCTGCCACGGCAGGCCCCTAAAACCAGTGGCGGACCCGAAGGGC
CCTCTCCTTTGCGCAGTAGCATCAGCCTCGCATCGGGAGCCCTCGGCGTCCTGCCTCTAAAC
CccccACAAGCCCGCCTAGCGGCACCAAGGTTGACCTCGGATCAGGTAGGAATACCCGCTGA
ACTTAA
k__Fungi;p__Ascomycota;c__Sordariomycetes;o__Incertae_sedis;f_
_Plectosphaerellaceae;g__Plectosphaerella;s__Plectosphaerella_
alismatis 0.990

>OTU_179
GAAATGCGATACCTGGTGTGAATTGCAGAATCCCGCGAACCATCGAGTCTTTGAACGCAAGT
TGCGCCCGAGGCCACTCGGCCGAGGGCACGCCTGCCTGGGCGTCACGCCAAAACACGCTCCC
ACACCCCTCATTGGGAATCGGGATGCGGCATCTGGTCCCTCGTCTCGCAAGGGGCGGTGGAC
CGAAGTTCGGGCTGCCGGCGTACCGTGTCAAACACAGCGCGTGGTGGGCGTCTTTGCTTTAT
CAACGCAGTGCATACGACGCGTAGCCGGCATGATGGCCTCAAAACGACCCAACAAACGTAGC
GCACGTCGCTTCGACCGCGACCCCAGGTCAGGCGGGACTACCCGCTGAGTTTAA
k__Fungi;p__Ascomycota;c__Eurotiomycetes;o__Eurotiales;f__Tric
hocomaceae;g__Emericella;s__Emericella_nidulans 1.000

>OTU_180
GAAATGCGATACTTGGTGTGAATTGCAGAATCCCGTGAACCATCGAGTTtttGAACGCAAGT

TGCGCCCGAAGCCATTAGGTTGAGGGCACGTCTGCCTGGGCGTCACGCATCTAGTCGCCACC
cccTCTCGTAATTACGGAGTGGAGTGGCGGATGTTGGCCTCCCGTGTTCCTGAGGCGCGGCT
GGCCTAAATTTGAGTCTTCGACGCGAGATGTCACGGCAAGTGGTGGTTGAAATCATCAACTC
GTGTGCTGTCGCGACCACTCCCGGCGCAGACTCCATCGACCCTAGAGTCCTCGACCCAAATT
TGAGTTGCGGGCCTTCGACTGCGACCCCAGGTCAGGCGGGATTACCCGCTGAGTTTAA
Psychotria asiatica

>OTU_181
GAAATGCGATAACTAGTGTGAATTGCAGAATTCCGTGAATCATCGAGTCTTTGAACGCACAT
TGCGCCcccTGGTATTCCGGggggCATGCCTGTCCGAGCGTCATTGCTGCCCATCAAGCACG
GCTTGtgtgtTGGGTCGTCGTCCCCTCTCCGGgggggACGGGCCCCAAAGGCAGCGGCGGCA
CCGCGTCCGATCCTCGAGCGTATGGGAAGCAACTCTTtttGTCACCCGCTCCTGTAGGTCCG
GCCGGCGCTTGCCGAACGCAAATCAATCTTTTCCAGGTTGACCTCGGATCAGGTAGGGATAC
CCGCTGAACTTAA
k__Fungi;p__Ascomycota;c__Eurotiomycetes;o__Eurotiales;f__Tric
hocomaceae;g__Aspergillus;s__Aspergillus_flavus 0.970

>OTU_182
GAAATGCGATAAGTAGTGTGAATTGCAGAATTCAGTGAATCATCGAATCTTTGAACGCACAT
TGCGCCCCTTGGTATTCCATGGGGCATGCCTGTTCGAGCGTCATTTGTACCCTCAAGCTTTG
CTTGGTGTTGGGTGTTTGTCCTCTCCTTTGCGTTTGGACTCGCCTTAAAGCAATTGGCAGCC
AGTGTTTTGGTATTGAAGCGCAGCACATTTTGCGATTCTAGCCGAAAGACTTGCGTCCATAA
GCCTTttttAACTTTTGACCTCGGATCAGGTAGGGATACCCGCTGAACTTAAGCATATCAAT
AAGCGGAGGAATCCGTAGGTGAACCTGCGGCATCGATGAAGAACGCA
k__Fungi;p__Ascomycota;c__Dothideomycetes 0.990

>OTU_183
GAAATGCGATACGTAGTGTGAATTGCAGAATTCAGTGAATCATCGAATCTTTGAACGCACAT
TGCGCCCTTTGGTATTCCAAAGGGCATGCCTGTTCGAGCGTCATTTGTACCCTCAAGCTTTG
CTTGGTGTTGGGCGTCTTGTCTCCAGTTCGCTGGAGACTCGCCTTAAAGTCATTGGCAGCCG
GCCTACTGGTTTCGGAGCGCAGCACAAGTCGCGCTctctTCCAGCCAAGGTCAGCGTCCAGT
AAGCCTTtttttCAACTTTTGACCTCGGATCAGGTAGGGATACCCGCTGAACTTAA
k__Fungi;p__Ascomycota;c__Dothideomycetes;o__Pleosporales;f__P
leosporaceae;g__Alternaria;s__Embellisia_phragmospora 1.000

>OTU_184
GAAATGCGATACTTGGTGTGAATTGCAGAATCCCGTGAACCATCGAGTCTTTGAACGCAAGT
TGCGCCCGAAGCCATTCGGCCGAGGGCACGTCTGCCTGGGCGTCACGCATCGCGTCGCCCCA
GACCATGCTCCcccATGGGGAAGTGTTTGGTCTGGGACGAAGAGTGGTCTCCCGTGTCGATG
GTGCGGTTGGCCTAAaaaGGAGTCCCCTTTGGCGGACGCACGGCTAGTGGTGGTTGTAAAGG
ACTTCGTAACGAGCCGTGTTGATGCTAGGGAATTGCTctctAAAGACCCTAACGTGTCGTCT
TACGACGATGCTTCGACCGCGACCCCAGGTCAGGCGGGACTACCCGCTGAGTTTAA
Carthamus glaucus

>OTU_185
GAAATGCGATAAGTAGTGTGAATTGCAGAATTCAGTGAATCATCGAATCTTTGAACGCACAT
TGCGCCCCTTGGTATTCCATGGGGCATGCCTGTTCGAGCGTCATTTGTACCTTCAAGCTTTG
CTTGGTGTTGGGTGTTTGTCCTGAGGGACTCGCCTTAAAGTAATTGGCAGCCAGTGTTTGGT
TTTGAAGCGCAGCACAAGTCGCGATTCAAGGCTATACGCCAGCTTCCACAAGCCTTtttCAC
TTTTGACCTCGGATCAGGTAGGGATACCCGCTGAACTTAA
k__Fungi;p__Ascomycota;c__unidentified;o__unidentified;f__unid
entified;g__unidentified;s__Ascomycota_sp_MG106 0.930

>OTU_186
GAAATGCGATACCTAGTGTGAATTGCAGCCATCGTGAATCATCGAGTTCTTGAACGCACATT
GCGCCCCTTGGTATTCCAGGgggCATGCCTGTTTGAGCGTCGTTTCCTTCTTGCGCAAGCAG
AGTTGGCGGgggCAGCGATGCCGCCGCTGAAAAGGAGCGATTGCGGACGCGAGCGAACTAAA
GTGGTTGCGCTTGGCCGCCGAACTTATACACTAAGCTCGACCTCAAATCAGGTAGGAATACC
CGCTGAACTTAA
k__Fungi;p__Ascomycota;c__Saccharomycetes;o__Saccharomycetales
;f__Pichiaceae;g__Pichia;s__Pichia_exigua 1.000

>OTU_187
GAAATGCGATACGTAATGTGAATTGCAGAATTCAGTGAATCATCGAGTCTTTGAACGCACAT
TGCGCCcccTGGTATTCCGGggggCATGCCTGTCCGAGCGTCATTGCTGCCCTCAAGCACGG
CTTGtgtgtTGGGCCcccGTCCCCTCCCTAGGGAGGGGACGGGCCCGAAAGGCAGCGGCGGC
ACCGTGTCCGGTCCTCGAGCGTATGGGAAGCACAATCTTtttAACCAGGTTGACCTCGGATC
AGGTAGGGATACCCGCTGAACTTAA
k__Fungi;p__Ascomycota;c__Eurotiomycetes;o__Eurotiales;f__Tric
hocomaceae;g__Aspergillus;s__Aspergillus_penicillioides 0.990

>OTU_188
CAAATGCGATAAGTAATGTGAATTGCAGAGTTCCGTGAATCATCGAATCTTTGAACGCACAT
TGCGCCCGCCAGTATTCTGGCGGGCATGCCTGTTCGAGCGTCATTTCAACCCTCAAGCACAG
CTTGGTGTTGGGACTCGCGTTAATTCGCGTTCCCCAAATTGATTGGCGGTCACGTCGAGCTT
CCATAGCGTAGTAGTAAAACCCTCGTTACTGGTAATCGTCGCGGCCACGCCGTTAAACCCCA
ACTTCTGAATGTTGACCTCGGATCAGGTAGGAATACCCGCTGAACTTAAGCATATCAATAAG
CGGAGGAGTATATCAATAAGCGGAGGG
k__Fungi;p__Ascomycota;c__Sordariomycetes;o__Hypocreales;f__Ne
ctriaceae;g__Fusarium;s__Fusarium_oxysporum 0.980

>OTU_189
GAAATGCGATAAGTAGTGTGAATTGCAGAATTCAGTGAATCATCGAATCTTTGAACGCACAT
TGCGCCTCCTGGTATTCCGGGAGGCATGCCTGTTCGAGCGTCATTAAAATCAACTCAAGCTC
TTTTGCTTGGTCATGGAAGAAGAGTCTGCATCGCGGTCTCCCCTCCGAAATCCAATGGCGGA
AGGTCACTGGTACCCGGCGTAGTATTAACGTTCGCTGTGTATCCAGAGCCATTCCCGCCCAA
CAACCcccTTTCTCGTGATTGACCTCGGATCAGGTAGGGATACCCGCTGAACTTAA
k__Fungi;p__Ascomycota;c__Pezizomycetes;o__Pezizales;f__Peziza

ceae;g__unidentified;s__Pezizaceae_sp_C14 1.000

>OTU_190
GAAATGCGATAAGTAGTGTGAATTGCAGAATTCAGTGAATCATCGAATCTTTGAACGCACAT
TGCGCCCCTTGGTATTCCATGGGGCATGCCTGTTCGAGCGTCATTTACACCCTCAAGCTCTG
CTTGGTGTTGGGCGTCTGTCCCGCTTCGTGCGCGGACTCGCCCTAAAGGTATTGGCAGCGGT
CATGCCAGCTTCTCGCGCAGCACATTGCGCTTCTCGAGGCACCGGCGGATCAGCGTCCATCA
AGCCCACTTCCACAGTTTGACCTCGGATCAGGTAGGGATACCCGCTGAACTTAA
k__Fungi;p__Ascomycota;c__Dothideomycetes;o__Pleosporales;f__u
nidentified;g__unidentified;s__Uncultured Pleosporales1.000

>OTU_191
GAAATGCGATACGTAATGTGAATTGCAGAATTCAGTGAATCATCGAGTCTTTGAACGCACAT
TGCGCCcccTGGTATTCCGGggggCATGCCTGTCCGAGCGTCATTGCTGCCCTCAAGCACGG
CTTGtgtgtTGGGCCcccGTCCccccTGCTTTCTAGGgggggACGGGCCCGAAAGGCAGCGG
CGGCACCGGGTCCGGTCCTCGAGCGTATGGGAAGCAACTTtttttGTCACCCGCTCCTGTA
GGTCCGGCCGGCGGCCTGCCcccAACCCTCAATCAATCTTTTAACCAGGTTGACCTCGGATC
AGGTAGGGATACCCGCTGAACTTAA
k__Fungi;p__Ascomycota;c__Eurotiomycetes;o__Eurotiales;f__Tric
hocomaceae;g__Aspergillus;s__Aspergillus_penicillioides 0.990

>OTU_192
GAAATGCGATACGTAATGTGAATTGCAGAATTCAGTGAATCATCGAGTCTTTGAACGCACAT
TGCGCCcccTGGTATTCCGGggggCATGCCTGTCCGAGCGTCATTGCTGCCCTCAAGCACGG
CTTGTTGTTGGGTCTACGTCCccccGGGGACGGGCCCGAAAGGCAGCGGCGGCATCGCGTCC
GGTCCTCGAGCGTATGGGGCTTCGTCACCCGCTCTTGTAGGCCCGGCCGGCGCCAGCCGACC
CCAACCATTtttCTCAGGTTGACCTCGGATCAGGTAGGGATACCCGCTGAACTTAA
k__Fungi;p__Ascomycota;c__Eurotiomycetes;o__Eurotiales;f__Tric
hocomaceae;g__Penicillium;s__Penicillium_cinnamopurpureum0.990

>OTU_193
GAAATGCGATAAGTAATGTGAATTGCAGAATTCCGTGAACCATCGAGTCTTTGAACGCAAGT
TGCGCCCGAAGCCCTTAGGCTGAGGGCACGCCTGCCTGGGTGTCACCAAAAGGCGCCccccG
TCTCGCCCGTCCCAGGGCACGGGGAGGgggCGAACGTTGGCCTCCCGGGAGCCCCTGGCTCG
CGGTTGGTTCAAAGAGACGGGCTCTTGGTGGGGAGCGGCACCGCGGCAGATGGTGGTCGAGA
ACAACCCTCGTGGCCAGTCGcgcgcgcCTCTCCcccGGTTCAAGGCACGGCGACCCGCGGGC
GACGTGGATCGTCCCGAGCGCGACCTCAGGTCAGGCGGGGCTACCCGCTGAGTTTAAGCATA
TCAATAAGCGGAGGATGTTGGACTTAA
Arachis hypogaea

>OTU_194
GAAATGCGATAAGTAGTGTGAATTGCAGAATTCAGTGAATCATCGAATCTTTGAACGCACAT
TGCGCCCTTTGGTATTCCTTAGGGCATGCCTGTTCGAGCGTCATTTAACCcccTCAAGCTCT
GCTTGGTGTTGGGCGTTTGTCCCGCCTCGTGCGCGGACTCGCCTTAAAATCATTGGCGGCCT

TTGGATTCGGCTCGAGCGCAGCACATCGCGTCTTCGGTCCGACCCGCTGGCGTCCAGCAAGC
GCAACTTTATTTTGACCTCGGATCAGGTAGGGATACCCGCTGAACTTAA
k__Fungi;p__Ascomycota 0.900

>OTU_195
GAAATGCGATAAGTAATGTGAATTGCAGAATTCAGTGAATCATCGAATCTTTGAACGCACCT
TGCGCCCTCTGGTATTCCGGAGGGCATGCCTGTTTGAGTGTCATGTAGACTCAACCccccTG
GTTTATGATCGGGAAGGGTTGGATGTGGGCGCTGCCAATTGCTGGCTCGCCTTAAATGTCTT
AGCGGCTTAGAAGCCCCGACCTAGCGTAATAAGTTTCGCTGGAGAGGGTGTGGATGACTGCT
TACAATCGCCCTCGGGCAATCTTTTGACTCTGGCCTCAAATCAGGTAGGACTACCCGCTGAA
CTTAA
k__Fungi;p__Basidiomycota;c__Tremellomycetes;o__Filobasidiales
;f__Filobasidiaceae;g__Cryptococcus;s__Cryptococcus_flavus
1.000

>OTU_196
GAAATGCGATACTTGGTGTGAATTGCAGAATCCCGTGAACCATCGAGTCTTTGAACGCAAGT
TGCGCCCCAAGCCTTCTGGCCGAGGGCACGTCTGCCTGGGTGTCACAAATCGTCGTTCCccc
TCATCCTTCAAGGATTCGGGACGGAAGCTGATCTCCCGTgtgtTACCGCACGCGGTTGATCG
AAATCCGAGCCAAGGATGCCTTGAGCGTCCCGACATGCGGTGGTGAACTCGTTCAACTCTCC
CTATCGTCGGTCGCTCTTGTCCGGAAGCTCTAGATGACCCAAAGTCTTCAATGCGACCCCAG
GTCAGGCGGGATCACCCGCTGAGTTTAA
Capsella bursa-pastoris

>OTU_197
GAAATGCGATAAGTAGTGTGAATTGCAGAATTCAGTGAATCATCGAATCTTTGAACGCACAT
TGCGCCCCTTGGTATTCCATGGGGCATGCCTGTTCGAGCGTCATTTGAACCCTCAAGCTCTG
CTTGGTGTTGGGCGTTTGTCCCGCGTTTGCGCGTGGACTCGCCTTAAAGCAATTGGCAGCCA
TGTAATTCGGCTTTGAGCGCAGCACAATGCGTACTCTCCGCTGGTACATTGGCATCCAGAAG
CCCTTtttttACTCTTGACCTCGGATCAGGTAGGGATACCCGCTGAACTTAA
k__Fungi;p__Ascomycota;c__Dothideomycetes;o__Pleosporales;f__M
orosphaeriaceae;g__Acrocalymma;s__Acrocalymma_ficus 0.990

>OTU_198
GAAATGCGATAAGTAATGTGAATTGCAGAATCTCGTGAATCATTGAATCTTTGAACGCACAT
TGCGCCCTATGGTATTCCGTAGGGCATGCCTGTCTGAGCGTCAGCTCGTCTTCTCAAGCTCT
TTTGCTTGGATAACATTGGAATAGCAATACAAGCTCTTTTGAGTCTGtgtgCTCTCCAGAAA
TatataGGCGGTACGGTTCTAGCAAACCAGACGTAATAATATTCTTATCTATCGTTAATTTG
TTGAAGCTGTTGCTTGCCTTTTAATCCACATCTTTCTTCAAGATGACCTCAGATCAGGTAGG
GATACCCGCTGAACTTAA
k__Fungi;p__Ascomycota;c__Pezizomycetes;o__Pezizales;f__uniden
tified;g__unidentified;s__Pezizales_sp_P10 1.000

>OTU_199

GAAATGCGATAAGTAATGTGAATTGCAGAATTCCGTGAATCATCGAATCTTTGAACGCACAT
TGCGCCccccTGGCATTCCGGggggCATGCCTGTCCGAGCGTCATTTCTGCCCTCAAGCACGG
CTTGtgtgtTGGGCCcccGTCCcccTCTTttttAGGgggggggggACGGGCCCGAAAGGCAGC
GGCGGCACCGTGTCCGGTCCTCGAGCGTATGGGGCTTTGTCACCCGCTCCCGTAGGTCCAGC
TGGCAGCTAGCCTCGCAACCAATCTTtttAACCAGGTTGACCTCGGATCAGGTAGGGATACC
CGCTGAACTTAA
k__Fungi;p__Ascomycota;c__Eurotiomycetes;o__Eurotiales;f__Tric
hocomaceae 0.980

>OTU_200
GAAATGCGATAATTAATGTGAATTGCAGAATTCAGTGAATCATCGAGTCTTTGAACGCACAT
TGCGCCcccTGGTATTCCGGggggCATGCCTGTCCGAGCGTCATTGCTGCCCATCAAGCACG
GCTTGtgtgtTGGGTCGTCGTCCCCTCTCCGGgggggACGGGCCCAAAAGGCAGTGGCGGCA
CCATGTCTGGTCCTCGAGCGTATGGGGCTTTGTCACCCGCTCTGTAGGCCCGGCCGGCGCTT
GCCGAACGCAAATCAATCTTtttAACCAGGTTGACCTCGGATCAGGTAGGGATACCCGCTGA
ACTTAA
k__Fungi;p__Ascomycota;c__Eurotiomycetes;o__Eurotiales;f__Tric
hocomaceae;g__Aspergillus 1.000

>OTU_201
AAACTGCGATAAGTAGCGTGAATTGCAGACGCTTTGAACGTTAAACTTTCGAACGCACATTG
CGCCGTAGGAGTCCTACCCTGCGGCACATCTGGTTGAGGGTCGTGATGAAaaaCAGCAGGAC
TGTGGCTGTAATAGAAATAGCTGGTGAATCACGAGTTCGGCCTACCGCCTCTGGTCGAACGT
GTTTTGCCATAATGCTAGAAGAGGACAATCACTCTGCTCGAGTGATTGCCTACCACACGGAT
GATCTATTAGCTGAAGCAATAGGTCTCGGTCACCGGGTATCATGCCTAtataCTCGGTGGTG
TCTGAGATACAGCTAGTGGGCCTCATACGTGCCTGGTATTGAGAGTATTCGCTCTCCCGACC
TCAACTCAGGTGTGATTACCCGCTGAACTTAA
Pratylenchus goodeyi

>OTU_202
GAAATGCGATAAGTAGTGTGAATTGCAGAATTCAGTGAATCATCGAATCTTTGAACGCACAT
TGCGCCCTTTGGTATTCCTTAGGGCATGCCTGTTCGAGCGTCATCTAAACCTTCAAGCACTG
CTTGGTGTTGGGTGCCTGTCCCGCCcccGcgcgTGGACTCACCTCAAATCCATTGGCGGCCC
TCACGTCGGCTTCGAGCGCAGCAGAAACGCGAACTCGTGGCCCGGCGGAGCGGCTCCCAGAA
GCTACTCTCACCATTTTGACCTCGGATCAGGTAGGGATACCCGCTGAACTTAAGCATATCAA
TAAGCGGAGGAATCCGTAGGTGAACCTGCGGCATCGATGAAGAACGCA
k__Fungi;p__Ascomycota;c__Dothideomycetes;o__Pleosporales;f__S
porormiaceae;g__Preussia 0.920

>OTU_203
GAAATGCGATAAGTAGTGTGAATTGCAGAATTCAGTGAATCATCGAATCTTTGAACGCACAT
TGCGCCCCTTGGTATTCCATGGGGCATGCCTGTTCGAGCGTCATTTGTACCCTCAAGCTCTG
CTTGGTGTTGGGTGTTTGTCCCGCTTTACGCGTGGACTCGCCTTAAAGCAATTGGCAGCCGG
CAATCTGGTTATAGAGCGCAGCACATTTTGCGCTTCTTGCCATGGATGTCGGCGTCCATCAA

GTACATTtttttGCTCTTGACCTCGGATCAGGTAGGGATACCCGCTGAACTTAA
k__Fungi;p__Ascomycota;c__Dothideomycetes;o__Pleosporales;f__P
leosporaceae;g__unidentified;s__Pyrenochaeta_sp 1.000

>OTU_204
GAAATGCGATAAGTAATGTGAATTGCAGATACAGTGAATCATCGAATCTTTGAACGCAAATG
GCACTCTATGGTATTCCGTAGAGTACGTCTGTTTGAGCGTCGCGAACATCTCCATAATTAGT
TttttCTTAAATTGATTGTGGGTTTTGAGGTTGTCATATAAACAGTGACTCCCTTTAAAATA
ATTAGTGATGACCTTATGAATGGGTTAATACTGtgtgtTATAATGGATTATATCCATCACCA
GTCAGagagTAATCTCGCCTTAGTAATTTGTAGTGATTGCTTCTAACTGCCAATTGGCAACA
ACCTGATCAAATCGACCTCAAATCAGATGGGATTACCCGCTGAACTTAA
k__Fungi;p__Basidiomycota;c__Wallemiomycetes;o__Wallemiales;f_
_Wallemiaceae;g__Wallemia;s__Wallemia_sp_F53 1.000

>OTU_205
AAAGTGCGATAACTAGTGTGAATTGCATATTCAGTGAATCATCGAGTCTTTGAACGCAGCTT
GCACTCTATGGTTtttCTATAGAGTACGCCTGCTTCAGTATCATCACAAACCCAcacaTAAC
ATTTGTTTATGTGGTAATGGGTCGCATCGCTGTTTTATTAAAGTGAGCACCTAAAATGtgtg
tgATTTTCTGTCTGGCTTGCTAGGCAGGAATATTACGCTGGTCTCAGGATCTTtttCTTTGG
TTCGCCCAGGAAGTAAAGTACAAGAGTATAATCCAGCAACTTTCAAACTATGATCTGAAGTC
AGGTGGGATTACCCGCTGAACTTAAGCATATCAATAAGCGGAGGATCTGAGGGGATCCGGAC
AGTCTGCCGGTGGAACATGGTACATACCGCTATGCCAGC
k__Fungi;p__Zygomycota;c__Incertae_sedis;o__Mucorales;f__Mucor
aceae;g__Rhizopus;s__Rhizopus_oryzae 1.000

>OTU_206
GAAATGCGATAAGTAGTGTGAATTGCAGAATTCAGCGAATCATCGAATCTTTGAACGCACAT
TGCGCCCTTCGGTTATTCCTTAGGGCATGCCTGTTCGAGCGTCATTTCAACCTTCAAGCCTG
GCTTGGTGTTGGGCGCTGTCCCGCCTCCGcgcgcgGACTCGCCCCAAATGAATTGGCAGTCG
CACCCTCCGAGCCGCGAGCGCAGCACAAGTCGcgcgGGCGGAACTCTGGggggACGGACGCT
CCACAAGACCCTTTCTCAGTCTTGACCTCGGATCAGGTAGGGATACCCGCTGAACTTAA
k__Fungi;p__Ascomycota;c__unidentified;o__unidentified;f__unid
entified;g__unidentified;s__ascomycete_sp_IBWF79B_90A 1.000

>OTU_207
GAAATGCGATACGTAATGTGAATTGCAGAATTCAGTGAATCATCGAATCTTTGAACGCACAT
TGCGCCCTGTGGTATTCCGCAGGGCATGCCTGTTCGAGCGTCATTTAACCACTCACGCCTAG
CGTGGTATTGGGGCACGCGGTCTTGCGGCCCTCAAAATTAGTGGCGGCGCCGGTGGGCTCTA
AGCGTAGTACATACTCCCGCTATAGAGTTCCccccGGTGGCTCGCCAGAACCccccAATTtttt
ACAGGTTGACCTCGGATCAGGTAGGGATACCCGCTGAACTTAA
k__Fungi;p__Ascomycota;c__Dothideomycetes;o__Pleosporales;f__P
leosporales incertaesedis;g__Latorua;s__Torula_caligans 1.000

>OTU_208

GAAATGCGATAAGTAATGTGAATTGCAGATACAGTGAATCATCGAATCTTTGAACGCATATT
GCACCTTTTGGTATTCCATAAGGTACGTCTGTTTGAGCGTCGCGAACATCTCATAATTAATG
AATTtttttttGTTAATTGTGGTCTTTGAGTTTGTCTAATAATAATTAGACTCACTTTAAAT
TGATTAGTAGTTTAACTTTTGAAAGGGTTAAAATTAGGTGTTttttAATGTACATTAGTTGT
GCATCATCTAATCAAGAGTTACTTACTCTGCCTTAGTATTAATGTTACTGCTTCTAATGGCT
ATAATTTATAGCAATTAATATTTCAATCGACCTCAAATCAGATGGGATTACCCGCTGAACTT
AA
Uncultured fungus

>OTU_209
GAAATGCGATACGTAATGTGAATTGCAGAATCCCGTGAACCATCGAGTCTTTGAACGCAAGT
TGCGCCCCAAGCCTTCTGGCCGAGGGCACGTCTGCCTGGGTGTCACAAATCGTCGTCCcccc
ATCCTCTCGAGGATATCGGACGGAAGCTGGTCTCCCGTgtgtTACCGCACGCGGTTGGCCAA
AATCCTAGCTAAGGATGCCAGGAGCGTCTTGACATGCGGTGGTGAATTCAATTCTCGTCAAA
TCGTCAGTCGTTTCGGTCCGAAAGCTCTTGATGACCCAAAGTCCTCAACGCGACCCCAGGTC
AAGCAGGATCACCCGCTGAGTTTAA
Brassica rapa

>OTU_210
CAGCTGCGTGAATTAATGTGAATTGCAGGACACATTGAACATCGATATCTTGAACGCATATT
GCGGCCTCGGGTAATCCCGAGGCCACGCCTGTCTCAGGGTCGGTTAAACGTCAATCGCGAGT
TGTTCTCCTCTCGCGCTCTGGACGTCGCAGTTTGCCCTGTGCGACTGCGTCGTCTCAAGTTC
AGACGGGTTGCTCGGCCGACGACGCCGGGACGATGACTCGACTCTCGAAGAGCCCGTCtctc
GGCCGCCGAATCGGGCGACTCAAGGCACGAAATACTAAaaaCGACTTCGACCTGAGATCAGA
CGAGATTACCCGCTGAACTTAA
Mesenchytraeus armatus

>OTU_211
GAAATGCGATAACTAATGTGAATTGCAGAATTCAGTGAATCATCGAGTCTTTGAACGCACAT
TGCGCCcccTGGTATTCCGGggggCATGCCTGTCCGAGCGTCATTACTGCCCTCAAGCCCGG
CTTGTATTGGGTCCTCGTCCccccGGGGACGGGCCCGAAAGGCAGCGGCGGCACCGCGTCCG
GTCCTCGAGCGTATGGGGCTTTGTCACCCGCTCTGTAGGCCCGGCCGGCGCCAGCCCACGCA
TAACCTTTATTttttCAGGTTGACCTCGGATCAGGTAGGGATACCCGCTGAACTTAA
k__Fungi;p__Ascomycota;c__Eurotiomycetes;o__Eurotiales;f__Tric
hocomaceae;g__Aspergillus;s__Aspergillus_flavipes 1.000

>OTU_212
GAAATGCGATAATTAATGTGAATTGCAGAATTCAGTGAATCATCGAGTCTTTGAACGCACAT
TGCGCCcccTGGTATTCCGGggggCATGCCTGTCCGAGCGTCATTGCTGCCCTCAAGCACGG
CTTGtgtgtTGGGCTTCCGTCCCTGGTAACGGGGACGGGCCCAAAAGGCAGTGGCGGCACCA
TGTCTGGTCCTCGAGCGTATGGGGCTTTGTCACCCGCTCCCGTAGGTCCAGCCTTtttAACC
AGGTTGACCTCGGATCAGGTAGGGATACCCGCTGAACTTAA
k__Fungi;p__Ascomycota;c__Leotiomycetes;o__Helotiales;f__Scler
otiniaceae;g__Botryotinia;s__Botryotinia_fuckeliana   1.000

>OTU_213
GAAATGCGATAAGTAGTGTGAATTGCAGAATTCAGTGAATCATCGAATCTTTGAACGCACAT
TGCGCCCCTTGGTATTCCGGggggCATGCCTGTTCGAGCGTCATTATAACCCTCAAGCCTAG
CTTGGTGTTGGAGCATGCTACCTAAGCAGCTCTTAAAATCAGTGGCAGTGCCCTTCGGCTCT
AAGCGTAGTAACTTCtctcGCTATGGAACCCGGAGGGTACGCGCCAGAACCcccAACTTTCT
ATGGTTGACCTCGGATCAGGTAGGGATACCCGCTGAACTTAA
Uncultured fungus

>OTU_214
GAAATGCGATAAGTAATGTGAATTGCAGAATTCCGTGAATCATCGAATCTTTGAACGCACAT
TGCGCCcccTGGCATTCCGGggggCATGCCTGTCCGAGCGTCATTTCTGCCCTCAAGCGCGG
CTTGtgtgtTGGGTGTGGTCCccccGGTGTTGGggggACCTGCCCGAAAGGCAGCGGCGACG
TCCCGTCTAGGTCCTCGAGCGTATGGGGCTTTGTCACCCGCTCGGGAGGGGCCTGCGGGCGT
TGGCCACCCACGATATTtttttACCGTTGACCTCGGATCAGGTAGGAGTTACCCGCTGAACT
TAA
k__Fungi;p__Ascomycota;c__Eurotiomycetes;o__Eurotiales;f__Tric
hocomaceae;g__Penicillium;s__Penicillium_purpurogenum 1.000

>OTU_215
GAAATGCGATAAGTAATGTGAATTGCAGAATTCAGTGAATCATCGAATCTTTGAACGCACAT
TGCGCCCGCTGGTACTCCGGCGGGCATGCCTGTCCGAGCGTCATTTCAACCCTCAAGCCCTC
CTTCGGGACCGGGCCTGGTGTTGGGGCTCGGCCGCCATCTCCGGCGGCCGTCCCCTAAATAC
AGTGGCGGTCACGCCGCGACTCCCTTGCGTAGTAGAAACACCTCGCACCGGATAGCGACGCG
GTCCACGCCGTAAAACCcccAATTtttAATGGTTGACCTCGGATCAGGTAGGAATACCCGCT
GAACTTAA
k__Fungi;p__Ascomycota;c__Sordariomycetes;o__Hypocreales;f__un
identified;g__unidentified;s__Hypocreales_sp_2_TMS_2011 0.950

>OTU_216
GAAATGCGATAAGTAATGTGAATTGCAGAATTCAGTGAATCATCGAATCTTTGAACGCACAT
TGCGCCCCTTGGTATTCCGGggggCATGCCTGTTCGAGCGTCATTTCAACCCTCAAGCTTAG
CTTGGTATTGAGTCTATGTCAGTAATGGCAGGCTCTAAAATCAGTGGCGGCGCCGCTGGGTC
CTGAACGTAGTAATATCtctcGTTACAGGTTCTCGGTGTGCTTCTGCCAAAACCCAAATTtt
tCTATGGTTGACCTCGGATCAGGTAGGGATACCCGCTGAACTTAA
k__Fungi;p__Ascomycota;c__Leotiomycetes;o__Helotiales;f__Scler
otiniaceae;g__Botryotinia;s__Botryotinia_fuckeliana 1.000

>OTU_217
GAAATGCGATACTTGGTGTGAATTGCAGAATCCCGTGAACCATCGAGTCTTTGAACGCAAGT
TGCGCCCCAAGCCTTCTGGCCGAGGGCACGTCTGCCTGGGTGTCACAAATCGTCGTCCcccc
ATCCTCTCGAGGATATGGGACGGAAGCTGATCTCCCGTgtgtTACCGCACGCGGTTGGCCAA
AATCCGAGCTAAGGACGTCAGGAGCGTCTTGACATGCGGTGGTGAATTTAATTCTCGTCATA
TAGTCAGACGTTCCGGTCCAAAAGCTCTTGATGACCCAAAGTCCTCAACGCGACCCCAGGTC

AGGCGGGATCACCCGCTGAGTTTAA
Brassica rapa

>OTU_218
GAAACGCGATATGTAATGTGAATTGCAGAATTCAGTGAATCATCGAATCTTTGAACGCACAT
TGCGCCTTCCAGTATTCTGGGAGGCATGCCTGTCCGAGCGTCGTTTCAACCCTCAAGCCCTC
GTGGTTTGGTGTTGGAGTCCTACAGTCTTGTAGTCTCCCAAAACCAGTGGCGGACCCGTCGC
GGCCCTTCCTTTGCGTAGTAGCATTCGCCTCGCATCGGGAGCCCACGGGACTTTCTGGCCTC
TAAACCccccACAAGTCCGCTCCGGCGGCACAAGGTTGACCTCGGATCAGGTAGGAATACCC
GCTGAACTTAA
k__Fungi;p__Ascomycota;c__Sordariomycetes;o__Hypocreales;f__Ne
ctriaceae;g__Volutella;s__Volutella_colletotrichoides 1.000

>OTU_219
GATCCGCGATACGTCCTGGAAGCCGCCGTGAACCATCAATTTTCGAACGCACATTGCATACA
GGGAGGGTGGGGTGAGTCTACTCTCACAATCGTGGTTTAAATACAAAGGATGACTAATAAAA
CCGTCACCCCTCATTTTCTCCcccGCCcccTCAGATTACCCGCTGAACTTAAGCATATCAAT
AAGCGGAGGAGCATATCAATAAGCGGAGGAGTCCGTAGGTGAACCTGCGGA
k__Fungi;p__Ascomycota;c__Saccharomycetes 1.000

>OTU_220
GAAATGCGATACTTGGTGTGAATTGCAGAATCCCGTGAACCATCGAGTTtttGAACGCAAGT
TGCGCCTGAAGCCATTAGGCCGAGGGCACGTCTGCCTGGGCGTCACGCATTGTGTTGCCCAC
CCAACTCAAGTCTTACCAAGGATTGGGTGTGGGTGGCGGATATTGGCCccccGTGCACATT
AGTGAACGGTCGGCCTAAaaaTAAAGTCCTTAGCAATGGACGTCACAACAAGTGGTGGTTGA
CAAACCGTTGCGTCCTGTTGTGCTTGCCcccATTGCTAATGGTTTACTTTTGACCCTAATGT
GCTGTTATCACGGCTTCGATCGCGACCCCAGGTCAGGTGGGATTACCCGCTGAACTTAA
Actinidia henanensis

>OTU_221
AAAATGCGATAAGTAATGTGAATTGCAGAATTCAGTGAATCATCGAATCTTTGAACGCACAT
TGCGCCCGCCAGTATTCTGGCGGGCATGCCTGTTCGAGCGTCATTTCAACCCTCAAGCCCTC
GGGTTTGGTGTTGGGGATCGGCGAGCCTTTCTGGCAAGCCGGCCCCGAAATCTAGTGGCGGT
CTCACTGCAGCCTCCATTGCGTAGTAGCTAACACCTCGCAACTGGAACGCGGTGCGGCCAAG
CCGTTAAACCccccAACTTCTGAATGTTGACCTCGGATCAGGTAGGAATACCCGCTGAACTT
AA
k__Fungi;p__Ascomycota;c__Sordariomycetes;o__Hypocreales;f__Ne
ctriaceae;g__Fusarium;s__Fusarium_redolens 1.000

>OTU_222
GAAATGCGATAAGTAATGTGAATTGCAGAATTCAGTGAATCATCGAATCTTTGAACGCACAT
TGCGCCCGCCAGTATTCTGGCGGGCATGCCTGTTCGAGCGTCATTTCAACCCTCAAGACCCC
TTCGGgggACTTGGTGTTGGGGACCGGCACAGGGCCTGCTTGCTTGTCTCGCAGCGCCTCGC
CGCCTCCGAAATGAATTGGCGGCCTCGTCGCGGCCTCCTCTGCGTAGTAGCACAACCTCGCA

ACGGGAGCGTGACGCGGCCACTGCCGTAAAACAACCCAATTTTATTAGAGTTGACCTCGAAT
CAGGTAGGAATACCCGCTGAACTTAA
k__Fungi;p__Ascomycota;c__Eurotiomycetes;o__Eurotiales;f__Tric
hocomaceae;g__Paecilomyces;s__Paecilomyces_marquandii 1.000

>OTU_223
GAAATGCGATAACTAGTGTGAATTGCAGAATTCCGTGAATCATCGAGTCTTTGAACGCACAT
TGCGCCCGCCAGAATACTGGCGGGCATGCCTGTCCGAGCGTCATTGCTGCCCTCAAGCACGG
CTTGtgtgtTGGGCTTCCGTCCCTGGTAACGGGGACGGGCCCAAAAGGCAGTGGCGGCACCA
TGTCTGGTCCTCGAGCGTATGGGGCTTTGTCACCCGCTCCCGTAGGTCCAGCTGGCAGCTAG
CCTCGCAACCAATCTTtttAACCAGGTTGACCTCGGATCAGGTAGGGATACCCGCTGAACTT
AA
k__Fungi;p__Ascomycota;c__Eurotiomycetes;o__Eurotiales;f__Tric
hocomaceae;g__Eurotium;s__Eurotium_niveoglaucum 1.000

>OTU_224
GAAATGCGATAAGTAATGTGAATTGCAGAATTCAGTGAATCATCGAATCTTTGAACGCACAT
TGCGCCCCTTGGTATTCCGGggggCATGCCTGTTCGAGCGTCATTATAACCCTCAAGCCTAG
CTTGGTGTTGGAGCATGCTACCTAGCAGCTCTTAAAATCAGTGGCAGTGCCCTTCGGCTCTA
AGCGTAGTAATTTCtctcGTTATAGAACCCGGATAGTATGCGCCAAAACCCCAACTTTCTAT
GGTTGACCTCGGATCAGGTAGGGATACCCGCTGAACTTAA
Uncultured fungus

>OTU_225
GAAATGCGATAAGTAATGTGAATTGCAGAATTCAGTGAATCATCGAATCTTTGAACGCACCT
TGCGCCTTTTGGTATTCCGAGAGGCATGCCTGTTTGAGTGTCATTAAATTCTCAACTTCAAA
AGCTTTTGTTtttGAAGCTTGGATGTGGAGGCTTTGCTGGCCCTTCTAGAGTCGGCTCCTCT
GAAATGCATTAGTGGAAACTGTTTGCAATCCGCATTGGTGTGATAATTATCTACGCTTGtgt
gtgGTTGCAGCTCTTTACGAGTTTAGTATCTGCTTCAAACCGTCCTAAGTCACTGGACAACT
TTGAACCTTTTGACCTCAAATCAGGTAGGACTACCCGCTGAACTTAA
k__Fungi;p__Basidiomycota;c__Agaricomycetes;o__Agaricales;f__M
arasmiaceae;g__Marasmius;s__Marasmius_nigrobrunneus 0.990

>OTU_226
GAAATGCGATACGTAATGTGAATTGCAGAATTCAGTGAATCATCGAGTCTTTGAACGCACAT
TGCGCCcccTGGTATTCCGGggggCATGCCTGTCCGAGCGTCATTGCTGCCCTCAAGCACGG
CTTGtgtgtTGGGCCcccGTCCcccTCTTttttAGGggggggggACGGGCCCGAAAGGCAGC
GGCGGCACCGTGTCCGGTCCTCGAGCGTATGGGAAGCAACCAATCTTtttAACCAGGTTGAC
CTCGGATCAGGTAGGGATACCCGCTGAACTTAA
k__Fungi;p__Ascomycota;c__Eurotiomycetes;o__Eurotiales;f__Tric
hocomaceae;g__Aspergillus;s__Aspergillus_penicillioides 1.000

>OTU_227
GAAATGCGATACTTGGTGTGAATTGCAGGATCCCGTGAACCACCGAGTCTTTGAACGCAAGT

TGCGCCCGGAGCCTTCTGGCCGAGGGCACGTCTGCCTGGGCGTCACGCATCGTTGTCCCCAC
CCAcacaACTCCcccGCCCACCGTGCGGGCTCGTTGTGCGGGCGAGGGCAcacacTGGCCTA
CCGTGCGCACCGTCGTGCGGATGGCTTAAATTCGAGTCCTCGACGCTCGTCGTCGCGACACT
ACGGTGGTTGATCCAACCTCGGTGACGCGTCTCGGCCTCGACGTCGCCTCCACGGACTCCTG
CATGACCCTCCGAACGTCGCCcccGCAAAGGGACGACACTCTCGACGCGACCCCAGGTCAGG
CGGGACTACCCGCTGAGTTTAA
Cucumis maderaspatanus

>OTU_228
GCTGGCATAGCGGTATGTACCATGTTCCACCGGCAGACTGTCCGGATCCAGACCGTATTTTA
TCATGGAGGCACGCCAGCCGCGGTGACGCATTCTCGTAGAATCCAGATTTGAATTGCCTTCG
ATAATTCCGATTCTGCGGTGCCCGTTTTCCAGAAGATAATCCATGGCCTTTTCCATCGCTCC
GGCCTCATCTGTGGTAACGTTGGGCACGTCCAAaaaTACCGTCCGGCAGATCGCCACCATGG
GAATCTCCTTATTCAGTACTTCATCCATAAATTCCAGATCGTCATCCTTCTGCGTGAGCACG
ATAATCCCGTCCAAaaaaGAGGGGTTCAGCGTCCCCGGCTCAT
No significant similarity

>OTU_229
GAAATGCGATAAGTAGTGTGAATTGCAGAATTCAGTGAATCATCGAATCTTTGAACGCACAT
TGCGCCCCTTGGTATTCCATGGGGCATGCCTGTTCGAGCGTCATTTGTACCCTCAAGCTTTG
CTTGGTGTTGGGCGTCTTGTCTACCTCTTTAGCGGGTAGACTCGCCTTAAAGTAATTGGCAG
CCAGTGTTtttGGCAGTAAGCGCAGCACATTTTGCGTCTTAGTCCCTAAACAGTGGCATCCA
CAAAGCCTCTTTCTCACTTTTGACCTCGGATCAGGTAGGGATACCCGCTGAACTTAA
k__Fungi;p__Ascomycota;c__Dothideomycetes;o__Pleosporales;f__u
nidentified;g__unidentified;s__Pleosporales_sp_3_TMS_20111.000

>OTU_230
GAAATGCGATAAGTAGTGTGAATTGCAGAATTCAGTGAATCATCGAATCTTTGAACGCACAT
TGCGCCCCTTGGTATTCCATGGGGCATGCCTGTTCGAGCGTCATCTACACCCTCAAGCTCTG
CTTGGTGTTGGGCGTCTGTCCCGCCTCTGCgcgcgGACTCGCCCCAAATTCATTGGCAGCGG
TCTTTGCCTCCTCTCGCGCAGCACAATTGCGTAGTAAGGgggTGCGGGTCGCGTCCACGAAG
CAACATTACCGTCTTTGACCTCGGATCAGGTAGGGATACCCGCTGAACTTAA
k__Fungi;p__Ascomycota;c__Dothideomycetes;o__Pleosporales;f__D
idymosphaeriaceae;g___Paraphaeosphaeria_neglecta   0.970

>OTU_231
GAAATGCGATAATTAATGTGAATTGCAGAATTCAGTGAATCATCGAGTCTTTGAACGCACAT
TGCGCCccccGGAATGCCTGTCCGAGCGTCATTTCTGCCCTCAAGCACGGCTTGtgtgtTGG
GCTTCCGTCCCTGGTAACGGGGACGGGCCCAAAAGGCAGTGGCGGCACCATGTCTGGTCCTC
GAGCGTATGGGGCTTTGTCACCCGCTCCCGTAGGTCCAGCTGGCAGCTAGCCTCGCAACCAA
TCTTtttAACCAGGTTGACCTCGGATCAGGTAGGGATACCCGCTGAACTTAA
k__Fungi;p__Ascomycota;c__Eurotiomycetes;o__Eurotiales;f__Tric
hocomaceae;g__Eurotium;s__Eurotium_niveoglaucum 1.000

>OTU_232
GAAATGCGATACCTGGTGTGAATTGCAGAATCCCGTGAACCATCGAGTTtttGAACGCAAGT
TGCGCCCGAGGCCTTCTGGTCGAGGGCACGCCTGCCTGGGCGTCACGTCAAAAGACACTCCC
AACCCATCCAAGGGGAGGGACGTGGTGTTTGGCCTCCCGTGCCGCAAGGCGCGGTTGGTCGA
AGTTGAGGCTGCCGGCATACCGTGTCGGGCACCGCACGTGGTGGGCGACTTCAAGTTGTTCT
CGGTGCAGCGCCCCGGCACGTAGCTAGCGTGTTGCCCTAAGGACCCATCGACCGTAGCGCTT
GCCGCTCGGACCGCGACCCCAGGTCAGACGAGACTACCCGCTGAGTTTAA
Salvia tesquicola

>OTU_233
GAAATGCGATAAGTAATGTGAATTGCAGAATTCCGTGAATCATCGAATCTTTGAACGCACAT
TGCGCCcccTGGCATTCCGGggggCATGCCTGTCCGAGCGTCATTTCTGCCCTCAAGCACGG
CTTGtgtgtTGGGTGTGGTCCccccGGGGACCTGCCCGAAAGGCAGCGGCGACGTCCGTCTG
GTCCTCGAGCGAGTGACAGAGCCCCATACGCTCGGGAAGGACCTGCGGgggTTGGTCACCAC
CATGTTtttACCACGGTTGACCTCGGATCAGGTAGGAGTTACCCGCTGAACTTAA
k__Fungi;p__Ascomycota;c__Eurotiomycetes;o__Eurotiales;f__Tric
hocomaceae;g__Penicillium;s__Penicillium_pinophilum 0.960

>OTU_234
GAAATGCGATAACTAGTGTGAATTGCAGAATTCCGTGAATCATCGAGTCTTTGAACGCACAT
TGCGCCcccTGGTATTCCGGggggCATGCCTGTCCGAGCGTCATTGCTGCCCATCAAGCACG
GCTTGtgtgtTGGGTCGTCGTCCCCTCTTCGGgggggACGGGCCCCAAAGGCAGCGGCGGCA
CCGCGTCCGATCCTCGAGCGTATGGGGCTTTGTCACCCGCTCTGTAGGCCCGGCCGGCGCTT
GCCGAACGCAAAACAACCATTCTTTCCAGGTTGACCTCGGTTCAGGTAGGGATACCCGCTGA
ACTTAAGCAGATCAATAAGCGGAGGAGCATATCAATAAGCGGAGGAGCATATCAATAAGCGG
AGGA
k__Fungi;p__Ascomycota;c__Eurotiomycetes;o__Eurotiales;f__Tric
hocomaceae;g__Aspergillus;s__Aspergillus_tamarii 0.990

>OTU_235
GAAATGCGATAAGTAATGCGAATTGCAGAATTTCCGTGAGTCATCGAATCTTTGAACGCACA
TTGCGCCCACTGGTATTCCGGTGGGCATGCCTGTTCGAGCGTCATTATCCTCCCTCAAACCT
CTGTGTTTGGTGTTGGGCCGCGTTGGCGTCACAGCCAACTGGTCTCAAAGACAATGACGGCG
TCCGTGGGACCCTCTTCGCAACGAGCATCTTCGGAAGCACGCGTCGAGTCTAAAGGACCTTC
CGGGCCGGTCTACCCTTTATCTTTCTAAGGTTGACCTCGGATCAGGTAGGAATACCCGCTGA
ACTTAA
k__Fungi;p__Ascomycota;c__Eurotiomycetes;o__Incertae_sedis;f__
Incertae_sedis;g__Sarcinomyces;s__Sarcinomyces_sp_SL_2011 1.000

>OTU_236
AAAATATGATACTTAGTATGAATTGCAGAATCCCTTGAACTATCAAGTCTCTAAAAGCAAGT
TGCACTCGAAGCCATCAGGCCTAGGGCATGTCTGCCTAGGCATCAAGCATCGCATCCccccT
ATATGCTGCTCAGCATCATGGTGCCGGATATTGGCCTCCCGTGTGAGTCCACGTCTACGAAC
ATCGCAGTAAGTAGTGGTTGTAACTCTACTctctTAGTGCCGCGGCTACAGCTCATCGCACG

TgtgtgCTCCCCGACCCTTCTGACTCTCGcgcgcTCCAACTGCAATCCCAGGTCAGGCGGGG
TTACCTACTGAGTTTAA
No significant similarity

>OTU_237
GAAATGCGAAAAGCAATGCGAATTGCAGAATTCCGCGAGTCATCAGATCTTCGAACGCACCT
CGCGCCAATCGGATCTCCGATCGGCATGTTTGATTCAGTGTCTTGTTGAGACTTGCATATCT
CATTGTGATTGACTCTTCGCTACTTGTAGAGAAGTGTCTTTCATGAAAATAGATTGCGCATA
CCGCAACGATGCAGTAGTCAGTTAACGCTGATACTCATCGGTGAAATGGTATGATTACGTAA
TGTTGGAAACAACAATACATAAAACTCATCCACATGCATCTGAATCAAGCAAGAACACCCGC
TGAACTTAA
Uncultured fungus

>OTU_238
GAAATGCGATACGTAGTGTGAATTGCAGAATTCAGTGAATCATCGAATCTTTGAACGCACAT
TGCGCCCTTTGGTATTCCAAAGGGCATGCCTGTTCGAGCGTCATTTGTACCCTCAAGCTTTG
CTTGGTGTTGGGCGTTttttGTCTTGCTCTCAGCAAGACTCGCCTTAAAATGATTGGCAGCC
GGCCTACTGGTTTCGGAGCGCAGCAGATTCTTtttGCGCTTGCAACCAGCAAAAGAGGTTGG
CGATCCAGCAAGTACATCTTCTCACTTTTGACCTCGGATCAGGTAGGGATACCCGCTGAACT
TAA
k__Fungi;p__Ascomycota;c__Dothideomycetes;o__Pleosporales;f__P
leosporaceae;g__Bipolaris 0.990

>OTU_239
GAAATGCGATACTTGGTGTGAATTGCAGAATCCCGTGAACCATCGAGTCTTTGAACGCAAGT
TGCGCCCGAAGCCCTTAGGCTGAGGGCACGCCTGCCTGGGTGTCACCAAAAGGCGCCccccc
GTCTCGCCCGTCCCAGGGCACGGGGAGGgggCGCACGTTGGCCTCCCGGGAGCCCCTGGCCC
GCGGTTGGTTCAAAGAGACGGGCTCTTGGTGGggggCGGCACCGAGGCAGAGGGTGGGCGAG
AACAGCCCGCGTGGCCAGTCGcgcgcgcCTCTCCccccGGTTCAGGGCACGGGGACCCGCGGG
CGACGGGGATCGTCCCGAGCGCGACCGCAGGTCAGGCGGGGCTACCCGCTGAGTTTAA
Arachis hypogaea

>OTU_240
AAATTGCGATAACTAGTGTGAATTGCAAATTCAGTGAATCATCGAGTCTTTGAACGCATCTT
GCGCTCATTGGTATTCCAGTGAGCACGCCTGTTTCAGTATCAAaaaCAACCCTCATTCAAAA
GTTttttCTTATGAATGGTTATGAAGGAAGCTTTAGCCCTTGGGCTTGGCAACCTTTTAAAT
TGAGTAAGGCCTGAATCTGTTTCATCTAGCCTGAACTTttttttAATATAAAGGAAAGCTCT
TGCGACTTGAACTTTGTTGGGGCCTCCCAAATAAAACTTttttCATCTTGATCTGAAATCAG
GTGGGACTACCCGCTGAACTTAA
k__Fungi;p__Zygomycota;c__Incertae_sedis;o__Mucorales;f__Choan
ephoraceae;g__Poitrasia;s__Poitrasia_circinans 1.000

>OTU_241
GAAATGCGATAACTAATGTGAATTGCAGAATTCAGTGAATCATCGAGTCTTTGAACGCACAT

TGCGCCcccTGGTATTCCGGggggCATGCCTGTCCGAGCGTCATTGCTGCCCTCAAGCACGG
CTTGtgtgtTGGGCCCCGTCCCCGGTACCcccGGGGACGGGCCCGAAAGGCAGCGGCGGCAC
CGCGTCCGGTCCTCGAGCGTATGGGGCTTTGTCACCCGCTCTGCAGGCCCGGCCGGCGCCAG
CCGACCAACCCAACCATTTTCTACAGGTTGACCTCGGATCAGGTAGGGATACCCGCTGAACT
TAA
k__Fungi;p__Ascomycota;c__Eurotiomycetes;o__Eurotiales;f__Trichocomaceae;g__Aspergillus;s__Aspergillus_candidus 1.000

>OTU_242
AAACTGCGATAAGTAGCGTGAATTGCAGACGCTTTGAACGTTAAACTTTCGAACGCACATTG
CGCCGTAGGAGTCCTACCCTGCGGCACATCTGGTTGAGGGTCGTGATCAAaaaCAGCAGGAC
TATGGCTGTAATAGAAATAGCTGGTGAATCACGAGTTCGGCCTACCGCCTCTGGCCGAACGT
GTTTTGCCATAATGCTAGAAGAGGACAATCACTCGGCAATCGAGTGATTGCCTACCACACGG
ATTATCTATTAGCTGAAGCAATAGGTCTCGGTCACCGGGTATCATACTAACTAtataCTCTG
TGGCGTCTGAGATACAGCTAGTGGGCTTCATACGTGCCTGGTATTGAGAGTATTCGCTCTCC
CGACCTCAACTCAGGTGTGATTACCCGCTGAACTTAA
Pratylenchus goodeyi

>OTU_243
GAAATGCGATAAGTAATGTGAATTGCAGAATTCAGTGAATCATCGAATCTTTGAACGCACCT
TGCGCCCTCTGGTATTCCGGAGGGCATGCCTGTTTGAGTGTCATGTAGACTCAATCCCTCGG
GTTTCCGAGGAGATTGGACTTGGGTGTTGCCGCTTCTGCCGGCTCACCTTAAAAGACTTAGC
GGGATAGCACCGTAGTCGGCGTAATAAGTTTCGTCGGTGAAGGTTGTGATGACTGCTTACAA
TCGCCCTCGGGCAATTtttGACTCTGACCTCAAATCAGGTAGGACTACCCGCTGAACTTAA
k__Fungi;p__Basidiomycota;c__Tremellomycetes;o__Filobasidiales;f__Filobasidiaceae;g__Cryptococcus;s__Cryptococcus_podzolicus 0.990

>OTU_244
GAAATGCGATAAGTAATGTGAATTGCAGAATTCAGTGAATCATCGAATCTTTGAACGCACAT
TGCGCCCTTTGGCATTCCGAGGGGCATGCCCGTTCGAGCGTCATTAAAACCCCTCAAGCCTA
GCTTGGTGTTGGGTGTCGGGGCCcccccGCACCTCTATATCGCCGGCTGGCCGTCGTCTGAC
TTAGCGTTGTGACACCATGTCGCTGATGCCAGACGCGGACTGCGCCGTTAGAACTctctTGA
ATGTCCCCGGACGTTCAGTACAGGTTGACCTCGGATCGGGTAGGAATACCCGCTGAACTTAA
k__Fungi;p__Ascomycota;c__Dothideomycetes;o__Capnodiales;f__Teratosphaeriaceae;g__Devriesia;s__Devriesia_strelitziicola 0.920

>OTU_245
GAAATGCGATAACTAATGTGAATTGCAGAATTCAGTGAATCATCGAGTCTTTGAACGCACAT
TGCGCCcccTGGTATTCCGGggggCATGCCTGTCCGAGCGTCATTGCTGCCCTCAAGCCCGG
CTTGtgtgtTGGGCCccccGTCCcccTCTTttttAGGgggggggACGGGCCCGAAAGGCAGC
GGCGGCACCGTGTCCGGTCCTCGAGCGTATGGGGCTTTGTCACCCGCTCTGTAGGCCCGGCC
GGCGCTTGCCGAACGCAAATCAATCTTtttCCAGGTTGACCTCGGATCAGGTAGGGATACCC
GCTGAACTTAA

k__Fungi;p__Ascomycota;c__Eurotiomycetes;o__Eurotiales;f__Tric
hocomaceae   1.000

Table S2 OTUs sequence and annotation in stored peanut kernels

>OTU_1
AAAGTGCGATAACTAGTGTGAATTGCATATTCAGTGAATCATCGAGTCTTTGAACGCAGCTT
GCACTCTATGGTTtttCTATAGAGTACGCCTGCTTCAGTATCATCACAAACCCAcacaTAAC
ATTTGTTTATGTGGTGATGGGTCGCATCGCTGTTTTATTACAGTGAGCACCTAAAATGtgtg
tgATTTTCTGTCTGGCTTGCTAGGCAGGAATATTACGCTGGTCTCAGGATCTTttttttGG
TTCGCCCAGGAAGTAAAGTACAAGAGTATAATCCAGTAACTTTCAAACTATGATCTGAAGTC
AGGTGGGATTACCCGCTGAACTTAAGCATATCAATAAGCGGAGGAGCATATCAATAAGCGGA
GGATGAGTCCGTAGGTGAACCTGCATATCAATAAGCGGAGGATGAGTCCGTAGGTGAACCTG
C
k__Fungi;p__Zygomycota;c__Incertae_sedis;o__Mucorales;f__Mucor
aceae;g__Rhizopus;s__Rhizopus_oryzae 1.000

>OTU_2
GAAATGCGATAATTAATGTGAATTGCAGAATTCAGTGAATCATCGAGTCTTTGAACGCACAT
TGCGCCcccTGGTATTCCGGggggCATGCCTGTCCGAGCGTCATTGCTGCCCTCAAGCACGG
CTTGtgtgtTGGGCTTCCGTCCCTGGTAACGGGGACGGGCCCAAAAGGCAGTGGCGGCACCA
TGTCTGGTCCTCGAGCGTATGGGGCTTTGTCACCCGCTCCCGTAGGTCCAGCTGGCAGCTAG
CCTCGCAACCAATCTTtttAACCAGGTTGACCTCGGATCAGGTAGGGATACCCGCTGAACTT
AAGCATATCAATAAGCGGAGGAGCATATCAATAAGCGGAGGAGCATATCAATAAGCGGAGGA
GCATATCAATAAGCGGAGGAGCATATCAATAAGCGGAGGATGAGTCCGTAGGTGAACCTGCG
GAAGGATAGACC
k__Fungi;p__Ascomycota;c__Eurotiomycetes;o__Eurotiales;f__Tric
hocomaceae;g__Aspergillus;s__Aspergillus_glaucus 1.000

>OTU_3
GAAATGCGATAAGTAATGTGAATTGCAGAATTCCGTGAATCATCGAATCTTTGAACGCACAT
TGCGCCcccTGGCATTCCGGggggCATGCCTGTCCGAGCGTCATTTCTGCCCTCAAGCACGG
CTTGtgtgtTGGGTGTGGTCCccccGGGGACCTGCCCGAAAGGCAGCGGCGACGTCCGTCTG
GTCCTCGAGCGTATGGGGCTCTGTCACTCGCTCGGGAAGGACCTGCGGgggTTGGTCACCAC
CATGTTtttACCACGGTTGACCTCGGATCAGGTAGGAGTTACCCGCTGAACTTAAGCATATC
AATAAGCGGAGGAATCCGTAGGTGAACCTGCGGGCCAGGTTGAAGAACGCAGCGTCGTCGCA
TCGATGAAGAACGCA
k__Fungi;p__Ascomycota;c__Eurotiomycetes;o__Eurotiales;f__Tric
hocomaceae;g__Penicillium;S__Penicillium_pinophilum   0.940

>OTU_4
GAAATGCGATACTTGGTGTGAATTGCAGAATCCCGTGAACCATCGAGTCTTTGAACGCAAGT
TGCGCCCGAAGCCCTTAGGCTGAGGGCACGCCTGCCTGGGTGTCACCAAAAGGCGCCccccc
GTCTCGCCCGTCCCAGGGCACGGGGAGGgggCGAACGTTGGCCTCCCGGGAGCCCCTGGCTC
GCGGTTGGTTCAAAGAGACGGGCTCTTGGTGGGGAGCGGCACCGCGGCAGATGGTGGTCGAG
AACAACCCTCGTGGCCAGTCGcgcgcgcCTCTCCcccGGTTCAAGGCACGGCGACCCGCGGG
CGACGTGGATCGTCCCGAGCGCGACCTCAGGTCAGGCGGGGCTACCCGCTGAGTTTAAGCAT

ATCAATAAGCGGAGGATCCGTAGGTGAACCTGCG
Arachis hypogaea 1.000

>OTU_5
GAAATGCGATAACTAATGTGAATTGCAGAATTCAGTGAATCATCGAGTCTTTGAACGCACAT
TGCGCCcccTGGTATTCCGGggggCATGCCTGTCCGAGCGTCATTGCTGCCCTCAAGCCCGG
CTTGtgtgtTGGGTCGCCGTCCcccTCTCCGGggggACGGGCCCGAAAGGCAGCGGCGGCAC
CGCGTCCGATCCTCGAGCGTATGGGGCTTTGTCACATGCTCTGTAGGATTGGCCGGCGCCTG
CCGACGTTTTCCAACCATTCTTTCCAGGTTGACCTCGGATCAGGTAGGGATACCCGCTGAAC
TTAAGCATATCAATAAGCGGAGGAGCATATCAATAAGCGGAGGAGCATATCAATAAGCGGAG
GA
k__Fungi;p__Ascomycota;c__Eurotiomycetes;o__Eurotiales;f__Tric
hocomaceae;g__Aspergillus;s__Aspergillus_niger 0.900

>OTU_6
GAAATGCGATAAGTAATGTGAATTGCAGATACAGTGAATCATCGAATCTTTGAACGCAAATG
GCACTCTATGGTATTCCGTAGAGTACGTCTGTTTGAGCGTCGCGAACATCTCCATAATTGGT
TtttttAAATTGATTGTGGGTTTTGAGGTTGTCATATAAACAATGACTCCCTTTAAAATAAT
TAGTGATGACCTTATGAATGGGTTAATACTGtgtgtTATAATGGATTACATCCATCACCAGT
CAGagagTAATCTCGCCTTAGTAATTTGTAGTGATTGCTTCTAACTGCCATTGGCAAACAAA
CTGATCAAATCGACCTCAAATCAGATGGGATTACCCGCTGAACTTAAGCATATCAATAAGCG
GAGGAGCATATCAATAAGCGGAGGAGCATATCAATAAGCGGAGGAATCCGTAGGTGAACCTG
CGGAC
k__Fungi;p__Basidiomycota;c__Wallemiomycetes;o__Wallemiales;f_
_Wallemiaceae;g__Wallemia;s__Wallemia_sebi 1.000

>OTU_7
GAAATGCGATAACTAGTGTGAATTGCAGAATTCCGTGAATCATCGAGTCTTTGAACGCACAT
TGCGCCcccTGGTATTCCGGggggCATGCCTGTCCGAGCGTCATTGCTGCCCATCAAGCACG
GCTTGtgtgtTGGGTCGTCGTCCCCTCTCCGGgggggACGGGCCCCAAAGGCAGCGGCGGCA
CCGCGTCCGATCCTCGAGCGTATGGGGCTTTGTCACCCGCTCTGTAGGCCCGGCCGGCGCTT
GCCGAACGCAAATCAATCTTTTCCAGGTTGACCTCGGATCAGGTAGGGATACCCGCTGAACT
TAAGCATATCAATAAGCGGAGGAATCCGTAGGTGAACCTGCGGCATATCAATAAGCGGAGGA
k__Fungi;p__Ascomycota;c__Eurotiomycetes;o__Eurotiales;f__Tric
hocomaceae;g__Aspergillus;s__Aspergillus_flavus 1.000

>OTU_8
GAAATGCGATAATTAATGTGAATTGCAGAATTCAGTGAATCATCGAGTCTTTGAACGCACAT
TGCGCCcccTGGTATTCCGGggggCATGCCTGTCCGAGCGTCATTGCTGCCCTCAAGCACGG
CTTGtgtgtTGGGCTTCCGTCCCTGGCAACGGGGACGGGCCCAAAAGGCAGTGGCGGCACCA
TGTCTGGTCCTCGAGCGTATGGGGCTTTGTCACCCGCTCCCGTAGGTCCAGCTGGCAGCTAG
CCTCGCAACCAATCTTtttAACCAGGTTGACCTCGGATCAGGTAGGGATACCCGCTGAACTT
AAGCATATCAATAAGCGGAGGAGTCCGTAGGTGAACCTGCGGAGCATATCAATAAGCGGAGG
AGTCCGTAGGTGAACCTGCG

k__Fungi;p__Ascomycota;c__Eurotiomycetes;o__Eurotiales;f__Trichocomaceae;g__Eurotium;s__Eurotium_cristatum 0.900

>OTU_9
GAAATGCGATAAGTAATGTGAATTGCAGAATTCAGTGAATCATCGAATCTTTGAACGCACATTGCGCCCCTTGGTATTCCGGggggCATGCCTGTTCGAGCGTCATTACAACCCTCAAGCTCTGCTTGGAATTGGGCACCGTCCTCACTGCGGACGCGCCTCAAAGACCTCGGCGGTGGCTGTTCAGCCCTCAAGCGTAGTAGAATACACCTCGCTTTGGAGTGGTTGGCGTCGCCCGCCGGACGAACCTTCTGAACTTTTCTCAAGGTTGACCTCGGATCAGGTAGGGATACCCGCTGAACTTAAGCATATCAATAAGCGGAGGATTCCGTAGGTGAACCTGCGGCATATCAATAAGCGGAGGATGAGTCCGTAGGTGAACCTGCG
k__Fungi;p__Ascomycota;c__Dothideomycetes;o__Botryosphaeriales;f__Botryosphaeriaceae;g__Lasiodiplodia;s__Lasiodiplodia_theobromae 1.000

>OTU_10
GAAATGCGATAAGTAATGTGAATTGCAGAATTCAGTGAATCATCGAATCTTTGAACGCACATTGCGCCCGCCAGTATTCTGGCGGGCATGCCTGTCTGAGCGTCATTTCAACCCTCATGCCCCTAGGGCGTGGTGTTGGGGATCGGCCAAAGCCCGCGAGGGACGGCCGGCCCCTAAATCTAGTGGCGGACCCGTCGTGGCCTCCTCTGCGAAGTAGTGATATTCCGCATCGGAGAGCGATGAGCCCCTGCCGTTAAACCcccAACTTTCTAAGGTTGACCTCAGATCAGGTAGGAATACCCGCTGAACTTAAGCATATCAATAAGCGGAGGAGCATAGCAATAAGCGGAGGAGCATATCAATAGCGGAGGAGCATATCAATAAGCGGAGGAATCCGTAGGTGAACCTGCGGA
k__Fungi;p__Ascomycota;c__Sordariomycetes;o__Hypocreales;f__Bionectriaceae;g__Bionectria;s__Clonostachys rosea 1.000

>OTU_11
GAAATGCGATAAGTAATGTGAATTGCAGATACAGTGAATCATCGAATCTTTGAACGCAAATGGCACTCTATGGTATTCCGTAGAGTACGTCTGTTTGAGCGTCGCGAACATCTCCATAATTAGTTtttttAAATTGATTGTGGGTTTTGAGGTTGTCATATAACAATGACTCCCTTTAAAATAATTAGTGATGACCTTATGAATGGGTTAATACTGtgtgtTATAATGGATTACATCCATCACCAGTCAGagagTAATCTCGCCTTAGTAATTTGTAGTGATTGCTTCTAACTGCCATTGGCAAACAAACTGATCAAATCGACCTCAAATCAGATGGGATTACCCGCTGAACTTAAGCATATCAATAAGCGGAGGAGCTACCCCATCGATGAAGAACGCAGCATATCAATAAGCGGAGGAATCCGTAGGTGAACCTGCG
k__Fungi;p__Basidiomycota;c__Wallemiomycetes;o__Wallemiales;f__Wallemiaceae;g__Wallemia 1.000

>OTU_12
GAAATGCGATACGTAATGTGAATTGCAGAATTCAGTGAATCATCGAGTCTTTGAACGCACATTGCGCCccccTGGTATTCCGGggggCATGCCTGTCCGAGCGTCATTGCTGCCCTCAAGCACGGCTTGtgtgtTGGGCCccccGTCCccccTCCCAGGAAGGGGACGGGCCCGAAAGGCAGCGGCGGCACCGTGTCCGGTCCTCGAGCGTATGGGAAGCAACTTTtttGTCACCCGCTCCTGTAGGTCCGGCCGGCGGCCTGCCCAACCCCAACCTTtttttAACCAGGTTGACCTCGGATCAGGTAGGGATA

CCCGCTGAACTTAAGCATATCAATAAGCGGAGGA
k__Fungi;p__Ascomycota;c__Eurotiomycetes;o__Eurotiales;f__Tric
hocomaceae;g__Aspergillus;s__Aspergillus_penicillioides 1.000

>OTU_13
AAAGTGCGATAACTAGTGTGAATTGCATATTCAGTGAATCATCGAGTCTTTGAACGCAGCTT
GCACTCTATGGTTtttCTATAGAGTACGCCTGCTTCAGTATCATCACAAACCCAcacaTAAC
ATTTGTTTATGTGGTGATGGGTCGCATCGCTGTTTTATTACAGTGAGCACCTAAAATGtgtg
tgATTTTCTGTCTGGCTTGCTAGGCAGGAATATTACGCTGGTCTCAGGATCTTtttCTTTGG
TTCGCCCAGGAAGTAAAGTACAAGAGTATAATCCAGTAACTTTCAAACTATGATCTGAAGTC
AGGTGGGATTACCCGCTGAACTTAAGCATATCAATAAGCGGAGGAGTCCGTAGGTGAACCTG
CGGCATATCAATAAGCGGAGGA
k__Fungi;p__Zygomycota;c__Incertae_sedis;o__Mucorales;f__Mucor
aceae;g__Rhizopus;s__Rhizopus_oryzae 1.000

>OTU_14
GAAATGCGATAAGTAATATGAATTGCAGATATTCGTGAATCATCGAATCTTTGAACGCACAT
TGCGCCCTTTGGTATTCCAAAGGGCATGCCTGTTTGAGCGTCATTTCTCCCTCAAACCCTCG
GGTTTGGTGTTGAGCGATACGCTGGGTTTGCTTGAAAGAAAGGCGGAGTATAAACTAATGGA
TAGGTTttttCCACTCATTGGTACAAACTCCAAAACTTCTTCCAAATTCGACCTCAAATCAG
GTAGGACTACCCGCTGAACTTAAGCATATCAATAAGCGGAGGATGAGTCCGTAGGTGAACCG
GCGCATATCAATAAGCGGAGGATGAGTCCGTAGGTGAACCTGCGGAGCATATCAATAAGCGG
AGGAGTCCGTAGGTGAACCTGCG
k__Fungi;p__Ascomycota;c__Saccharomycetes;o__Saccharomycetales
;f__Incertae_sedis;g__Candida;s__Candida_parapsilosis 1.000

>OTU_15
GAAATGCGATACGTAATGTGAATTGCAGAATTCAGTGAATCATCGAGTCTTTGAACGCACAT
TGCGCCcccTGGTATTCCGGggggCATGCCTGTCCGAGCGTCATTGCTGCCCTCAAGCACGG
CTTGtgtgtTGGGCCcccGTCCcccTCTTttttAGGggggggggACGGGCCCGAAAGGCAGC
GGCGGCACCGTGTCCGGTCCTCGAGCGTATGGGAAGCAACTCTTtttGTCACCCGCTCCTGT
AGGTCCGGCCGGCGGCCTGCCCAACCCTCAATCAATCTTttttAACCAGGTTGACCTCGGAT
CAGGTAGGGATACCCGCTGAACTTAAGCATATCAATAAGCGGAGGAATCCGTAGGTGAACCT
k__Fungi;p__Ascomycota;c__Eurotiomycetes;o__Eurotiales;f__Tric
hocomaceae;g__Aspergillus;s__Aspergillus_penicillioides 1.000

>OTU_16
GAAATGCGATAAGTAATGTGAATTGCAGATACAGTGAATCATCGAATCTTTGAACGCATATT
GCACCTTTTGGTATTCCATAAGGTACGTCTGTTTGAGCGTCGCGAACATCTCATAATTAATG
AATTtttttttGTTAATTATGGTCTTTGAGTTTGTCTATAATTtttAGACTCACTTTAAATT
GATTAGTAGTTTAACTTTTGAAAGGGTTAAAATTAGGTGTTttttAATGTACATTACTTTGT
GCATCATCTAATCAAGAGTTACTTACTCTGCCTTAGTATTAATGTTACTGCTTCTAATAGCT
TATTAAGCAAGTAATATTTCAATCGACCTCAAATCAGATGGGATTACCCGCTGAACTTAAGC
ATATCAATAAGCGGAGGAATCCGTAGGTGAACCTGCGGCATATCAATAAGCGGAGGAATCCG

TAGGTGAACCTGCGGA
Uncultured fungus 1.000

>OTU_17
GAAATGCGATAAGTAATGTGAATTGCAGAATTCAGTGAATCATCGAGTCTTTGAACGCACAT
TGCGCCccccTGGTATTCCGGggggCATGCCTGTCCGAGCGTCATTGCTGCCCTCAAGCACGG
CTTGtgtgtTGGGCTTCGCCccccGGCTCCCGGggggCGGGCCCGAAAGGCAGCGGCGGCAC
CGCGTCCGGTCCTCGAGCGTATGGGGCTTCGTCACCCGCTCTGTAGGCCCGGCCGGCGCCCG
CCGGCGACCCCAATCAATCTTTCCAGGTTGACCTCGGATCAGGTAGGGATACCCGCTGAACT
TAA
k__Fungi;p__Ascomycota;c__Eurotiomycetes;o__Eurotiales;f__Tric
hocomaceae;g__Penicillium;s__Penicillium_simplicissimum 0.950

>OTU_18
GAAATGCGATAACTAATGTGAATTGCAGAATTCAGTGAATCATCGAGTCTTTGAACGCACAT
TGCGCCCTCTGGTATTCCGGAGGGCATGCCTGTCCGAGCGTCATTGCTGCCCTCAAGCCCGG
CTTGtgtgtTGGGCCCCGTCCcccccGCCGGggggACGGGCCCGAAAGGCAGCGGCGGCACC
GCGTCCGGTCCTCGAGCGTATGGGGCTTCGTCACCCGCTCTAGTAGGCCCGGCCGGCGCCAG
CCGACCcccAACCTTTAATTATCTCAGGTTGACCTCGGATCAGGTAGGGATACCCGCTGAAC
TTAA
k__Fungi;p__Ascomycota;c__Eurotiomycetes;o__Eurotiales;f__Tric
hocomaceae;g__Penicillium;s__Penicillium_citrinum 1.000

>OTU_19
GAAATGCGATACCTAGTGTGAATTGCAGCCATCGTGAATCATCGAGTTCTTGAACGCACATT
GCGCCCCTCGGCATTCCGGggggCATGCCTGTTTGAGCGTCGTTTCCATCTTGCGCGTGCGC
AGAGTTGGgggAGCGGAGCGGACGACGTGTAAAGAGCGTCGGAGCTGCGACTCGCCTGAAAG
GGAGCGAAGCTGGCCGAGCGAACTAGACTTtttttCAGGGACGCTTGGCGGCCGAGAGCGAG
TGTTGCGAGACAACAAaaaGCTCGACCTCAAATCAGGTAGGAATACCCGCTGAACTTAAGCA
TATCAATAAGCGGAGGATGAGTCCGTAGGTGAACCTGCGGAGGA
k__Fungi;p__Ascomycota;c__Saccharomycetes;o__Saccharomycetales
;f__Incertae_sedis;g__Candida;s__Pichia_kudriavzevii 1.000

>OTU_20
GAAATGCGATAAGTAATGTGAATTGCAGAATTCAGTGAATCATCGAATCTTTGAACGCACAT
TGCGCCCCTTGGTATTCCGGggggCATGCCTGTTCGAGCGTCATTTCAACCCTCAAGCTCTG
CTTGGTATTGGGCACCGTCCTTTGCGGGCgcgcCTCAAAGACCTCGGCGGTGGCGTCTTGCC
TCAAGCGTAGTAGAATACACCTCGCTTCGGAGCGTAAGGCGTCGCCCGCCGGACGAACCTTC
TGAACTTTTCTCAAGGTTGACCTCGGATCAGGTAGGGATACCCGCTGAACTTAAGCATATCA
ATAAGCGGAGGAGCATATCAATAAGCGGAGGAGCATATCAATAAGCGGAGGA
K__Fungi;p__Ascomycota;c__Dothideomycetes;o__Botryosphaeriales
;f__Botryosphaeriaceae;g__Macrophomina;s__Macrophomina_phaseol
ina

>OTU_21
TAACTGCGATAAGTAGCGTGAATTGCAGACGCTTTGAACGTTAAACTTTCGAACGCACATTG
CGCCGTAGGAGTTCTACCCTGCGGCACATCTGGTTGAGGGTCGTGATCAAaaaCTGCCCGAA
TGCAGCAGTACAGATAGCTGGCGAACCATGATGAAAGTCGTGATTTGCCATACGACGCTATA
GAGGACAATTCTCCACGTGAgagagTTGCCTGCCACACGGATGATCTACTAGTTGACGCAGT
AGATCCCGGTCCCAGGTATCGTATTATGCCGAGGGATGCGCGGGGATACAGCTAGTGAgaga
ATCTGTGCGGCGCTATGAGCTATTCGATTTCCCGACCTCAACTCAGGTGTGATTACCCGCTG
AACTTAAGCATATCAATAAGCGGAGGAGCATATCAATAAGCGGAGGAATCCGTAGGTGAACC
TGCG
Pratylenchus goodeyi

>OTU_22
GAAATGCGATACTTGGTGTGAATTGCAGAATCCCGTGAACCATCGAGTCTTTGAACGCAAGT
TGCGCCCCAAGCCTTCTGGCCGAGGGCACGTCTGCCTGGGTGTCACAAATCGTCGTCCcccc
ATCCTCTCGAGGATATGGGACGGAAGCTGATCTCCCGTgtgtTACCGCACGCGGTTGGCCAA
AATCCGAGCTAAGGACGTCAGGAGCGTCTTGACATGCGGTGGTGAATTTAATTCTCGTCATA
TAGTCAGACGTTCCGGTCCAAAAGCTCTTGATGACCCAAAGTCCTCAACGCGACCCCAGGTC
AGGCGGGATCACCCGCTGAGTTTAAGCATATCAATAAGCGGAGGAGCATATCAATAAGCGGA
GGAGCATATCAATAAGCGGAGGA
Brassica rapa

>OTU_23
GAAATGCGATACTTGGTGTGAATTGCAGAATCCCGTGAACCATCGAGTCTTTGAACGCAAGT
TGCGCCCCAAGCCTTCTGGCCGAGGGCACGTCTGCCTGGGTGTCACAAATCGTCGTCCCTCA
CCATCCTTTGCTGATGCGGGACGGAAGCTGGTCTCCCGTgtgtTACCGCACGCGGTTGGCCT
AAATCCGAGCCAAGGACGCCTGGAGCGTACCGACATGCGGTGGTGAACTTGATCCATTACAT
TTTATCGGTCGCTCTTGTCCGGAAGCTGTAGATGACCCAAAGTCCATATAGCGACCCCAGGT
CAGGCGGGATTACCCGCTGAGTTTAAGCATATCAATAAGCGGAGGAATCCGTAGGTGAACCT
GCG
Arabidopsis thaliana

>OTU_24
GAAATGCGATAAGTAATGTGAATTGCAGAATTCAGTGAATCATCGAATCTTTGAACGCACAT
TGCGCCCGCCAGTATTCTGGCGGGCATGCCTGTTCGAGCGTCATTACAACCCTCAGGCCccc
GGGCCTGGCGTTGGGGATCGGCGGAAGCCcccTGCGGGCACAACGCCGTCCcccAAATACAG
TGGCGGTCCCGCCGCAGCTTCCATTGCGTAGTAGCTAACACCTCGCAACTGGAGAGCGGCGC
GGCCACGCCGTAAAACACCCAACTTCTGAATGTTGACCTCGAATCAGGTAGGAATACCCGCT
GAACTTAAGCATATCAATAAGCGGAGGAATCCGTAGGTGAACCTGCGGAGCATATCAATAAG
CGGAGGA
k__Fungi;p__Ascomycota;c__Sordariomycetes;o__Hypocreales;f__Ne
ctriaceae;g__Fusarium;s__Fusarium solani 1.000

>OTU_25
GAAATGCGATAAGTAATGTGAATTGCAGATACAGTGAATCATCGAATCTTTGAACGCAAATG

GCACTCTATGGTATTCCGTAGAGTACGTCTGTTTGAGCGTCGCGAACATCTCCATAATTAGT
TtttttttAAATTGATTGTGGGTTTTGAGGTTGTCATATAAACAATGACTCCCTTTAAAATA
ATTAGTGATGACCTTATGAATGGGTTAATACTGtgtgtTATAATGGATTACATCCATCACCA
GTCAGagagTAATCTCGCCTTAGTAATTTGTAGTGATTGCTTCTAACTGCCATTGGCAAACA
AACTGATCAAATCGACCTCAAATCAGATGGGATTACCCGCTGAACTTAAGCATATCAATAAG
CGGAGGAGCATATCAATAAGCGGAGGAGTCCGTAGGTGAACCTGCG
k__Fungi;p__Basidiomycota;c__Wallemiomycetes;o__Wallemiales;f__Wallemiaceae;g__Wallemia 1.000

>OTU_26
AAAATGCGATAAGTAATGTGAATTGCAGAATTCAGTGAATCATCGAATCTTTGAACGCACAT
TGCGCCCGCCAGTATTCTGGCGGGCATGCCTGTTCGAGCGTCATTTCAACCCTCAAGCACAG
CTTGGTGTTGGGACTCGCGTTAATTCGCGTTCCTCAAATTGATTGGCGGTCACGTCGAGCTT
CCATAGCGTAGTAGTAAAACCCTCGTTACTGGTAATCGTCGCGGCCACGCCGTTAAACCCCA
ACTTCTGAATGTTGACCTCGGATCAGGTAGGAATACCCGCTGAACTTAAGCATATCAATAAG
CGGAGGAATCCGTAGGTGAACCTGCGGA
k__Fungi;p__Ascomycota;c__Sordariomycetes;o__Hypocreales;f__Nectriaceae;g__Fusarium;s__Fusarium_oxysporum 0.980

>OTU_27
GAAATACGATAAGTAATGTGAATTGCAGAATTCCGTGAATCATCGAATCTTTGAACGCACAT
TGCGCCcccTGGCATTCCGGggggCATGCCTGTCCGAGCGTCATTTCTGCCCTCAAGCACGG
CTTGtgtgtTGGGTGTGGTCCccccGGGGACCTGCCCGAAAGGCAGCGGCGACGTCCGTCTG
GTCCTCGAGCGTATGGGGCTCTGTCACTCGCTCGGGAAGGACCTGCGGgggTTGGTCACCAC
CATGTTtttACCACGGTTGACCTCGGATCAGGTAGGAGTTACCCGCTGAACTTAAGCATATC
AATAAGCGGAGGAAaaaTGGCAGCATCGATGAAGAAC
k__Fungi;p__Ascomycota;c__Eurotiomycetes;o__Eurotiales;f__Trichocomaceae;g__Penicillium;s__Penicillium_pinophilum 0.970

>OTU_28
TTGCACTCTATGGTTtttCTATAGAGTACGCCTGCTTCAGTATCATCACAAACCCAcacaTA
ACATTTGTTTATGTGGTGATGGGTCGCATCGCTGTTTTATTACAGTGAGCACCTAAAATGtg
tgtgATTTTCTGTCTGGCTTGCTAGGCAGGAATATTACGCTGGTCTCAGGATCTTttttttG
GTTCGCCCAGGAAGTAAAGTACAAGAGTATAATCCAGTAACTTTCAAACTATGATCTGAAGT
CAGGTGGGATTACCCGCTGAACTTAAGCATATCAATAAGCGGAGGAGCATATCAATAAGCGG
AGGAGCATATCAATAAGCGGAGGA
k__Fungi;p__Zygomycota;c__Incertae_sedis;o__Mucorales;f__Mucoraceae;g__Rhizopus;s__Rhizopus_oryzae 1.000

>OTU_29
GAAATGCGATAAGTAATGTGAATTGCAGAATTCAGTGAATCATCGAATCTTTGAACGCACAT
TGCGCCcccTGGTATTCCGGggggCATGCCTGTTCGAGCGTCATTTCACCACTCAAGCCTCG
CTTGGTATTGGGCAACGCGGTCCGCCGCGTGCCTCAAATCGACCGGCTGGGTCTTCTGTCCC
CTAAGCGTTGTGGAAACTATTCGCTAAAGGGTGCTCGGGAGGCTACGCCGTAAAACAAACCC

ATTTCTAAGGTTGACCTCGGATCAGGTAGGGATACCCGCTGAACTTAAGCATATCAATAAGC
GGAGGAGTCCGTAGGTGAACCTGCGGAC
k__Fungi;p__Ascomycota;c__Dothideomycetes;o__Capnodiales;f__My
cosphaerellaceae;g__Cladosporium;s__Cladosporium_tenuissimum
0.870

>OTU_30
GAAATGCGATAACTAATGTGAATTGCAGAATTCAGTGAATCATCGAGTCTTTGAACGCACAT
TGCGCCcccTGGTATTCCGGggggCATGCCTGTCCGAGCGTCATTTCTCCCCTCCAGCCCCG
CTGGTTGTTGGGCCGCGCCcccccGGgggCGGGCCTCGAgagaAACGGCGGCACCGTCCGGT
CCTCGAGCGTATGGGGCTCTGTCACCCGCTCTATGGGCCCGGCCGGGGCTTGCCTCGACCcc
cAATCTTCTCAGATTGACCTCGGATCAGGTAGGGATACCCGCTGAACTTAAGCATATCAATA
AGCGGAGGAATCCGTAGGTGAACCTGCGGCATCGATGAAGAAC
k__Fungi;p__Ascomycota;c__Eurotiomycetes;o__Eurotiales;f__Tric
hocomaceae;g__Aspergillus;s__Aspergillus_aculeatus 0.810

>OTU_31
GAAATGCGATAAGTAATGTGAATTGCAGATACAGTGAATCATCGAATCTTTGAACGCAAATG
GCACTCTATGGTATTCCGTAGAGTACGTCTGTTTGAGCGTCGCGAACATCTCCACAATTAGT
TtttttAATTAGTTGAGGGTTTTGAGGTTGTCATATAAACAGTGACTCCCTTTAAAATAATT
AGTGATGACCTTATGAATGGGTTAATACTGtgtgtTATAATGGATTACATCCATCACCAGTC
AGagagTAATCTCGCCTTAGTAATTTGTAGTGATTGCTTCTAACTGCCAATTGGCAACAACC
TGATCAAATCGACCTCAAATCAGATGGGATTACCCGCTGAACTTAAGCATATCAATAAGCGG
AGGA
k__Fungi;p__Basidiomycota;c__Wallemiomycetes;o__Wallemiales;f_
_Wallemiaceae;g__Wallemia;s__Wallemia_sebi   0.980

>OTU_32
AAAATGCGATACTTGTTGTGAATTGCAGAATCCCGTGAACCATCGAGTCTTTGAACGCAAGT
TGCGCCCCAAGCCTTCTGGCCGAGGGCTCTTCTGCCTGGGTGTCACAAATCGTCGTCCcccc
ATCCCCTCGAGGATATGGGATGGAAGCTGATCTCCCGTgtgtTACCGCATGCGGTTGGCTAA
AATCCAAGCTCAATTCTCGTCATATAGTCAGACTTTCCGGTCCAAAAGCTCTAGATGACCCA
AAGTCCTCAACGCGACCCCAGGTCAGGCGGGATCACCCGCTGAGTTTAGGCATATCAATAAG
CGGAGGAATCCGTAGGTGAACCTGCG
Brassica napus

>OTU_33
GAACTGCGATAAGTAATGTGAATTGCAGAATTCAGTGAATCATCGAGTCTTTGAACGCACAT
TGCGCCcccTGGCATTCCGGggggCATGCCTGTCCGAGCGTCATTGCTGCCCATCAAGCCCG
GCTTGtgtgtTGGGTCGTCGTCCcccccGGgggACGGGCCCGAAAGGCAGCGGCGGCACCGT
GTCCGGTCCTCGAGCGTATGGGGCTTTGTCACCCGCTCGACTAGGGCCGGCCGGGCGCCAGC
CGACGTCTCCAACCATTtttCTTCAGGTTGACCTCGGATCAGGTAGGGATACCCGCTGAACT
TAA
k__Fungi;p__Ascomycota;c__Eurotiomycetes;o__Eurotiales;f__Tric

hocomaceae;g__Aspergillus;s__Aspergillus_versicolor   1.000

>OTU_34
AAAATGCGATAAGTAATGTGAATTGCAGAATTCAGTGAATCATCGAATCTTTGAACGCACAT
TGCGCCCGCCAGTATTCTGGCGGGCATGCCTGTTCGAGCGTCATTTCAACCCTCAAGCCCAG
CTTGGTGTTGGGAGCTGCAGTCCTGCTGCACTCCCCAAATACATTGGCGGTCACGTCGAGCT
TCCATAGCGTAGTAATTTACACATCGTTACTGGTAATCGTCGCGGCCACGCCGTTAAACCCC
AACTTCTGAATGTTGACCTCGGATCAGGTAGGAATACCCGCTGAACTTAAGCATATCAATAA
GCGGAGGA
k__Fungi;p__Ascomycota;c__Sordariomycetes;o__Hypocreales;f__Ne
ctriaceae;g__Fusarium;s__Fusarium_graminearum   0.990

>OTU_35
AAACTGCGATAAGTAGCGTGAATTGCAGACGCTTTGAACGTTAAACTTTCGAACGCACATTG
CGCCGTAGGAGTCCTACCCTGCGGCACATCTGGTTGAGGGTCGTGATCAAaaaCAGCAGGAC
TATGGCTGTAATAGAAATAGCTGGTGAATCACGAGTTCGGCCTACCGCCTCTGGCCGAACGT
GTTTTGCCATAATGCTAGAAGAGGACAATCTCACGAGAGTGTGATTGCCTACCACACGGATG
ATCTATTAGCTGAAGCAATAGGTCTCGGTCACCGGGTATCATTCTTTTAtataCCATGTGGC
GTCTGAGATACAGCTAGTGGGCTTCATACGTGCCTGGTATTGAGAGTATTCGCTCTCCCGAC
CTCAACTCAGGTGTGATTACCCGCTGAACTTAA
Pratylenchus goodeyi

>OTU_36
AAATCGCGATATGTAATGTGATCTGCCTATAGTGAATCATCAAATCTTTGAACGCATCTTGC
ACCTTATGGTATTCCATAAGGTACGTCTGTTTCAGTACCACTAATAAATCtctctctATCCT
TGATGATAGAAaaaaaaGAGATAAATTATTACTGGTCCTGGTGATTCTTttttttttttAT
TAAaaaGAACCACTCTCGGCCTAAATATAAGGCTCGACTTttttttACCAGATCTTGCATCT
AGTAAaaaCCTAGTCGGCTTTAATAGATTtttATTTTCTATTAAGTTTATAGCCATTCTTAT
ATTttttAAAATCTTGGCCTGAAATCAGATGGGACTACCCGCTGAACTTAAGCATATCAATA
AGCGGAGGA
k__Fungi;p__Zygomycota;c__Incertae_sedis;o__Mucorales;f__Cunni
nghamellaceae;g__Cunninghamella;s__Cunninghamella_bertholletia
e 1.000

>OTU_37
GAATTGCGATAAGTAATGTGAATTGCAGAATTCAGTGAATCATCGAATCTTTGAACGCAGCT
TGCGCTctctGGTATTCCGGAGAGCATGCCTGTTTCAGTGTCATGAAATCTCAACCACTAGG
GTTTCCTAATGGATTGGATTTGGGCGTCTGCGATCTCTGATCGCTCGCCTTAAAAGAGTTAG
CAAGTTTGACATTAATGTCTGGTGTAATAAGTTTCACTGGGTCCATTGTGTTGAAGCGTGCT
TCTAATCGTCCGCAAGGACAATTACTTTGACTCTGGCCTGAAATCAGGTAGGACTACCCGCT
GAACTTAAGCATATCAATAAGCGGAGGAATCCGTAGGTGAACCTGCGGA
k__Fungi;p__Basidiomycota;c__Tremellomycetes;o__Tremellales;f_
_Trichosporonaceae;g__Trichosporon;s__Trichosporon_asahii 0.900

>OTU_38
AAACTGCGATAAGTAGCGTGAATTGCAGACGCTTTGAACGTTAAACTTTCGAACGCACATTG
CGCCGTAGGAGTCCTACCCTGCGGCACATCTGGTTGAGGGTCGTGATCAAaaaCAGCAGGAC
TATGGCTGTAATAGAAATAGCTGGTGAATCACGAGTTCGGCCTACCGCCTCTGGCCGAACGT
GTTTTGCCATAATGCTAGAAGAGGACAATCACTCTGCTCGAGTGATTGCCTACCACACGGAT
GATCTATTAGCTGAAGCAATAGGTCTCGGTCACCGGGTATCATACTTAACTAtataCTCTGT
GGCGTCTGAGATACAGCTAGTGGGCCTCATACGTGCCTGGTATTGAGAGTATTCGCTCTCCC
GACCTCAACTCAGGTGTGATTACCCGCTGAACTTAAGCATATCAATAAGCGGAGGA
Pratylenchus goodeyi

>OTU_39
GAAATGCGATAAGTAGTGTGAATTGCAGAATTCAGTGAATCATCGAATCTTTGAACGCACAT
TGCGCCCTTTGGTATTCCAAAGGGCATGCCTGTTCGAGCGTCATTTGTACCCTCAAGCTTTG
CTTGGTGTTGGGCGTCTTGTCTCTAGCTTTGCTGGAGACTCGCCTTAAAGTAATTGGCAGCC
GGCCTACTGGTTTCGGAGCGCAGCACAAGTCGCACTctctATCAGCAAAGGTCTAGCATCCA
TTAAGCCTTtttttCAACTTTTGACCTCGGATCAGGTAGGGATACCCGCTGAACTTAAGCAT
ATCAATAAGCGGAGGAGCATATCAATAAGCGGAGGA
k__Fungi;p__Ascomycota;c__Dothideomycetes;o__Pleosporales;f__P
leosporaceae;g__Alternaria;s__Alternaria_brassicae 0.990

>OTU_40
GAAATGCGATAAGTAATGTGAATTGCAGAATTCAGTGAATCATCGAATCTTTGAACGCACCT
TGCGCTCCTTGGTATTCCGAGGAGCATGCCTGTTTGAGTGTCATGAAACCCTCAAACCCAAG
TTTTGGATTTCGATCCATGCTTGAGTTTGGATTTGGATGTTTGCCGGTGATGAACCGACTCA
TCTTAAAAGTATTAGCTTGGATCTGTCTATATGACTGGTTTGACTTGGCATAATAAGTATTT
TGCTGAGGACATCTTCGGATGGCCAGGACCTAGACTACTGTCTGCTAACTAAACCATCACTT
TAAGTGCATCTTTGGATGTTACTCATTGTGTAACTTTGACATCTGGCCTCAAATCAAGTAGG
ACTACCCGCTGAACTTAAGCATATCAATAAGCGGAGGAATCCGTAGGTGAACCTGCGGA
k__Fungi;p__Basidiomycota;c__Tremellomycetes;o__Filobasidiales
;f__Filobasidiaceae;g__Cryptococcus;s__Cryptococcus_magnus
0.960

>OTU_41
GAAATGCGATAAGTAATGTGAATTGCAGATACAGTGAATCATCGAATCTTTGAACGCAAATG
GCACTCTATGGTATTCCGTAGAGTACGTCTGTTTGAGCGTCGCGAACATCTCTATAATTAGT
TttttttAAATTGATTATGGGTTTTGAGGTTGTCATGTATAATGACTCCCTTTAAAATGATT
AGTGATGACCTTATGAATGGGTTAATACTGtgtgtTATAATGGATTACATCCATCACCAGTC
AGagagTAATCTCGCCTTAGTAATTTGTAGTGATTGCTTCTAACTGCCAATTtttGGCAAAC
AACCTGATCAAATCGACCTCAAATCAGATGGGATTACCCGCTGAACTTAA
k__Fungi;p__Basidiomycota;c__Wallemiomycetes;o__Wallemiales;f_
_Wallemiaceae;g__Wallemia;s__Wallemia_sebi 0.920

>OTU_42
GAAATGCGATAAGTAATGTGAATTGCAGAATTCAGTGAATCATCGAATCTTTGAACGCACAT

TGCGCCCGCCAGCATTCTGGCGGGCATGCCTGTTCGAGCGTCATTTCAACCCTCGAGCCCGA
CTCTTTTGAGGACGGCTCGGCGTTGGgggACGGCAGACTCCccccGGCATCTCGCCGGGGAC
GCCGCCcccGAAATCTAGTGGCGGCCCTCCGAGGCGACCTCTGCGTAGTAACTTGTTCTCGC
ACCGGAAAGCTCAGAGCGGCCACGCCGTAAAACCCCAACTTttttCAGGTTGACCTCGAATC
AGGTAGGACTACCCGCTGAACTTAAGCATATCAATAAGCGGAGGAGCATATCAATAAGCGGA
GGA
k__Fungi;p__Ascomycota;c__Leotiomycetes;o__Helotiales;f__Incertae_sedis;g__Gloeotinia;s__Gloeotinia_temulenta 1.000

>OTU_43
GAAATGCGATAATTAATGTGAATTGCAGAATTCAGTGAATCATCGAGTCTTTGAACGCACAT
TGCGCCcccTGGTATTCCGGggggCATGCCTGTCCGAGCGTCATTGCTGCCCTCAAGCACGG
CTTGtgtgtTGGGCTTCCGTCCCTGGTAACGGGGACGGGCCCAAAAGGCAGTGGCGGCACCA
TGTCTGGTCCTCGAGCGTATGGGGCTTTGTCACCCGCTCCCGTAGGTCCAGCTGGCAGCTAG
CCTCGCAACCAATCTTtttAACCAGGTTGACCTCGGATCAGGTAGGGATACCCGCTGAACTT
AAGCATATCAATAAGCGGAGGAATCCGTAGGTGAACCTGCGGAACTctctcCCCTCCTTGTG
ATGGGGAAAGTACTTCTTCATCT
k__Fungi;p__Ascomycota;c__Eurotiomycetes;o__Eurotiales;f__Trichocomaceae;g__Eurotium;s__Eurotium_niveoglaucum 1.000

>OTU_44
GAAATGCGATACGTAGTGTGAATTGCAGAATTCAGTGAATCATCGAATCTTTGAACGCACAT
TGCGCCCTTTGGTATTCCAAAGGGCATGCCTGTTCGAGCGTCATTTGTACCCTCAAGCTTTG
CTTGGTGTTGGGCGTTTTGTCTTTGCATCAAAGACTCGCCTTAAAACGATTGGCAGCCGGCC
TACTGGTTTCGGAGCGCAGCACATTtttGCGCTTGCAATCAGCAAGAGGTCGGCAATCCATC
AAGTCCATTTCTCACTTTTGACCTCGGATCAGGTAGGGATACCCGCTGAACTTAAGCATATC
AATAAGCGGAGGA
k__Fungi;p__Ascomycota;c__Dothideomycetes;o__Pleosporales;f__Pleosporaceae;g__Curvularia;s__Curvularia_sp_LH3 0.990

>OTU_45
AAAATGCGATAAGTAATGTGAATTGCAGAATTCAGTGAATCATCGAATCTTTGAACGCACAT
TGCGCCCGCCAGTATTCTGGCGGGCATGCCTGTTCGAGCGTCATTTCAACCCTCAAGCTCAG
CTTGGTGTTGGGACTCGCGGTAACCCGCGTTCCCCAAATCGATTGGCGGTCACGTCGAGCTT
CCATAGCGTAGTAATCATACACCTCGTTACTGGTAATCGTCGCGGCCACGCCGTAAAACCCC
AACTTCTGAATGTTGACCTCGGATCAGGTAGGAATACCCGCTGAACTTAAGCATATCAATAA
GCGGAGGAATCCGTAGGTGAACCTGCGGA
k__Fungi;p__Ascomycota;c__Sordariomycetes;o__Hypocreales;f__Nectriaceae;g__Fusarium;s__Fusarium_chlamydosporum   0.880

>OTU_46
GAAATGCGATAAGTAATGTGAATTGCAGAATTCAGTGAATCATCGAATCTTTGAACGCACAT
TGCGCCCATTAGTATTCTAGTGGGCATGCCTGTTCGAGCGTCATTTCAACCCCTAAGCACAG
CTTATTGTTGGGACTCTACGGCTTCGTAGTTCCCCAAAGACATTGGCGGAGTGGCAGCAGTC

CTCTGAGCGTAGTAATTCTTTATCTCGCTTTTGTTAGGCGCTGCCcccccGGCCGTAAAACC
cccAATTttttCTGGTTGACCTCGGATCAGGTAGGAATACCCGCTGAACTTAAGCATATCAA
TAAGCGGAGGA
k__Fungi;p__Ascomycota;c__Sordariomycetes;o__Trichosphaeriales
;f__Incertae_sedis;g__Khuskia;s__Nigrospora_oryzae 1.000

>OTU_47
GAAATGCGATACGTAATGTGAATTGCAGAATTCAGTGAATCATCGAGTCTTTGAACGCACAT
TGCGCCCTCTGGTATTCCGGggggCATGCCTGTCCGAGCGTCATTGCTGCCCTCAAGCACGG
CTTGtgtgtTGGGCCcccGTCCCGGTTAAGCATTAGCCCCGGGACGGCCCGAAAGGCAGCG
GCGGCACCGTGTCCGGTCCTCGAGCGTATGGGGCTCTGTCACCCGCTCGTGTAGGTCCGGCC
GGCGGCCAGCCCGACCCTTTAACCAATCTTTTAACCAGGTTGACCTCGGATCAGGTAGGGAT
ACCCGCTGAACTTAA
k__Fungi;p__Ascomycota;c__Eurotiomycetes;o__Eurotiales;f__Tric
hocomaceae;g__Aspergillus;s__Aspergillus_vitricola 0.950

>OTU_48
GAAATGCGATAAGTAGTGTGAATTGCAGAATTCAGTGAATCATCGAATCTTTGAACGCACAT
TGCGCCCCTTGGTATTCCATGGGGCATGCCTGTTCGAGCGTCATTTGTACCTTCAAGCTTTG
CTTGGTGTTGGGTGTTTGTCTCGCCTTTGCGTGTAGACTCGCCTTAAAACAATTGGCAGCCG
GCGTATTGATTTCGGAGCGCAGTACATCTCGCGCTTTGCACTCATAACGACGACGTCCAAAA
GTACATTtttACACTCTTGACCTCGGATCAGGTAGGGATACCCGCTGAACTTAAGCATATCA
ATAAGCGGAGGAGTCCGTAGGTGAACCTGCGGAGCATATCAATAAGCGGAGGAATACGTAGG
TGAACCTGCGGA
k__Fungi;p__Ascomycota;c__Dothideomycetes;o__Pleosporales
;f__Pezizaceae;g__Leptosphaerulina;s__Leptosphaerulina_arac
hidicola 0.860

>OTU_49
GAAATGCGATAAGTAATGTGAATTGCAGAATTCAGTGAATCATCGAATCTTTGAACGCACAT
TGCGCCCGCCAGTATTCTGGCGGGCATGCCTGTTCGAGCGTCATTTCAACCCTCAAGCCCCA
GCGGCTTGGTGTTGGGGACCGGCCCCGGCCGCCccccAAATGCAGTGGCGACCTCGCCGCAG
CCTCCCCTGCGTAGTAGCACAACTCGCACCGGAGCGCGGAGACGGTCACGCCGTAAAACGCC
CAACTTCTCAGAGTTGACCTCGGATCAGGTAGGAATACCCGCTGAACTTAAGCATATCAATA
AGCGGAGGAGTCCGTAGGTGAACCTGCGGCATATCAATAAGCGGAGGATGAGTCCGTAGGTG
AACCTGCG
k__Fungi;p__Ascomycota;c__Sordariomycetes;o__Hypocreales;f__Op
hiocordycipitaceae;g__Tolypocladium;s__Tolypocladium_inflatum
0.830

>OTU_50
CAAATGCGATAAGTAATGTGAATTGCAGAGTTCCGTGAATCATCGAATTtttGAACGCATAT
TGCGCCTTGTGGTAATCCGCAAGGCATGCCTGTCTGAGCGTCATTACATCATTCAAGTAAAA
GTTttttttttttACTTGGTCTTGGAATTTGACTTtttACCAACTTGTTGGTAGTTATGTCT

GTTCTGTAATGTATGATTGCAGTCTATTGACACTTTTAGTTGTAGTACATACCATCAACTTT
AAAAGCCTCTAGACCTGGTCACCTTTACCATCAATCGCTTGATTGAAATTTTCTTGTAAAGT
TTGACCTCAGATCAGGTAGGACTACCCGCTGAACTTAAGCATATCAATAAGCGGAGGA
Uncultured fungus

>OTU_51
GAAATGCGATAAGTAATGTGAATTGCAGAATTCCGTGAATCATCGAATCTTTGAACGCACAT
TGCGCCcccTGGCATTCCGGggggCATGCCTGTCCGAGCGTCATTTCTGCCCTCCAGCACGG
CTGGGTGTTGGGTGCTGTCCccccGGGGACACGCCCCAAAAGCAGTGGCGGCGCCGCGTCGG
GTCCTCGAGCGTATGGGGCTCTGTCACCCGCTCGGGAGGGACTCGGTCGGCGCTGGTCTTCC
TCTAGGCGACCCTTCGGGGCTCGTCTCCTCCGGTTGACCTCGGATCAGGTAGGGCTACCCGC
TGAACTTAA
k__Fungi;p__Ascomycota;c__Eurotiomycetes;o__Eurotiales;f__Tric
hocomaceae;g__Penicillium;s__Penicillium_radicum 0.800

>OTU_52
GAAATGCGATAAGTAATGTGAATTGCAGAATTCCGTGAATCATCGAATCTTTGAACGCACAT
TGCGCCccccGGAATGCCTGTCCGAGCGTCATTTCTGCCCTCAAGCACGGCTTGtgtgtTGG
GTGTGGTCCccccGGGGACCTGCCCGAAAGGCAGCGGCGACGTCCGTCTGGTCCTCGAGCGT
ATGGGGCTCTGTCACTCGCTCGGGAAGGACCTGCGGgggTTGGTCACCACCATGTTtttACC
ACGGTTGACCTCGGATCAGGTAGGAGTTACCCGCTGAACTTAAGCATATCAATAAGCGGAGG
AGCATATCAATAAGCGGAGGA
k__Fungi;p__Ascomycota;c__Eurotiomycetes;o__Eurotiales;f__Tric
hocomaceae;g__Penicillium 0.980

>OTU_53
GAAATGCGATACGTAATGTGAATTGCAGAATTCAGTGAATCATCGAGTCTTTGAACGCACAT
TGCGCCcccTGGTATTCCGGggggCATGCCTGTCCGAGCGTCATTGCTGCCCTCAAGCACGG
CTTGtgtgtTGGGCCcccGTCCccccccAAGGGGACGGGCCCGAAAGGCAGCGGCGGCACCG
TGTCCGGTCCTCGAGCGTATGGGAAGCAACTTtttttGTCACCCGCTCCTGTAGGTCCGGCC
GGCGGCCTGCCCAACCCCAACCTTTTACTTTAACCAGGTTGACCTCGGATCAGGTAGGGATA
CCCGCTGAACTTAA
k__Fungi;p__Ascomycota;c__Eurotiomycetes;o__Eurotiales;f__Tric
hocomaceae;g__Aspergillus;s__Aspergillus_penicillioides 1.000

>OTU_54
GAAATGCGATAAGTAATGTGAATTGCAGAATTCAGTGAATCATCGAGTCTTTGAACGCACAT
TGCGCCcccTGGTATTCCGGggggCATGCCTGTCCGAGCGTCATTGCACCCCTCAAGCCCGG
CTTGTCCTTGGGTTGTGTCCCTCCGTTCCGGggggACAGGCCTCAAATGCAATGGCGGCACC
GCGTCCGGTCCTCGAGCGTATGGGGCTTTGTCACCCGCTTTGTAGGCCGGCCGGTCGCTTGC
CCGTCAAGCACAACTTCTTATGTTGACCTCGGATCAGGTAGGGATACCCGCTGAACTTAAGC
ATATCAATAAGCGGAGGAATCCGTAGGTGAACCTGCG
k__Fungi;p__Ascomycota;c__Eurotiomycetes;o__Eurotiales;f__Tric
hocomaceae;g__Penicillium;s__Penicillium_georgiense 1.000

>OTU_55
TAACTGCGATAAGTAGCGTGAATTGCAGACGCTTTGAACGTTAAACTTTCGAACGCACATTG
CGCCGTAGGAGTTCTACCCTGCGGCACATCTGGTTGAGGGTCGTGATCAAaaaCTGCCCGAA
TGCAGCAGTATAGAGATAGCTGGCGAACCATGATGAAAGTCGTGATTTGCCATACGACGCTA
TAGAGGACAATTCTCCACGTGAgagagTTGCCTGCCACACGGATGATCTACTAGTTGACGCA
GTAGATCCCGGTCCCAGGTATTATGCACGAAACGTATTATGCCGAGGGATGCGCGGGGATAC
AGCTAGTGGGAGAATCCGTGCGAGCTATATGAGCTATTCGATTTCCCGACCTCAACTCAGGT
GTGATTACCCGCTGAACTTAA
Pratylenchus goodeyi

>OTU_56
AAAGTGCGATAATTATTGCGACTTGCATTCATAGTGAATCATCGAGTTCTTGAACGCATCTT
GCGCCTAGTAGTCAATCTACTAGGCACGGTTGTTTCAGTATCATCCTAACCAATCAACCTTT
CTTGGGTTGGAACTGGACTTTTGACAGCATTCAGTTGCTCTCATGGTCTTAAATACCAAGTC
TTTGTGCATTCACATTGTGAGTGTACGGGTGGAAATTttttAAACAGTGAGCAATGAGCGTC
TTGGGTTGTGGGAACCcccAAGTGTCAGTCTTTGAGCGTCGACCTGGTTTCCTACTTGAAAC
GACTTGAGAATGCACTAGCATTCAAGACAACGAAAATCTCACCATCTTGATCTGAAATCAAC
TGAGACTACCCGCTGAACTTAAGCATATCAATAAGCGGAGGAATCAGTAGGTGAACCTGCGG
A
k__Fungi;p__Zygomycota;c__Incertae_sedis;o__Mucorales;f__Licht
heimiaceae;g__Lichtheimia;s__Lichtheimia_hyalospora   1.000

>OTU_57
GAAATGCGATAAGTAATGTGAATTGCAGAATTCAGTGAATCATCGAATCTTTGAACGCACAT
TGCGCCCGCCAGAATACTGGCGGGCATGCCTGTCTGAGCGTCATTTCAACCCTCATGCCCCT
AGGGCGTGGTGTTGGGGATCGGCCAAAGCCCGCGAGGGACGGCCGGCCCCTAAATCTAGTGG
CGGACCCGTCGTGGCCTCCTCTGCGAAGTAGTGATATTCCGCATCGGAGAGCGATGAGCCCC
TGCCGTTAAACCcccAACTTTCTAAGGTTGACCTCAGATCAGGTAGGAATACCCGCTGAACT
TAAGCATATCAATAAGCGGAGGAGTCCGTAGGTGAACCTGCGGAAGGACGTTG
k__Fungi;p__Ascomycota;c__Sordariomycetes;o__Hypocreales;f__Bi
onectriaceae;g__Bionectria;s__Clonostachys_rosea   0.990

>OTU_58
TGGATGCTCCGGTGAGCGGCGGTGAACCGAAAGCCATCGACGGTACGCTGTCAGTGATGGTG
GGCGGCGACAAGGCTATTTTCGACAAATACTATGATTTGATGAAAGCGATGGCGGGTTCCGT
GGTGCATACCGGGGAAATCGGTGCAGGTAACGTCACCAAACTGGCAAATCAGGTCATTGTGG
CGCTGAATATTGCCGCGATGTCAGAAGCGTTAACGCTGGCAACTAAAGCGGGCGTTAACCCG
GACCTGGTTTATCAGGCAATTCGCGGTGGACTGGCGGGCAGTACCGTGCTGGATGCCAAAGC
GCCGATGGTGATGGACCGCAACTTCAAGCCGGGCTTCCGTATTGATCT
Escherichia coli

>OTU_59
AGATCAATACGGAAGCCCGGCTTGAAGTTGCGGTCCATCACCATCGGCGCTTTGGCATCCAG

CACGGTACTGCCCGCCAGTCCACCGCGAATTGCCTGATAAACCAGGTCCGGGTTAACGCCCG
CTTTAGTTGCCAGCGTTAACGCTTCTGACATCGCGGCAATATTCAGCGCCACAATGACCTGA
TTTGCCAGTTTGGTGACGTTACCTGCACCGATTTCCCCGGTATGCACCACGGAACCCGCCAT
CGCTTTCATCAAATCATAGTATTTGTCGAAAATAGCCTTGTCGCCGCCCACCATCACTGACA
GCGTACCGTCGATGGCTTTCGGTTCACCGCCGCTCACCGGAGCATCCA
Escherichia coli

>OTU_60
GAAATGCGATACTTGGTGTGAATTGCAGAATCCCGTGAACCATCGAGTCTTTGAACGCAAGT
TGCGCCCGAAGCCATCAGGCCGAGGGCACGTCTGCCTGGGCGTCACGCACCGTTGCCCTCCC
AACATCACCGTCCTTCGAGGCTACGTTTTGTTGCGAAGGCGCATATTGGCTTCCCGTGAGCT
TTGCCTCGTGGTTGGCCCAAAATCTTAGTCGTTGGCGACCCGTGCCGCGACGACGGTGGTTG
TCGAAACTTCGGTGCCCTGTCGTgtgtgCGGGTCGTTCGTGACGCAAGGACCCAACGCATTC
GAATGTGATGCCTCTCGATGCGACCCCAGGTCAGGCGGGGCTACCCGCTGAGTTTAA
Hippophae tibetana

>OTU_61
GAAATGCGATAATTAATGTGAATTGCAGAATTCAGTGAATCATCGAGTCTTTGAACGCACAT
TGCACCcccTGGTATTCCGGggggTATGCCTGTCCGAGCGTCATTGCTGCCCTCAAGCACGG
CTTGtgtgtTGGGTCGTCGTCCccccccAGGgggACGGGCCCGAAAGGCAGCGGCGGCACCG
CGTCCGGTCCTCGAGCGTATGGGGCTTTGTCACCCGCTCTTGTAGGCCCGGCCGGCTGCTGG
CCGACGCTGAAAAGCAACCAACTATTtttCCAGGTTGACCTCGGATCAGGTAGGGATACCCG
CTGAACTTAA
k__Fungi;p__Ascomycota;c__Eurotiomycetes;o__Eurotiales;f__Tric
hocomaceae;g__Aspergillus;s__Aspergillus_ochraceus 0.940

>OTU_62
GAAATGCGAAAAGCAATGCGAATTGCAGAATTCCGCGAGTCATCAGATCTTCGAACGCACCT
CGCGCCAATCGGATCTCCGATCGGCATGTTTGATTCAGTGTCTTGTTGAGACTTGCATATCT
CATTGTGATTGACTCTTCGCTACTTGTAGAGAAGTGTCTTTCATGAAAATAGATTGCGCATA
CCGCAACGATGCAGTAGTCAGTTAACGCTGATACTCATCGGTGAAATGGTATGATTACGTAA
TGTTGGAAACAACAATACATAAAACTCATCCACATGCATCTGAATCAAGCAAGAACACCCGC
TGAACTTAAGCATATCAATAAGCGGAGGATGAGTCCGTAGGTGAACCTGCG
Cryptocaryon irritans

>OTU_63
GAAATGCGATAAGTAATATGAATTGCAGATTTTCGTGAATCATCGAATCTTTGAACGCACAT
TGCACCTTGTGGTATTCCACAAGGTATGCCTGTTTGAGCGTCATTTCTCCCTCAACCcccGC
GGGTTGGCGTTGAATGGCACGAGCTCTTAGTCAGTCCATTCGAAAAGTATTtttCTTGAGTT
GTTATTTTCTAATTTAGTAGTGACAACTACACTAAaaaCACATTTTCCTCAAATCAGGTAGG
ACTACCCGCTGAACTTAAGCATATCAATAAGCGGAGGAGCATATCAATAAGCGGAGGAGTCC
GTAGGTGAACCTGCGGA
k__Fungi;p__Ascomycota;c__Saccharomycetes;o__Saccharomycetales
;f__Pichiaceae;g__Pichia;s__Pichia_burtonii 1.000

>OTU_64
GAAATGCGATAAGTAATGTGAATTGCAGATACAGTGAATCATCGAATCTTTGAACGCAAATG
GCACTCTATGGTATTCCGTAGAGTACGTCTGTTTGAGCGTCGCGAACATCTCCATAATTGGT
TtttttAAATTGATTGTGGGTTTTGAGGTTGTCATATAAACAATGACTCCCTTTAAAATAAT
TAGTGATGACCTTATGAATGGGTTAATACTGtgtgtTATAATGGATTACATCCATCACCAGG
CAGagagTAATCTCGCCTTAGTAATTTGTAGTGATTGCTTCTAACTGCCATTGGCAAACAAA
CTGATCAAATCGACCTCAAATCAGATGGGATTACCCGCTGAACTTAAGCATATCAATAAGCG
GAGGAGTCCGTAGGTGAACCTGCGGA
k__Fungi;p__Basidiomycota;c__Wallemiomycetes;o__Wallemiales;f__Wallemiaceae;g__Wallemia 1.000

>OTU_65
TAGATACGTTAATCGGTGTTATATGCTATAGCCATAAGACATCACTCGACTATTCTAATGCG
CAATGCAGCTAGTCCCTTAAAGACTAGTTACATCTGATTGAGAGTATCATTAAGTTTGTTCA
ATAATTATTATTGAATGAGAACTGCATTTGACTTATAGTGATGACTATACTTGTATAGACGT
CATGTAAAAGCGATACTAAGGCACGCTCGTGTGCTTTGGATCGCTATTGTGTTTCACTATGA
AACTAATTTCAAaaaGCAAaaaaGCAATTtttAGCTATGTTTGAAGTAATCAAATGAATACT
CAGAACACGTATCTCAGTCAGGTGAGAATACCCGCTGAACTTAAGCATATCAATAAGCGGAG
GA
Acarapis woodi

>OTU_66
GAAACGCGATATGTAATGTGAATTGCAGAATTCAGTGAATCATCGAATCTTTGAACGCACAT
GGCGCCTTCCAGTATCCTGGGAGGCATGCCTGTCCGAGCGTCGTTTCAACCCTCGAGCCccc
GTGGCCCGGCGTTGGGGACCTGCCCAGGCAGTCCCCGAAAACCAGTGGCGGACCCGACGGGC
CCTTCCTTTGCGTAGTAACATCTGCCTCGCATCGGGAGCCcccGGGCTATCCGGCCTCTAAA
CCccccTCAAGCCCGCTCCGGCGGCACCAAGGTTGACCTCGGATCAGGTAGGAATACCCGCT
GAACTTAAGCATATCAATAAGCGGAGGA
k__Fungi;p__Ascomycota;c__Sordariomycetes;o__Incertae_sedis;f__Plectosphaerellaceae;g__Gibellulopsis;s__Gibellulopsis_nigrescens 1.000

>OTU_67
GAAATGCGATAAGTAATGTGAATTGCAGAATTCAGTGAATCATCGAATCTTTGAACGCACAT
TGCGCCCGCCAGTATTCTGGCGGGCATGCCTGTCCGAGCGTCATTTCAACCCTCGAACCCCT
CCGGggggTCGGCGTTGGGGATCGGCCCTTTACGGGGCCGGCCCCGAAATACAGTGGCGGTC
TCGCCGCAGCCTCTCCTGCGCAGTAGTTTGCACACTCGCATCGGGAGCGCGGCGCGTCCACA
GCCGTTAAACACCCCAAACTTCTGAAATGTTGACCTCGGATCAGGTAGGAATACCCGCTGAA
CTTAAGCATATCAATAAGCGGAGGATCCGTAGGTGAACCTGCG
k__Fungi;p__Ascomycota;c__Sordariomycetes;o__Hypocreales;f__Hypocreaceae;g__Trichoderma;s__Trichoderma_virens 0.970

>OTU_68

GAAATGCGATAAGTAATGTGAATTGCAGAATTCAGTGAATCATCGAGTCTTTGAACGCACAT
TGCGCCcccTGGTATTCCGGggggCATGCCTGTCCGAGCGTCATTGCTGCCCTCAAGCACGG
CTTGtgtgtTGGGCTCTCGCCccccGCTTCCGGggggCGGGCCCGAAAGGCAGCGGCGGCAC
CGCGTCCGGTCCTCGAGCGTATGGGGCTTCGTCACCCGCTCTGTAGGCCCGGCCGGCGCCCG
CCGGCGAACACCATCAATCTTAACCAGGTTGACCTCGGATCAGGTAGGGATACCCGCTGAAC
TTAA
k__Fungi;p__Ascomycota;c__Eurotiomycetes;o__Eurotiales;f__Tric
hocomaceae;g__Penicillium;s__Penicillium_ oxalicum 0.980

>OTU_69
GAAATGAGATAATTAATGTGAATTGCAGAATTCAGTGAATCATCGAGTCTTTGAACGCACAT
TGCGCCcccTGGTATTCCGGggggCATGCCTGTCCGAGCGTCATTGCTGCCCTCAAGCACGG
CTTGtgtgtTGGGCTTCCGTCCCTGGTAACGGGGACGGGCCCAAAAGGCAGTGGCGGCACCA
TGTCTGGTCCTCGAGCGTATGGGGCTTTGTCACCCGCTCCCGTAGGTCCAGCTGGCAGCTAG
CCTCGCAACCAATCTTtttAACCAGGTTGACCTCGGATCAGGTAGGGATACCCGCTGAACTT
AAGCATATCAATAAGCGGAGGAATCAGTCCTTAAGCATATCAATAAGCGGAGGA
k__Fungi;p__Ascomycota;c__Eurotiomycetes;o__Eurotiales;f__Tric
hocomaceae;g__Eurotium;s__Eurotium_niveoglaucum 1.000

>OTU_70
GAAATGCGATAAGTAATGTGAATTGCAGAATTCAGTGAATCATCGAATCTTTGAACGCACAT
TGCGCCCGCCAGTATTCTGGCGGGCATGCCTGTTCGAGCGTCATTTCAACCCTCAGGCCccc
AGTGCCTGGTGTTGGGGATCGGCCCAGCCTTCTCGCAAGGCCGCCGGCCCCGAAATCTAGTG
GCGGTCTCGCTGTAGTCCTCCTCTGCGTAGTAGCACAACCTCGCAGTTGGAACGCGGCGGTG
GCCATGCCGTTAAACACCCCACTTCTGAAAGTTGACCTCGGATCAGGTAGGAATACCCGCTG
AACTTAAGCATATCAATAAGCGGAGGA
k__Fungi;p__Ascomycota;c__Sordariomycetes;o__Hypocreales;f__In
certae_sedis;g__Myrothecium;s__Myrothecium_verrucaria 0.980

>OTU_71
GAAATGCGATACGTAGTGTGAATTGCAGAATTCAGTGAATCATCGAATCTTTGAACGCACAT
TGCGCCCTTTGGTATTCCAAAGGGCATGCCTGTTCGAGCGTCATTTGTACCCTCAAGCTTTG
CTTGGTGTTGGGCGTTtttGTCTTTGGCCCGCCAAAGACTCGCCTTAAAATGATTGGCAGCC
GGCCTACTGGTTTCGCAGCGCAGCACATTtttGCGCTTGCAATCAGCAAAAGAGGACGGCAA
TCCATCAAGACTCCTTCTCACGTTTGACCTCGGATCAGGTAGGGATACCCGCTGAACTTAA
k__Fungi;p__Ascomycota;c__Dothideomycetes;o__Pleosporales;f__P
leosporaceae;g__Bipolaris;s__Bipolaris_tetramera   0.920

>OTU_72
GAAATGCGATAATTAATGTGAATTGCAGAATTCAGTGAATCATCGAGTCTTTGAACGCACAT
TGCGCCcccTGGTATTCCGGggggCATGCCTGTCCGAGCGTCATTGCTGCCCTCAAGCACGG
CTTGtgtgtTGGGCCcccGTCCccccTCCAAAGGGGACGGGCCCGAAAGGCAGCGGCGGCAC
CGTGTCCGGTCCTCGAGCGTATGGGAAGCAACTTtttGTCACCCGCTCCTGTAGGTCCGGCC
GGCGGCCTGCCCAACCCCAATCAATCTTTTAACCAGGTTGACCTCGGATCAGGTAGGGATAC

CCGCTGAACTTAA
k__Fungi;p__Ascomycota;c__Eurotiomycetes;o__Eurotiales;f__Tric
hocomaceae;g__Aspergillus;s__Aspergillus_penicillioides 0.980

>OTU_73
GAAATGCGATACGTAATGTGAATTGCAGAATTCAGTGAATCATCGAGTCTTTGAACGCACAT
TGCGCCcccTGGTATTCCGGggggCATGCCTGTCCGAGCGTCATTGCTGCCCTCAAGCACGG
CTTGtgtgtTGGGCCcccGTCCCCTCCCTAGGGAGGGGACGGGCCCGAAAGGCAGCGGCGGC
ACCGTGTCCGGTCCTCGAGCGTATGGGAAGCACAATCTTttttGTCAACCCGCTCCTGTAGG
TCCGGCCGGCGGCCTGCCCCAATCAACCCATTGTTtttAACCAGGTTGACCTCGGATCAGGT
AGGGATACCCGCTGAACTTAA
k__Fungi;p__Ascomycota;c__Eurotiomycetes;o__Eurotiales;f__Tric
hocomaceae;g__Aspergillus;s__Aspergillus_penicillioides 1.000

>OTU_74
GAAATGCGATAAGTAATGTGAATTGCAGAATTCAGTGAATCATCGAATCTTTGAACGCACAT
TGCGCCCCTTGGTATTCCGAGGGGCATGCCTGTTCGAGCGTCATTACACCACTCAAGCACTG
CTTGGTATTGGGCATCGTCCGTCGAAAGGCGGGCGTGCCTCGAAGACCTCGGCGGGGTTTCT
CCAACTTCGGGCGTAGTAGAGTTAAATCGAACGTCTTATAAGCTTGGTGGGACTCCATTGCC
GTTAAACCTTTTATTTTCTAGGTTGACCTCGGATCAGGTAGGGATACCCGCTGAACTTAA
k__Fungi;p__Ascomycota;c__Dothideomycetes;o__Dothideales;f__Do
thideaceae;g__Dothidea;s__Dothidea_sp_CanS_64 0.960

>OTU_75
GAAATGCGATAACTAATGTGAATTGCAGAATTCAGTGAATCATCGAGTCTTTGAACGCACAT
TGCGCCcccTGGTATTCCGGggggCATGCCTGTCCGAGCGTCATTACTGCCCTCAAGCCCGG
CTTGTATTGGGTCCTCGTCCcccTCCCCGGgggACGGGCCCGAAAGGCAGCGGCGGCACCGC
GTCCGGTCCTCGAGCGTATGGGGCTTTGTCACCCGCTCTGTAGGCCCGGCCGGCGCCAGCCC
ACGCAACACCTTttttttCAGGTTGACCTCGGATCAGGTAGGGATACCCGCTGAACTTAA
k__Fungi;p__Ascomycota;c__Eurotiomycetes;o__Eurotiales;f__Tric
hocomaceae;g__Aspergillus;s__Aspergillus_flavipes 1.000

>OTU_76
GTCGTCGCATCGATGAAGAACGCAGCTTGCACTCTATGGTTtttCTATAGAGTACGCCTGCT
TCAGTATCATCACAAACCCAcacaTAACATTTGTTTATGTGGTGATGGGTCGCATCGCTGTT
TTATTACAGTGAGCACCTAAAATGtgtgtgATTTTCTGTCTGGCTTGCTAGGCAGGAATATT
ACGCTGGTCTCAGGATCTTtttttttGGTTCGCCCAGGAAGTAAAGTACAAGAGTATAATCC
AGTAACTTTCAAACTATGATCTGAAGTCAGGTGGGATTACCCGCTGAACTTAAGCATATCAA
TAAGCGGAGGAGTCCGTAGGTGAACCTGCGCATATCAATAAGCGGAGGA
k__Fungi;p__Zygomycota;c__Incertae_sedis;o__Mucorales;f__Mucor
aceae;g__Rhizopus;s__Rhizopus_oryzae 1.000

>OTU_77
GAAATGCGATAAGTAATGTGAATTGCAGAATTCAGTGAATCATCGAATCTTTGAACGCACAT

TGCGCCCCTTGGTATTCCGAGGGGCATGCCTGTTCGAGCGTCATTACACCACTCAAGCTATG
CTTGGTATTGGGCGTCGTCCTTAGTTGGGCgcgcCTTAAAGACCTCGGCGAGGCCACTCCGG
CTTTAGGCGTAGTAGAATTTATTCGAACGTCTGTCAAAGGAGAGGAACTCTGCCGACTGAAA
CCTTTATTtttCTAGGTTGACCTCGGATCAGGTAGGGATACCCGCTGAACTTAAGCATATCA
ATAAGCGGAGGA
k__Fungi;p__Ascomycota 1.000

>OTU_78
AAAATGCGATAAGTAATGTGAATTGCAGAATTCAGTGAATCATCGAATCTTTGAACGCACAT
TGCGCCCGCCAGTATTCTGGCGGGCATGCCTGTTCGAGCGTCATTTCAACCCTCAAGCCccc
GGGTTTGGTGTTGGGGATCGGCGAGCCCTTGCGGCAAGCCGGCCCCGAAATCTAGTGGCGGT
CTCGCTGCAGCTTCCATTGCGTAGTAGTAAAACCCTCGCAACTGGTACGCGGCGCGGCCAAG
CCGTTAAACCcccAACTTCTGAATGTTGACCTCGGATCAGGTAGGAATACCCGCTGAACTTA
A
k__Fungi;p__Ascomycota;c__Sordariomycetes;o__Hypocreales;f__Ne
ctriaceae;g__Fusarium;s__Fusarium_proliferatum 0.880

>OTU_79
GAAATGCGATACGTAATGTGAATTGCAGAATTCAGTGAATCATCGAGTCTTTGAACGCACAT
TGCGCCCTCTGGTATTCCGGggggCATGCCTGTCCGAGCGTCATTGCTGCCCTCAAGCACGG
CTTGtgtgtTGGGCCcccGTCCCGGTTCTTTAAGCCGGGACGGGCCCGAAAGGCAGCGGCGG
CACCGTGTCCGGTCCTCGAGTGTATGGGGCTCTGTCACCCACTCGTGTAGGTCCGGCCGGCG
GCCAGCCTCTTCAACCAAACTTTTAACCAGGTTGACCTCGGATCAGGTAGGGATACCCGCTG
AACTTAA
k__Fungi;p__Ascomycota;c__Eurotiomycetes;o__Eurotiales;f__Tric
hocomaceae;g__Aspergillus;s__Aspergillus_restrictus 1.000

>OTU_80
AAAGTGCGATAACTAGTGTGAATTGCATATTCGTGAATCATCGAGTCTTTGAACGCAGCTTG
CACTCTATGGATCTTCTATAGAGTACGCTTGCTTCAGTATCATAACCAACCCAcacaTAAAA
TTTATTTTATGTGGTGATGGACAAATTCGGTTAGATTTAATTATTATACCGATTGTCTAAAA
TACAGCCTCTTTGTAATTTTCATTAAATTACGAACTACCTAGCCATCGTGCTTttttGGTCC
AACCAAaaaaCATTTAATCTAGGGGTTCTGCCAGCCAGCAGATATTTTAATGCTCTTTAACT
ATGATCTGAAGTCAAGTGGGACTACCCGCTGAACTTAA
k__Fungi;p__Zygomycota;c__Incertae_sedis;o__Mucorales;f__Mucor
aceae;g__Rhizopus;s__Rhizopus_microsporus 1.000

>OTU_81
AAAATGCGATAAGTAATGTGAATTGCAGAATTCAGTGAATCATCGAATCTTTGAACGCACAT
TGCGCCCGCCAGTATTCTGGCGGGCATGCCTGTCTGAGCGTCATTTCAACCCTCGGGACCCC
GTTCGCGGGACCCGGCGTTGGGGATCAGCCCGAAGCCcccAGGGCGGGCGGCTGGCCCCGAA
ATCTAGTGGCGGTCCccccAGCGACCTCCTCTGCGCAGTAGTAACTCACCTCGCAGCTGGAC
AGCGGGAGGGCCACGCCGTAAAACCcccAACTTCTCAAAGGTTGACCTCAGATCAGGTAGGA
ATACCCGCTGAACTTAA

k__Fungi;p__Ascomycota;c__Sordariomycetes;o__Hypocreales;f__Incertae_sedis;g__Acremonium;s__Acremonium_sp_OUCMBI101028 1.000

>OTU_82
GAAATGCGATACTTGGTGTGAATTGCAGAATCCCGTGAACCATCGAGTCTTTGAACGCAAGT
TGCGCCCCAAGCCTTTGGCCGAGGGCACGTCTGCCTGGGTGTCACAAATCGTCGTCCccccc
AATCCTTTTGGATAAGGGACGGAAGTTGGTCTCCCGTGTGATACCGCATGCGGTTGGCCGAA
ATCCGAGCTTAGGACGTCAGGAGCGTCTCGACATGCGGTGGTGAAAATCTCCCTCATTATGT
CGGTCGTTCCTGTCCATAAGCTCTCGATGACCCAAAGTCCTCAACGCGACCCCAGGTCAGGC
GGGATCACCCGCTGAGTTTAA
Braya glabella

>OTU_83
AAAATGCGATACTTGGTGTGAATTGCAGAATCCCGTGAATCATCGAGTTtttGAACGCAAGT
TGCGCCCGAAGCCTTTCGGCCGAGGGCACGCCTGCCTGGGTGTCACGCAATTGTCGCCccccA
ACCCTTTCGATACATCGAGAGGggggCGGATTATGGCCTCCCGTGCGCCTCGTGCATGCGGT
TGGCCTAAaaaTTGAGTCCCCGGCGACTATCGCCACGGCAATCGGTGGTTGTAAGACTctct
GAAACTGCCGTGCgcgcTCGTCTGCCAAGAGGGAACCCTCGAGACCCCGATGCTGCCGTAAA
GGGCATGCTCCAACTGCGACCCCAGGTCAGGCGGGATTACCCGCTGAGTTTAA
Ricinus communis

>OTU_84
GAAATGCGATAAGTAATGTGAATTGCAGAATTCAGTGAATCATCGAATCTTTGAACGCACAT
TGCGCCCATTAGTATTCTAGTGGGCATGCCTGTTCGAGCGTCATTACAACCCCTAAGCACAG
CTTATTGTTGGGCGTCTACGTCTGTAGTGCCTCAAAGACATTGGCGGAGCGGCAGCAGTCCT
CTGAGCGTAGTAATTCTTTATCTCGCTTCTGTTAGGCGCTGCCcccccGGCCGTAAAACCcc
cAATTttttCTGGTTGACCTCGGATCAGGTAGGAATACCCGCTGAACTTAA
k__Fungi;p__Ascomycota;c__Sordariomycetes;o__Trichosphaeriales;f__ Trichosphaeriaceae;g__Nigrospora;s__Nigrospora_oryzae 1.000

>OTU_85
GAAATGCGATAACTAATGTGAATTGCAGAATTCAGTGAATCATCGAGTCTTTGAACGCACAT
TGCGCCCTCTGGTATTCCGGAGGGCATGCCTGTCCGAGCGTCATTGCTGCCCTCAAGCACGG
CTTGtgtgtTGGGCCcccGTCCccccTCTGCCGGggggACGGGCCCGAAAGGCAGCGGCGG
CACCGCGTCCGGTCCTCGAGCGTATGGGGCTTCGTCACCCGCTCTTGTAGGCCCGGCCGGCG
CCAGCCGACCCCAACCCTAAATTtttttCAGGTTGACCTCGGATCAGGTAGGGATACCCGCT
GAACTTAA
k__Fungi;p__Ascomycota;c__Eurotiomycetes;o__Eurotiales;f__Trichocomaceae;g__Penicillium;s__Penicillium_meleagrinum 0.970

>OTU_86
GAAATGCGATAAGTAATGTGAATTGCAGAATTCAGTGAATCATCGAATCTTTGAACGCACAT
TGCGCCCTTTGGCATTCCGAAGGGCATGCCCGTTCGAGCGTCATTACACCAATCAAGCCTGG
CTTGGTATTGGGCGTCGGGGACCCTCCcccGCGCCcccAATTCGTCGGCTGGACGGTTCGAA

TCTCAGCGTTGTGGTCAATCAATTCGCTGGCGACGACGACCGGACGCGCCGTTAAACCTCAC
TACAGGTTGACCTCGGATCGGGTAGGGATACCCGCTGAACTTAA
k__Fungi;p__Ascomycota;c__Dothideomycetes;o__Capnodiales;f__Teratosphaeriaceae;g__Devriesia;s__Devriesia_sp_NG_p52 1.000

>OTU_87
AAAGTGCGATAATTATTGCGACTTGCATTCATAGTGAATCATCGAGTTCTTGAACGCATCTT
GCGCCTAGTAGTCAATCTACTAGGCACAGTTGTTTCAGTATCTGCATCCACCAATCAACACG
ACTTGCTTGTGTTGGAACTGGGCTTACTTTTGATGGCATTTAGTTGCTGTCATGGCCTTAAA
TGTATTTAGTCCTAGGTGTTAACTTGTTAATGCCGGATGGAGACTCTAGAGTGCCTTAGAAG
CAGCTTGGTTAGTGAGTTCATAATTCCAAGTGTTAGTCTTTTATTGAACTGGGTTTCTAGTC
TATGGGACATGGAGTCGTAAGACTCTTAAACAACAAACTCACATTTAGATCTGAAATCAACT
GAGACCACCCGCTGAACTTAA
k__Fungi;p__Zygomycota;c__Incertae_sedis;o__Mucorales;f__Lichtheimiaceae;g__Lichtheimia;s__Lichtheimia_corymbifera 1.000

>OTU_88
GAAATGCGATAAGTAATGTGAATTGCAGAATTCAGTGAATCATCGAATCTTTGAACGCACAT
TGCGCCCGCCAGTATTCTGGCGGGCATGCCTGTCCGAGCGTCATTTCAACCCTCGAACCCCT
CCGGgggATCGGCGTTGGGGATCGGGACCCCTCACACGGGTGCCGGCCCCTAAATACAGTGG
CGGTCTCGCCGCAGCCTCTCCTGCGCAGTAGTTTGCACAACTCGCACCGGGAGCGCGGCGCG
TCCACGTCCGTAAAACACCCAACTTTCTGAAATGTTGACCTCGGATCAGGTAGGAATACCCG
CTGAACTTAA
k__Fungi;p__Ascomycota;c__Sordariomycetes;o__Hypocreales;f__Hypocreaceae;g__Trichoderma;s__Trichoderma_asperellum   1.000

>OTU_89
GAAATGCGATAAGTAATGTGAATTGCAGAATTCAGTGAATCATCGAATCTTTGAACGCACCT
TGCGCCTTTTGGTATTCCGAAAGGCATGCCTGTTTGAGTGTCATGAAATCTCAATCCcccTG
GGTTTATGATCTGGGTCGGACTTGGATATGGGCGTCTGCCGGTCACACGGCTCGCCTCAAAT
GACTTAGTGGATCtctctGCATCCGTGACAGACGTAATAAGTTTCGTCTTGTCCCTTGCTTA
TGAGTCTGCTCATAACCTGCCATCGCGCACTTTAGACTCTGACCTCAAATCAGGTAGGACTA
CCCGCTGAACTTAA
k__Fungi;p__Basidiomycota;c__Tremellomycetes;o__Filobasidiales;f__Filobasidiaceae;g__Cryptococcus;s__Cryptococcus_laurentii   0.990

>OTU_90
GAAATGCGATAACTAATGTGAATTGCAGAATTCAGTGAATCATCGAGTCTTTGAACGCACAT
TGCGCCccccTGGTATTCCGGggggCATGCCTGTCCGAGCGTCATTACTGCCCTCAAGCCCGG
CTTGTATTGGGTCCTCGTCCccccGGGGACGGGCCCGAAAGGCAGCGGCGGCACCGCGTCCG
GTCCTCGAGCGTATGGGGCTTTGTCACCCGCTCTGTAGGCCCGGCCGGCGCCAGCCCACGCA
TAACCTTTATTttttCAGGTTGACCTCGGATCAGGTAGGGATACCCGCTGAACTTAA
k__Fungi;p__Ascomycota;c__Eurotiomycetes;o__Eurotiales;f__Tric

hocomaceae;g__Aspergillus;s__Aspergillus_flavipes 0.820

>OTU_91
GAAATGCGATACTTGGTGTGAATTGCAGAATCCCATGAACCATCGATTCCTTGAACGCAAGT
TGCGCCCGAAGCCCTTAGGCTGAGGGCACGCCTGCCTGGGTGTCACCAAAAGTCGCCccccc
GTCTCGCCTGTCCCAGGGCACGGGGAGGgggCAAACGTTGGCCTCCCGGGAGCCCCTGGCTC
GCGGTTGGTTCAAaaaGACGGGCTCTTGGTGGAGAGCGGCACCGCGGCAGATAGTGGTCGAG
AACAACACTCGTGGCCAGTTGCGCGTGCCTCTCCcccGGTTCAAGGCACGGCGACCTCAGGT
CAGGCGGGGCTACCCGCTGAGTTTAA
Arachis hypogaea

>OTU_92
GAATTGCGTTAAGAATCACGAATTACAGATATTATGAGTGATATGTTTTCGATTGCATATTG
CATCGTTGGGCACTTGCCCATCGGTATACCCAACTCAGGGTgtgtATCACTAGTTGAAAGAC
ACAATGCACTTGTTGGTTGTTTCTAAAATTTGGAGGTTTGAAGTACTAGTTACTTCAAACCT
ACTGTTACGGTGTGGCTGAATTGTAGAAATGAAGGAGAATGAGGTTGAAAATACTTGGTGGT
TGTTGGAAGTTTAATGAATTtttGAGCTTTCGGTGACTATTCGGTTtttCGACTATTtttAC
ACAGCCCTATACacacCAGCCAATCGTTtttGGTTTAATCCACCTGAGTCGGATATGATCAC
CCGCCGAACTTAA
Aphelenchoides

>OTU_93
GAAATGCGATAAGTAATGTGAATTGCAGAATTCAGTGAATCATCGAATCTTTGAACGCACAT
TGCGCCCGCCAGTATTCTGGCGGGCATGCCTGTTCGAGCGTCATTTCAACCATCAAGCCccc
GGCTTGTGTTGGGGACCTGCGGCTGCCCGCAGGCCCTGAAAACCAGTGGCGGGCTCGCTGTC
ACACCGAGCGTAGTAGCATACATCTCGCTCAGGGCGTGCTGCGGGTTCCGGCCGTTAAACGA
CCTCTATAACCCAAGGTTGACCTCGGATCAGGTAGGAAGACCCGCTGAACTTAA
k__Fungi;p__Ascomycota;c__Sordariomycetes;o__Sordariales;f__Ch
aetomiaceae 0.910

>OTU_94
GAAATGCGATAAGTAATGTGAATTGCAGAATTCAGTGAATCATCGAATCTTTGAACGCACAT
TGCGCCCGCTAGCACTCTAGCGGGCATGCCTGTCCGAGCGTCATTTCAACCCTCAGACCccc
TTCGGggggACTGGCGTTGGGGATCGGCCCGCCTCAGTGCGGTGCCGTCCcccAAATACAGT
GGCGGTCTCGCTGCAGCCTCCCCTGCGTAGTAGCACACCTCGCATCGGAGAGCGGTGAGGCC
ACGCCGTGAAACCccccACTTCTGAACGTTGACCTCGGATCAGGTAGGAATACCCGCTGAAC
TTAA
k__Fungi;p__Ascomycota;c__Sordariomycetes;o__Hypocreales;f__In
certae_sedis;g__Stachybotrys;s__Stachybotrys_longispora 0.960

>OTU_95
GAAATGCGATAAGTAATGTGAATTGCAGAATTCAGTGAATCATCGAATCTTTGAACGCACAT
TGCGCTCCTTGGTATTCCGGGGAGCATGCCTGTTTGAGAATCAGTAAATTCATCGAACACGG
TTTGGTTTCATACCTGCTGTGATCGGATATGGGCCTCTTTGCGCCTGGCGCAATGGCCTGAA

ATTCAATGTCACGGAATCATTGGACACCAGTCCGGTTTCAAGCGCATGAAAGGGTTAGTTCt
ctcGATTACGTCTTGTAGTATCGTTCTGGTTTTGAAGGACTTTGACTTCTGGGATTCAACTA
TCACTCCAGATGGCATTCTTTTAGGGTGCTTTCTGAACACTTGGTCTCAAATCAGGTAGGAA
AACCCGCTGAACTTAA
k__Fungi;p__Chytridiomycota;c__Chytridiomycetes;o__Rhizophlyct
idales;f__Rhizophlyctidaceae;g__Rhizophlyctis;s__Rhizophlyctis
_rosea 1.000

>OTU_96
GAAATGCGATACTTGGTGTGAATTGCAGAATCCCGTGAACCATCGAGTCTTTGAACGCAAGT
TGCGCCCGAAGCCCTTAGGCTGAGGGCACGCCTGCCTGGGTGTCACCAAAGGCGCCccccG
TCCCGCCCGTCCGAATGTTGGCGAATGTTGGCCTCCCGGGAGCCCCTGGCTCGCGGTTGGTT
CAAAGAGACGGGCTCTTGGTGGGGAGCGGCACCGCGGCAGATGGTGGTCGAGAACAACCCTC
GTGGCCAGTCGcgcgcgcCTCTCCcccGGTTCAAGGCACGGCGACCCGCGGGCGACGTGGAT
CGTCCCGAGCGCGACCTCAGGTCAGGCGGGGCTACCCGCTGAGTTTAA
Arachis hypogaea

>OTU_97
GAAATGCGATAAGTAGTGTGAATTGCAGAATTCAGTGAATCATCGAATCTTTGAACGCACAT
TGCGCCCTATGGTATTCCGTAGGGCATGCCTGTTCGAGCGTCATTCAACCCCTCAAGCTCTG
CTTGGTGTTGGGCGTCTGTCCCGCCTCACGGCGCGGACTCGCCTCAAATCTATTGGCGGCCG
GCACGTTGGCTTCGAGCGCAGCAGAAACGCGAACTCGAGGCCCGGCGGATCGGCTCCCAGAA
GCTACCccccATGAATTTGACCTCGGATCAGGTAGGGATACCCGCTGAACTTAA
k__Fungi;p__Ascomycota;c__Dothideomycetes;o__Pleosporales;f__S
porormiaceae;g__Preussia 1.000

>OTU_98
GAAGTGCGATAAGCAATGCGAATTGCAGAACCGTGAGTCATCAGATTtttGAACGCAACTGG
CGCTGGCTGGGTCTCCAGCCAGCATGCTTGTTTCAGTGTCTTGTTTTCTCCTCACCCAAACC
TTAATGCGAgagaTACCCTTCTCTTGCCAAGCACGAAAGCACTCTGCGCTCTGCGAGCTTCT
CCTTGACTAGCTCAGGgggTCGCACTCAATGCAGCCGTCACATTTCTCACAATGTGAACTCA
TTGGGAGCAGAGGCTGTCAGCTCACGCTGTCAGTGCTTAGTCACTTAAACTTTCTTTGCATC
TGAAATCAAGCAGGATCACCCGCTGAACTTAA
Uncultured fungus

>OTU_99
GAAATGCGATAAGTAATGTGAATTGCAGAATTCAGTGAATCATCGAATCTTTGAACGCACAT
TGCGCCCGCCAGCATTCTGGCGGGCATGCCTGTTCGAGCGTCATTTCAACCCTCAAGCTCAG
CTTGGTGTTGGGGCCCTACGCCTGGCGTAGTCCCCTAAAGGTAGTGGCGGACCCTCTCGGAG
CCTCCTTTGCGTAGTAACTAACGTCTCGCACTGGGATCCGGAGGGACCTCTCGCCGTAAAAC
CccсAATTtttCTTAGGTTGACCTCGGATCAGGTAGGAATACCCGCTGAACTTAA
k__Fungi;p__ Ascomycota;c__ Sordariomycetes;o__
Glomerellales;f__Glomerellaceae;g__Colletotrichum;s__Colletotr
ichum_chlorophyt 1.000

>OTU_100
ACATTTCCAACCTGCGTCGTAAACTGCCGGATCGTAAAGATGGTCACCCGTGGTTTAAAACC
TTGCGTGGTCGCGGCTATCTGATGGTTTCTGCTTCATGATAGGCAGCTTAACCGcgcgcATC
TTCGCCATCTTCTGGCTGACGCTGGCGCTGGTGTTGATGTTGGTTTTGATGTTACCCAAGCT
CGATTCACGCCAGATGACCGAGCTTCTGGATAGCGAACAGCGTCAGGGGCTGATGATTGAGC
A
Escherichia coli

>OTU_101
AAAATGCGAAAAGTCGTGTGACCTGCATACTTCGTGAATCATCAAATCTTTGAACGCAACTT
GCACCTGTCTGCCTCGTGTAAACAGGTATACTTGTTTGAGTATCGCATACATCTCACTACCT
GTAGGGAGAGGAGTGGATCTGGGCATGTCGATGGGATCGACTCGCCTGAAATGCAGTGCTGC
CAGCGCAGATGCCGATGGGATAACATCACATAGGTACTGTGGCAGAGGGTGGCATATTATTA
TTGTCATGGGGAAGACAACGCACATCGATCTCAAATCAAGTAAGGGTACCCGCTGAACTTAA
Uncultured fungus

>OTU_102
GAAATGCGATAAGTAATGTGAATTGCAGAATTCAGTGAATCATCGAATCTTTGAACGCACAT
TGCGCCCTATGGTATTCCGTAGGGCATGCCTGTTCGAGCGTCATTACacacCTCAAGCTCTG
CTTGGTATTGGGCGTCGTCCCCTAAACGGACGTGCCTCAAAGACCTCGGCGGTGGCGTCTTT
GCCTCAAGCGTAGTAATACTTTTATCTCGCTTTGGAGTCGAAGGCGTCGCCCGCCGGACGAA
ACCTTTATTATTTCTATCAAGGTTGACCTCGGATCAGGTAGGGATACCCGCTGAACTTAA
k__Fungi;p__Ascomycota;c__Dothideomycetes;o__Botryosphaeriales
;f__Botryosphaeriaceae;g__Aplosporella;s__Aplosporella_hesperi
dica 1.000

>OTU_103
GAAATGCGATAAGTAATGTGAATTGCAGAATTCAGTGAATCATCGAATCTTTGAACGCACAT
TGCGCCcccTGGTATTCCGGggggCATGCCTGTTCGAGCGTCATTTCACCACTCAAGCCTCG
CTTGGTATTGGGCGACGCGGTCCGCCGcgcgcCTCAAATCGACCGGCTGGGTCTTTCGTCCC
CTCAGCGTTGTGGAAACTATTCGCTAAAGGGTGCCGCGGGAGGCCACGCCGTAAAACAACCC
CATTTCTAAGGTTGACCTCGGATCAGGTAGGGATACCCGCTGAACTTAA
k__Fungi;p__Ascomycota;c__Dothideomycetes;o__Capnodiales;f__My
cosphaerellaceae;g__Cladosporium;s__Cladosporium_halotolerans
1.000

>OTU_104
GAAATGCGATACTTGCATCGAGTCTTTGAACGCAAGTTGCGCCCGAAGCCCTTAGGCTGAGG
GCACGCCTGCCTGGGTGTCACCAAAAGGCGCCccccccGTCTCGCCCGTCCCAGGGCACGGGG
AGGgggCGAACGTTGGCCTCCCGGGAGCCCCTGGCTCGCGGTTGGTTCAAAGAGACGGGCTC
TTGGTGGGGAGCGGCACCGCGGCAGATGGTGGTCGAGAACAACCCTCGTGGCCAGTCGcgcg
cgcCTCTCCccccGGTTCAAGGCACGGCGACCCGCGGGCGACGTGGATCGTCCCGAGCGCGAC
CTCAGGTCAGGCGGGGCTACCCGCTGAGTTTAA

Arachis hypogaea

>OTU_105
TAGATACGTTAATCGGTGTTATATGCTATAGCCATAAGACATCACTCGACTATTCTAATGCG
CAATGCAGCTAGTCCCTTAAAGACTAGTTACATCTGATTGAGAGTATCATTAAGTTTGTTCA
ATAATTAATTATTATTGAATGAGAACTGCATTTGACTTATAGTGATGACTAGTGTAGTCGTC
ATGTAAAAGCGATACTAAGGCACGCTCGTGTGCTTTGGATCGCTATTGTGTTTCACTATGAA
ACTAATTTCAAaaaGCAAaaaaGCAATTtttAGCTATGTTTGAAGTAATCAAATGAATACTC
AGAACACGTATCTCAGTCAGGTGAGAATACCCGCTGAACTTAA
Acarapis

>OTU_106
GAAATGCGATAAGTAATGTGAATTGCAGAATTCCGTGAATCATCGAATCTTTGAACGCACAT
TGCGCCcccTGGCATTCCGGggggCATGCCTGTCCGAGCGTCATTTCTGCCCTCAAGCACGG
CTTGtgtgtTGGGTCGCCGTCCcccTCTCCGGggggACGGGCCCGAAAGGCAGCGGCGACGT
CCGTCTGGTCCTCGAGCGTATGGGGCTCTGTCACTCGCTCGGGAAGGACCTGCGGgggTTGG
TCACCACCATGTTtttACCACGGTTGACCTCGGATCAGGTAGGAGTTACCCGCTGAACTTAA
k__Fungi;p__Ascomycota;c__Eurotiomycetes;o__Eurotiales;f__Tric
hocomaceae;g__Penicillium;s__Penicillium_pinophilum   0.950

>OTU_107
GAAATGCGATAAGTAATGTGAATTGCAGAATTCCGTGAATCATCGAATCTTTGAACGCACAT
TGCGCCcccTGGCATTCCGGggggCATGCCTGTCCGAGCGTCATTTCTGCCCTCAAGCACGG
CTTGtgtgtTGGGTGTGGTCCccccGGGGACCTGCCCGAAAGGCAGCGGCGACGTCCGTCTG
GTCCTCGAGCGTATGGGGCTCTGTCACTCGCTCGGGAAGGACCTGCGGgggTTGGTCACCAC
CATGTTtttACCACGGTTGACCTCGGATCAGGTAGGAGTTACCCGCTGAACTTAAGCATATC
AATAAGCGGAGGAGCATATCAATAAGCGGAGGAGCATATCAATAAGCGGAGGA
k__Fungi;p__Ascomycota;c__Eurotiomycetes;o__Eurotiales;f__Tric
hocomaceae;g__Penicillium;s__Penicillium_pinophilum   1.000

>OTU_108
GAAATGCGATAAGTAATGTGAATTGCAGAATTCAGTGAATCATCGAATCTTTGAACGCAACT
TGCGCCCTCTGGTATTCCGGAGGGCATGCCTGTTTGAGTGTCATGTAGACTCGATCCCTCGG
GTTTCCGAGGAGATTGGATTTGGGTGTCTGCCTCTTCTGGCTCACCTCAAAAGACTTAGCGG
GATAGCACCGTAGTCGACGTAATAAGTTTCGTCGGTAAAAGTGTGATGTCTGCTCACAATCG
CCCCTGGGCACTTTTGACTCTGACCTCAAATCAGGTAGGACTACCCGCTGAACTTAA
k__Fungi;p__Basidiomycota;c__Tremellomycetes;o__Filobasidiales
;f__Filobasidiaceae;g__Cryptococcus;s__Cryptococcus_ podzolicus
0.830

>OTU_109
GAAATGCGATAAGTAGTGTGAATTGCAGAATTCAGTGAATCATCGAATCTTTGAACGCACAT
TGCGCCCCTCGGTATTCCGTGGGGCATGCCTGTTCGAGCGTCATCTACACCCTCAAGCTCTG
CTTGGTGTTGGGCGTCTGTCCCGCCTCCGcgcgTGGACTCGCCCCAAATTCATTGGCAGCGG

TCCTTGCCTCCTCTCGCGCAGCACATTGCGCTTCTCGAGGGGCTACGGCTCGCGTCCAACAA
GCACATTTACCGTCTTTGACCTCGGATCAGGTAGGGATACCCGCTGAACTTAA
k__Fungi;p__Ascomycota;c__Dothideomycetes;o__Pleosporales;f__P
leosporaceae;g__Leptosphaerulina;s__Leptosphaerulina_chartarum
1.000

>OTU_110
GAAATGCGATAAGTAATGTGAATTGCAGAATTCAGTGAATCATCGAATCTTTGAACGCACAT
TGCGCCCGCCAGTACTCTGGCGGGCATGCCTGTTCGAGCGTCATTTCAACCCTCAAGCTCTG
CTTGGTGTTGGGGATCGGCGTAGTGCCTTCGGGCGCTGCCGTCCCCTAAATCTAGTGGCGGT
CCCGCTGTAGCCTCCTCTGCGTAGTAGTTTTCTCCTCGCAATGGAATCTCGGCGGTGCCTTG
CCGTTAAACCcccAACTTCTGAAAGGTTGACCTCGAATCAGGTAGGAATACCCGCTGAACTT
AA
k__Fungi;p__Ascomycota;c__Sordariomycetes;o__Hypocreales;f__Ne
ctriaceae;g__Volutella;s__Volutella_consors 0.990

>OTU_111
GAAATGCGATAAGTAATGTGAATTGCAGAATCTCGTGAATCATTGAATCTTTGAACGCACAT
TGCGCCCTATGGTATTCCGTAGGGCATGCCTGTCTGAGCGTCAGCTCGTCTTCTCAAGCTCT
TTTGCTTGGATAACATTGGAACAGCAATACAAGCTCCATCTGGGGTCTGtgtgCTCTCCAGA
AATCAATAGGCGGTACGGTTCTAGCAAACCAGACGTAATAATATTCTTATCTATCGTTAATT
TGTTTAAACTGTTGCTTGCCTCTTAATCCACATCTTtttCAAGATGACCTCAGATCAGGTAG
GGATACCCGCTGAACTTAA
k__Fungi;p__Ascomycota;c__Pezizomycetes;o__Pezizales;f__uniden
tified;g__unidentified;s__Pezizales_sp_P10 1.000

>OTU_112
AAACTACGTTAATCAGTGTAAACTGCACAATAGCAGAACACTTGAACTTCGAACGCACATTG
CGGCTAAAGGGTAACTCCTCTAGCCTTATCTGCGTGAGGGCTGTATAGAGATATTGAACCCA
TGTGTAGGGAGTGATCACTTGAACGTGAGACTCCGCAGAGTGAACCTCGCTAGTGGTCTCTG
ACTGCTGACGTCTCAGGCAGGCAAGACCGCGGGTTAGCGAAGGTGACTAACACTCAcacacT
TATCTACAACAGCCTCACGTCAGGTAAGATCACCCGCTGAACTTAA
k__Fungi 1.000

>OTU_113
GAAATGCGATAAGTAATGTGAATTGCAGAATTCAGTGAATCATCGAATCTTTGAACGCACCT
TGCGCTCCTTGGTATTCCTTGGAGCATGCCTGTTTGAGTATCATGATATCTTCAAAGTAAAT
CTTTTGTTAATTCAATTGGTTCTACTTTGGTATTGGAGGTTtttGCAGTTTCACACCTGCTC
CTCTTTGTGCATTAGCTGGATCTCAGTGTTATGCTTGGTTCCACTCGGCGTGATAAGTTATC
TATCGCTGAGGACACTGTAACAGGTGGCCAAGGTAAATGCAGATGAACCGCTTCTAATAGTC
CATTGACTTGGACAATATTtttATGATCTGATCTCAAATCAGGTAGGACTACCCGCTGAACT
TAA
k__Fungi;p__Basidiomycota;c__Agaricomycetes;o__Cantharellales;
f__Ceratobasidiaceae;g__Ceratobasidium sp. AG-V 0.950

>OTU_114
GAAACGCGATAAGTAATGTGAATTGCAGAATTCAGTGAATCATCGAATCTTTGAACGCACAT
GGCGCCTTCCAGTATCCTGGGAGGCATGCCTGTCCGAGCGTCGTTTCAACCCTCGAGCCccc
GTGGCCCGGCGTTGGGGATCTGCCCAGGCAGGCCCCGAAAACCAGTGGCGGACCCGTTACAG
GCCCTTCCTTTGCGTAGTAATACTGCCTCGCATCGGGAGCCGGCGGGCTTCCAGCCTCTAAA
CCcccAGCAAGTCCGCCCCGGCGGCACCAAGGTTGACCTCGGATCAGGTAGGAATACCCGCT
GAACTTAA
k__Fungi;p__Ascomycota;c__Sordariomyhcetes;o__CGlomerellales;f__Plectosphaerellaceae;g__Chordomyces;S__Chordomyces__antarcticum 1.00

>OTU_115
GAAATGCGATAAGTAATGTGAATTGCAGAATTCAGTGAATCATCGAATCTTTGAACGCACAT
TGCGCCCGCCAGAATTCTGGCGGGCATGCCTGTTCGAGCGTCATTTCAACCCTCGGTCTCCC
TCCGGGAgagaCCGGCGTTGGGGACCGGCACTTACCCCGCCGGCCCCGAAATGAAGTGGCGG
CCCGTCCGCGGCGACCTCTGCGTAGTAACTCCACTCGCACCGGAACCCGGGCGCGGCCACGC
CGTAAAACCcccAACTTCCGAATGTTGACCTCGAATCAGGTAGGAATACCCGCTGAACTTAA
k__Fungi;p__Ascomycota;c__Sordariomycetes;o__Hypocreales;f__Incertae_sedis;g__Acremonium;s__Acremonium_sp_06857 0.99

>OTU_116
GAAATGCGATAAGTAATGTGAATTGCAGAATTCAGTGAATCATCGAATCTTTGAACGCACAT
TGCGCCCGCCAGTATTCTGGCGGGCATGCCTGTCTGAGCGTCATTTCAACCCTCATGCCCCT
AGGGCGTGGTGTTGGGGATCGGCCAAAGCCCGCGAGGGACGGCCGGCCCCTAAATCTAGTGG
CGGACCCGTCGTGGCCTCCTCTGCGAAGTAGTGATATTCCGCATCGGAGAGCGATGCGCCCC
TGCCGTTAAACCcccAACTTTCTAAGGTTGACCTCAGATCAGGTAGGAATACCCGCTGAACT
TAAGCATATCAATAAGCGGAGGAGCATATCAATAAGCGGAGGAATCCGTAGGTGAACCTGCG
GA
k__Fungi;p__Ascomycota;c__Sordariomycetes;o__Hypocreales;f__Bionectriaceae;g__ Clonostachys;s__Clonostachys_rosea   0.990

>OTU_117
GAATTGCGTTAAGAATCACGAATTACAGATATTATGAGTGATATGTTTTCGATTGCATATTG
CATCGTTGGGCACTTGCCCATCGGTATACCCAACTCAGGGTgtgtATCACTAATCGGAAGAC
ACAATGCACTTGTTGGTTGTGCCTGAATTCGGAGCTGTGCCAATACTTGTATTGCATTGTTT
TATTTGAGGTGTGGCTGAATTGTAGAAATGAAGGAGAATGAGGTTGAAAATGCTTGGTGGTT
GTTGGAAGTTTAATGAATTtttGAGCTTCTGGTGACTATTCGGTTttttGACTTtttCTACA
TAGCCCTGCATACACCAGCCAATTGTTtttGGTTTAATCCACCTGAGTCGGATATGATCACC
CGCCGAACTTAA
Aphelenchoides sp.

>OTU_118
GAAATGCGATAAGTAATGTGAATTGCAGAATTCAGTGAATCATCGAATCTTTGAACGCACAT

TGCGCCCATTAGTATTCTAGTGGGCATGCCTGTTCGAGCGTCATTTCAACCCTTAAGCCTAG
CTTAGTGTTGGGAGACTGCCTAATACGCAGCTCCTCAAAACCAGTGGCGGAGTCTGTTCGTG
CTCTGAGCGTAGTAATTCTTTATCTCGCTTCTGCAAGCCGATTAGACAACAGCCATAAACCG
CACCCTTCGGgggCACTTtttAATGGTTGACCTCGGATCAGGTAGGAATACCCGCTGAACTT
AA
k__Fungi;p__Ascomycota;c__Sordariomycetes;o__Xylariales;f__Hyp
onectriaceae;g__ Microdochium;s__ Microdochium_nivale 1.00

>OTU_119
GAAATGCGATAATTAATGTGAATTGCAGAATTCAGTGAATCATCGAGTCTTTGAACGCACAT
TGCGCCcccTGGTATTCCGGggggCATGCCTGTCCGAGCGTCATTGCTGCCCTCAAGCCCGG
CTTGtgtgTTGGGTGTGGTCCccccGGGGACCTGCCCGAAAGGCAGCGGCGGCACCGCGTCC
GATCCTCGAGCGTATGGGGCTTTGTCACATGCTCTGTAGGATTGGCCGGCGCCTGCCGACGT
TTTCCAACCATTCTTTCCAGGTTGACCTCGGATCAGGTAGGGATACCCGCTGAACTTAA
k__Fungi;p__Ascomycota;c__Eurotiomycetes;o__Eurotiales;f__Tric
hocomaceae;g__Aspergillus;s__Aspergillus_niger 0.960

>OTU_120
TAACTGCGATAAGTAGCGTGAATTGCAGACGCTTTGAACGTTAAACTTTCGAACGCACATTG
CGCCGTAGGAGTTCTACCCTGCGGCACATCTGGTTGAGGGTCGTGATCAAaaaCTGCCCGAA
TGCAGCAGTACAGATAGCTGGCGAACCATGATGAAAGTCGTGATTTGCCATACGACGCTATA
GAGGACAATTCTCCACGTGAgagagTTGCCTGCCACACGGATGATCTACTAGTTGACGCAGT
AGATCCCGGTCCCAGGTATCGTATTATGCCGAGGGATGCGCGGGGATACAGCTAGTGAgaga
ATCTGTGCGGCGCTATGAGCTATTCGATTTCCCGACCTCAACTCAGGTGTGATTACCCGCTG
AACTTAAGCATATCAATAAGCGGAGGAATCCGTAGGTGAACCTGCGG
Pratylenchus goodeyi

>OTU_121
GAAATGCGATAAGTAATGTGAATTGCAGATACAGTGAATCATCGAGTCTTTGAACGCAAGTT
GCGCCCGAAGCCCTTAGGCTGAGGGCACGCCTGCCTGGGTGTCACCAAAGGCGCCccccGT
CCCGCCCGTCCGAGGGCACGGGGAGGgggCGAATGTTGGCCTCCCGGGAGCCCCTGGCTCGC
GGTTGGTTCAAAGAGACGGGCTCTTGGTGGGGAGCGGCACCGCGGCAGATGGTGGTCGAGAA
CAACCCTCGTGGCCAGTCGcgcgcgcCTCTCCcccGGTTCAAGGCACGGCGACCCGCGGGCG
ACGTGGATCGTCCCGAGCGCGACCTCAGGTCAGGCGGGGCTACCCGCTGAGTTTAA
Arachis hypogaea

>OTU_122
AAATCGCGATATGTAATGTGATCTGCCTATAGTGAATCATCAAATCTTTGAACGCATCTTGC
ACCTTATGGTATTCCATAAGGTACGTCTGTTTCAGTACCACTAATAAATCtctctctATCCT
TGATGATAGAAaaaaaaGAGATAAATTATTACTGGTCCTGGTGATTCTTTCTCTTTTGAAaa
aaTCACTCTCGGCCTAAATATAAGGCTCGACTTTtttttACCAGATCTTGCATCTAGTAAaa
aCCTAGTCGGCTTTAATAGATTtttATTTTCTATTAAGTTTATAGCCATTCTTATATTtttt
AAAATCTTGGCCTGAAATCAGATGGGACTACCCGCTGAACTTAA
k__Fungi;p__Zygomycota;c__Incertae_sedis;o__Mucorales;f__Cunni

nghamellaceae;g__Cunninghamella;s__Cuninghamella_polymorpha
1.000

>OTU_123
GAAATGCGATACTTGGTGTGAATTGCAGAATCCCGTGAACCATCGAGTCTTTGAACGCAAGT
TGCGCCCGAAGCCATTAGGCCGAGGGCACGCCTGCCTGGGTGTCAcacaTCGTTTCCCCAAC
GCAAACATGTAACAATGTTGCTGCGCGGGGTGTATGCTGACCTCCCGCGAGCACCCGCCTCG
TGGTTGGTTGAAATCTGGGTTCATGGCCGACTTCGCCGTGATAAAATGGTGGATGAGCCACG
CTCGAGACCAATCACGTGCGAGCCGGTCAGTTCTGGACCCATCGACGACCCTTTGCGTGCAC
GCACGCTCCCAACGAGACCTCAGGTCAGGCGGGGCTACCCGCTGAGTTTAA
Glycine max

>OTU_124
GAAATGCGATACTTGGTGTGAATTGCAGAATCCCGTGAACCATCGAGTCTTTGAACGCAAGT
TGCGCCCGACGCCTTCGGGCTGAGGGCACGCCTGCCTGGGCGTCACGCATCGCGTCGCCccc
TATACCAATTTGGTGCGGggggCGGATAATGGCATCCCGTTAGCTTGGTTTGCCCAAaaaGG
ATCCCTCATCGACGGATGTCACAACCAGTGGTGGTTGAAAGATCATTGGTGTTGTTGTGCAT
CACTCTGTCGCATGCTTGGGCATCGTTATAAAACAATGGTGCTAACGCGCCTTCGACCGCGA
CCCCAGGTCAGACGGGACTACCCGCTGAGTTTAA
Plantago camtschatica

>OTU_125
GAAATGCGATAACTAATGTGAATTGCAGAATTCAGTGAATCATCGAGTCTTTGAACGCACAT
TGCGCCcccTGGTATTCCGGggggCATGCCTGTCCGAGCGTCATTGCTGCCCTCAAGCCCGG
CTTGtgtgtTGGGCCCCGCCccccGGCTCCGGggggCGGGCCCGAAAGGCAGCGGCGGCACC
GCGTCCGGTCCTCGAGCGTATGGGGCTTCGTCACCCGCTCCGTAGGCCCGGCCGGCGCCCGC
CGGCGACCccccTCAATCTTTCTCAGGTTGACCTCGGATCAGGTAGGGATACCCGCTGAACT
TAA
k__Fungi;p__Ascomycota;c__Eurotiomycetes;o__Eurotiales;f__Tric
hocomaceae;g__Penicillium;s__Penicillium_janthinellum 0.870

>OTU_126
GAAATGCGATACTTGGTGTGAATTGCAGAATCCCGCGAATCATCGAGTCTTTGAACGCAAGT
TGCGCCCGAAGCCATTCGGCCGAGGGCACGTCTGCCTGGGTGTCACGCATCGTTGCCCCAAC
CCCAAACACTTCTTATGATGtgtgGGGTGCGGGGAAGACATTGGCCTCCCGTGTGCTTCTGC
TCGCGGTTAGCCTAAAAGTGAGTCCTAGGCGACGAGCGCCACGACAATCGGTGGTTGAGAAA
CCCTCGTGACCCGTCGTgtgtTGCCCGTCGCTGTGAAGGTGCTCCTCGACCCTATTGCGTCG
TTCCTGCGACTCTACCATCGCGACCCCAGGTCAGGCGGGATTACCCGCTGAATTTAA
Juglans regia

>OTU_127
GAAATGCGATAATTAATGTGAATTGCAGAATTCAGTGAATCATCGAGTCTTTGAACGCACAT
TGCGCCcccTGGTATTCCGGggggCATGCCTGTCCGAGCGTCATTGCTGCCCATCAAGCACG
GCTTGtgtgtTGGGTCGTCGTCCCCTCTCCGGgggggACGGGCCCAAAAGGCAGTGGCGGCA

CCATGTCTGGTCCTCGAGCGTATGGGGCTTTGTCACCCGCTCTGTAGGCCCGGCCGGCGCTT
GCCGAACGCAAATCAATCTTtttCCAGGTTGACCTCGGATCAGGTAGGGATACCCGCTGAAC
TTAA
k__Fungi;p__Ascomycota;c__Eurotiomycetes;o__Eurotiales;f__Tric
hocomaceae;g__Aspergillus;s__Aspergillus_flavus 0.980

>OTU_128
ATATCAATAAGCGGAGGACGCCTGCTTCAGTATCATCACAAACCCAcacaTAACATTTGTTT
ATGTGGTGATGGGTCGCATCGCTGTTTTATTACAGTGAGCACCTAAAATGtgtgtgATTTTC
TGTCTGGCTTGCTAGGCAGGAATATTACGCTGGTCTCAGGATCTTtttttttGGTTCGCCCA
GGAAGTAAAGTACAAGAGTATAATCCAGTAACTTTCAAACTATGATCTGAAGTCAGGTGGGA
TTACCCGCTGAACTTAA
k__Fungi;p__Zygomycota;c__Incertae_sedis;o__Mucorales;f__Mucor
aceae;g__Rhizopus;s__Rhizopus_oryzae 1.000

>OTU_129
GAAATGCGATAAGTAATGTGAATTGCAGAATTCAGTGAATCATCGAATCTTTGAACGCACAT
TGCGCCCGCCAGTATTCTGGCGGGCATGCCTGTTCGAGCGTCATTTCAACCATCAAGCCCCG
GGCTTGTGTTGGGGACCTGCGGCTGCCGCAGGCCCTGAAATGCAGTGGCGGGCTCGCTGTCA
CTCCGAGCGTAGTAGTTACATCTCGCTCTGGGCGTGCTGCGGGTTCCGGCCGTTAAAAGCCT
TATTTACCCAAGGTTGACCTCGGATCAGGTAGGAAGACCCGCTGAACTTAA
k__Fungi;p__Ascomycota;c__Sordariomycetes;o__Sordariales;f__Ch
aetomiaceae;g__Humicola;s__Humicola_fuscoatra 1.000

>OTU_130
ATCGATGAAGAACGCAGCATCGATGAAGAACGCAGCATCGATGAAGAACGCAGCATCGATGA
AGAACGCAGCATCGATGAAGAACGCAGCATCGATGAAGAACGCAGCATCGATGAAGAACGCA
GCATCGATGAAGAACGCAGCATCGATGAAGAACGCAGCATCGATGAAGAACGCAGCATCGAT
GAAGAACGCAGCATCGATGAAGAACGCAGCATCGATGAAGAACGCAGCATCGATGAAGAAC
Uncultured *Oidiodendron*

>OTU_131
GAAATGCGATACGTAATGTGAATTGCAGAATTCAGTGAATCATCGAGTCTTTGAACGCACAT
TGCGCCcccTGGTATTCCGGggggCATGCCTGTCCGAGCGTCATTGCTGCCCTCAAGCACGG
CTTGtgtgtTGGGCCcccGTCCcccTGTTCTAGGgggggACGGGCCCGAAAGGCAGCGGCGG
CACCGTGTCCGGTCCTCGAGCGTATGGGAAGCAACTTtttGTCACCCGCTCCTGTAGGTCCG
GCCGGCGGCCTGCCCAACCcccATCAATCTTtttttAACCAGGTTGACCTCGGATCAGGTAG
GGATACCCGCTGAACTTAA
k__Fungi;p__Ascomycota;c__Eurotiomycetes;o__Eurotiales;f__Tric
hocomaceae;g__Aspergillus;s__Aspergillus_penicillioides 1.000

>OTU_132
GAAATGCGATAAGTAATGTGAATTGCAGAATTCCGTGAATCATCGAATCTTTGAACGCACAT
TGCGCCCTCTGGTATTCCGGggggCATGCCTGTCCGAGCGTCATTGCAACCCCTTCAAGCCC

GGCTTGtgtgtTGGGCGTCGTCCCCGCTGGACGCGCCCGAAAGGCAGTGGCGGCTCCGTGTC
CGGTGCCCGAGCGTATGGGCTTCAATCACCCGCTCTGGTGGCCCGGCCGGCGCTGGCCTTCT
CAGTCTTGACCAACTTTTGGTTGAGGCCTTGTGAACTTTCGTGGTTGACCTCGGATCAGGTA
GGGATACCCGCTGAACTTAA
Uncultured fungus

>OTU_133
GAAATGCGATACGTAATATGAATTGCAGATATTCGTGAATCATCGAATCTTTGAACGCACAT
TGCGCCCTCTGGTATTCCGGAGGGCATGCCTGTTTGAGCGTCGTTTCTCCCTCAAACCGCTG
GGTTTGGTGTTGAGCAATACGACTTGGGTTTGCTTGAAAGACGGTAGTGGTAAGGCGGGATC
GCTTTGACAATGGCTTAGGTCTAACCAAaaaCATTGCTTGCGGCGGTAACGTCCACCACGTA
TATCTTCAAACTTTGACCTCAAATCAGGTAGGACTACCCGCTGAACTTAA
k__Fungi;p__Ascomycota;c__Saccharomycetes;o__Saccharomycetales
;f__Incertae_sedis;g__Candida;s__Candida_albicans 1.000

>OTU_134
GAAATGCGATACGTAATGTGAATTGCAGAATTCAGTGAATCATCGAGTCTTTGAACGCACAT
TGCGCCcccTGGTATTCCGGggggCATGCCTGTCCGAGCGTCATTGCTGCCCTCAAGCACGG
CTTGtgtgtTGGGCCCCGCCccccGGTCCCGGggggCGGGCCCGAAAGGCAGCGGCGGCACC
GCGTCCGGTCCTCGAGCGTATGGGGCTTTGTCACCCGCTCCGTAGGCCCGGCCGGCGCCCGC
CGGCGACCcccAATCAATCTATCCAGGTTGACCTCGGATCAGGTAGGGATACCCGCTGAACT
TAA
k__Fungi;p__Ascomycota;c__Eurotiomycetes;o__Eurotiales;f__Tric
hocomaceae;g__Penicillium;s__Penicillium ochrochloron 1.000

>OTU_135
GAAATGCGATACCTGGTGTGAATTGCAGAATCCCGCGAACCATCGAGTCTTTGAACGCAAGT
TGCGCCCGAGGCCACTCGGCCGAGGGCACGCCTGCCTGGGCGTCACGCCAAAACACGCTCCC
AACCACTCTCAACGGGAATCGGGATGCGGCATCTGGTCCCTCGTCtctcAAGGGACGGTGGA
CCGAAGATTGGGCTGCCGGCGTACCGCGCCGGACACAGCGCATGGTGGGCGTCCTCGCTTTA
TCAATGCAGTGCATCCGGCgcgcAGCTGGCATTATGGCCTTTGAACGACCCAACAAACGAAG
CGCACGTCGCTTCGACCGCGACCCCAGGTCAGGCGGGACTACCCGCTGAGTTTAA
Triticum aestivum

>OTU_136
GAAATGCGATAAGTAATGTGAATTGCAGAATTCAGTGAATCATCGAATCTTTGAACGCACCT
TGCGCCTTTTGGTATTCCGAAAGGCATGCCTGTTTGAGTGTCATGAAATATCAACCCCTCCT
GGTTTCTGATCAGTGTTGGGCTTGGACTTGGGTGTCTGCCAGTTCGCTGGCTCGCCTTAAAA
GAGTTAGTGGTAATAACATCCACGGCTAAGACGTAATAAGTTTCGTCTGGTCAAGGTGTGAC
GACTGCTCATAACCCGACCGCAAGGTCATTATTTTATGCTCTGACCTCAAATCAGGTAGGAC
TACCCGCTGAACTTAA
k__Fungi;p__Basidiomycota;c__Tremellomycetes;o__Tremellales;f_
_Tremellaceae;g__unidentified;s__uncultured Bulleromyces albus
1.000

>OTU_137
GAAACGCGATAGGTAATGTGAATTGCAGAATTCCGTGAATCATCGAATCTTTGAACGCACCT
TGCGCTCTATGGCAATCCGTAGAGCATGCCTGTTTGAGTGCCATGAAATCTCCCACCCCAAG
CGGTTtttAAATGAAACGGCTTGGCGGATGGGGTCTGGATGGGTGCCTCTGCCTGCGCTACC
TAGCACAGGCTCGCCCGAAATGCATGAGCGCCTTGAGACACTTTGCATCCGCCTctctGTTT
GGGAGGAGGCGGCCAAGCAGTGTTtttCTCCTGGCATGGCATGATACGTCATTTGCTATGTC
ACCTAAAGGAGGAATGTTTGGTTGTGTCTGCGTGTGCTTCAAACTTGCCTCTGTGGCACATC
CCAATTTCACTTCTGGTCTCAAATCAGGTAGGATCACCCGCTGAACTTAA
Uncultured *Malassezia*

>OTU_138
GAAATGCGATACGTAGTGTGAATTGCAGAATTCAGTGAATCATCGAATCTTTGAACGCACAT
TGCGCCCTTTGGTATTCCAAAGGGCATGCCTGTTCGAGCGTCATTTGTACCCTCAAGCTTTG
CTTGGTGTTGGGCGTCTTGTCTCCAGTTCGCTGGAGACTCGCCTTAAAGTCATTGGCAGCCG
GCCTACTGGTTTCGGAGCGCAGCACAAGTCGCGCTctctTCCAGCCAAGGTCAGCGTCCAGT
AAGCCTTtttttCAACTTTTGACCTCGGATCAGGTAGGGATACCCGCTGAACTTAA
k__Fungi;p__Ascomycota 1.000

>OTU_139
GAAATGCGATAACTAATGTGAATTGCAGAATTCAGTGAATCATCGAGTCTTTGAACGCACAT
TGCGCCcccTGGTATTCCGGggggCATGCCTGTCCGAGCGTCATTGCTGCCCTCAAGCCCGG
CTTGtgtgtTGGGTCGCCGTCCCCTCTCCGGgggggACGGGCCCCAAAGGCAGCGGCGGCAC
CGCGTCCGATCCTCGAGCGTATGGGGCTTTGTCACCCGCTCTGTAGGCCCGGCCGGCGCTTG
CCGACGTTTTCCAACCATTCTTTCCAGGTTGACCTCGGATCAGGTAGGGATACCCGCTGAAC
TTAA
k__Fungi;p__Ascomycota;c__Eurotiomycetes;o__Eurotiales;f__Tric
hocomaceae;g__Aspergillus;s__Aspergillus_ellipticus 0.970

>OTU_140
GAAATGCGATACTTGGTGTGAATTGCAGAATCCCGTGAACCATCGAGTCTTTGAACGCAAGT
TGCGCCCGAAGCCCTTAGGCTGAGGGCACGCCTGCCTGGGTGTCACCAAAAGGCGCCccccG
TCTCGCCCGTCCCAGGGCACGGGGAGGgggCGAACGTTGGCCTCCCGGGAGCCCCTGGCTCG
CGGTTGGTTCAAAGAGACGGGCTCTTGGTGGGGAGCGGCCCCGCGGGAGAGGGTGGGAgaga
ACAACCCTGGTGGCCAGGCGGGCGGGCCTTGCCcccGGTTCAAGGGACGGCGACGcgcgGGC
GACGGGGATCGTCCCGAGCGCGACCTCAGGTCAGGCGAGGCTACGGGCTGAGTGTAA
Arachis hypogaea

>OTU_141
TGCTCAATCATCAGCCCCTGACGCTGTTCGCTATCCAGAAGCTCGGTCATCTGGCGTGAATC
GAGCTTGGGTAACATCAAAACCAACATCAACACCAGCGCCAGCGTCAGCCAGAAGATGGCGA
AGATGCgcgcgGTTAAGCTGCCTATCATGAAGCAGAAACCATCAGATAGCCGCGACCACGCA
AGGTTTTAAACCACGGGTGACCATCTTTACGATCCGGCAGTTTACGACGCAGGTTGGAAATG
T

Eschericha coli

>OTU_142
GAAGTGCGATAAGCAATGCGAATTGCAGAACCGTGAGTCATCAGATTtttGAACGCAACTGG
CGCTGGCTGGATCTCCAGCCAGCATGCTTGTTTCAGTGTCTTGTTTCCTCCTCACCCAAACC
TTAATGCGAgagaTACCCTTCTCTTGTCAAGCACGAAAGCACTCTGCGCTCTGCGAGCGGCT
TCTGACTAGCTCAGAGGTCGCACTCAATGCAGACGTCACATTTCTCACAATGTGAACTCATT
GAGAGTAGAGGCTGTCAGCTCACGCTGTCAGTGCTTAGTCACTCAAACTTTCTTTGCATCTG
AAATCAAGCAGGATCACCCGCTGAACTTAA
Uncultured fungus

>OTU_143
TGGATGCGCCGGTGAGCGGCGGCGAACCGAAAGCCATCGACGGCACGCTGTCGGTCATGGTC
GGCGGCGATAAAGCGATCTTCGATAAATACTACGATCTGATGAAAGCGATGGCCGGCTCCGT
AGTGCATACCGGCGATATTGGCGCGGGCAACGTCACCAAACTGGCTAACCAGGTGATTGTCG
CGCTGAACATCGCGGCGATGTCTGAAGCGCTGACCCTGGCGACTAAAGCCGGCGTCAATCCG
GATCTGGTTTATCAGGCTATTCGCGGCGGTCTGGCGGGCAGTACCGTGCTGGATGCGAAAGC
CCCGATGGTGATGGACCGTAACTTCAAACCGGGTTTCCGTATCGACCT
Klebsiella pneumoniae

>OTU_144
GAAATGCGATACGTAATGTGAATTGCAGAATTCAGTGAATCATCGAGTCTTTGAACGCACAT
TGCGCCcccTGGTATTCCGGggggCATGCCTGTCCGAGCGTCATTGCTGCCCTCAAGCACGG
CTTGtgtgtTGGGCCcccGTCCcccTCTCCGGggggACGGGCCCGAAAGGCAGCGGCGGCAC
CGCGTCCGATCCTCGAGCGTATGGGGCTTTGTCACCCGCTCTGTAGGCCCGGCCGGCGCTTG
CCGAACGCAAATCAATCTTtttCCAGGTTGACCTCGGATCAGGTAGGGATACCCGCTGAACT
TAA
k__Fungi;p__Ascomycota;c__Eurotiomycetes;o__Eurotiales;f__Aspe
rgillaceae;g__Penicillium;s__Penicillium_infrapurpureum 0.970

>OTU_145
GAAATGCGATAACTAGTGTGAATTGCAGAATTCCGTGAATCATCGAGTCTTTGAACGCACAT
TGCGCCcccTGGTATTCCGGggggCATGCCTGTCCGAGCGTCATTGCTGCCCATCAAGCACG
GCTTGtgtgtTGGGTGTGGTCCccccGGGGACCTGCCCGAAAGGCAGCGGCGACGTCCGTCT
GGTCCTCGAGCGTATGGGGCTTTGTCACCCGCTCCCGTAGGTCCAGCTGGCAGCTAGCCTCG
CAACCAATCTTtttAACCAGGTTGACCTCGGATCAGGTAGGGATACCCGCTGAACTTAA
k__Fungi;p__Ascomycota;c__Eurotiomycetes;o__Eurotiales;f__Tric
hocomaceae 1.000

>OTU_146
AAAATATGATACTTAGTATGAATTGCAGAATCCCTTGAACTATCAAGTCTCTAAAAGCAAGT
TGCACTCGAAGCCATCAGGCCTAGGGCATGTCTGCCTAGGCATCAAGCATCGCATCCccccT
ATATGCTGCTCAGCATCATGGTGCCGGATATTGGCCTCCCGTGTGAGTCCACGTCTACGAAC
ATCGCAGTAAGTAGTGGTTGTAACTCTACTctctTAGTGCCGCGGCTACAGCTCATCGCACG

TgtgtgCTCCCCGACCCTTCTGACTCTCGcgcgcTCCAACTGCAATCCCAGGTCAGGCGGGG
TTACCTACTGAGTTTAA
No similar sequence

>OTU_147
GAAATGCGATAAGTAATGTGAATTGCAGAATTCAGTGAATCATCGAATCTTTGAACGCACAT
TGCGCCCCTTGGTATTCCGAGGGGCATGCCTGTTCGAGCGTCATTACAACCCTCAAGCTCTG
CTTGGTATTGGGCGCCGTCCTCCCCGCTGGggggggACGCGCCTCAAAGACCTCGGCGGTGG
CTCTGGCCCTCAAGCGTAGTAGATTATACACCTCGCTTTGGAGCGGTCTGGAGTCGCCCGCC
GGACGAACCTTCTGAACTTTTCTCAAGGTTGACCTCGGATCAGGTAGGGATACCCGCTGAAC
TTAA
k__Fungi;p__Ascomycota;c__Dothideomycetes 0.990

>OTU_148
GAAATGCGATACTTGGTGTGAATTGCAGGATCCCGCGAACCATCGAGTTtttGAACGCAAGT
TGCGCCCGAAGCCTTCTGGCCGAGGGCACGTCTGCCTGGGTGTCACTCAATCGTCGCCCCAA
CCTCCTCCTTTCGGGAGGggggCGGAAAATGGCCTCCCGTAAACTCTTTGTTTGCGGTTGGC
CCAAATGTCCGGTCCTCGGCAACGATGCCACGGCAATCGGTGGTTGTATGACCCTCGCTAAA
TGCTGTGACCGCTCGGTTGCTGACGTGACTTACGAGGCCCCAAAGTGTTTCTGAAGAAGCGC
TCGCAATGCGACCCCAGGTCAGGCGGGATTACCCGCTGAGTTTAA
Euphorbia maculata

>OTU_149
GAAATGCGATAAGTAATGTGAATTGCAGAATTCAGTGAATCATCGAGTCTTTGAACGCACAT
TGCGCCcccTGGTATTCCGGgggTCATGCCTGTCCGAGCGTCATTGCTGCCCTCAAGCACGG
CTTGtgtgtTGGGCTTCCGTCCCTGGTAACGGGGACGGGCCCAAAAGGCAGTGGCGGCACCA
TGTCTGGTCCTCGAGCGTATGGGGCTTTGTCACCCGCTCCCGTAGGTCCAGCTGGCAGCTAG
CCTCGCAACCAATCTTtttAACCAGGTTGACCTCGGATCAGGTAGGGATACCCGCTGAACTT
AAGCATATCAATAAGCGGAGGAGCATATCAATAAGCGGAGGAGTCACCTGCG
k__Fungi;p__Ascomycota;c__Eurotiomycetes;o__Eurotiales;f__Tric
hocomaceae;g__Eurotium;s__Eurotium_niveoglaucum 1.000

>OTU_150
GAAATGCGATACTTGGTGTGAATTGCAGAATCCCGTGAACCATCGAGTCTTTGAACGCAAGT
TGCGCCCGAAGCCCTTAGGCTGAGGGCACGCCTGCCTGGGTGTCACCAAAAGGCGCCTCCCG
TCTCGCCCGTCCCAGGGGCCTCCCGGGAGCCCCTGGCTCGCGGTTGGTTCAAAGAGACGGGC
TCTTGGTGGGGAGCGGCACCGCGGCAGATGGTGGTCGAGAACAACCCTCGTGGCCAGTCGcg
cgcgcCTCTCCcccGGTTCAAGGCACGGCGACCCGCGGGCGACGTGGATCGTCCCGAGCGCG
ACCTCAGGTCAGGCGGGGCTACCCGCTGAGTTTAA
Arachis hypogaea

>OTU_151
GAAATGCGATAAGTAATGTGAATTGCAGATACAGTGAATCATCGAATCTTTGAACGCACATT
GCGCCCGCCAGTATTCTGGCGGGCATGCCTGTTCGAGCGTCATTTCAACCCTCAAGCCCAGC

TTGGTGTTGGGACTCGCGAGTCAAATCGCGTTCCCCAAATTGATTGGCGGTCACGTCGAGCT
TCCATAGCGTAGTAGTAAAACCCTCGTTACTGGTAATCGTCGCGGCCACGCCGTTAAACCCC
AACTTCTGAATGTTGACCTCGGATCAGGTAGGAATACCCGCTGAACTTAA
k__Fungi;p__Ascomycota;c__Sordariomycetes;o__Hypocreales;f__Nectriaceae;g__Gibberella;s__Fusarium_verticillioides 0.980

>OTU_152
TAACTGCGATAAGTAGCGTGAATTGCAGACGCTTTGAACGTTGAACTTTCGAACGCACATTG
CGCCGTAGGAGTTCTACCCTACGGCACATCTGGTTGAGGGTCGTGATCAAAACTAGCCGGAA
TCGTATGGCTATAAGagagGAATAGCTGGTGAATTACTCGGCGAGCCGATGCCTCCGGCTCG
CAGTGGTTCCCCATCATGCTATACAGGGGACACCCGCTCGCGGGAGCCCGCCACACGGATGA
TCGACGGCTAGCCGATGCCCGTAGATCCCGGTCCAACGGTATTATACTTATATGCTCGAGGA
CGTCTGGGATACGGCTCGCCGTATTGTGGTTGTACGTGCCCGTGGCGAATGCACTCGTTCTC
CCGACCTCAACTCAGGTGTGATTACCCGCTGAACTTAA
Pratylenchus goodeyi

>OTU_153
GAAATGCGATACTTGGTGTGAATTGCAGAATCCCGTGAACCATCGAGTCTTTGAACGCAAGT
TGCGCCCGAAGCCACTAGGCCGAGGGCACGCCTGCCTGGGCGTCACACGCCGTTGCCccccc
cATCTACTCCTTCGGGATTGCGGgggggCGGATGATGGCCTCCCGTGTTCCTCGCCGcgcgG
TTGGCATAAATACCAAGTCCTCGGCGACGCATGCCACGACAATCGGTGGTTGCGAAACCTCG
GTTGCCCGTCGTGTGCGTTCGTCGCGCATCGAGGGCTCGAAaaaaTGCTCGGCTCCGGCTCG
GCTTTCAACGCGACCCCAGGTCAGGCGGGGTTACCCGCTGAATTTAA
Prunus persica

>OTU_154
GAAATGCGATACGTAATGTGAATTGCAAATTCAGTGAATCATCGAGTCTTTGAACGCACATT
GCGCCcccTGGTATTCCGGggggCATGCCTGTCCGAGCGTCATTGCTGCCCTCAAGCCCGGC
TTGtgtgtTGGGCCCCGTCCTCCGATTCCGGgggACGGGCCCGAAAGGCAGCGGCGGCACCG
CGTCCGGTCCTCGAGCGTATGGGGCTTTGTCACCCGCTCTGTAGGCCCGGCCGGCGCTTGCC
GATCAACCCAAATTtttATCCAGGTTGACCTCGGATCAGGTAGGGATACCCGCTGAACTTAA
k__Fungi;p__Ascomycota;c__Eurotiomycetes;o__Eurotiales;f__Trichocomaceae;g__Penicillium;s__Penicillium expansum 1.000

>OTU_155
GAAATGCGATAAGTAATGTGAATTGCAGAATTCAGTGAATCATCGAATCTTTGAACGCACAT
TGCGCCcccTGGTATTCCGGggggCATGCCTGTTCGAGCGTCATTACAACCCTCAAGCTCTG
CTTGGTATTGGGCCCCGCCGGTTCCGGCGGGCCCTAAAGTCAGTGGCGGTGCCATCCGGCTC
CGAGCGTAGTAATTCTTCTCGCTCTGGAGACCCGGCTGtgtgCTTGCCAGCAACCccccAATT
ttttAAGGTTGACCTCGGATCAGGTAGGGATACCCGCTGAACTTAA
k__Fungi 1.000

>OTU_156
AAAATGCGATAGCTGGTGTGAATTGCAGGACACTACGAGCACCGAGTATTCGAACGCATATG

GCGGTTTCGGGTCTAGCCCGTAACCACGTCTGTCTGAGGGTCGTTCCCAAGCATGCAAAAGG
TTTTCCTTtttGGTTGCATCGGAAGATTCCCAATTGGATTCCGTCAACCCAAGAACGGAGCA
TCCTCGTTCGTAGAAGGTGTCAAATCCGCGGGGAACAAATCTCCCTGAAATGCGGAGGAGCA
CTGGAACTGGGGTTCTCCAGAAGGAACTACGTCGTCTCCGACCTCAGATCAGGCGAGGACAC
CCGCTGAATTTAA
No similar sequence

>OTU_157
GAAATGCGATAAGTAATGTGAATTGCAGAATTCAGTGAATCATCGAATCTTTGAACGCACAT
TGCGCCCTTTGGCATTCCGAGGGGCATGCCCGTTCGAGCGTCATTAAAACCCCTCAAGCCTA
GCTTGGTGTTGGGTGTCGGGGCCccccccGCACCTCTATATCGCCGGCTGGCCGTCGTCTGAC
TTAGCGTTGTGACACCATGTCGCTGATGCCAGACGCGGACTGCGCCGTTAGAACTctctTGA
ATGTCCCCGGACGTTCAGTACAGGTTGACCTCGGATCGGGTAGGAATACCCGCTGAACTTAA
k__Fungi;p__Ascomycota;c__Dothideomycetes;o__Capnodiales;f__Te
ratosphaeriaceae;g__Devriesia;s__Devriesia_strelitziicola 0.920

>OTU_158
GAAATGCGATACTTGGTGTGAATTGCAGAATCCCGTGAACCATCGAGTTtttGAACGCAAGT
TGCGCCCGAAGCCATTAGGTTGAGGGCACGTCTGCCTGGGCGTCACGCATCTAGTCGCCACC
cccTCTCGTAATTACGGAGTGGAGTGGCGGATGTTGGCCTCCCGTGTTCCTGAGGCGCGGCT
GGCCTAAATTTGAGTCTTCGACGCGAGATGTCACGGCAAGTGGTGGTTGAAATCATCAACTC
GTGTGCTGTCGCGACCACTCCCGGCGCAGACTCCATCGACCCTAGAGTCCTCGACCCAAATT
TGAGTTGCGGGCCTTCGACTGCGACCCCAGGTCAGGCGGGATTACCCGCTGAGTTTAA
Psychotria asiatica

>OTU_159
GAAATGCGATAACTAGTGTGAATTGCAGAATTCCGTGAATCATCGAGTCTTTGAACGCACAT
TGCGCCcccTGGTATTCCGGggggCATGCCTGTCCGAGCGTCATTGCTGCCCATCAAGCACG
GCTTGtgtgtTGGGTCGTCGTCCCCTCTCCGGgggggACGGGCCCCAAAGGCAGCGGCGGCA
CCGCGTCCGATCCTCGAGCGTATGGGGCTTTGTCACCCGCTCTGTAGGCCCAGCCGGCGCTT
GCCGAACGCAAATCAATCTTTTCCAGGTTGACCTCGGATCAGGTAGGGATACCCGCTGAACT
TAAGCATATCAATAAGCGGAGGACATCGATGAAGAACGCAGCATCGATGAAGAAC
k__Fungi;p__Ascomycota;c__Eurotiomycetes;o__Eurotiales;f__Tric
hocomaceae;g__Aspergillus;s__Aspergillus_flavus 1.000

>OTU_160
GAAATGCGATAAGTAATGTGAATTGCAGAATTCAGTGAATCATCGAATCTTTGAACGCACAT
TGCGCCCTTTGGTATTCCGAAGGGCATGCCTGTTCGAGCGTCATTATCAACCATCAAGCTCT
GCTTGGCATTGGGTGTCAACCTCCCCTAACCGGGCGGTCGCGCCTCAAACTGTTCGGCGGTG
GCTCAGGGCCTCAAGCGTTAGTAATACTTCCCGCTTCAGAGAAACTGAGTTGCCTGCCTCTA
GAAACCCACATCTTAAGGTTGACCTCGGATCAGGTAGGGATACCCGCTGAACTTAA
Uncultured fungus

>OTU_161

AAAGTGCGATAACTAGTGTGAATTGCATATTCAGTGAATCATCGAGTCTTTGAACGCAGCTT
GCACTCTATGGTTtttCTATAGAGTACGCCTGCTTCAGTATCATCACAAACCCAcacaTAAC
ATTTGTTTATGTGGTGATGGGTCGCATCGCTGTTTTATTACAGTGAGCACCTAAAATGtgtg
tgATTTTCTGTCTGGCTTGCTAGGCAGGAATATTACGCTGGTCTCAGGATCTTtttttttGG
TTCGCCCAGGAAGTAAAGTACAAGAGTATAATCCAGCAACTTTCAAACTATGATCTGAAGTC
AGGTGGGATTACCCGCTGAACTTAAGCATATCAATAAGCGGAGGATGAGTCCGTAGGTGAAC
CTGCGGAGCGGAGGAC
k__Fungi;p__Zygomycota;c__Incertae_sedis;o__Mucorales;f__Mucor
aceae;g__Rhizopus;s__Rhizopus_oryzae 1.000

>OTU_162
AAACTGCGATAAGCAATGCGAACCGCAGTGTCTCGTGAGTCATCCGGTATTCGAACGCACAT
GGCAGGTCCTCGGACCTACGTCTGGTTGAGGGTCATATGGACTACTCATGGAGTGATGTGGC
ACGGCGCTTGCCGTGCTCAAAGGCATGGCGCAGGGCTGCTTGGTGGACAATCAGGTGGCCAT
GCACTGCCCGCAGTAGCGTGGGCATTGATGCTTGACCTCAACTCAGACGAGACGACCCGCTG
AACTTAA
Uncultured fungus

>OTU_163
GATCCGCGATACGTCCTGGAAGCCGCCGTGAACCATCAATTTTCGAACGCACATTGCATACA
GGGAGGGTGGGGTGAGTCTACTCTCACAATCGTGGTTTAAATACAAAGGATGACTAATAAAA
CCGTCACCCCTCATTTTCTCCcccGCCcccTCAGATTACCCGCTGAACTTAAGCATATCAAT
AAGCGGAGGAGCATATCAATAAGCGGAGGAGCATATCAATAAGCGGAGGAGCATATCAATAA
GCGGAGGAGCATATCAATAAGCGGAGGA
k__Fungi;p__Ascomycota;c__Saccharomycetes;o__Saccharomycetales
;f__Phaffomycetaceae;g__Komagataella;s__Pichia_pastoris 1.000

>OTU_164
GAAATGCGATAAGTAATGTGAATTGCAGAATTCAGTGAATCATCGAATCTTTGAACGCACCT
TGCGCTCCCTGGTATTCCGGGGAGCATGCCTGTTTGAGTGTCATGAAAACCCTCAACCTTAG
ATTGGTTAACACCTTTCTTTGGCTTGGATTTGGACGTTTGCCGATGATAAGTCGGCTCGTCT
TAAAAGTAATAGCTGGATCTGTCTCGCGACATGGTTTGACTTGGCGTAATAAGTATTTCGCT
AAGGACATCTTCGGATGGCCGCGTTGCAAGACTAAAGACCGCTTTCTAATCCATTGATCTTC
GGATTAATATTCTTGACTCTGGCCTGAAATCAGGTAGGACTACCCGCTGAACTTAA
k__Fungi;p__Basidiomycota;c__Tremellomycetes;o__Filobasidiales
;f__mitosporic
Filobasidiales;g__Cryptococcus;s__Cryptococcus_diffluens 1.000

>OTU_165
ACAATCGGGACGGCCGGTGCCTGGGCCGCGATGATGCGGTCGACCGCGTGCCGCTCCCGCCA
TGCGAGACTTACGGATTCGGATGTCCTGTCATCCGGTACGAAAGCGATGACGGACGGTCCCG
AGCCCGAGACGAATGCGTGGCTTGCACCAGCCTCAAGCGCCGCTTCGATGGCCTGCCCGCTA
CGGGGATGCAGTGAGATCGCAGCCTGCTGCAGATGATTGTCATCACCTTCTCCGGCGCCGAT
CCGATCGAATGTCGCATATCAATAAGCGGAGGAGTATAGG

Bifidobacterium dentium

>OTU_166
AAAATGCGATAAGTAGTGTGAATTGCAGAATTCAGTGAATCATCGAATCTTTGAACGCACAT
TGCGCCCCTTGGTATTCCATGGGGCATGCCTGTTCGAGCGTCATTTGTACCTTCAAGCTTTG
CTTGGTGTTGGGTGTTTGTCCTGAGGGACTCGCCTTAAAGTAATTGGCAGCCAGTGTTTGGT
TTTGAAGCGCAGCACAAGTCGCGATTCAAAGCTATACGCCCGCTTCCACAAGCCTTtttCAC
TTTTGACCTCGGATCAGGTAGGGATACCCGCTGAACTTAA
k__Fungi;p__ Ascomycota;c__ Dothideomycetes;o__ Pleosporales;
f__Phaeosphaeriaceae;g__Paraphoma;s__Paraphoma_chrysanthemicol
a 1.000

>OTU_167
GAAATGCGATAAGTAGTGTGAATTGCAGAATTCAGTGAATCATCGAATCTTTGAACGCACAT
TGCGCCTCCTGGTATTCCGGGAGGCATGCCTGTTCGAGCGTCATTAAaaaCCACTCAAGCTC
TTTTGCTTGGTATTGGAAGATGAGTATGCTTTGTTATACTCACTTTCGAAATTCAAAGGCGT
AGAGCCCCACGTGCCACGGCGTAGTAAGTTTTCTTTCGCTTGGAATGTGAGGTGATATTGCC
ACAAACCcccAATTtttCTAGGTTGACCTCGGATCAGGTAGGGATACCCGCTGAACTTAA
k__Fungi;p__Ascomycota;c__Pezizomycetes;o__Pezizales;f__Pyrone
mataceae;g__Pseudaleuria;s__Pseudaleuria sp. MF-3 1.000

>OTU_168
GAAATGCGATAACTAATGTGAATTGCAGAATTCAGTGAATCATCGAGTCTTTGAACGCACAT
TGCGCCcccTGGTATTCCGGggggCATGCCTGTCCGAGCGTCATTGCTGCCCTCAAGCCCGG
CTTGtgtgtTGGGCCCTCGTCCcccGGCTCCCGGgggACGGGCCCGAAAGGCAGCGGCGGCA
CCGCGTCCGGTCCTCGAGCGTATGGGGCTTCGTCTTCCGCTCCGTAGGCCCGGCCGGCGCCC
GCCGACGCATTTATTTGCAACTTGTTtttttCCAGGTTGACCTCGGATCAGGTAGGGATACC
CGCTGAACTTAA
k__Fungi;p__Ascomycota;c__Eurotiomycetes;o__Eurotiales;f__Tric
hocomaceae;g__Aspergillus;s__Aspergillus_terreus 1.000

>OTU_169
GAAATGCGATAAGTAATGTGAATTGCAGATACAGTGAATCATCGAATCTTTGAACGCATATT
GCACCTTTTGGTATTCCATAAGGTACGTCTGTTTGAGCGTCGCGAACATCTCATAATTAATG
AATTtttttttGTTAATTATGGTCTTTGAGTTTGTCTATAATTtttAGACTCACTTTAAATT
GATTAGTAGTTTAACTTTTGAAAGGGTTAAAATTAGGTGTTttttAATGTACATTACTTTGT
GCATCATCTAATCAAGAGTTACTTACTCTGCCTTAGTATTAATGTTACTGCTTCTAATAGCT
TATTAAGCAAGTAATATTGCAATCGACCTCAAATCAGATGGGATTACCCGCTGAACTTAAGC
ATATCAATAAGCGGAGGAATCGTGCCTGAATGTTGACCTCGGATCAGGTAGGAATACCCGCT
GAACTTAA
Uncultured fungus

>OTU_170
GAAATGCGATACTTGGTGTGAATTGCAGAATCCCGTGAACCATCGAGTCTTTGAACGCAAGT

TGCGCCCGAAGCCCTTAGGCTGAGGGCACGCCTGCCTGGGTGTCACCAAAAGGCGCCccccc
GTCTCGCCCGTCCCAGGGCACGGGGAGGgggCGAACGTTGGCCTCCCGGGAGCCCCTGGCTC
GCGGTTGGTTCAAAGACTCGATGGTGGTCGAGAACAACCCTCGTGGCCAGTCGcgcgcgcCT
CTCCcccGGTTCAAGGCACGGCGACCCGCGGGCGACGTGGATCGTCCCGAGCGCGACCTCAG
GTCAGGCGGGGCTACCCGCTGAGTTTAA
Arachis hypogaea

>OTU_171
GAAATGCGATAAGTAATGTGAATTGCAGAATTCAGTGAATCATCGAATCTTTGAACGCACAT
TGCGCCCCTTGGTATTCCGGggggCATGCCTGTTCGAGCGTCATTATAACCCTCAAGCTCAG
CTTGGTGTTGGGGCATACCTCCGGGTAGCTCTCAAAATCAGTGGCAGTGCCTCTCGGCTCTA
AGCGTAGTAATACTCCTCGCTATGGAGTCCGGTAGGTTCGCGCCAGAAACCCTAATTTTCTA
TGGTTGACCTCGGATCAGGTAGGGATACCCGCTGAACTTAA
Uncultured fungus

>OTU_172
GAAATGCGATAAGTAATGTGAATTGCAGAATTCAGTGAATCATCGAATCTTTGAACGCACAT
TGCGCCCCTTGGTATTCCGGggggCATGCCTGTTCGAGCGTCATTATAACCCTCAAGCCTAG
CTTGGTGTTGGAGCATGCTACCTAGCAGCTCTTAAAATCAGTGGCAGTGCCCTTCGGCTCTA
AGCGTAGTAACACCTCTCGCTATGGAAACCGGATGGTACGCGCCAGAACCcccAACTTTCTA
TGGTTGACCTCGGATCAGGTAGGGATACCCGCTGAACTTAA
Uncultured *Helotiales*

>OTU_173
AAAGTGCGATAATTATTGCGACTTGCATTCATAGTGAATCATCGAGTTCTTGAACGCATCTT
GCGCCTAGTAGTCAATCTACTAGGCACAGTTGTTTCAGTATCATCCTCACCAATCAACCCAA
CTTTGGGTTGGAACTGGACTCTTGACAGCATTCAGTTGCTCTCATGGTCTTAAATACCAAGT
CTTTGTGCACTctctctAGAGTGAGTGTACGGGTGGAAATTttttAAACAGTGAGCAACGAG
CTTCTTGGGTTGTGGGAACCCCAAGTGTTAGTCTTTGAGCGTCGACCTGGTTTCCGACTTGA
AACGACTTGAGAATGCTTCGCATTCAAGACTGAAaaaTCTCACAATCTTGATCTGAAATCAA
CTGAGACTACCCGCTGAACTTAA
k__Fungi;p__Zygomycota;c__Incertae_sedis;o__Mucorales;f__Licht
heimiaceae;g__Lichtheimia 1.000

>OTU_174
GAAATGCGATAACTAGTGTGAATTGCAGAATTCCGTGAATCATCGAGTCTTTGAACGCACAT
TGCGCCccccTGGTATTCCGGggggCATGCCTGTCCGAGCGTCATTGCTGCCCATCAAGCACG
GCTTGtgtgtTGGGCTTCCGTCCCTGGTAACGGGGACGGGCCCAAAAGGCAGTGGCGGCACC
ATGTCTGGTCCTCGAGCGTATGGGAAGCAACTCTTtttGTCACCCGCTCCCGTAGGTCCAGC
TGGCAGCTAGCCTCGCAACCAATCTTtttAACCAGGTTGACCTCGGATCAGGTAGGGATACC
CGCTGAACTTAA
k__Fungi;p__Ascomycota;c__Eurotiomycetes;o__Eurotiales;f__Tric
hocomaceae;g__Eurotium;s__Eurotium_niveoglaucum 0.910

>OTU_175
GAAATGCGATAACTAATGTGAATTGCAGAATTCAGTGAATCATCGAGTCTTTGAACGCACAT
TGCGCCCTCTGGTATTCCGGAGGGCATGCCTGTCCGAGCGTCATTGCTGCCCTCAAGCACGG
CTTGtgtgtTGGGCCcccGCCccccccGCACCGGgggggCGGGCCCGAAAGGCAGCGGCGGCA
CCGCGTCCGGTCCTCGAGCGTATGGGGCTTCGTCACCCGCTCTTGTAGGCCCGGCCGGCGCC
AGCCGACCccccTCAATCTATTttttCAGGTTGACCTCGGATCAGGTAGGGATACCCGCTGA
ACTTAA
k__Fungi;p__Ascomycota;c__Eurotiomycetes;o__Eurotiales;f__Tric
hocomaceae;g__Penicillium;s__Penicillium_terrigenum 1.000

>OTU_176
GAAATGCGATAAGTAATGTGAATTGCAGAATTCAGTGAATCATCGAATCTTTGAACGCACAT
TGCGCCCGCCAGTATTCTGGCGGGCATGCCTGTCTGAGCGTCATTTCAACCCTCATGCCCCT
AGGGCGTGGTGTTGGGGATCGGCCAAAGCCCGCGAGGGACGGCCGGCCCCTAAATCTAGTGG
CGGACCCTTCGTGGCCTCCTCTGCGAAGTAGTGATATTCCGCATCGGAGAGCGATGAGCCCC
TGCCGTTAAACCcccAACTTTCTAAGGTTGACCTCAGATCAGGTAGGAATACCCGCTGAACT
TAAGCATATCAATAAGCGGAGGATGAGTCCGTAGGTGAACCTGCGGCGAGGCGGAAaaaCGC
GCTAAAGTTTGTG
k__Fungi;p__Ascomycota;c__Sordariomycetes;o__Hypocreales;f__Bi
onectriaceae;g__ Clonostachys;s__Clonostachys_rosea 0.990

>OTU_177
GAAATGCGATAAGTAATGTGAATTGCAGATACAGTGAATCATCGAATCTTTGAACGCACATT
GCGCCCGCCAGTATTCTGGCGGGCATGCCTGTTCGAGCGTCATTTCAACCCTCAAGCACAGC
TTGGTGTTGGGACTCGCGTTAATTCGCGTTCCCCAAATCGATTGGCGGTCACGTCGAGCTTC
CATAGCGTAGTAATCATACACCTCGTTACTGGTAATCGTCGCGGCCACGCCGTTAAACCCCA
ACTTCTGAATGTTGACCTCGGATCAGGTAGGAATACCCGCTGAACTTAA
k__Fungi;p__Ascomycota;c__Sordariomycetes;o__Hypocreales;f__Ne
ctriaceae;g__Fusarium 0.930

>OTU_178
GAAATGCGATAAGTAATGTGAATTGCAGAATTCAGTGAATCATCGAATCTTTGAACGCACAT
TGCGCCCCTTGGTATTCCATGGGGCATGCCTGTTCGAGCGTCATCTACACCCTCAAGCTCTG
CTTGGTGTTGGGCGTCTGTCCCGCCTCTGCGCGTGGACTCGCCTCAAAATCATTGGCAGCGG
TCCTTGCCTCCTCTCGCGCAGCACATTGCGCTTCTCGAGGGGCGTGGGTCGCGTCCACGAAG
CAACATTACCGTCTTTGACCTCGGATCAGGTAGGGATACCCGCTGAACTTAA
k__Fungi;p__Ascomycota;c__ Dothideomycetes;o__ Pleosporales;f__
Didymosphaeriaceae;g__ Paraconiothyrium;s__ Paraconiothyrium
brasiliense 0.990

>OTU_179
GAAATGCGATAAGTAATGTGAATTGCAGAATTCAGTGAATCATCGAATCTTTGAACGCACAT
TGCGCCCCTTGGTATTCCGGggggCATGCCTGTTCGAGCGTCATTATAACCCTCAAGCCTAG
CTTGGTGTTGGAGCATGCTACCTAGCAGCTCTTAAAATCAGTGGCAGTGCCCTCTGGCTCTA

AGCGTAGTAACTCTTCTCGCTATGGAACCCAGAGGGACCGCGCCAAAACCcccAACTTCTAA
TGATTGACCTCGGATCAGGTAGGGATACCCGCTGAACTTAA
k__Fungi;p__Ascomycota;c__ Leotiomycetes;o__Helotiales;f__
Hyaloscyphaceae;g__Cistella;s__Cistella_albidolutea 0.970

>OTU_180
GAAATGCGATAAGTAGTGTGAATTGCAGAATTCAGCGAATCATCGAATCTTTGAACGCACAT
TGCGCCCTTCGGTTATTCCTTAGGGCATGCCTGTTCGAGCGTCATTTCAACCTTCAAGCCTG
GCTTGGTGTTGGGCGCTGTCCCGCCTCCGcgcgcgGACTCGCCCCAAATGAATTGGCAGTCG
CACCCTCCGAGCCGCGAGCGCAGCACAAGTCGcgcgGGCGGAACTCTGGggggACGGACGCT
CCACAAGACCCTTTCTCAGTCTTGACCTCGGATCAGGTAGGGATACCCGCTGAACTTAA
k__Fungi;p__Ascomycota;c__ Dothideomycetes;o__ Pleosporales;f__
Phaeosphaeriaceae;g__ Septoriella;s__ Septoriella_oudemansii
1.000

>OTU_181
GAAATGCGATAAGTAATGTGAATTGCAGAATTCAGTGAATCATCGAATCTTTGAACGCACAT
TGCGCCCCTTGGTATTCCGGggggCATGCCTGTTCGAGCGTCATTTCAACCCTCAAGCTTAG
CTTGGTATTGAGTCTATGTCAGTAATGGCAGGCTCTAAAATCAGTGGCGGCGCCGCTGGGTC
CTGAACGTAGTAATATCtctcGTTACAGGTTCTCGGTGTGCTTCTGCCAAAACCCAAATTtt
tCTATGGTTGACCTCGGATCAGGTAGGGATACCCGCTGAACTTAA
k__Fungi;p__Ascomycota;c__Leotiomycetes;o__Helotiales;f__Scler
otiniaceae;g__Botryotinia;s__Botryotinia_fuckeliana 0.980

>OTU_182
GAAATGCGATAAGTAATGTGAATTGCAGAATTCAGTGAATCATCGAATCTTTGAACGCACAT
TGCGCCCGCCAGCATTCTGGCGGGCATGCCTGTCCGAGCGTCATTTCCACCATCAAGCCCAC
GGGCTTGTGTTGGGGACCTGCGGCTGCCCGCAGGCCCCGAAAACCAGTGGCGGGGCCCGCTG
TCACCCCGAGCGTAGTAGTAAACCACCTCGCTCAGGGCGTGCTCCGGGTCCCGGCCGTTAAA
CGGCGCCcccccAAGGGCGCCACGAACCCCACAAGGTTGACCTCGGATCAGGTAGGAAGACC
CGCTGAACTTAA
k__Fungi;p__Ascomycota;c__Sordariomycetes;o__Sordariales;f__Ch
aetomiaceae 0.900

>OTU_183
GATCCGCGATACGTCCTGGAAGCCGCCGTGAACCATCAATTTTCGAACGCACATTGCATACA
GGGAGGGTGGGGTGAGTCTACTCTCACTATCGTGGTTTAAATACAAAGGATGACTAATACAA
TCGTCAGCCcccAAATTTTCTCCcccGCCcccTCAGATTACCCGCTGAACTTAAGCATATCA
ATAAGCGGAGGAGCATATCAATAAGCGGAGGAGCATATCAATAAGCGGAGGAGTCCGTAGGT
GAACCTGCGGCATATCAATAAGCGGAGGAGTCCGTAGGTGAACCTGCG
k__Fungi;p__Ascomycota;c__Saccharomycetes;o__Saccharomycetales
;f__Incertae_sedis;g__Candida;s__Candida_cellae 0.990

>OTU_184

GAAATGCGATAAGTAGTGTGAATTGCAGAATTCAGTGAATCATCGAATCTTTGAACGCACAT
TGCGCCCCTTGGTATTCCATGGGGCATGCCTGTTCGAGCGTCATTTGTACCCTCAAGCTCTG
CTTGGTGTTGGGTGTTTGTCCACTGTCGTGGACTCGCCTTAAAGTCATTGGCAGCCAGTGTT
TTGGTATTGAAGCGCAGCACATTTTGCGCCTCTAGCCTAGAGCACTCGCGTCCAGTAAGCCT
TtttCCACTTTTGACCTCGGATCAGGTAGGGATACCCGCTGAACTTAA
k__Fungi;p__Ascomycota;c__Dothideomycetes;o__Pleosporales;f__Leptosphaeriaceae;g__Leptosphaeria;s__Leptosphaeria_sp_SF96 1.000

>OTU_185
TAACTGCGATAAGTAGCGTGAATTGCAGACGCTTTGAACGTTAAACTTTCGAACGCACATTG
CGCCGTAGGAGTTCTACCCTGCGGCACATCTGGTTGAGGGTCGTGATCAAaaaCTGCCCGAA
TGTAGCAGTATAGATAGCTGGCGAACCATGATGAAAGTCGTGATTTGCCATATAACGCTATA
GAGGATAGTCTCCTGTACGGgggACTACCTGCCACACGGATGATCTACTAGTTGACGCAGTA
GGTCCCGGTCCCAGGTATTATGCACGAAACGTATTATGCCGAGGGACGTCAGGGATACGGCT
AGTGGgggAATCTGTGCGAGCTATATGAGCTATTCGATTTCCCGACCTCAACTCAGGTGTGA
TTACCCGCTGAACTTAA
Pratylenchus goodeyi

>OTU_186
AAAATGCGATAAGTAATGTGAATTGCAGAATTCAGTGAATCATCGAATCTTTGAACGCACAT
TGCGCCCGCTGGTATTCCGGCGGGCATGCCTGTTCGAGCGTCATTTCAACCCTCAAGCCccc
GGGTTTGGTGTTGGGGATCGGCTCTGCCTTCTGGCGGCGCCGCCcccGAAATACATTGGCGG
TCTCGCTGCAGCCTCCATTGCGTAGTAGCTAACACCTCGCAACTGGAACGCGGCGCGGCCAT
GCCGTAAAACCCCAACTTCTGAATGTTGACCTCGGATCAGGTAGGAATACCCGCTGAACTTA
A
k__Fungi;p__Ascomycota;c__Sordariomycetes;o__Hypocreales;f__Nectriaceae;g__Fusarium;s__Fusarium_flocciferum 1.000

>OTU_187
AGGTCGATACGGAAACCCGGTTTGAAGTTACGGTCCATCACCATCGGGGCTTTCGCATCCAG
CACGGTACTGCCCGCCAGACCGCCGCGAATAGCCTGATAAACCAGATCCGGATTGACGCCGG
CTTTAGTCGCCAGGGTCAGCGCTTCAGACATCGCCGCGATGTTCAGCGCGACAATCACCTGG
TTAGCCAGTTTGGTGACGTTGCCCGCGCCAATATCGCCGGTATGCACTACGGAGCCGGCCAT
CGCTTTCATCAGATCGTAGTATTTATCGAAGATCGCTTTATCGCCGCCGACCATGACCGACA
GCGTGCCGTCGATGGCTTTCGGTTCGCCGCCGCTCACCGGCGCATCCA
Klebsiella pneumoniae

>OTU_188
GAATTGCGTTAAGAATCACGAATTACAGATATTATGAGTGATATGTTTTCGATTGCATATTG
CATCGTTGGGCACTTGCCCATCGGTATACCCAACTCAGGGTgtgtATCACTAATCGGAAGAC
ACAATGCACTTGTTGGTTGTGTCTGAGCTTGGAGCTGTGCAATACCTGTGATTGCATTGTTT
TATTGAGGTGTGGCTGAATTGTAGAAATGAAGGAGAATGAGGTTGAAAATGCTTGGTGGTTG
TTGGAAGTTTAATGAATTtttGAGCTTTCGGTGACCGTTCGGTTtttCGACTTttttCAcac

aGCCTTGCATACACCAGCCAATTGTTtttGGTCTAATCCACCTGAGTCGGATATGATCACCC
GCCGAACTTAA
Aphelenchoides

>OTU_189
GAAATGCGATAAGTAATGCGAATTGCAGAATTTCCGTGAGTCATCGAATCTTTGAACGCACA
TTGCGCCCATTGGTATTCCGATGGGCATGCCTGTTCGAGCGTCATTATCCTCCCTCAAACCT
CGCGTTTGGTGTTGGACCGCGTCGGTTCTCCGTGACCGACGGGTCTCAAAGTCAATGACGGC
GTCCGTGGGACCCTCGGTGCAACGAGCTTTTCGGAGCACGCGTCGAGTCGAAAGGACCCTCC
GGGCCGGTCAGACCTTTCCATTttttATCAGGTTGACCTCGGATCAGGTAGGAATACCCGCT
GAACTTAA
k__Fungi 1.000

>OTU_190
GAAATGCGATAAGTAATGTGAATTGCAGATACAGTGAATCATCGAATCTTTGAACGCAAATG
GCACTCTATGGTATTCCGTAGAGTACGTCTGTTTGAGCGTCGCGAACATCTCCACAATTAGT
TttttttAATTAGTTGAGGGTTTTGAGGTTGTCATATAAACAATGACTCCCTTTAAAATAAT
TAGTGATGACCTTATGAATGGGTTAATACTGtgtgtTATAATGGATTACATCCATCACCAGT
CAGagagTAATCTCGCCTTAGTAATTTGTAGTGATTGCTTCTAACTGCCATTGGCAAACAAA
CTATGATCTGAAGTCAGGTGGGATTACCCGCTGAACTTAA
k__Fungi;p__Basidiomycota;c__Wallemiomycetes;o__Wallemiales;f_
_Wallemiaceae;g__Wallemia 1.000

>OTU_191
GAAATGCGATAACTAGTGTGAATTGCAGAATTCCGTGAATCATCGAGTCTTTGAACGCACAT
TGCGCCcccTGGTATTCCGGggggCATGCCTGTCCGAGCGTCATTGCTGCCCATCAAGCACG
GCTTGtgtgtTGGGCCcccGTCCcccTCTTttttAGGgggggggACGGGCCCGAAAGGCAG
CGGCGGCACCGTGTCCGGTCCTCGAGCGTATGGGGCTTTGTCACCCGCTCTGTAGGCCCGGC
CGGCGCTTGCCGAACGCAAATCAATCTTTTCCAGGTTGACCTCGGATCAGGTAGGGATACCC
GCTGAACTTAA
k__Fungi;p__Ascomycota;c__Eurotiomycetes;o__Eurotiales;f__Tric
hocomaceae;g__Aspergillus;s__Aspergillus_flavus 0.990

>OTU_192
GAAATGCGATAACTAGTGTGAATTGCAGAATTCCGTGAATCATCGAGTCTTTGAACGCACAT
TGCGCCcccTGGTATTCCGGggggCATGCCTGTCCGAGCGTCATTGCTGCCCATCAAGCACG
GCTTGtgtgtTGGGTCGTCGTCCcccTCTCCGGggggACGGGCCCGAAAGGCAGCGGCGGCA
CCGCGTCCGATCCTCGAGCGTATGGGGCTTTGTCACATGCTCTGTAGGATTGGCCGGCGCTT
GCCGAACGCAAATCAATCTTTTCCAGGTTGACCTCGGATCAGGTAGGGATACCCGCTGAACT
TAA
k__Fungi;p__Ascomycota;c__Eurotiomycetes;o__Eurotiales;f__Tric
hocomaceae;g__Aspergillus;s__Aspergillus_flavus 1.000

>OTU_193

GAAATGCGATAAGTAATGTGAATTGCAGAATTCAGTGAATCATCGAATCTTTGAACGCACCT
TGCGCTCCTTGGTATTCCGAGGAGCATGCCTGTTTGAGTGTCATGAAACTCTCACCCTCTAG
CTTTCTTAATCGTGGCTAGCGGCGTGGACGTGAGCGCTGCTGCTTTGTTGCGGCTCGCTCGA
AATGCATTAGCAGACCCTTTTCGTAATCGGTTCCACTCAACGTGATAAGTATTTCGTTGAGG
ACAGTTGCAGCAATGCGGCTGGCCGGGATAAGAAAGGCATAGTTGTCAGCTTCTAATCGCCC
TTGGGCAATTttttATGATCTGGCCTCAAATCAGGTAGGACTACCCGCTGAACTTAA
Uncultured fungus

>OTU_194
GAAATGCGATAAGTAGTGTGAATTGCAGAATTCAGTGAATCATCGAATCTTTGAACGCACAT
TGCGCCCTTTGGTATTCCTTAGGGCATGCCTGTTCGAGCGTCATCTAAACCTTCAAGCACTG
CTTGGTGTTGGGTGCCTGTCCCGCCcccGcgcgTGGACTCACCTCAAATCCATTGGCGGCCC
TCACGTCGGCTACGAGCGCAGCAGAAACGCGAACTCGTGGGCCCGGCGGGGTGGCTCCCAGA
AGCTACACTCACCATTTTGACCTCGGATCAGGTAGGGATACCCGCTGAACTTAA
k__Fungi;p__Ascomycota;c__Dothideomycetes;o__Pleosporales;f__S
porormiaceae;g__Preussia;S__Preussia_polymorpha 0.990

>OTU_195
GAAATGCGATAAGTAATGTGAATTGCAGAATTCAGTGAATCATCGAATCTTTGAACGCACAT
TGCGCCCGCCAGTATTCTGGCGGGCATGCCTGTCTGAGCGTCATTTCAACCCTCGGGACCCC
GTTCGCGGGACCCGGCGTTGGGGATCAGCCCTCTCCGGggggCTGGCCCCGAAATCTAGTGG
CGGTCCGCCAGCGATCTCCTCTGCGCAGTAGTTGATACCTCGCAGCTGGATAGCGGTCGGGC
CACGCCGTAAAACCcccAACTTCTCAAGGTTGACCTCAGATCAGGTAGGAATACCCGCTGAA
CTTAA
k__Fungi;p__Ascomycota;c__Sordariomycetes;o__Hypocreales;f__In
certae_sedis;g__Acremonium 0.990

>OTU_196
GAAATGCGATAAGTAATGTGAATTGCAGAATTCAGTGAATCATCGAATCTTTGAACGCATCT
TGCGCTCCTTGGTATTCCGAGGAGCATGCCTGTTTGAGTGTCATTAAAAGTTCTCAACCATT
CTGGTTtttttCCAGTGTGGCTTGGATTTGAGGGGTTTTGCAGGCTTTTGAACTGAAGTCGG
CTCCCTTGAAAAGGATTAGTGGTATCTGAGCAGAAGCCACCATGGGCGTGATAATTATCTAT
GTCTTGGTGAATGCCTGACCACAATAAGTGTTGTGTTTTGACGAATTTGACCTCAAATCAGG
TAGGACTACCCGCTGAACTTAA
k__Fungi;p__Basidiomycota;c__Agaricomycetes;o__Agaricales;f__I
nocybaceae;s__Inocybe_nitidiuscula 0.980
